# The variability of soils and vegetation of hydrothermal fields in the Valley of Geysers at Kamchatka Peninsula

**DOI:** 10.1038/s41598-021-90712-7

**Published:** 2021-05-26

**Authors:** I. N. Semenkov, G. V. Klink, M. P. Lebedeva, V. V. Krupskaya, M. S. Chernov, O. V. Dorzhieva, M. T. Kazinskiy, V. N. Sokolov, A. V. Zavadskaya

**Affiliations:** 1 Lomonosov Moscow State University, Moscow, Russia; 2 Institute for Information Transmission Problems (Kharkevich Institute) of the Russian Academy of Sciences, Moscow, Russia; 3 V.V. Dokuchaev Soil Science Institute, Moscow, Russia; 4 Institute of Geology of Ore Deposits, Petrography, Mineralogy and Geochemistry of the Russian Academy of Sciences (IGEM RAS), Moscow, Russia; 5 Kronotsky Federal Nature Biosphere Reserve, Elizovo, Russia

**Keywords:** Environmental chemistry, Mineralogy, Biodiversity, Plant ecology, Boreal ecology

## Abstract

The picturesque and high conservation value thermal landscapes of the Valley of Geysers feature endothermal (heated by endogenous fluids) soils which support endangered and unique species. However, such soils have not been distinguished as a separate taxon within most classification systems. In this study, we described the soil morphology at macro-, meso- and micro-scales, chemistry, mineralogy and vegetation of these landscapes as they are affected by the steam-heated acid-sulfate waters. The studied catenary sequence from exothermal (non-heated) to endothermal soils was characterized by decreasing contents of soil organic carbon, sand fraction, essential nutrients (Ca, K, Mg, Mn and Si), increasing soil acidity, amounts of fine particle-size fractions and contents of trace elements (Al, As, Co, Cr, Cu, Fe, Pb, Ti and V) as well as the development of sodium-sulfate salinity, kaolinization and ferrugination. In phytocenoses supported by endothermal soils, species of order *Rosales* and *Asparagales* were overrepresented among obligate and facultative thermophytes respectively, and species of order *Poales* were underrepresented among facultative thermophytes in relation to the flora of the Valley of Geysers. Phytocenoses on the non-heated Andosols were enriched in *Polypodiopsida* species. The results of our comparative analysis of the thermally-induced variability in the soils and vegetation contribute to the general understanding of mineralogical, bio-abiotic and biological systems affected by steam-heated acid-sulfate waters. We hope that our findings will provide a basis for future transdisciplinary studies of the influence of steam-heated waters of a hot spring on the thermal landscapes.

## Introduction

The picturesque thermal landscapes of the Valley of Geysers attract 1000–4100 tourists annually and are part of the UNESCO World Natural Heritage Site "Volcanoes of Kamchatka", which is one of 20 world natural heritage sites to meet all the qualifying criteria^1,2^. Areas, where hot fluids are discharged from the ground, can be used for obtaining renewable or ‘green’ energy. Such territories can also serve as natural laboratories to study the effects of increased CO_2_ concentrations in the soil atmosphere^3^ and climate warming^4^. Specific endothermal soils^5^ provide habitats for endangered, unique and protected plant species^1,4,6–9^ and microorganisms^10–14^. In Kamchatka’s thermal landscapes (where the soils have summer temperatures of more than 30 °C at a 50-cm depth^8^), the composition of vegetation and soil microbiota depends on changes in soil acidity, H_2_S concentration, temperature, salinity and concentrations of toxic compounds^4,6,7^. However, there are still many unresolved questions about the relationship between the plants and soils within steam hydrotherm areas and around hot springs. Endothermal soils are not distinguished as a separate taxon within most classification systems^15^ and their mineralogical and micromorphological characteristics^16–18^ as well as their associated plant communities^1,4,8^ have been insufficiently studied.

Unlike endothermal soils that experience the influence of steam-heated chloride and hydrocarbonate waters^19–22^, endothermal soils that are affected by acid-sulfate waters differ considerably from non-heated soils^1,5,18,23–25^. Their topsoils are depleted in Ca, Mg, Na, K and are intensively ferruginized up to the formation of large ferruginous nodules enriched in Al, Fe and Ti^18,23,26^. In their subsoils, a specific clayey variegated horizon is formed^18,23,24^—a probable analogue of the well-studied deep hydrothermal clays^27–29^ forming under higher pressure and temperature.

The aim of the present study is to close the transdisciplinary gap between pedology and botany within the zone of influence of steam-heated acid-sulfate waters of a hydrothermal field in the Valley of Geysers. We assume that the intensity of the effect of endogenous fluids on the soil matrix changes depends on the temperature, which affects the morphological and chemical properties and mineralogical composition of soils and the plant species composition.

## Results and discussion

### The catenary sequence of soils

**Figure 1:**
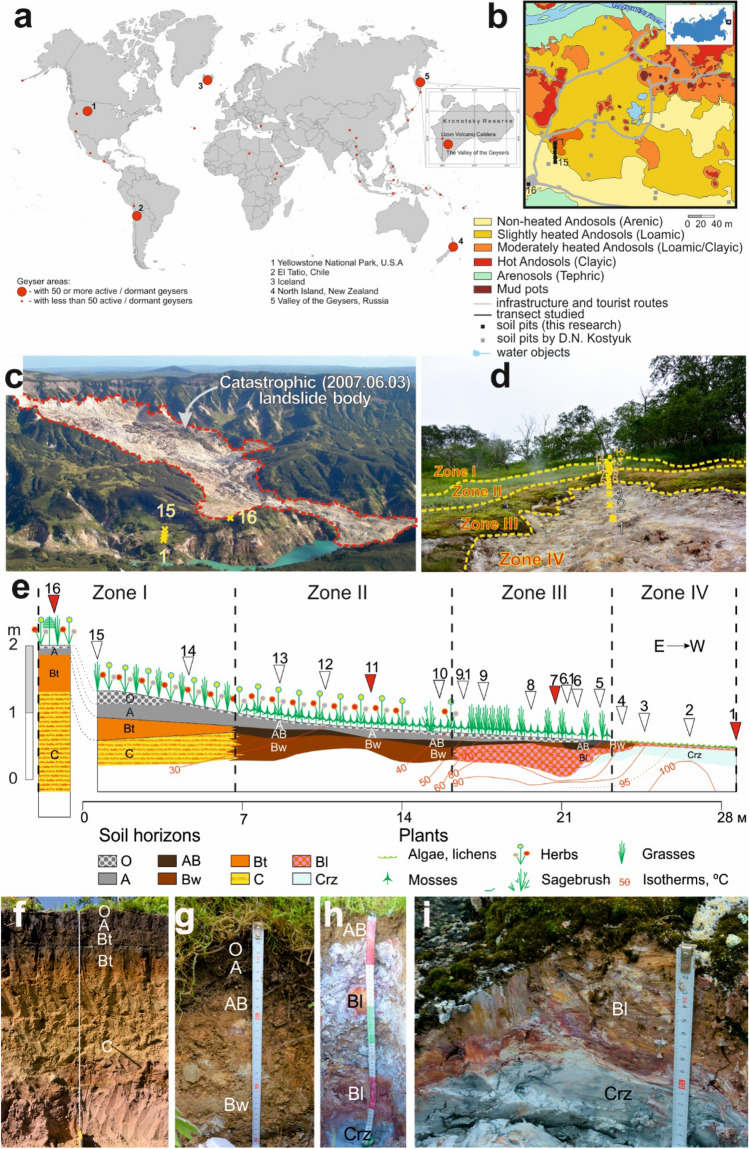
Location of study area (**a**–**c**), soil pits along the catena (**d**, **e**) and photos of soil pits (**f**–**i**). (**a b**) Study area location. Soil map is from^133^ (open access) with additions and corrections by I.N. Semenkov based on the map of soil temperature at a depth of 15 cm in the Valley of Geysers^131^ and the soil names from^44^ using CorelDraw X7 software (https://www.coreldraw.com/). (**c**) Top view of the left side of the Geysernaya River with the body of a catastrophic landslide, a visitor center (in the left lower part) and the location of the transect studied (1–15). (**d**) The location of transect studied. (**e**) A schematic profile of the catena studied with numbered soil pits. The main soil pits selected for comprehensive analyses (see section ‘Soil analyses’) are in red. (**f**) Non-heated Eutrosilic Silandic Andosols (Arenic, Cutanic) on pyroclastic material (pit no 16, Zone I), within levelled parts of the interfluve, under tall-herb meadow communities with local patches of Erman’s birch woods. (**g**) Slightly heated Eutrosilic Aluandic Andosols (Cutanic, Loamic, Natric) in the upper part of the catena, on hydrothermally altered sandy-loamy pyroclastic material (pit no 12, Zone II), on slightly heated slopes, under tall-herb meadows. (**h**) Moderately heated Eutrosilic Gleyic Aluandic Andosols (Loamic, Reductic, Protosalic, Hyperthionic) in the middle part of the catena, on hydrothermally altered clayey pyroclastic material (pit no 9.1, Zone III), under different moss and ‘microzonal’ communities. (**i**) Hot Gleyic Aluandic Andosols (Clayic, Reductic, Salic, Hyperthionic) in the lower part of the catena, on hydrothermal clays (pit no 4, Zone IV), on most heated bare slopes.

The catena of Andosols down a slope near a hot spring in the Valley of Geysers was subdivided into four thermal zones (Fig. 1a–e), which are described below.

#### Zone I. Non-heated Eutrosilic Silandic Andosols (Arenic, Cutanic) under Kamchatka’s tall herb communities and fragmented Erman’s birch woods

**Figure 2:**
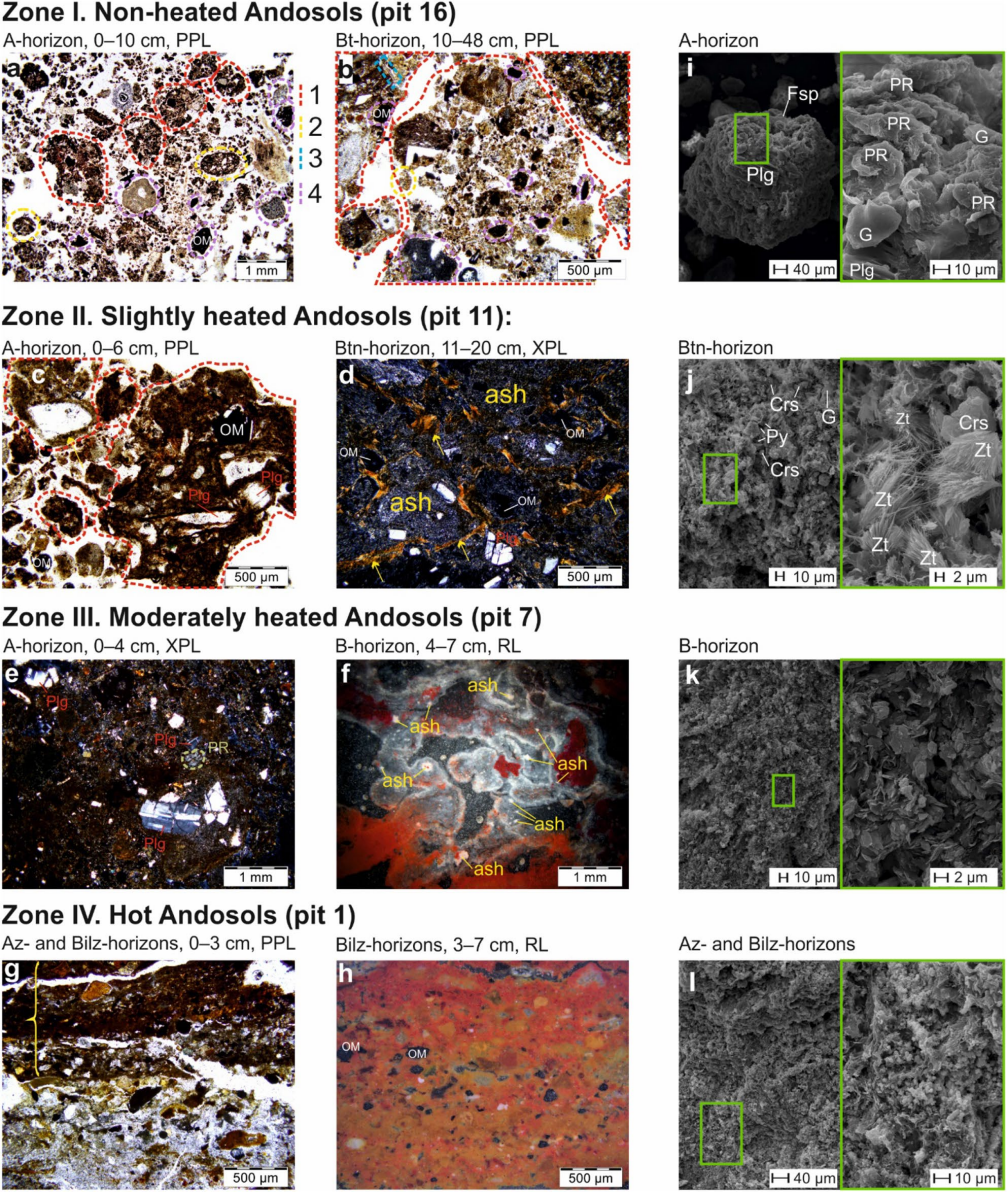
Soil Fabric Of Andosols In The Valley of Geysers. Two left rows—optical microscopy images (plane polarized light—PPL; cross polarized light—XPL; reflected light—RL; photos by M.P. Lebedeva). Right row (photos by M.S. Chernov): i—l—SEM images at two magnifications: on the left—a general view of a microaggregate, on the right—a detailed image of the surface: (**a**) Loose, porous, microaggregated material with fragments of deeply altered rocks and minerals, brown microaggregates (peds—1) and coprolites of soil micro- and mesofauna (2), fragments of semidecayed plant remains (3). (**b**) Inclusions of deeply altered rocks and minerals (4) within a subangular blocky microaggregates (1) with Fe-humus-clay microconcentrations and small coprolites of mesofauna (2). (**c**) microaggregates (1) of different sizes consisting of a brown collomorphic organomineral material and a finely-dispersed material that binds fragments of plagioclases (Plg), ore minerals (OM), with coatings of a collomorphic material (arrow). (**d**) Light-colored particles of argillized ash, with layered brownish yellow Fe-clay coatings (arrows) within intrapedal pores, singular small cracked grains of plagioclases (Plg) and ore minerals. (**e**) Granular, highly porous, brown material consisting of deeply altered fragments of volcanogenic minerals, assimilation of cracked sand-sized grains of plagioclases (Plg) and silt-sized grains of pyroxenes (PR). (**f**) A highly porous zone with rounded argillized ash particles with dark coatings and zones of iron hydroxide concentrations. (**g**) The uppermost part of a biogenic crust with microlayers of brown collomorphic material (brace) and yellow-white fine-crystalline material with charred particles as well as silty-collomorphic material with inclusions of silt-sized grains. (**h**) A red-colored zone with an intense intrusive impregnation by collomorphic Fe, with yellow altered argillized minerals and abundant charred particles and fine-silt-sized ore minerals (OM). (**i**) Sand- and silt-sized organomineral microaggregates consisting of volcanic glass (G) fragments (volcanic ash or tephra particles), crystals of plagioclases (Plg) and potassium feldspars (Fsp), microaggregates of clay particles and organic matter, with traces of activity of soil microorganisms and plant remains. (**j**) A non-oriented mass of densely packed silt–clay microaggregates consisting of kaolinite, smectite, mixed-layered clay minerals and fragments of primary volcanic minerals such as volcanic glass with intrapedal pores fully filled by newly formed (hydrothermal) minerals including pyrite, cristobalite and, presumably, zeolite (acicular crystals of zeolite minerals). (**k**) Kaolinite microaggregates with ‘domain-like’ microstructure in a non-oriented clay matrix. (**l**) A dense groundmass consisting of kaolinite and kaolinite-smectite mixed layered minerals microaggregates.

Non-heated Andosols with temperatures of < 25 °C at the depth of 15 cm were stratified (Table S1, Figure S1). In the sandy A-horizon (Fig. 1f), the micromass of soil fabric was well-structured due to highly active micro- and mesofauna and contained numerous brownish coprolites typical for Andosols^30,31^. Both modern and buried A-horizons showed a granular–angular blocky structure and contained plant remains, oval-shaped dense biogenic aggregates and small concentrations of brown collomorphic Al–Fe-humus material (Fig. 2a).

The Bt-horizon had a massive macrostructure (Table S2) and a pellicular grain microstructure consisting of microaggregates of loose consistency (containing silt-sized grains of plagioclases, weathered volcanic ash and heavy minerals^32^) covered with collomorphic Fe-humus coatings (Fig. 2b). The soil horizon buried under volcanic ash contained many sand-sized opaque and optically isotropic minerals (opal/cristobalite, volcanic glass). The coagulation of finely-dispersed ferruginous and collomorphic substances resulted from ‘intra-soil metamorphism’^33^ or ‘andozolization’^34^—the main pedogenic process in non-heated Andosols. Neither illuvial clay coatings nor unaltered volcanogenic sand-silt material were present.

In the topsoil (0–10 cm), the particle-size distribution (Table S3) was characterized by the prevalence of PM_>50_ with very small amounts of PM_5–50_ (< 10%) and PM_<1_ (0.5%), while the subsoil (Table S4) had higher contents of PM_<5_ and PM_10–50_ (abbreviations of particle-size fractions are explained in Methods), lower contents of PM_500–1000_ and similar contents of PM_50–250_ (Table S5). With depth, pH increased from 5.7 to 6.8 and redox potential (Eh) from − 35 to + 497 mV, while the value of electrical conductivity (EC) and the content of soil organic carbon (SOC) decreased by 2 and 30 times, respectively. A low EC value reflected an absence of salinization in non-heated Andosols. A high SOC content and a weakly acid reaction in the topsoil were favorable for the formation of Al-organic complexes and unfavorable for allophane formation^33,35^.

**Figure 3:**
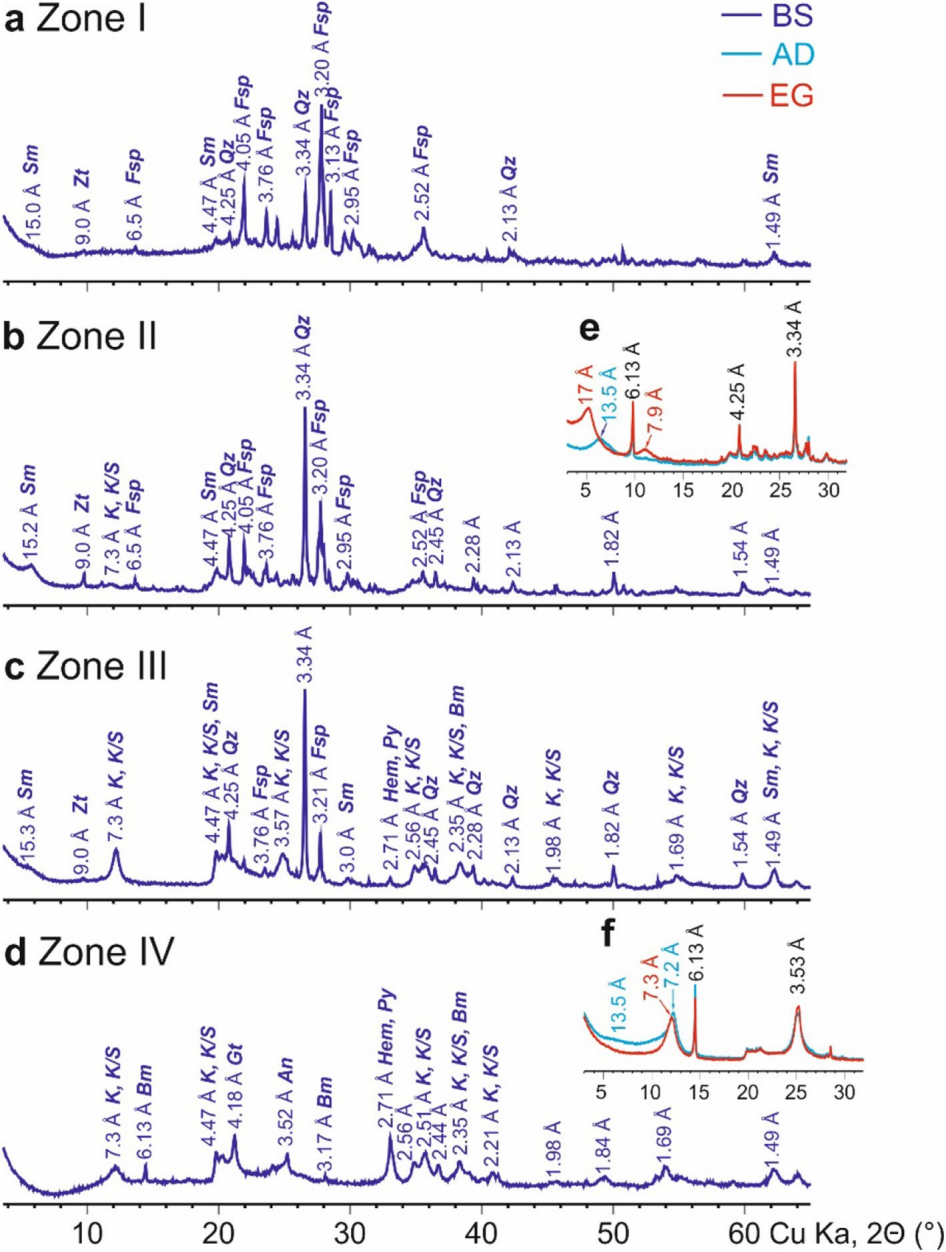
X-Ray diffraction patterns: (**a**–**d**) Bulk samples (BS) of Andosols from zones I (**a**), II (**b**), III (**c**), IV (**d**). (**e**, **f**) Fragments of XRD patterns from oriented ≤ 1 µm samples in air-draied (AD) and ethylene-glycole solvated (EG) states for Andosols from zones II (**e**) and IV (**f**). Crystal lattice planes in Angstroms. The main reflections represent the following minerals: *Sm*—smectite, *Zt*—zeolites, *K*—kaolinite, *K/S*—kaolinite-smectite mixed layered minerals, *Bm*—boehmite, *Gt*—goethite, *Qz*—quartz, *Fs*—feldspars, *Hem*—hematite, *Py*—pyrite.

In the Zone I soils, feldspars (albite-anorthite) were the main primary minerals constituents with cristobalite and rarer zeolites presented in smaller amounts. The non-heated Andosols also contained clay minerals, predominantly swelling minerals—dioctahedral smectites (probably montmorillonites). Smectites were unambiguously diagnosed by the X-ray diffraction patterns obtained from both powder and oriented air-dried and saturated with ethylene glycol specimens (Fig. 3a,e).

The upper 2 m-thick layer of non-heated Andosols had the mineralogical composition typical for this soil type^27,36^ and parent materials in the Valley of Geysers^32^, with predominant feldspars and smectite, frequent kaolinite, zeolites and occasional quartz, anatase, pyrite and goethite (Table S6). According to SEM and XRD data, the mineralogical and elemental composition changed insignificantly with depth, except for the appearance of newly formed opal and rare fragments of etched volcanic glass at a depth of more than 15 cm (Fig. 2i).

#### Zone II. Slightly heated Eutrosilic Aluandic Andosols (Cutanic, Loamic, Natric) under tall herb communities at the periphery of the steam hydrotherm

Slightly heated Andosols had temperatures of 25–40 °C at a depth of 15 cm (Table 1). Their A-horizons (Fig. 1g) was better structured as compared to that of non-heated Andosols, which was due to higher contents of collomorphic Fe-organic material and clays (Table S7). Excrements of soil microbiota occurred within some microzones of the topsoil. Dense silt–clay microaggregates contained inclusions of volcanogenic rock fragments, clay minerals and ash and had a random packing, according to SEM data.

**Table 1:** Main groups of vegetation and soils studied.

Geothermal features (soil pits)	Vegetation			Soils	*T*, °C at depths of 15/50 cm
	Picture	Communities	Protected species		
Zone I Non-heated. Without geothermal influences, soils warmed only by solar power (14–16)		Tall herb communities and local areas of *Betula ermanii*	*Lycopus uniflorus*	Non-heated Eutrosilic Silandic Andosols (Arenic) of smectite-plagioclase composition on monogenous sandy pyroclastic material	< 25/ < 30
Zone II Slightly heated. Little inflow of hot fluids (10–13)		Modified tall herb communities	*Agrostis geminate, Ophioglossum thermale, (Fimbristylis ochotensis), Lycopus uniflorus*	Slightly heated Eutrosilic Aluandic Andosols (Loamic, Natric) of feldspar-quartz-plagioclase-smectite composition on hydrothermally altered heterogenous sandy-loamy pyroclastic material	25–40/34–50
Zone III Moderately heated. Intensive inflow of hot fluids (4–9)		Predominantly *Agrostis geminata* and fragmentary *Fimbristyleta ochotensis* formations, moss communities	*Agrostis geminate, Fimbristylis ochotensis, Lycopus uniflorus*	Moderately heated Eutrosilic Gleyic Aluandic Andosols (Loamic, Reductic, Protosalic, Hyperthionic) of kaolinite-smectite composition on hydrothermally altered clayey pyroclastic material	40–80/80–98
Zone IV Hot spot. Very intensive inflow of hot fluids (1–4)		Primitive vegetation, moss communities	*Agrostis geminate, Fimbristylis ochotensis, Lycopus uniflorus*	Hot Gleyic Aluandic Andosols (Clayic, Reductic, Salic, Hyperthionic) of kaolinite composition on hydrothermal clays	80–100/ > 98

Photos by A.V. Zavadskaya.

The hydrothermally-induced alteration of slightly heated Andosols caused considerable changes in soil fabric (Fig. 2c,d). In the Btn-horizon, the number and distinctness of clayey layers of different colors consisting of a brown collomorphic organomineral material and a finely-dispersed material increased in the direction towards the hydrothermal field. The number of clay coatings within intrapedal pores increased with depth. Such coatings were probably formed due to alkalization and release of Na during weathering of primary minerals (with exchangeable Na content of 12.9 (+)µmol/kg in the Btn-horizon and only 0.3–0.8 (+) µmol/kg in upper horizons, Table S5). Such a process is typical for soils of the margins of thermal fields in the ultra humid environments of Kamchatka^18^.

The particle-size distribution was characterized by the predominance of PM_>10_. Compared to the non-heated Andosols, the slightly heated Andosols of zone II had a higher content of fine fractions (PM_<10_) and a lower content of PM_250-500_ (Tables S8, S9). The content of PM_<5_ increased with depth, with insignificant differences in the contents of coarser fractions. Properties (pH, SOC and EC) of Andosols from both zones showed similar absolute values and similar vertical distribution.

**Figure 4:**
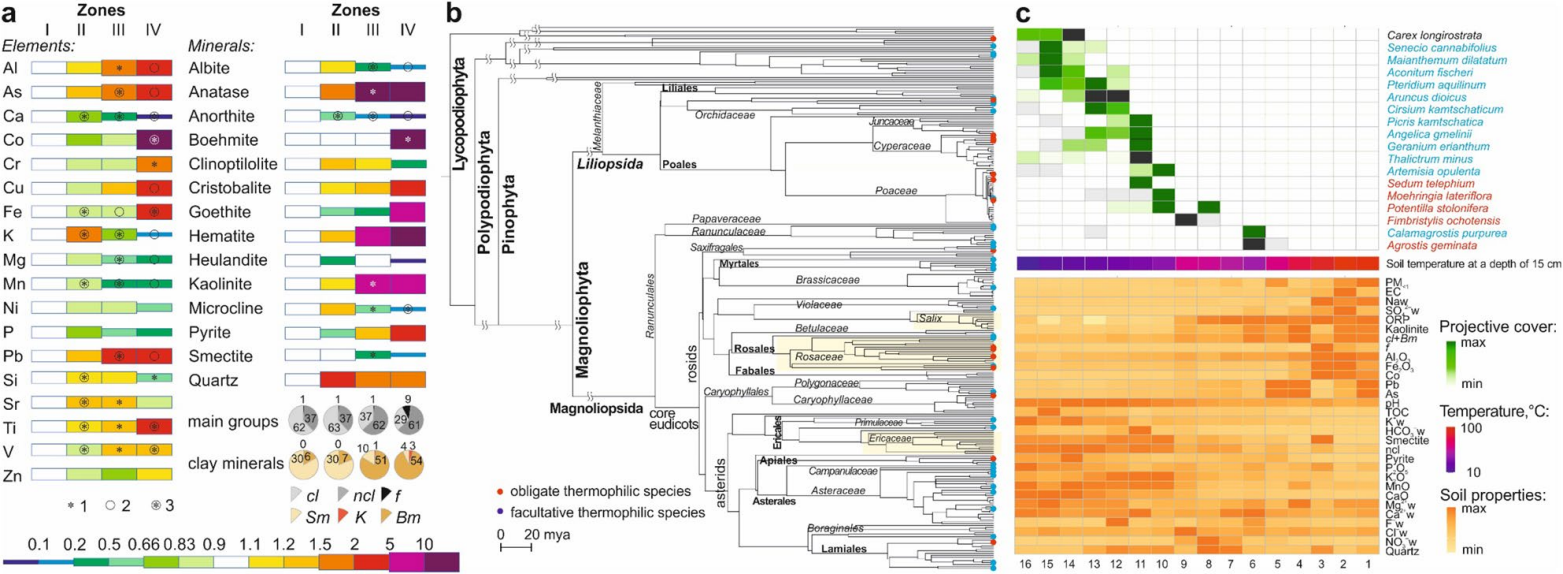
Partition of the topsoil (0–10 cm) and plants in different thermal zones of catena studied: (**a**) Element and mineral composition. Significant (*p* < 0.05) differences between composition of soils: 1—adjacent zones (I and II, II and III or III and IV), 2—zone I and other zones (II, III and IV), 3—in the zone under consideration and composition of soils both in zone I and in a cooler adjacent zone. The concentration of chemical elements and minerals are normalized to the composition in soils of zone I: depletion—blue and green, accumulation—yellow and red. Main groups of minerals (100%): *cl—*clay minerals (kaolinite, smectite, boehmite), *ncl*—non-clay minerals (microcline, albite, anorthite, clinoptilolite, heulandite, anatase, quartz, cristobalite), *f—*minerals of iron (pyrite, hematite, goethite, jarosite, marcasite). Clay minerals (100%): *Sm*—smectite, *K*—kaolinite, *Bm*—boehmite. (**b**) The thermophilic species observed along the catena on the phylogenetic tree of all the vascular plants occurred in the Valley of Geysers. (**c**) The key factors limiting plant species (black, non-thermal species; blue, facultative thermophytes; red, obligate thermophytes) growth within 1 × 1 m plots at the catena studied. 1–16, numbers of plots.

Slightly heated Andosols had higher contents of Si, K and Sr, lower contents of Ti, V, Fe, Co and Ca and similar contents of Cr, Ni, Cu, Zn, As, Pb, Al, P and Mg as compared to those of Zone I. Most elements showed uniform vertical distribution patterns, with the exception of P, which showed increased values in the topsoil (Fig. 4a).

The topsoil of zone II differed from that of zone I in the predominance of smectite, noticeable amounts (> 10%) of feldspars and quartz (Table 2), lower amounts (< 5%) of kaolinite, pyrite and cristobalite and the absence of considerable vertical differentiation (Table S8, Fig. 3b). Based on SEM data, newly formed hydrothermal pyrite and cristobalite also occurred (up to 5%) and occasionally large intrapedal pores filled with acicular mordenite (> 10%) (Fig. 2j).

**Table 2:** Mineral associations in soils and parent materials within zones of discharge of steam-heated acid-sulfate waters.

Territory		Parent materials	Temperature, °C	Sampling depth, cm	Minerals		References
					Rock-forming	Accessory and markers	
Russia, Kamchatka Peninsula	Valley of Geysers	Rhyolite-dacites or andesites	30–50	0–40 and 220–280	Smectite, feldspars, quartz	Plagioclases, anatase, zeolites, kaolinite, cristobalite	This study
			50–80	0–25	Kaolinite, anatase, zeolites	Plagioclases, quartz, feldspars, smectite	
			80–100	0–8	Kaolinite	Anatase, zeolites, plagioclases, hematite, quartz, cristobalite, feldspars, pyrite, smectite, **boehmite**	
			100	0–10	Kaolinite, quartz	Halloysite, non-ordered smectite (montmorillonite), clinoptilolite	^129^
	East Pauzhetka thermal field	Andesitic lavas	20–30	0–70	Kaolinite	Pyrite, α-quartz, **alunite, jarosite, goethite**	^58^
			35–70	70–250	Kaolinite	Pyrite, α-quartz, opal, feldspars, marcasite, **alunite, jarosite, goethite**	
			70–80	250–290	Kaolinite, smectite	–	
			80–95	300–350	Smectite	α-Quartz, opal, feldspars (albite), pyrite and other sulfides, **magnesio-calcite**	
			95–100	350–400	Smectite, opal, quartz, magnesio-calcite	Pyrite	
	Upper Pauzhetka thermal field	Andesites	< 98	0–30	Kaolinite, limonite	Dioctahedral smectite, pyrite, quartz, heulandite, plagioclases, **elemental sulfur, jarosite**	^134^
				5–50	‘Blue clays’: pyrite, silica (opal, chalcedony, quartz)	Jarosite, (hydro)goethite, hematite	
				30 (50)–180 (200)	Montmorillonite	Pyrite, hematite, (hydro)goethite, feldspars, silica (opal, chalcedony, quartz), illite–smectite, chlorite-smectite, magnetite, titanomagnetite	
	South Kambalnoe Dalnee thermal area	Andesites	80–95	25–162	Smectite**	Kaolinite, anatase, gypsum, opal, hematite, pyrite, gypsum	^145^
		Tuffites		150–310	Kaolinite	Smectite, quartz, cristobalite, and opal	^5,25^
			100	Topsoil	Opal, quartz, kaolinite	Alunite, sulfur, goethite	^131^
	Na	Andesites and basalts	40	0–200	Opal	**Alunite, sulfur**, gypsum, pyrite, **alum** (Al hydrated sulfate), (hydro)goethite	^44^
			20–100	0–400	Kaolinite	**Alunite**, gypsum, opal, mordenite, pyrite, (hydro)goethite	
Russia, Kuril Islands	North Paramushir hydrothermal magmatic system	Calcareous tuffite and tuffs	100*	Na	Opal, cristobalite	Jarosite, hematite, Fe hydroxides	^129^
					Silica, smectite, chlorite (up to 25%)	**Jarosite**, gypsum	
					Quartz, alunite	–	
Saint Lucia, Soufriere Volcanic Centre	Sulfur Springs	Dacites	41 – 97	Surface	Kaolinite, quartz	Feldspars, cristobalite, (natro)alunite, **jarosite**	^58^
USA, California	Little Hot Springs Valley (Lassen Volcanic National Park)	Travertines	Na	Surface	*Kaolinite, montmorillonite, alunite, natroalunite, opal, cristobalite	*Anatase, goethite, jarosite, hematite, Fe-sulfate minerals, native sulfur	^134^
		Landslides and colluvial sediments derived primarily from andesitic lavas	20 – 100	0–100	Kaolinite, smectite	**Alunite**	
Mexico	Los Azufres geothermal field	Pleistocene rhyolite	Na	Near-surface	Cristobalite, tridymite, opal, quartz, kaolinite	–	^145^
Iceland	Thermal fields Theistareykir and Namafjall	Basaltic hyaloclastites	> 20	Surface	*Pyrite, smectite, kaolinite	*Anatase, zeolites, alugene	^5,25^

*PM_<2_. **Following reconstruction, with samples taken from cooled areas (the hydrothermal system with regressive trend in the evolution). Markers are in bold.

#### Zone III. Moderately heated Eutrosilic Gleyic Aluandic Andosols (Loamic, Reductic, Protosalic, Hyperthionic) under different moss and microzonal communities

Moderately heated Andosols with temperatures of 40–80 °C at a depth of 15 cm (Figure S2) were characterized by the formation of a surface crust (brown or cream colored, humified, with a fine subangular blocky—crumbly structure; Table S2) or, at the margin of this zone, an A-horizon with a structure from fine angular blocky to coarse subangular blocky and inclusions of ferruginized plant remains (Fig. 2h). Fragments of volcanic rocks were also considerably ferruginized, which indicated an intensive inflow of fluids saturated with mobile Fe. The topsoil (0–4 cm) of zone III as compared to that of zone I had similar porosity, lower content of organic debris and collomorphic organomineral microaggregates and higher degrees of ferrugination of groundmass which isometric fragments consist of cracked sand-sized grains of plagioclases, weathered volcanic ash and heavy minerals. The SEM analysis showed that the topsoil had inherited the matrix microstructure similar to that in soils of zone II with apparent pseudomorphs over primary structural elements and a total replacement of initial rocks as a result of hydrothermal alteration as previously reported for thermally altered tuffites in Kamchatka^28^. The non-oriented clay matrix contained silty peds (Fig. 2k). In the subsoil, volcanogenic source rocks were strongly argillized.

Below the root zone, a relatively homogenous brown Bw-horizon of an altered volcanogenic material with a subangular blocky—angular blocky structure containing less than 20% of PM_>10_ was most often present. The Bw-horizon was underlain by a clayey B1-horizon with an angular blocky—prismatic or massive structure and various colors (alternating white, violet, ochric, red, brown and lilac stripes and mottles). On a micro scale, this material showed mostly dark gray hues in plain polarized light and a red, fluidal texture in reflected light (Fig. 2f), indicating the presence of intrusive ferruginous pedofeatures and a high content of anatase and alunite-jarosite. Similar brightly colored B1-horizons have been detected in endothermal soils of Iceland^5^, Kamchatka^18,24^ and California^37^. Therefore, it could be suggested that such horizons, which form under the influence of steam-heated acid sulfate water, occurred worldwide.

The Bl-horizon was underlain by a bleached white (whitish gray background matrix with cream and light blue mottles) clayey parent material (Fig. 1h). Some angular blocky and prismatic peds had red films on their surface and sides.

Compared to the non-heated Andosols, the moderately heated Andosols had higher amounts of fine particle-size fractions and lower content of PM_250–500_. The topsoil of zone III was dominated by PM_>10_ and the subsoil (> 10 cm) by PM_<5_ (Tables S8, S9). In the subsoil, PM_250–1000_ were absent, the content of PM_10–50_ was lower and the content of PM_<5_ higher than in the topsoil, while the content of PM_5–10_ and PM_50–250_ was similar.

In comparison to non-heated Andosols, moderately heated Andosols had higher Eh and EC and lower pH and SOC values due to the biogenic oxidation of sulfides with the production of sulfuric acid, which was confirmed by the presence of colonies of iron-oxidizing bacteria (Fig. 2f). SOC, pH, EC and Eh changed insignificantly with depth.

The elemental composition of moderately heated Andosols differed from that of non-heated Andosols by a higher content of Fe, As, Pb and Al and a lower content of Mn, Ca, K, Mg and Sr, with similar content levels of V, Cr, Co, Ni, Cu, Zn, Si and P (Tables S8, S10). The topsoil of zone III had higher contents of Ca, K, Mg, Mn and Si and the subsoil was enriched in Al, As, Cu, Fe, Ni, Pb, Ti, V and Zn. The content of P, Sr, V, Cr, Co and Ca varied insignificantly with depth.

Compared to non-heated Andosols, moderately heated Andosols had a higher content of kaolinite (the predominant phase in matrix and peds) and anatase, a lower content of smectite, potassium feldspars and plagioclases and a similar content of pyrite, zeolites, quartz, cristobalite, hematite and goethite (Table S8, Fig. 4b) which coincides with data^38^ on the disappearance of smectite in hydrothermal altered rocks with a temperature exceeding 85–95 °C at the Wairakei geothermal field, New Zealand. The topsoil was enriched in potassium feldspars and albite and impoverished in zeolite, anatase, quartz and cristobalite (SEM data showed that the last three minerals occurred within large intrapedal pores inherited from soils), with similar amounts of kaolinite, smectite and pyrite.

Judging from the XRD full patterns of kaolinite, it had a disordered structure (Fig. 3d–f). It should be mentioned that the calculation of different indices (Hinckley, AGFI) using several peaks of the XRD pattern was a very popular method for assessing the degree of kaolinite order/disorder. However, recent studies^39^ showed that only the analysis of the XRD full pattern was suitable for this. All the studied kaolinites were characterized as low-ordered, since they had poorly defined non-basal (*h,k*) peaks and the modulation of peaks at large angles 2θ was almost absent. The low ordering of the studied kaolinite structure was apparently caused by the following reasons: (1) low stacking order of the layers due to certain geological conditions during kaolinite synthesis, (2) occurrence of a small amount (up to 5–10%) of mixed-layered kaolinite-smectite (K/S) minerals. This was also indicated by the presence of a specific displacement of basal (00*l*) peaks after oriented specimen saturation with ethylene glycol (Fig. 3e,f). When calculating the mineral composition, the phases of kaolinite and K/S did not differ.

**Figure 5:**
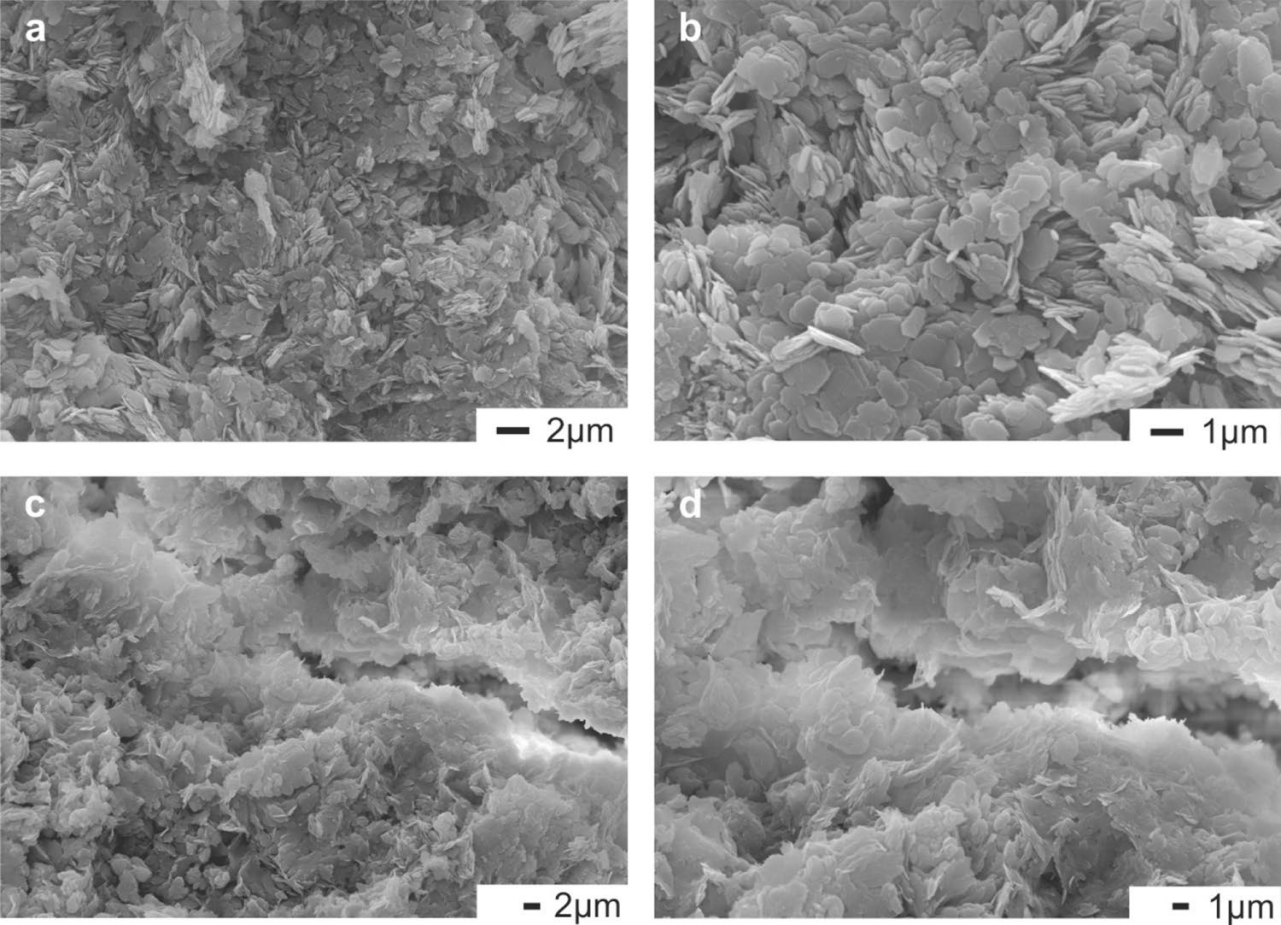
SEM images of clay mass of moderately heated Andosols in the Valley of Geysers: (**a**) Non-oriented clay matrix from moderately heated Andosols. (**b**) Pseudohexagonal crystals of kaolinite. (**c**) Open microcracks in clay matrix. (**d**) Microaggregates with sheet-like curved shape, composed of kaolinite and kaolinite-smectite particles, which form the walls of open microcracks.

According to the SEM study of the moderately heated Andosols, the kaolinite had a diverse particle morphology, which obviously showed the degree of its structure order/disorder. Pseudohexagonal crystals of kaolinite (Fig. 5b) were found within a non-oriented matrix (Fig. 5a). In contrast, crystals with poorly defined habitus or with irregular sheet-like curved shape (Fig. 5d) were found in microaggregates oriented along the walls of open microcracks (Fig. 3c). Thus, based on SEM and XRD, we suggested that these were particles of disordered kaolinites and kaolinite-smectite mixed-layer minerals. Such elongated aggregates were found in the dense groundmass of the layer (Fig. 2k).

#### Zone IV. Hot Gleyic Aluandic Andosols (Clayic, Reductic, Salic, Hyperthionic) near the center of the steam hydrotherm

Shallow and hot Andosols with temperatures of > 80 °C (Figure S2) exhibited a mottled (light gray and reddish) biocrust of thermophilic algae on the surface of a bright-colored clayey Bilz-horizon (Table S2), which was underlain by greenish, bluish and whitish clays (Crz-horizon) formed as a result of a deep hydrothermal alteration and bleaching of volcanic rocks. On a micro-scale, the zone IV topsoil consisted of laminated crusts of silty material impregnated and bonded by collomorphic Fe–Al-organomineral substances with a fluidal (after^40^) texture. Below, the argillized material was very heterogeneous in color (e.g., with whitish mottles corresponding to locally reducing conditions) and contained inclusions of various minerals (Figs. 1i, 2g,h). Such argillized material was typical for the shallow hydrothermal sediments of Kamchatka^28,41–43^ and the Iturup Island^27^. SEM analyses showed that hot Andosols consisted of kaolinite aggregates, pseudomorphs of which had replaced rock-forming minerals. The soil microfabric was typical for Kamchatka’s hydrothermal clays (Fig. 2l), i.e., inherited from the parent materials^25^, with a high porosity resulting from an intensive rise of vapors from endogenous hot fluids. Such pores were colonized by thermophilic bacteria.

The particle-size distribution of hot Andosols was characterized by the predominance of PM_<5_ and PM_10–50_ (Table S8). In comparison with non-heated Andosols, hot Andosols had a lower content of PM_>10_, lower pH and SOC values and higher values of Eh and EC due to an intensive inflow of endogenous fluids and the biogenic oxidation of sulfides (Table S7).

Hot Andosols as compared to non-heated Andosols were also enriched in Ti, Cr, Fe, Co, Cu, As and Pb, depleted in Mn, Ca, Si, K and Mg and had the similar contents of V, Ni, Zn, P and Sr (Table S7). Leaching of Ca, K, Na and Mg from volcanic rocks was known to be induced by steam-heated acid-sulfate waters^44^, while enrichment in Fe, Cu and Co could be caused by a selective biogenic accumulation of metals (including Ca, Mn and Sr^45^) within biocrusts. The impact of steam-heated acid-sulfate waters caused an enrichment of Ti within deep layers of subsurface sediments in Kamchatka^46^ and the formation of mineral coatings on stones in a hot river on the Iturup Island^27^.

The volcanogenic source rocks of the hot Andosols were deeply altered and mostly consisted of poorly-ordered kaolinite and kaolinite-smectite mixed layered minerals (Fig. 3e,f) and small amounts of boehmite, anatase and quartz (Table S8, Fig. 2l). Most samples of hot Andosols also had considerable contents of potassium feldspars, boehmite, pyrite, cristobalite and hematite. Plagioclases, goethite and zeolites occurred rarely. The smectite content was low, this being the main mineral of soils in non-heated and slightly heated Andosols. Boehmite, jarosite, pyrolusite, marcasite and calcite occurred only in hot Andosols (Table 2). In comparison with non-heated Andosols, hot Andosols were enriched in kaolinite, anatase, hematite and boehmite and impoverished in smectite, potassium feldspars, plagioclases and zeolites (Table S11). A high content of kaolinite and a low content of albite and quartz resulted from the duration of exposure to and/or high temperature of a steam hydrotherm^47,48^.

The microstructure of hot Andosols (Fig. 2l) differed from that of less heated Andosols by the presence of numerous round opal grains, which often formed collomorphic microaggregates, pore infillings and bridges between clay micropeds. At the surface of opal pedofeatures, there were occasional crystals of pyrite, anatase and cristobalite.

### Botanical transect description

Within the studied catena, from the periphery of the thermal field with communities (association of *Betuletum ermanii calamagrostidosum subassociations typicum* or *dryopteridosum*) on non-heated Andosols to its bare ground center, plant communities replaced each other in the following order: slightly modified tall herb meadow → modified meadow with dominance of *Thalictrum minus* or *Pteridium aquilinum* → communities with dominance of *Artemisia opulenta* and monodominant communities of *Calamagrostis langsdorfii* → microzonal communities^8^ with dominance of *Agrostis geminata, Lycopus uniflorus* or *Potentilla stolonifera* → microzonal communities with dominance of the *Marchantiophyta* (*Gymnocolea inflate* and *Solenostoma vulcanicola*)^49,50^ and *Fimbristylis ochotensis* → *Marchantiophyta* communities → biocrust.

The cover of vascular plants dropped at around 60 °C, with cover values not exceeding 20% above this temperature. Mosses decreased in cover at 65 °C and disappeared completely at around 90 °C. The total number of species varied from 9 to 24 per 1 m^2^ on the non-heated soils. With increase in temperature it decreased and did not reach more than 6 species per 1 m^2^ at a topsoil temperature of more than 43 °C. Thus, together with previous studies^51–54^, our research showed that in the Valley of the Geysers, non-vascular plants dominated at the moderately heated and hot soils.

The clear majority (263 of 292, see Methods) of species belonged to *Magnoliophyta*. Both facultative (Net Relatedness Index, NRI = 1.7 ± 0.5, *p* = 0.036) and obligate (NRI = 1.3 ± 0.8, *p* = 0.084) thermal species seemed to be clustered on the phylogenetic tree of *Magnoliophyta* from the Valley of Geysers (Table S12). However, they did not form a monophyletic clade and were organized in small clusters^55^ of closely related thermal species that were distributed throughout the phylogenetic tree (Fig. 4c).

Only 9 of 60 families of vascular plants contained obligate thermophyte species (Fig. 4c, Figure S3). *Rosaceae* (4 of 18 species) were overrepresented with a false discovery rate (FDR) level of 15%. The other two families represented by more than one species were *Cyperaceae* and *Poaceae* that had been typical for Cenozoic floras of geothermally influenced wetland hydrophytic and dryland mesophytic communities^56^. As far as orders were concerned, only *Rosales* were overrepresented in the obligate thermal flora (Table S13).

Among 24 families that contained facultative thermophytes, no families were significantly enriched or depleted in such species. At the level of orders, *Poales* were underrepresented and *Asparageles* were overrepresented among facultative thermophytes (Table S13).

Among 6 families containing only thermal species (both facultative and obligate thermophytes) in the Valley of the Geysers, 5 families (*Dennstaedtiaceae, Geraniaceae, Urticaceae, Rubiaceae*, and *Iridaceae*) were represented by only one species, and *Amaryllidaceae* were represented by two species *Allium ochotense* and *A. strictum*. Although this was not surprising having a probability of 0.03 to occur by chance, the fact that both these species were thermal may relate to the fact that the main center of *Allium* genera diversity located in southwest and central Asia^57^ i.e., *Allium* sp. might serve as an indicator of thermal habitats in Kamchatka.

At the non-heated habitats near the catena, among families of the Valley of Geysers with only non-thermal species, *Thelypteridaceae*, *Equisetaceae* and *Betulaceae* were overrepresented (Table S13). Overrepresented orders were *Equisetales*, *Polypodiales*, *Fagales* and *Liliales*, while the order *Poales* was underrepresented. At the class level, the nearest non-thermal flora at non-heated Andosols (group B, see Methods) was enriched with *Polypodiopsida* species, which could result from the higher humidity of this territory^18^.

### Alteration of Andosols under the impact of steam-heated acid-sulfate waters

In the Valley of Geysers, the inflow of hydrothermal fluids into Andosols seemed to cause a decrease in TOC content, salinization (Figure S4), alkalinization (only at the hydrotherm margin), accumulation of Al, As, Co, Cr, Cu, Fe, Pb, Ti and V, removal of Ca, K, Mg, Mn and Si resulted from acid hydrolysis and a deep mineralogical alteration of the parent materials. The biogenic oxidation of sulfides^15,22,23,43,58^ provided forming of potential acid sulfate soils. A decrease in the TOC content resulted from both a small amount of litter—the projective cover of vegetation decreases from the periphery to the center of the hydrotherm—and sulfuric acid decomposition of organic matter. These processes were typical for endothermal soils affected by the steam-heated acid-sulfate waters in Kamchatka^18^, New Zealand^9,59–62^, Iceland^22^, California^37^, Japan (Hokkaido^63^ and the Kusatsu-Shirane volcano region^64^).

The hydrothermal alteration in endothermal soils was characterized by the transformation of smectite (the predominant clay mineral) to kaolinite, the assemblage of non-clay minerals (albite, anatase, anorthite, clinoptilolite, cristobalite, heulandite, microcline, quartz), a decrease in the proportion of plagioclases (with a relative increase in microcline) and the appearance of noticeable amounts of jarosite and boehmite (Fig. 4c). New mineral phases and an increased proportion of clay in the particle-size distribution were previously reported in endothermal soils of Kamchatka^18,24^, Japan^64^, California^37^, and Iceland^5^.

The A-horizon of the non-heated Andosols had a weakly developed macrostructure and a good microstructure. Due to the more intense alteration of rocks and minerals and a heavier texture, in slightly heated Andosols, the structure was well-developed both at a macro and micro scale. In zones III and IV, the A-horizon degraded to a biocrust. A similar trend was observed in the B-horizon, i.e., the structure was most developed at the hydrotherm’s periphery because of the favorable combination of factors such as a loamic texture and the release of bases from weathered primary minerals.

In the topsoil (0–10 cm), the SiO_2_ content in zones I–III was typical for non-heated Andosols of Kamchatka^65^, but higher than those in zone IV, where Al, Fe and Ti were introduced by hydrothermal fluids and contributed to the formation of disordered kaolinite, anatase, hematite, goethite and boehmite.

Soils with temperatures below and above 50 °C differed in their mineralogical composition. Soils of zones III and IV had lower contents of plagioclases, mordenite, potassium feldspars and smectite and higher contents of anatase, boehmite, kaolinite and hematite (Table 2). The studied endothermal soils in relation to non-heated Andosols had higher contents of clay minerals and Fe- and S-containing minerals and lower contents of other non-clay minerals: albite, anorthite, clinoptilolite, microcline (Fig. 4c).

### Clustering of thermal species on the phylogenic tree

In the Valley of Geysers, the *Poaceae* (30 species) and *Cyperaceae* (19) were the most represented families after the *Asteraceae* (31). But only one *Poacea* species and no *Cyperacea* species were among 33 facultative thermophytes, which makes *Poales* the most underrepresented order among facultative thermophytes (Table S13). Given that this order was the most prevalent among obligatory thermophytes (Table S14), we suggest that there are different constraints of thermophiticity and thermotolerance^66^. And for *Poales*, specialization was the more preferred way of adaptation to the extreme environment (high acidity, salinity, temperature and clay texture) rather than generalization. According to^67^, *Poales* and *Asterales* were over‐represented by alien plants on Mediterranean islands due to their late flowering, large seed size and anemochory. The prevalence of the *Poales* among obligate thermophytes could resulted from their ecological competitiveness^68^. For example, the *Poaceae* and *Cyperaceae* are well adapted to drought^69^. Additionally, the *Poaceae* are well adapted to saline habitats^70^. Data on the fossil floras of ancient hot carbonated springs support the fact that vegetation pre-adapted to chemically and physically stressed environments colonizes heated habitats^56^ i.e., plants occupy free niches.

In the geobotany analysis described above, all the species that occurred in the Valley of Geysers (in areas with either temperature, but had not been found at the catena and at the surrounding territory) were merged in the group of distant territory (both non-heated and heated). It was justified by several reasons. Firstly, the temperature, humidity, salinity and altitude differed throughout the Valley of Geysers^1,18^. And our analysis aimed to compare vegetation of the specific thermal site affected by steam-heated acid-sulfate waters with its surrounding and distant territories represented by all other parts of the Valley of Geysers. Secondly, it appeared to be impossible to clarify which species had been identified as "thermophytes" without a detailed soil temperature survey in^71^ later provided in^1,8^. When we specified species with the status "thermal" in either distant territories (the Geysernaya River valley’s plant species list compiled by L.I. Rassokhina: see Table S14) or in the catena studied as "thermal" and species with "non-thermal" status in either study as "non-thermal", the results were overlapping, but not the same (Table S15). The *Cyperaceae* was the overrepresented family among obligate thermophytes, but underrepresented among facultative thermophytes. The *Ericaceae* and *Saxifragaceae* tended to be non-thermal (simultaneously overrepresented among non-thermal species and underrepresented among thermal species), while the *Caryophyllaceae* and *Juncaceae* were the thermal species (vice versa). 10 of the 12 *Ericaceae* and all 4 of the 4 *Saxifragaceae* species were non-thermal; 9 of the 10 *Caryophyllaceae* and 8 of the 9 *Juncaceae* species were thermal. We suggested that the differences between the results obtained by the two calculation methods were caused by the diversity of thermal habitats in terms of Eh, pH, cation–anion composition and salinity of waters, heating and moistening of soils^1,18,24,48^.

### Relationships between the composition of plant communities and soil properties

The depth of plant root occurrence decreased from non-heated Andosols to heated Andosols of zones II and III (Table S2). Plant roots were not found in hot Andosols. In non-heated Andosols, slightly and moderately heated Andosols, the diameter of plant roots decreased, too. We did not notice any features (e.g., horst- or cushion-forming) of underground plant parts or roots that were preserved alive where there was direct contact with heated ground.

In the catena studied, the most drastic changes in soil morphology at the meso-, micro and nano-scales, elemental composition and particle-size distribution occurred when the temperature at a depth of 50 cm rose above 60 °C, with the most significant mineralogical changes occurring with the temperature transition at 50 °C. The species diversity of the facultative thermophytes changed sharply when the soil temperature at a depth of 50 cm rose above 98 °C, although that of obligate thermophytes changed only slightly (Figure S5). The pairwise comparison of species compositions from the thermal zones showed that zones II–IV had more similar species than expected by chance and zone I differed significantly from zone II due to the loss of non-thermal species in zones II, III and IV and their high diversity in non-heated areas. At the catena studied and its surrounding territory (both non-thermal and thermal landscapes), the number of obligatory thermophytes gradually decreased with an increase in temperature, while the number of facultative thermophytes remained the same in zones II and III and sharply decreased in zone IV that could have resulted from the absence of any visible morphological adaptations, for example (Figure S5).

The absolute majority of the thermophilic vascular plants were mesophytes and not psychrophytes in relation to temperature and humidity, respectively (Table S16). In comparison to non-thermal flora, there were no overrepresented or underrepresented biomorphs (Fisher's exact test, *p* value > 0.05).

Based on the relationships between the cover values of plants and edaphic conditions at the catena we distinguished 11 ecological groups of species (Table S17–S19). The main edaphic factors limiting plant growth included EC, the content of sulfates in water extract, particle-size distribution and pH. Even for the thermal zones, such factors as the soil temperature, SOC content, concentration of toxic compounds and Eh were less important.

In thermal landscapes, biodiversity can increase or decrease depending on the maximal temperature at the certain hotspot. When a temperature of heated neutral solutions is low (less than 30 °C) e.g., in the Bolshezemelskaya tundra (Russian Subarctic)^72^, due to higher frequency of soil freezing–thawing, environmental harshness may result in decrease in the floristic and microbiota richness. Such a partition was observed at the Hengill valley in Iceland^73^. In the South Sandwich Islands (maritime Antarctic), bryoflora richness was maximal at the thermal ecosystems where the topsoil surface temperature reached 47 °C^74,75^. In the forest landscapes of Kamchatka^76^, Hokkaido^48,77^, California^37^ and New Zealand^7,9,61,62,78^, the inflow of heated thermal waters resulted in disappearance of woody plants. Vegetation was sparse at the endothermal soils with surface temperatures of more than 50 °C. In thermal ecosystems, the highest vascular plant^79^ and moss^80^ species richness was typical for areas where the topsoil temperature is 30–40 and 30–50 °C, respectively due to the upper thermal limit for cell activity lied between 45 and 55 °C.

The subsoil temperature seems to be the main factor controlling the vegetation of geothermal areas, while soil chemical factors are thought to have less influence^1,8,81–84^. Because soil temperature is the simplest and most reliable indicator to assess the plant partition at thermal ecosystems during the field study, other environmental factors (e.g., pH, soil moisture, a content of phytotoxic substances) were studied insufficiently. But soil temperature affects the vegetation together with other physical and chemical soil properties that also depend on the intensity of the inflow of heated fluids and waters. A relationship between the vegetation and the environment has only been analyzed based on the data collected at transects from a center of a hydrotherm to its periphery. However, such approach does not allow separating the influence of soil temperature and chemical properties on vegetation as these factors are dependent variables. To understand the role of soil temperature and chemical properties on plant growth separately, more detailed sampling should be carried out into one thermal zone.

## Materials and methods

### Study area

The narrow and deeply incised valley of the Geysernaya River has been hiding from human eyes remarkable and unique natural objects until its discovery in April 1941 by an employee of the Kronotsky Reserve, geologist Tatiana Ustinova (1913–2009). Since its discovery and due its location within the Kronotsky Federal Nature Biosphere Reserve, securing the highest level of protection of unique ecosystems and creating opportunities to use them as etalons for studying undisturbed course of events, the ecosystem of the Valley of Geysers became one of the most popular objects for scientific investigations.

The Valley of Geysers and the Uzon Caldera are located 180 km northeast of Petropavlovsk-Kamchatsky City and surrounded by volcanoes, arranged in a chain along the eastern coast of the Kamchatka Peninsula within the so-called Eastern volcanic belt, being part of the Pacific Ring of Fire^85^. The Valley of Geysers is the second largest geyser field in the world (Fig. 1a) after the Yellowstone National Park^32,48,86^. More than 100 described geysers and about the same number of dwarf and unnamed geysers are located on an area of 50 thousand m^2^^77^. According to^87^, their total number exceeds 200.

#### Geology

Geologically, the hydrothermal system of the Valley of Geysers is situated within the margin of the Uzon-Geyser Caldera, where acidic crustal volcanism has developed over the last 40 ka^1^. The studied territory on the left (east) side of the Geysernaya River with various thermal springs including geysers is a part of the depression named Geysernaya caldera^88,89^—the oldest part of the caldera system, the age of which is estimated at ~ 280 ka^90,91^. In the Geysernaya River valley, andesitic tuffs and lacustrine sediments are the main parent materials^92,93^, i.e., Geysernaya and Pemzovaya units (Q_3_^4^gr,pmz)^89,94^. An intensive drainage of heated water began 9–12 ka^1,24^, when the formation of the studied ecosystems had probably started.

#### Climate

According to the Köppen−Geiger classification, the climate of Kamchatka is characterized as Dfc^18,41^ with a mean annual precipitation of 2000–4000 mm^1^. In the studied part of the Geysernaya River valley (Fig. 1b–d), there is a persistent characteristic smell of H_2_S due to its permanent release from the ground (Table S20). The soil temperature regimes range from frigid in the non-thermal areas to hypothermic within the center of the parahydrotherm.

#### Soils

The Geysernaya River valley is located at the boundary between the Central Soil Province with pyroclastics of a rhyolite-dacite composition (where the youngest andesitic ashes are from the Opal Volcano erupted in 606 BC) and the Northern Soil Province with andesite ashes^24^, where Vitric Andosols (Arenic)^95^ with contents of oxalate-soluble Fe and Al of 0.8–1.8%^44^ are formed. The water enters the hydrothermal system from meteoric sources and moves rapidly through the permeable rock and fractures to where it is heated by the magma. The water composition of the geysers and hot springs (Table S1) varies from sodium-chloride to magnesium-calcium-sulfate(-hydrocarbonate) types^96^.

The first systematic soil survey of the Geysernaya River valley was conducted by Danila Kostuk and headed by Anna Zavadskaya in 2010 and provided information for the soil mapping of the territory (Fig. 1b)^97^. Descriptions of some of the soil samples and/or pits from the Geysernaya River valley were represented in^98^.

#### Thermally induced variability in soils and vegetation

For the non-heated Andosols, the vegetation of the Geysernaya River valley is represented by tall-grass, forest and prostrate shrub (i.e., stlanik) communities including *Betula ermanii, Salix udensis* and *Alnus kamtschaticae*^9,53,59,61,99,100^. Increasing influences of endogenous fluids and temperatures within a distance of up to 50 m are accompanied by a transition from non-heated Andosols (Arenic) with several buried soils under tall herb communities to hot Gleyic Andosols (Clayic) (Table 1) that can support only thermoacidophile species^9,53,82,84,101,102^. Slightly heated surfaces are colonized by Kamchatka’s tall herb communities (e.g., *Filipenduleta camtschaticae, Calamagrostideta langsdorffi* and *Saussurieto pseudo-tilesii-Geranieta erianthis*), while moderately heated Andosols are occupied predominantly by thermophyte mosses^4,9,60,61,78,101,103,104^. Biocrusts, which are formed within Kamchatka’s hot spring and hydrothermal field areas due to the activity of *Cyanidium caldarium*^53,105^, also include *Streptomyces* and other actinomycetes^54^. Aerobic autotrophic sulfur-oxidizing bacteria of the genus *Sulfurihydrogenibium* (phylum *Aquificae*) are dominant in most streams of the Valley of Geysers. Another widely distributed group is anaerobic bacteria of the genus *Caldimicrobium* (phylum *Thermodesulfobacteria*). Archaea of the genus *Vulcanisaeta* are abundant in slightly acidic hot springs, where they are accompanied by numerous *Nanoarchaeota*^63^.

#### Geothermal vegetation

The vegetation cover of geothermal areas is represented by so called geothermal vegetation, which has characteristics determined by the current and former input of geothermally-derived energy (heat) or materials e.g., solid, fluid or gas^99^. Previous studies have outlined the fundamental role of soil-chemical composition and temperature in influencing the establishment and growth, spatial structure and composition of geothermal vegetation^102,106,107^.

In geothermal areas, plant communities occupy habitats with very specific soil temperatures and replace each other in a particular order. Spatial distribution patterns depending on the soil temperature have been observed in vegetation communities surrounding geothermal features elsewhere in the world: in New Zealand^8,82^, Iceland^56^, Canada^53^, Hokkaido^49^, Italy^63^, the Yellowstone National Park^7,101,102,107^ and the Kamchatka peninsula^108^. Thus, the spatial distribution of communities reflects the configuration of the thermal fields. On the periphery of thermal fields, on the slightly heated soils, plant communities cover considerably large areas. Closer to the hottest area of the thermal field with moderately heated and hot soils, vegetation forms small mosaics of specific geothermal communities. As the size of such geothermal plant communities in most cases is extremely small (less than 1 m^2^), they are named “microzonal thermophilic complexes”^20,21^*.*

Geothermal vegetation is usually floristically very simple^8,50,81,82,109–111^ and dominated by bryophytes in Iceland^4,53,61,62,101^, NE Kamchatka peninsula^1,112^ and Japa^51–54,63,84,101^, by grasses and herbs in the Yellowstone National Park^53,78,99,106^, the SE East-European plain^53^, the Baikal rift zone^60,81,83,109,113^ and Kamchatka peninsula^109^ or by woody vegetation in New Zealand^109^. Such geothermal communities often contain endemic and threatened species^60,113^.

Patterns in vegetation structure, appearing with temperature and soil-chemical composition gradients of thermal fields have been observed by numerous authors^71,81,113^. Overall, the total vegetation cover of communities tends to decrease as the temperature increases, but with plant groups responding differently. Vascular plant cover tends to drop in different geothermal areas at the temperature range of around 45–60 °C (here and further measurements of substrate temperature at depth of 10–15 cm used for describing soil temperature gradient). Their total cover reduces rapidly above this temperature. Mosses tend to have a higher limit of tolerance to the surface temperature. They decrease in cover at 55–65 °C and disappear in different geothermal sites at around 75–95 °C^60,114^. The total number of vascular plants within a geothermal ecosystem is inversely proportional to the surface temperature: being greatest at non-heated or slightly heated soils surrounding geothermal areas, it decreases as the temperature rises, and reaches less then several species per plot with moderately heated or hot soils. So, non-vascular plants dominate geothermal highly heated areas, as the tolerance of normal plant cell activity of vascular plants ranges from between 45 and 55 °C^82,115,116^.

Studies of species distribution in geothermal areas have allowed to suggest classifications of plant species according to the soil temperature of the habitat^1,24^. Generalizing these classifications, the following groups of plants of geothermal areas can be distinguished:

1. Plants occurring only in geothermal habitats with extreme environmental conditions, or “obligate (extreme) thermophiles”^117^;
2. Plants which are able to tolerate conditions within geothermal habitats, but may also occur in neighboring non-geothermal vegetation, or “facultative thermophiles”^118^;
3. Disjuncts—plants outside their normal latitudinal and altitudinal range with warmer climate, which establish in geothermal sites as they mimic aspects of its usual habitats^119^;
4. Plants on the periphery of the geothermal feature;
5. Ruderal plants.

In relation to the latter group of plants, many authors noticed the very high susceptibility of slightly warm and warm geothermal habitats for weed invasion and the necessity of preserving the native plant communities and species of such thermal sites^120,121^. Some studies showed strong negative correlations between species abundance and surface temperature, and proposed using these plants as indicators of change in the underlying geothermal conditions^120,121^ and for mapping soil temperature^122–124^.

Although regional case studies mentioned above found many general patterns in vegetation structure and composition change along environmental gradients in geothermal areas, all the authors emphasized that their fundamental characteristics are specific for each geothermal field or region and need to be studied in detail for each region. And while relationships between vegetation changes and soil surface temperature have been well described and understood in the majority of geothermal regions, less attention have been paid to studying the influence of other environmental factors on the vegetation of distinct ecosystems of thermal fields. Our study aims to help fill this gap for the Valley of the Geysers in Kamchatka.

### Field work

On the left (east) bank of the Geysernaya River, on a catena slope with a height difference of 2 m and length of 30 m from the periphery to the center of the thermal site, we studied the vegetation within 1 × 1 m plots (Fig. 1, Figure S6), and 18 soil pits (Table S1, Figure S1), from which a total of 65 samples were collected in the NW of geyser Bolshoi and in the SE of geysers Shchel, Grotik, Vrata Ada, Kotly, Vanna, mad pot Krasny and lakes Utinoye and Teploye (Table S21). We selected a catena that was a good representation of the typical ranges for soils and plant communities observed around a hot spot^125^ and met all the following criteria: round-like shape; size sufficient for digging soil pits and characterizing 1 × 1 m plots at least in triplicates in all the thermal zones (I–IV); minimized influence of other hydrothermal fields and hot springs, tourist activity and consequences of landslides.

Color was described in field-moist soils, using^126^. Soil temperatures were measured at depths of 15 and 50 cm using a TekhnoAs TK-5 contact thermometer (Russia). Soil descriptions were performed in accordance with^39^. Soils and horizons were named using^123^.

### The vertical gradient of temperatures within a layer of 0–50 cm

In the non-heated area (pits 14, 15 and 16 in Zone I, Fig. 1d), the temperature of the non-heated Eutrosilic Silandic Andosols (Arenic) did not exceed 30 °C even at a depth of 2 m (Figure S2). Equation (1) Describes the change in soil temperature with angle α = arctg(k) values of 4.6–5.2°.

1 $${\text{T}}_{0} = {\text{kd}} + {\text{T}}_{{{15}}} ,$$

where T_0_—temperature of the analyzed soil layer, d—depth between 0–50 cm, T_15_—temperature at a depth of 15 cm.

As compared to non-heated Andosols, the slight heating of Andosols (Loamic) in Zone II (Table 1) was confirmed by significantly (*p* = 0.034) increased α values (10–19°) and temperatures of 34–54 °C at the depth of 50 cm. Moderately heated Andosols (Loamic) in Zone III were characterized by even more significantly (*p* = 0.011) increased α values (23–37°). Specifically, zone III soils were characterized by relatively cool (60–80 °C) upper layers and hot (> 80 °C) lower layers. In the upper 50-cm-thick layer of hot Andosols (Clayic) in zone IV, temperature was evenly distributed or, at the zone’s periphery, increased with depth from 90 to 100 °C.

### Soil analyses

For the chemical analyses, bulk soil samples were collected from all soil horizons, air dried and sieved through a sieve with a mesh size of 1 mm as recommended in^40^. Particle-size distribution, element composition, redox potential (Eh) and SOC content were determined (Table S22). Water extract analyses including pH in suspension, electrical conductivity, basicity from HCO_3_^−^ and the cation–anion composition were also performed in filtrates (Table S22). The cation–anion composition of water extracts was determined by high performance ion chromatography (HP-IC). The particle-size distribution was characterized using the Russian system of particle sizes (µm) developed by N.A. Kachinskii^127^: 1000–500 (PM_500–1000_, coarse sand), 500–250 (PM_250–500_, medium sand), 250–50 (PM_50–250_, fine sand), 50–10 (PM_10–50_, coarse silt), 10–5 (PM_5–10_, medium silt), 5–1 (PM_1–5_, fine silt) and less than 1 (PM_1_, clay).

The mineralogical composition (phyllosilicates and other minerals) of bulk soil samples and clay fractions (< 1 µm) was determined using ‘Ultima-IV’ and ‘Rigaku’ X-ray diffractometers (XRD). Minerals were identified by comparing the data obtained with standard XRD patterns from the PDF-2 database with the use of the MDI Jade-6.5 software (https://materialsdata.com/prodjd.html) and methodological recommendations^119^. Quantitative mineralogical analysis was carried out using the Rietveld full-pattern fitting method^29,128–133^ and the PROFEX GUI for the BGMN software^5,58,134,135^ as modern tools (used in soil science since 2009) for the most precise quantitative assessements of the mineralogical composition of a sample. Investigation of di-tri species of clay minerals and degree of deffects (ordering/disordering) of kaolinite minerals were carried out on non-oriented powder samples (Table S23)^136^. Investigation of expandable minerals was made according to routine procedure^137^. Composition of mixed-layered minerals and ratios of kaolinite and kaolinite/smectite were determined for soils in zones III and IV.

Based on the macromorphological analyses (Table S2) and the primary processing of data on the chemical and mineralogical composition of soils, we selected representative soil pits (1, 7, 11 and 16, Figure S2, Fig. 1) for more detailed micromorphological studies and extractions of exchangeable and oxalate-soluble compounds.

Micromophological analysis of thin sections were performed using standard techniques and an Olympus BX51 polarizing microscope (Japan) with an Olympus DP26 digital camera and specialized computer software. Soil microfabrics of main genetic horizons were compared by analyzing large (4 × 5 cm) thin sections with replications. The international terminology for micromorphological descriptions was used^138^.

Based on the results of above analyses, five samples were selected for detailed investigation using a LEO 1450VP scanning electron microscope (SEM) equipped with an INCA 300 microanalyzer (Carl Zeiss, Germany). Field-moist samples were dried using the vacuum freeze-drying technique and their micro/nano structure and composition then studied.

Short-range-order Al_ox_, Fe_ox_, Si_ox_ compounds (Figure S7) were extracted after shaking the soil with a 0.2 M ammonium oxalate solution buffered at pH 3.0^127^. Exchangeable compounds were extracted using an ammonium acetate buffer at pH 7.0^139^. The element composition of both extracts was determined using an ‘iCAP-6500’—inductively coupled plasma atomic emission spectrometer by ‘Thermo Scientific’ (USA).

### Data processing

#### Characteristics of soil properties

The mineralogical composition was assessed using the parameters of clinoptilolite and heulandite as varieties of zeolite, albite and anorthite as varieties of plagioclases and microcline as a representative of potassium feldspars. The above mentioned minerals (clinoptilolite, heulandite, albite, anorthite, microcline) are the most likely to occur in volcanic materials affected by hot fluids (Table 2), according to the results of previous studies in Kamchatka^109^ and other regions of the world^140^.

To reveal changes in soil properties, data were grouped based on the following criteria:

1. Locations of soil pits within thermal zones I–IV (Fig. 1, Table 1, Table S2);
2. Temperatures (°C) of specific samples, i.e., < 30 (non-heated), 30–50 (slightly heated), 50–80 (moderately heated) and > 80 (hot);
3. Soil sampling depth.

Descriptive statistics were computed using R software (version 4.0.3, h ttps://www.R-project.org/)^141^. To analyze differences between mean values, the one-sided p-level Mann–Whitney U test was applied, with Bonferroni correction for multiple tests, regarding values with *p* < 0.05 as significant.

Degrees of alteration of parent materials were assessed with the use of weathering indices—silica–titanium index (STI)^141^ and Silica/R_2_O_3_^142^:

2 $$STI = \frac{{SiO_{2} }}{{\frac{{SiO_{2} }}{{TiO_{2} }} + \frac{{Al_{2} O_{3} }}{{TiO_{2} }} + \frac{{SiO_{2} }}{{Al_{2} O_{3} }}}} \times 100$$

3 $$Silica/R_{2} O_{3} = \frac{{SiO_{2} }}{{Al_{2} O_{3} + Fe_{2} O_{3} + TiO_{2} }}$$

#### Characteristics of plant communities composition

##### Comparison of species composition between thermal zones

Data on the species composition of plant communities were represented by three groups as follows: data on the studied catena (group T), its surrounding territory (group B; Figure S6) and all flora of the Geysernaya River valley (group Ya + R) (Table S14). Group T included 29 species of vascular plants recorded during the present study at 18 points corresponding to soil pits within the catena (Table S24). Group B included 66 vascular plant species (58 *Magnoliophyta*, 7 *Polypodiophyta* and 1 *Lycopodiophyta* species) recorded by M.V. Prozorova and A.V. Zavadskaya^55^ during botanical surveys of 178 plots on the left bank of the Geysernaya River—in thermal fields (including the studied catena) as well as non-heated territories. Group Ya + R included 292 species of vascular plants with their ecology and habitats (e.g., hydro-, hygro-, meso-, xero-, petro- and psychrophytes) recorded throughout the flora of the Valley of Geysers as a result of long-term studies by L.I. Rassokhina^1^ supplemented by her own unpublished data.

Thermophyte species of the Valley of Geysers were subdivided into obligate (occurring only in heated habitats^119^) and facultative (observed both in heated and non-heated habitats). The datasets analyzed were as follows: 1. groups T and B, 2. all groups (T, TB, Ya + R) and 3. distant territory.

##### Under- and overrepresentation of taxa in groups of plants

To identify which plant taxa (families, orders and classes) were significantly overrepresented/underrepresented in a group, for each taxon (for each family, order and class, respectively) we calculated the probability of selecting the same or more/less species from this taxon from the entire species pool of the Valley of Geysers. Assuming that there are *N* species in a species pool, of which *K* are from the taxon under consideration, and there are *n* species in a group, of which *k* are from this taxon. The probability *p*_*(k)*_ that *k* species from this taxon have occurred in the group by chance is calculated with the formula of hypergeometric probability:

4 $$p\left( k \right) = \frac{{C_{K}^{k} \times C_{N - K}^{n - k} }}{{C_{N}^{n} }}$$

Then the probability P*over* for *k* or more species from this family to occur in this group by chance is a sum of *p(i)*, *i* $\in$ [*k*, min(*K*, *n*)], and the probability P*under* for *k* or less species from this family to occur in this group by chance is a sum of *p(i)*, *i* $\in$ [0,*k*].

If P*over* (P*under*) is low, it is unlikely that *k* or more (less) species from this taxon can appear among *K* species in the group by chance. We assumed that taxa with P*over* ≤ 0.05 for a particular group were overrepresented in it, and taxa with P*under* ≤ 0.05 for a particular group were underrepresented in it.

Calculations were made using custom Perl scripts.

##### Phylogenetic clustering of thermal species

As a measure of clustering of thermal species on a phylogenetic tree of the Valley of Geysers vascular plants, we calculated the net relatedness index—NRI^1^:

5 $${\text{NRI}} = - 1 \times \frac{{{\text{MPDobs}} - {\text{min}}\left( {{\text{MPDexp}}} \right)}}{{{\text{SD}}\left( {{\text{MPDexp}}} \right)}}$$

To calculate NRI, it was necessary to first compute mean observed pairwise phylogenetic distances (MPDobs) between species from the set. Due to computational difficulties^128^—if species formed more than 100 pairs—we randomly took 100 of them to calculate MPDobs, and took all pairs otherwise. To make a null distribution for MPD (MPDnull), we randomly subsampled the same number of pairs from the entire species pool represented in the phylogenetic tree for 10,000 times. In addition to NRI, we estimated a significance of phylogenetic clustering by ranking MPDobs in the distribution of null values. To account for stochasticity of subsampling, we repeated the whole procedure 10 times for each phylogenetic tree and calculated median NRI and *p* value over these 10 replicates.

NRI is a standardized mean phylogenetic distance (MPD) between all pairs of species from the same group, multiplied by − 1. NRI is positive when species from a group are clustered on a phylogenetic tree (i.e., species from the group tend to be closely related), and negative when species are evenly spread across a phylogenetic tree. The significance of this relatedness can be obtained by ranking the observed MPD in comparison with the distribution of null values.

We built phylogenetic trees of 292 vascular plants species found in the Valley of Geysers based on a phylogenetic tree from^24^. From our species pool, 224 of 292 species were already present in the tree. Then we added 68 remaining species from our species pool to the tree by adding their branches using 3 different methods with R scripts provided in^119^: 1. as basal polytomies within their families, 2. randomly within their families, 3. based on Phylomatic^130^. Furthermore, we calculated NRIs for each group of species using each tree with custom Perl scripts and averaged the results from 3 trees. Separately, we performed the same procedures on 263 Magnoliophyta plants that have been found in the Valley of the Geysers.

#### Calculations were made using custom Perl scripts

##### Comparison of species composition between thermal zones

For each pair of thermal zones I–IV, we estimated the significance of having more similar species content than it would be for the random sampling of the same number of species from the list of all species that were found on 178 experimental plots (groups T and B)^29^. For each pair of zones (namely, zone I with *N* species and zone II with *M* species), the null distribution of number of common species between the two zones was constructed by subsampling of *N* and *M* species from the species pool for 1000 times. Then a right-sided and left-sided *p* value was obtained by calculating the fraction of subsamplings with the same or more/less common species, respectively than it was in the real data (Tables S13, S15). A low right-sided or left-sided *p* value means that the species contents in the two zones are more or less similar than in case where all species were spatially mixed. Phylogenetic trees were reconstructed made in R version 4.0.3 (2020-10-10)^136^. Calculations were made using custom Perl scripts.

#### Evaluation of limiting edaphic factors for plants

To identify edaphic factors which limit the distribution of plant species in the Geysernaya River valley (Tables S17, S18), we analyzed 81 soil properties (some of which were correlated) including particle-size distribution, chemical and mineralogical composition, Eh, pH and water extract composition in combination with the cover values of 28 vascular plant species (group T) recorded at 12 points within the studied catena. We evaluated the importance of each soil property for each of those 28 vascular plants by calculating the correlation (in R environment) between the species cover values and the soil parameter value within a plot. For a monotonic correlation, we applied Spearman’s correlation test from the default functional set of R, and for a non-monotonic correlation, we applied Hoeffding’s test with the “Hmisc” package^143^.

Soil parameters that showed positive or negative correlations with the cover values of species with *p* ≤ 0.01 were considered as important for this species. Soil properties particularly important for obligate and facultative thermophyte species were identified using Fisher’s Exact Test with R^136^, which showed whether the property was important for more thermophyte species than randomly expected.

## Conclusion

The presented data on soil morphology, chemistry and mineralogy can be used for substantiating the taxonomic position of endothermal soils and to characterize the habitats of rare species of organisms. In the Valley of Geysers, depending on soil properties, elemental and mineralogical composition and the degree of hydrothermal alteration—at the macro-, meso- and micro-scales—under the influence of steam-heated acid-sulfate waters, we have distinguished four variants of Andosols. Non-heated Andosols (soils studied at zone I) are considerably different from thermally influenced Andosols in zones II–IV. We propose:

1. Using in the FAO-WRB system^133,144^ of soil temperature as a diagnostic feature that is obligate for soils occurring at/near hydrotherms.
2. Using the supplementary qualifier ‘thermic’ in the FAO-WRB system^133,144^ for soils with a temperature ≥ 50 °C at a depth of 50 cm and the subqualifier hyperthermic for soils with a temperature ≥ 80 °C;
3. Definition of a new diagnostic horizon (e.g., the Narcisic horizon from Latin *Narcissus*, for its beauty) as a horizon consisting of mineral material and all of the following:
  - Bright Munsell color in moist state; hue ≥ 5R, value ≥ 3, chroma 6–8;
  - Soil temperature ≥ 50 °C at a depth of 50 cm;
  - Loamy texture or finer;
  - Mineral composition dominated by kaolinite.
    An additional feature is a fluidal texture in reflected light (Munsell color; hue ≥ 5R, value 2.5–4, chroma 4–8), indicating the presence of intrusive ferruginous pedofeatures.
4. Definition of a new Reference Soil Group—Narcisols having a Narcisic horizon starting ≤ 100 cm from the soil surface.

Despite Narcisols are among the least extensive Reference Soil Groups on earth, they provide many important functions, including highly valuable environmental services: the picturesque thermal landscapes that attract tourists, provide habitats for endangered, unique and protected plant species and microorganisms with unique metabolic pathways. Unfortunately, these unique soils are threatened with destruction serving as renewable or ‘green’ sources of energy, as these soils are fragile and restore slowly.

In the Valley of Geysers, the *Rosaceae, Poales* and *Rosales* species are the most prevalent among the obligate thermal flora. *Poales* species from the *Poaceae* and *Cyperaceae* families are obligate thermophytes rather than facultative thermophytes, which highlights the different mechanisms of these ecological strategies. Pteridophytes tend to be overrepresented in the nearest non-heated habitats to thermal fields due to high humidity. On the phylogenetic tree of Magnoliophyta for the Valley of Geysers, thermophytes form small clusters of closely related thermal species, which are distributed throughout the evolutionary tree. The thermally influenced area can be distinguished from the non-thermally influenced area based on the higher number of Pteridophytes*,* the *Rosaceae, Poaceae* and *Cyperaceae* species. Based on the main characteristics of habitats within the Valley of Geysers, we grouped thermophytes within the following groups:

1. Mesophytes (both obligate and facultative thermophytes):
  - Mostly North Pacific species preferring rather acidic than neutral soils with high contents of Mn and SOC, but low contents of NO_3_^–^, Al, As, Pb (and to a lesser degree Sr) and clay minerals suppressed by sulfates and high electrical conductivity (*Pteridium aquilinum, Thalictrum minus, Angelica gmelinii, Artemisia opulenta, Aruncus dioicus, Cirsium kamtschaticum, Geranium erianthum, Maianthemum dilatatum*);
  - Mostly boreal species preferring cool sandy soils predominantly in Erman’s birch woods and herbaceous plant communities of Kamchatka (*Aruncus dioicus, Maianthemum dilatatum, Pteridium aquilinum, Thalictrum minus*);
  - Mostly boreal species occurring in Eurasia and North America without significant dependence on edaphic factors (*Allium ochotense, Filipendula camtschatica, Galium boreale, Ophioglossum vulgatum, Sedum telephium, Viola selkirkii*).
2. Obligate thermophytes that prefer soils:
  - Enriched in NO_3_^–^ and hematite (*Fimbristylis ochotensis*);
  - With high contents of P, Si (quartz) and goethite, but low sulfate and electrical conductivity (*Potentilla stolonifera*);
  - Enriched in soil organic carbon (*Dactylorhiza aristata*);
  - With low contents of As and Pb (*Epilobium glandulosum*);
  - Without obvious requirements for edaphic factors (*Moehringia lateriflora*).
3. Facultative thermophytes that prefer soils with:
  - A low carbonate alkalinity and low feldspar contents, but high electrical conductivity, sulfate and kaolinite contents (*Agrostis geminata*);
  - A high carbonate alkalinity (*Picris hieracioides*)

Therefore, this paper provides the basis for future transdisciplinary studies closing the gap between pedology and botany within the zone of influence of hydrothermal fields. Such studies are necessary to distinguish the influence of different environmental factors on vegetation, which in turn will help in understanding of the consequences of the global climate change.

## Supplementary Information

Supplementary Information.
