# Supplementary material for: The variability of soils and vegetation of hydrothermal fields in the Valley of Geysers at Kamchatka Peninsula
Source: Sci Rep. 2021 May 26;11:11077. doi: 10.1038/s41598-021-90712-7 (PMC8154911; doi:10.1038/s41598-021-90712-7)
Supplement: Supplementary file 1 — Supplementary Information. [file 41598_2021_90712_MOESM1_ESM.docx]

**The variability of soils and vegetation of hydrothermal fields in the Valley of Geysers at Kamchatka Peninsula**

**Authors**: I.N. Semenkov^1^*, G.V. Klink^2^, M.P. Lebedeva^3^, V.V. Krupskaya^1,4^, M.S. Chernov^1^, O.V. Dorzhieva^4^, M.T. Kazinskiy^1^, V.N. Sokolov^1^, A.V. Zavadskaya^5^

^1^ Lomonosov Moscow State University, Moscow, Russia

^2^ Institute for Information Transmission Problems (Kharkevich Institute) of the Russian Academy of Sciences, Moscow, Russia

^3^ V.V. Dokuchaev Soil Science Institute, Moscow, Russia

^4^ Institute of Geology of Ore Deposits, Petrography, Mineralogy and Geochemistry of the Russian Academy of Sciences (IGEM RAS), Moscow, Russia

^5^ Kronotsky Federal Nature Biosphere Reserve, Elizovo, Russia

*Correspondence to: [semenkov@geogr.msu.ru](mailto:semenkov@geogr.msu.ru), tel. +79264949435

# **Supplementary tables**

## *Table S 1. Selected characteristics of points along the transect studied*

| Zone | Point (cross-section number) | Coordinates | | Temperature, °C | | The date of the temperature measurements |
| --- | --- | --- | --- | --- | --- | --- |
|  |  | N | E | 15 cm | 50 cm |  |
| IV | 1 | 54°26'12.24" | 160°08'14.76" | 98 | 98.7 | 2015.21.08 |
| IV | 2 | 54°26'12.18" | 160°08'14.74" | 98.6 | 99.3* | 2015.21.08 |
| IV | 3 | 54°26'12.13" | 160°08'14.71" | 89.2 | 99.4* | 2015.21.08 |
| IV | 4 | 54°26'12.08" | 160°08'14.72" | 79.1 | 98.4 | 2015.21.08 |
| III | 5 | 54°26'12.05" | 160°08'14.72" | 61.6 | 97.6 | 2015.22.08 |
| III | 6 | 54°26'12.03" | 160°08'14.69" | 44.7 | 82.2 | 2015.22.08 |
| III | 6.1 | 54°26'11.99" | 160°08'14.70" | 61.7 | 95.8 | 2016.17.08 |
| III | 7 | 54°26'11.95" | 160°08'14.69" | 50.9 | 84.8 | 2015.22.08 |
| III | 8 | 54°26'11.90" | 160°08'14.64" | 90.0 | 89.6 | 2015.22.08 |
| III | 9 | 54°26'11.83" | 160°08'14.62" | 59.7 | 96.6 | 2015.22.08 |
| III | 9.1 | 54°26'11.81" | 160°08'14.61" | 54.8 | 81.6 | 2016.17.08 |
| II | 10 | 54°26'11.79" | 160°08'14.60" | 38.3 | 54.3 | 2015.22.08 |
| II | 11 | 54°26'11.73" | 160°08'14.60" | 32.2 | 38.1** | 2015.22.08 |
| II | 12 | 54°26'11.67" | 160°08'14.59" | 29.3 | 37.1*** | 2015.22.08 |
| II | 13 | 54°26'11.59" | 160°08'14.59" | 25.7 | 34.0 | 2015.22.08 |
| I | 14 | 54°26'11.49" | 160°08'14.55" | 22.9 | 26.7 | 2015.22.08 |
| I | 15 | 54°26'11.37" | 160°08'14.55" | 19.7 | 23.3 | 2015.22.08 |
| I | 16 | 54°26'09.55" | 160°08'10.33" | 11.0 | 15.1 | 2015.28.08 |

* 20 cm. ** 30 cm. *** 35 cm

##

## *Table S 2. Main morphological features of Andosols within the catena described according to* ^16^

| Thermal zone – soil name (pit numbers) | Horizon, depth, cm | Average thickness, cm | Munsell color moist | Texture | Structure | Roots | | Boundary | |
| --- | --- | --- | --- | --- | --- | --- | --- | --- | --- |
|  |  |  |  |  |  | A | D, cm | Shape | Distinctness |
| I – Non-heated Eutrosilic Silandic Andosols (Arenic) (14 – 16) | O 0 – 14 | 7 | 10YR 4/3 | na | M | 3f2m1c | 36±11 | Smooth | Abrupt |
|  | A 3 – 27 | 12 | 10YR (2.5 – 5)/(3 – 4) | S | SBC | 3f3m2c | – | Smooth | Distinct |
|  | Bt 10 – 51 | 23 | 2.5Y (5 – 7)/4 | S | M | 1f1m1c | – | – | – |
| II – Slightly heated Eutrosilic Aluandic Andosols (Loamic, Natric) (10 – 13) | O 0–3 | 3 | na | na | SBC | 3f2m | 12±2 | Smooth | Abrupt |
|  | A 2 – 11 | 6 | 10YR (3 – 4)/(2 – 3) | L | SB | 3f3m | – | Smooth | Distinct |
|  | AB 4 – 18 | 8 | 2.5Y (5 – 7)/4 | L | SB | 2f1m | – | Irregular | Diffuse |
|  | Bwn 11+ | na | 2.5Y (7 – 8)/4 | L | SB | 1f | – | – | – |
| III – Moderately heated Eutrosilic Gleyic Aluandic Andosols (Loamic, Reductic, Protosalic, Hyperthionic) (5 – 9) | O 0 – 4 | 3 | na | L | M | 3f | 4±4 | Smooth | Distinct |
|  | A 0 – 7 | 3 | 7.5–10YR (4–8)/(3–6) | L | SB | 2-3f | – | Smooth | Diffuse |
|  | AB 1 – 11 | 4 | 5–7.5YR (5–7)/(2–4) | L | SAB | 1-2f | – | Smooth | Sharp |
|  | Bw 0–12 | 7 | 10YR 7/3 | CL | M | – | – | Smooth | Sharp |
|  | Bl 4 – 54 | 28 | 5–7.5(Y)R (5–7)/(2–6) | CL | P | – | – | Smooth | Sharp |
|  | Cr 13+ | na | 7–9/N | C | M | – | – | – | – |
| IV – Hot Gleyic Aluandic Andosols (Clayic, Reductic, Salic, Hyperthionic) (1 – 4) | Az 0–0.8 | 0.6 | 2.5R 7/3 | C | M | – | 0 | Smooth | Distinct |
|  | Bjlz 1–10 | 3 | 2.5–10YR(5–8)/4 | C | M | – | – | Wavy | Abrupt |
|  | Crz 3+ | na | B(P,G), N (5–8)/(1–5) | C | M | – | – | – | – |

Notes: na – not applicable. Texture classes: C – Clay, CL – Clay loam, L – Loam, S – sand. Structure: M – massive, SBC – fine subangular blocky-crumbly, SB – subangular blocky, SAB – subangular-angular blocky, P – coarse blocky-prismatic. Roots, A: f — fine, m — medium, c — coarse, 1 — single, 2 — few, 3 — many. Roots, D: depth range from the surface of plant roots.

## *Table S 3. Topsoil (0 – 10 cm) properties in zones I – IV*

| **Properties** | | **Zone** | **Me** | **MAD** | **M** | **SD** | **Normality test p–value** | | | **n** |
| --- | --- | --- | --- | --- | --- | --- | --- | --- | --- | --- |
|  |  |  |  |  |  |  | **Shapiro** | **Lillie** | **Anderson** |  |
| Physico–chemical properties | SOC, g/100g | I | 8.9 | 3.2 | 11.0 | 6.6 | 0.240 | NA | NA | 4 |
|  |  | II | 5.1 | 6.1 | 5.2 | 4.0 | 0.086 | 0.372 | 0.128 | 8 |
|  |  | III | 1.7 | 2.0 | 3.3 | 3.8 | 0.002 | 0.007 | 0.002 | 17 |
|  |  | IV | 1.3 | 0.9 | 1.9 | 1.6 | 0.075 | 0.089 | NA | 6 |
|  | pH | I | 5.7 | 0.4 | 5.7 | 0.4 | 0.796 | NA | NA | 4 |
|  |  | II | 5.7 | 0.4 | 5.8 | 0.5 | 0.534 | 0.815 | 0.524 | 9 |
|  |  | III | 3.7 | 0.4 | 3.8 | 0.7 | 0.030 | 0.010 | 0.013 | 22 |
|  |  | IV | 3.4 | 0.5 | 3.4 | 0.4 | 0.774 | 0.538 | 0.682 | 8 |
|  | Eh, mV | I | –35 | 305 | –1 | 289 | 0.429 | NA | NA | 4 |
|  |  | II | –239 | 102 | –83 | 308 | 0.017 | 0.126 | 0.020 | 8 |
|  |  | III | 602 | 52 | 520 | 265 | 0.000 | 0.000 | 0.000 | 22 |
|  |  | IV | 613 | 28 | 610 | 22 | 0.575 | 0.783 | 0.658 | 8 |
|  | EC,µS/cm | I | 192 | 61 | 187 | 64 | 0.958 | NA | NA | 4 |
|  |  | II | 195 | 82 | 172 | 75 | 0.415 | 0.597 | 0.393 | 9 |
|  |  | III | 292 | 213 | 481 | 464 | 0.000 | 0.001 | 0.000 | 22 |
|  |  | IV | 2240 | 1263 | 2279 | 962 | 0.420 | 0.669 | 0.551 | 8 |
|  | LOI | I | 23 | 14 | 26 | 14 | NA | NA | NA | 3 |
|  |  | II | 17 | 9 | 15 | 8 | 0.395 | 0.534 | 0.409 | 9 |
|  |  | III | 10.8 | 4.6 | 14.4 | 10.5 | 0.000 | 0.007 | 0.000 | 20 |
|  |  | IV | 12 | 3 | 12 | 2 | 0.387 | 0.459 | NA | 7 |
| Grain–size fractions, vol. % | PM_1000–500_ | I | 38 | 2 | 41 | 7 | 0.035 | NA | NA | 4 |
|  |  | II | 25 | 17 | 28 | 18 | 0.729 | 0.398 | 0.563 | 9 |
|  |  | III | 15 | 22 | 20 | 19 | 0.014 | 0.108 | 0.027 | 22 |
|  |  | IV | 0 | 0 | 2.05 | 4.27 | 0.000 | 0.004 | NA | 7 |
|  | PM_500–250_ | I | 27 | 2 | 27 | 2 | 0.757 | NA | NA | 4 |
|  |  | II | 15 | 4 | 14 | 7 | 0.362 | 0.420 | 0.331 | 9 |
|  |  | III | 12 | 10 | 14 | 11 | 0.131 | 0.229 | 0.217 | 22 |
|  |  | IV | 0 | 0 | 3.37 | 7.57 | 0.000 | 0.002 | NA | 7 |
|  | PM_250–50_ | I | 22 | 2 | 21 | 3 | 0.500 | NA | NA | 4 |
|  |  | II | 20 | 7 | 20 | 6 | 0.976 | 0.865 | 0.911 | 9 |
|  |  | III | 16 | 6 | 14 | 8 | 0.059 | 0.119 | 0.072 | 22 |
|  |  | IV | 0.70 | 0.74 | 3.73 | 5.59 | 0.002 | 0.001 | NA | 7 |
|  | PM_50–10_ | I | 8.2 | 1.9 | 8.3 | 1.9 | 0.935 | NA | NA | 4 |
|  |  | II | 19 | 8 | 24 | 14 | 0.048 | 0.173 | 0.056 | 9 |
|  |  | III | 17 | 10 | 19 | 10 | 0.614 | 0.725 | 0.406 | 22 |
|  |  | IV | 23 | 4 | 25 | 9 | 0.757 | 0.478 | NA | 7 |
|  | PM_10–5_ | I | 0.90 | 0.30 | 0.98 | 0.39 | 0.712 | NA | NA | 4 |
|  |  | II | 4.7 | 2.7 | 5.0 | 2.4 | 0.470 | 0.338 | 0.468 | 9 |
|  |  | III | 6.1 | 4.7 | 6.8 | 4.1 | 0.039 | 0.518 | 0.112 | 22 |
|  |  | IV | 9.5 | 8.5 | 10.1 | 7.6 | 0.499 | 0.343 | NA | 7 |
|  | PM_5–1_ | I | 0.90 | 0.15 | 0.98 | 0.30 | 0.279 | NA | NA | 4 |
|  |  | II | 5.7 | 4.2 | 5.6 | 3.0 | 0.722 | 0.780 | 0.762 | 9 |
|  |  | III | 9.9 | 9.5 | 15.3 | 14.1 | 0.002 | 0.001 | 0.001 | 22 |
|  |  | IV | 35 | 13 | 31 | 11 | 0.676 | 0.300 | NA | 7 |
|  | PM_1_ | I | 0.20 | 0.00 | 0.23 | 0.05 | 0.001 | NA | NA | 4 |
|  |  | II | 1.3 | 0.9 | 1.3 | 0.8 | 0.693 | 0.819 | 0.672 | 9 |
|  |  | III | 3.0 | 3.0 | 8.2 | 11.2 | 0.000 | 0.000 | 0.000 | 22 |
|  |  | IV | 17 | 11 | 18 | 8 | 0.461 | 0.680 | NA | 7 |
| Major elements, g/100g | Al_2_O_3_ | I | 16 | 2 | 15 | 4 | 0.414 | NA | NA | 4 |
|  |  | II | 18 | 3 | 19 | 4 | 0.208 | 0.761 | 0.305 | 9 |
|  |  | III | 29 | 13 | 30 | 15 | 0.016 | 0.234 | 0.054 | 22 |
|  |  | IV | 45 | 4 | 45 | 5 | 0.553 | 0.879 | 0.513 | 8 |
|  | CaO | I | 5.0 | 0.0 | 5.0 | 0.0 | NA | NA | NA | 4 |
|  |  | II | 4.0 | 0.0 | 3.5 | 0.6 | 0.004 | 0.001 | 0.003 | 8 |
|  |  | III | 1.00 | 1.11 | 0.96 | 1.03 | 0.001 | 0.000 | 0.000 | 22 |
|  |  | IV | 0 | 0 | 0.03 | 0.06 | 0.000 | 0.000 | 0.000 | 8 |
|  | Fe_2_O_3_ | I | 8.6 | 0.5 | 8.1 | 1.3 | 0.159 | NA | NA | 4 |
|  |  | II | 7.9 | 0.4 | 7.9 | 0.8 | 0.093 | 0.084 | 0.117 | 9 |
|  |  | III | 7.6 | 2.3 | 8.8 | 5.3 | 0.001 | 0.000 | 0.000 | 22 |
|  |  | IV | 25 | 12 | 25 | 8 | 0.294 | 0.678 | 0.462 | 8 |
|  | K_2_O | I | 1.1 | 0.1 | 1.1 | 0.1 | 0.637 | NA | NA | 4 |
|  |  | II | 1.8 | 0.1 | 1.7 | 0.2 | 0.012 | 0.074 | 0.013 | 8 |
|  |  | III | 0.82 | 0.53 | 0.80 | 0.51 | 0.149 | 0.438 | 0.280 | 22 |
|  |  | IV | 0.18 | 0.04 | 0.23 | 0.14 | 0.010 | 0.002 | 0.008 | 8 |
|  | MgO | I | 1.6 | 0.1 | 1.5 | 0.1 | 0.263 | NA | NA | 4 |
|  |  | II | 1.5 | 0.0 | 1.5 | 0.1 | 0.048 | 0.006 | 0.036 | 8 |
|  |  | III | 0.79 | 0.28 | 0.80 | 0.33 | 0.563 | 0.560 | 0.397 | 22 |
|  |  | IV | 0.60 | 0.41 | 0.57 | 0.29 | 0.046 | 0.205 | 0.055 | 8 |
|  | MnO | I | 2001 | 64 | 1867 | 323 | 0.027 | NA | NA | 4 |
|  |  | II | 1861 | 79 | 1859 | 62 | 0.184 | 0.248 | 0.173 | 8 |
|  |  | III | 508 | 253 | 663 | 540 | 0.004 | 0.002 | 0.002 | 22 |
|  |  | IV | 451 | 182 | 488 | 255 | 0.346 | 0.521 | 0.340 | 8 |
|  | P_2_O_5_ | I | 0.48 | 0.05 | 0.50 | 0.09 | 0.445 | NA | NA | 4 |
|  |  | II | 0.35 | 0.08 | 0.32 | 0.10 | 0.169 | 0.104 | 0.165 | 8 |
|  |  | III | 0.26 | 0.10 | 0.26 | 0.09 | 0.713 | 0.520 | 0.689 | 22 |
|  |  | IV | 0.20 | 0.17 | 0.25 | 0.16 | 0.477 | 0.403 | 0.483 | 8 |
|  | SiO_2_ | I | 56 | 1 | 55 | 1 | 0.272 | NA | NA | 4 |
|  |  | II | 60 | 2 | 61 | 3 | 0.039 | 0.398 | 0.065 | 8 |
|  |  | III | 62 | 3 | 56 | 17 | 0.000 | 0.000 | 0.000 | 22 |
|  |  | IV | 30 | 11 | 27 | 10 | 0.621 | 0.863 | 0.688 | 8 |
|  | TiO_2_ | I | 1.1 | 0.1 | 1.0 | 0.2 | 0.251 | NA | NA | 4 |
|  |  | II | 1.2 | 0.1 | 1.1 | 0.1 | 0.291 | 0.156 | 0.260 | 9 |
|  |  | III | 1.6 | 0.3 | 1.5 | 0.5 | 0.654 | 0.249 | 0.448 | 22 |
|  |  | IV | 2.3 | 0.5 | 2.2 | 0.5 | 0.774 | 0.804 | 0.735 | 8 |
| Micro elements, mg/kg | As | I | 6.5 | 2.2 | 6.5 | 2.1 | 0.995 | NA | NA | 4 |
|  |  | II | 8.0 | 0.7 | 7.6 | 1.4 | 0.045 | 0.004 | 0.030 | 8 |
|  |  | III | 12 | 4 | 21 | 19 | 0.000 | 0.000 | 0.000 | 22 |
|  |  | IV | 18 | 2 | 22 | 12 | 0.123 | 0.010 | 0.042 | 8 |
|  | Co | I | 36 | 12 | 33 | 16 | 0.679 | NA | NA | 4 |
|  |  | II | 27 | 16 | 27 | 10 | 0.237 | 0.617 | 0.392 | 8 |
|  |  | III | 31 | 22 | 111 | 188 | 0.000 | 0.000 | 0.000 | 22 |
|  |  | IV | 930 | 340 | 752 | 426 | 0.178 | 0.181 | 0.170 | 8 |
|  | Cr | I | 49 | 1 | 47 | 5 | 0.065 | NA | NA | 4 |
|  |  | II | 45 | 1 | 44 | 1 | 0.041 | 0.034 | 0.043 | 8 |
|  |  | III | 48 | 4 | 57 | 30 | 0.000 | 0.000 | 0.000 | 22 |
|  |  | IV | 94 | 27 | 93 | 25 | 0.739 | 0.856 | 0.827 | 8 |
|  | Cu | I | 26 | 2 | 22 | 10 | 0.041 | NA | NA | 4 |
|  |  | II | 24 | 5 | 28 | 9 | 0.091 | 0.020 | 0.061 | 8 |
|  |  | III | 32 | 10 | 65 | 69 | 0.000 | 0.000 | 0.000 | 22 |
|  |  | IV | 65 | 42 | 86 | 60 | 0.048 | 0.168 | 0.059 | 8 |
|  | Ni | I | 38 | 1 | 37 | 1 | 0.272 | NA | NA | 4 |
|  |  | II | 35 | 1 | 35 | 2 | 0.773 | 0.469 | 0.575 | 8 |
|  |  | III | 36 | 7 | 43 | 20 | 0.000 | 0.000 | 0.000 | 22 |
|  |  | IV | 22 | 26 | 23 | 19 | 0.444 | 0.503 | 0.541 | 8 |
|  | Pb | I | 20 | 9 | 19 | 9 | 0.929 | NA | NA | 4 |
|  |  | II | 25 | 18 | 25 | 12 | 0.361 | 0.488 | 0.423 | 8 |
|  |  | III | 63 | 34 | 93 | 82 | 0.000 | 0.007 | 0.000 | 22 |
|  |  | IV | 91 | 22 | 98 | 55 | 0.519 | 0.156 | 0.275 | 8 |
|  | Sr | I | 237 | 10 | 241 | 14 | 0.322 | NA | NA | 4 |
|  |  | II | 286 | 27 | 291 | 31 | 0.095 | 0.028 | 0.098 | 8 |
|  |  | III | 320 | 100 | 412 | 217 | 0.001 | 0.001 | 0.000 | 22 |
|  |  | IV | 230 | 202 | 215 | 141 | 0.501 | 0.722 | 0.613 | 8 |
|  | V | I | 154 | 11 | 141 | 32 | 0.084 | NA | NA | 4 |
|  |  | II | 145 | 16 | 142 | 14 | 0.214 | 0.535 | 0.272 | 9 |
|  |  | III | 196 | 70 | 196 | 60 | 0.667 | 0.461 | 0.475 | 22 |
|  |  | IV | 214 | 56 | 208 | 60 | 0.710 | 0.845 | 0.709 | 8 |
|  | Zn | I | 143 | 14 | 163 | 51 | 0.053 | NA | NA | 4 |
|  |  | II | 138 | 21 | 144 | 20 | 0.310 | 0.580 | 0.360 | 8 |
|  |  | III | 118 | 24 | 190 | 160 | 0.000 | 0.000 | 0.000 | 22 |
|  |  | IV | 150 | 103 | 162 | 90 | 0.227 | 0.689 | 0.317 | 8 |
| Mineral composition, g/100g | Albite | I | 6.5 | 0.0 | 7.0 | 0.8 | NA | NA | NA | 3 |
|  |  | II | 4.4 | 2.5 | 5.5 | 3.2 | 0.228 | 0.128 | 0.219 | 10 |
|  |  | III | 0.8 | 1.2 | 1.8 | 2.4 | 0.001 | 0.015 | 0.001 | 18 |
|  |  | IV | <0.1 | 0 | 1.2 | 2.0 | 0.001 | 0.033 | 0.002 | 9 |
|  | Anatase | I | 0.3 | <0.1 | 0.23 | 0.1 | NA | NA | NA | 3 |
|  |  | II | 0.8 | 0.2 | 1.0 | 0.9 | 0.000 | 0.000 | 0.000 | 10 |
|  |  | III | 3.9 | 4.2 | 5.2 | 4.6 | 0.027 | 0.429 | 0.063 | 18 |
|  |  | IV | 8.0 | 3.4 | 7.2 | 3.7 | 0.383 | 0.665 | 0.374 | 9 |
|  | Anorthite | I | 39 | 0 | 39 | 1 | NA | NA | NA | 3 |
|  |  | II | 16 | 3 | 17 | 7 | 0.461 | 0.163 | 0.219 | 10 |
|  |  | III | 1.0 | 1.5 | 3.8 | 4.5 | 0.002 | 0.002 | 0.001 | 18 |
|  |  | IV | <0.1 | 0 | 1.0 | 2.1 | 0.000 | 0.002 | 0.000 | 9 |
|  | Boehmite | I | <0.1 | 0 | <0.1 | 0 | NA | NA | NA | 3 |
|  |  | II | <0.1 | 0 | <0.1 | 0 | NA | NA | NA | 10 |
|  |  | III | <0.1 | 0 | 1.2 | 5.1 | 0.000 | 0.000 | 0.000 | 18 |
|  |  | IV | 3.6 | 4.5 | 3.4 | 3.0 | 0.074 | 0.311 | 0.125 | 9 |
|  | Calcite | I | <0.1 | 0 | <0.1 | 0 | NA | NA | NA | 3 |
|  |  | II | <0.1 | 0 | <0.1 | 0 | NA | NA | NA | 10 |
|  |  | III | <0.1 | 0 | 0.1 | 0.4 | 0.000 | 0.000 | 0.000 | 18 |
|  |  | IV | <0.1 | 0 | 0.5 | 1.0 | 0.000 | 0.000 | 0.000 | 9 |
|  | Clinoptilolite | I | 7.9 | 0.0 | 7.2 | 1.2 | NA | NA | NA | 3 |
|  |  | II | 11 | 6 | 11 | 4 | 0.421 | 0.888 | 0.563 | 10 |
|  |  | III | 5.6 | 7.3 | 7.0 | 5.7 | 0.068 | 0.346 | 0.123 | 18 |
|  |  | IV | 0 | 0 | 1.9 | 4.4 | 0.000 | 0.000 | 0.000 | 9 |
|  | Cristobalite | I | 0 | 0 | 1.7 | 3.0 | NA | NA | NA | 3 |
|  |  | II | 1.0 | 0.8 | 2.3 | 2.7 | 0.007 | 0.017 | 0.005 | 10 |
|  |  | III | 0.5 | 0.7 | 3.4 | 6.0 | 0.000 | 0.000 | 0.000 | 18 |
|  |  | IV | 2.4 | 3.6 | 7.9 | 9.7 | 0.014 | 0.025 | 0.013 | 9 |
|  | Goethite | I | <0.1 | 0 | <0.1 | 0 | NA | NA | NA | 3 |
|  |  | II | <0.1 | 0 | 0.2 | 0.3 | 0.000 | 0.000 | 0.000 | 10 |
|  |  | III | <0.1 | 0 | 0.2 | 0.6 | 0.000 | 0.000 | 0.000 | 18 |
|  |  | IV | 0.7 | 1.0 | 3.4 | 4.3 | 0.029 | 0.028 | 0.032 | 9 |
|  | Hematite | I | <0.1 | 0 | <0.1 | 0 | NA | NA | NA | 3 |
|  |  | II | <0.1 | 0 | 0.1 | 0.1 | 0.000 | 0.000 | 0.000 | 10 |
|  |  | III | <0.1 | 0 | 1.2 | 2.5 | 0.000 | 0.000 | 0.000 | 18 |
|  |  | IV | 3.1 | 3.9 | 4.3 | 5.2 | 0.029 | 0.054 | 0.035 | 9 |
|  | Heulandite | I | <0.1 | 0 | 1.0 | 1.8 | NA | NA | NA | 3 |
|  |  | II | <0.1 | 0 | 0.8 | 1.7 | 0.000 | 0.000 | 0.000 | 10 |
|  |  | III | <0.1 | 0 | 1.0 | 3.0 | 0.000 | 0.000 | 0.000 | 18 |
|  |  | IV | <0.1 | 0 | <0.1 | 0 | NA | NA | NA | 9 |
|  | Jarosite | I | <0.1 | 0 | <0.1 | 0 | NA | NA | NA | 3 |
|  |  | II | <0.1 | 0 | <0.1 | 0 | NA | NA | NA | 10 |
|  |  | III | <0.1 | 0 | <0.1 | 0 | NA | NA | NA | 18 |
|  |  | IV | <0.1 | 0 | 0.3 | 0.7 | 0.000 | 0.000 | 0.000 | 9 |
|  | Kaolinite | I | 6.3 | 0.0 | 5.9 | 0.7 | NA | NA | NA | 3 |
|  |  | II | 5.6 | 2.4 | 8.4 | 7.9 | 0.000 | 0.010 | 0.001 | 10 |
|  |  | III | 50 | 27 | 48 | 27 | 0.775 | 0.752 | 0.863 | 18 |
|  |  | IV | 55 | 24 | 54 | 21 | 0.713 | 0.945 | 0.826 | 9 |
|  | Marcasite | I | <0.1 | 0 | <0.1 | 0 | NA | NA | NA | 3 |
|  |  | II | <0.1 | 0 | <0.1 | 0 | NA | NA | NA | 10 |
|  |  | III | <0.1 | 0 | <0.1 | 0 | NA | NA | NA | 18 |
|  |  | IV | <0.1 | 0 | 0.1 | 0.1 | 0.000 | 0.000 | 0.000 | 9 |
|  | Microcline | I | 10.4 | <0.1 | 10.0 | 0.8 | NA | NA | NA | 3 |
|  |  | II | 10.5 | 2.1 | 11.4 | 2.1 | 0.312 | 0.074 | 0.232 | 10 |
|  |  | III | 5.6 | 4.1 | 5.7 | 3.8 | 0.359 | 0.246 | 0.389 | 18 |
|  |  | IV | 1.2 | 1.8 | 1.6 | 1.5 | 0.373 | 0.613 | 0.435 | 9 |
|  | Pyrite | I | 0.5 | <0.1 | 0.6 | 0.2 | NA | NA | NA | 3 |
|  |  | II | 0.3 | 0.2 | 0.2 | 0.1 | 0.198 | 0.035 | 0.139 | 10 |
|  |  | III | 0.2 | 0.3 | 0.5 | 1.3 | 0.000 | 0.000 | 0.000 | 18 |
|  |  | IV | 0.6 | 0.9 | 1.4 | 2.7 | 0.000 | 0.000 | 0.000 | 9 |
|  | Pyrolusite | I | <0.1 | 0 | <0.1 | 0 | NA | NA | NA | 3 |
|  |  | II | <0.1 | 0 | <0.1 | 0 | NA | NA | NA | 10 |
|  |  | III | <0.1 | 0 | <0.1 | 0 | NA | NA | NA | 18 |
|  |  | IV | <0.1 | 0 | 0.1 | 0.4 | 0.000 | 0.000 | 0.000 | 9 |
|  | Quartz | I | 3.4 | 0.0 | 3.2 | 0.3 | NA | NA | NA | 3 |
|  |  | II | 14 | 3 | 15 | 3 | 0.362 | 0.245 | 0.302 | 10 |
|  |  | III | 12 | 11 | 9 | 8 | 0.006 | 0.065 | 0.004 | 18 |
|  |  | IV | 8.2 | 9.2 | 8.0 | 6.0 | 0.222 | 0.449 | 0.275 | 9 |
|  | Smectite | I | 26 | 0 | 25 | 2 | NA | NA | NA | 3 |
|  |  | II | 28 | 6 | 28 | 6 | 0.973 | 0.632 | 0.912 | 10 |
|  |  | III | 5.8 | 8.5 | 11.5 | 12.9 | 0.002 | 0.000 | 0.001 | 18 |
|  |  | IV | 0 | 0 | 3.7 | 11.0 | 0.000 | 0.000 | 0.000 | 9 |
| Short-range-order Al, Fe and Si compounds, g/kg | Al_ox_ | I | 6.6 | 0.0 | 6.6 | NA | NA | NA | NA | 1 |
|  |  | II | 3.4 | 0.9 | 3.4 | 0.8 | NA | NA | NA | 2 |
|  |  | III | 1.9 | 0.4 | 2.7 | 1.4 | 0.076 | 0.134 | NA | 5 |
|  |  | IV | 2.8 | 0.4 | 2.9 | 0.5 | 0.780 | NA | NA | 4 |
|  | Fe_ox_ | I | 9.4 | 0.0 | 9.4 | NA | NA | NA | NA | 1 |
|  |  | II | 5.7 | 2.4 | 5.7 | 2.3 | NA | NA | NA | 2 |
|  |  | III | 5.9 | 0.9 | 5.9 | 1.2 | 0.800 | 0.577 | NA | 5 |
|  |  | IV | 19 | 7 | 18 | 8 | 0.804 | NA | NA | 4 |
|  | Si_ox_ | I | 2.0 | 0.0 | 2.0 | NA | NA | NA | NA | 1 |
|  |  | II | 1.2 | 0.1 | 1.2 | 0.1 | NA | NA | NA | 2 |
|  |  | III | 0.4 | 0.1 | 0.4 | 0.1 | 0.276 | 0.585 | NA | 5 |
|  |  | IV | 0.3 | 0.2 | 0.3 | 0.2 | 0.971 | NA | NA | 4 |
| Cation composition, mmol (+)/100g | Ca^2+^ | I | 37 | 9 | 37 | 9 | NA | NA | NA | 2 |
|  |  | II | 36 | 11 | 35 | 13 | 0.988 | 0.793 | 0.914 | 10 |
|  |  | III | 30 | 8 | 35 | 18 | 0.001 | 0.000 | 0.000 | 22 |
|  |  | IV | 30 | 14 | 46 | 33 | 0.042 | 0.139 | NA | 7 |
|  | K^+^ | I | 6.7 | 0.2 | 6.7 | 0.2 | NA | NA | NA | 2 |
|  |  | II | 3.1 | 3.4 | 3.7 | 2.3 | 0.105 | 0.112 | 0.105 | 10 |
|  |  | III | 2.0 | 1.5 | 2.1 | 1.5 | 0.169 | 0.802 | 0.403 | 22 |
|  |  | IV | 0.84 | 0.61 | 1.11 | 0.79 | 0.108 | 0.522 | NA | 7 |
|  | Mg^2+^ | I | 15 | 2 | 15 | 2 | NA | NA | NA | 2 |
|  |  | II | 12 | 6 | 12 | 6 | 0.499 | 0.617 | 0.468 | 10 |
|  |  | III | 9.6 | 4.8 | 11.0 | 7.0 | 0.001 | 0.030 | 0.007 | 22 |
|  |  | IV | 14 | 10 | 32 | 37 | 0.006 | 0.123 | NA | 7 |
|  | Na^+^ | I | 4.2 | 0.9 | 4.2 | 0.8 | NA | NA | NA | 2 |
|  |  | II | 4.9 | 1.1 | 5.3 | 1.5 | 0.191 | 0.340 | 0.165 | 10 |
|  |  | III | 5.6 | 4.0 | 9.1 | 11.3 | 0.000 | 0.000 | 0.000 | 22 |
|  |  | IV | 73 | 14 | 56 | 38 | 0.028 | 0.063 | NA | 7 |
|  | NH_4_^+^ | I | 1.8 | 0.2 | 1.8 | 0.2 | NA | NA | NA | 2 |
|  |  | II | 0.032 | 0.000 | 0.714 | 1.119 | 0.000 | 0.000 | 0.000 | 10 |
|  |  | III | 1.1 | 1.6 | 4.3 | 5.6 | 0.000 | 0.000 | 0.000 | 22 |
|  |  | IV | 2.8 | 4.1 | 4.3 | 5.9 | 0.026 | 0.282 | NA | 7 |
| Anion composition, mmol (–)/100g | Cl^–^ | I | 1.4 | 0.0 | 1.4 | 0.0 | NA | NA | NA | 2 |
|  |  | II | 1.2 | 0.3 | 1.3 | 0.4 | 0.601 | 0.612 | 0.457 | 10 |
|  |  | III | 1.4 | 0.5 | 1.8 | 1.2 | 0.000 | 0.001 | 0.000 | 22 |
|  |  | IV | 1.4 | 0.7 | 1.6 | 1.1 | 0.134 | 0.126 | NA | 7 |
|  | F^–^ | I | 0.60 | 0.39 | 0.60 | 0.37 | NA | NA | NA | 2 |
|  |  | II | 2.3 | 3.0 | 3.0 | 3.9 | 0.003 | 0.042 | 0.007 | 10 |
|  |  | III | 1.9 | 1.4 | 2.0 | 1.5 | 0.020 | 0.285 | 0.138 | 22 |
|  |  | IV | 1.3 | 0.9 | 1.3 | 0.6 | 0.837 | 0.970 | NA | 7 |
|  | HCO_3_^–^ | I | 0.31 | 0.10 | 0.37 | 0.20 | 0.305 | NA | NA | 4 |
|  |  | II | 0.38 | 0.18 | 0.59 | 0.44 | 0.050 | 0.101 | 0.044 | 9 |
|  |  | III | <0.1 | 0 | 0.1 | 0.2 | 0.000 | 0.000 | 0.000 | 20 |
|  |  | IV | <0.1 | 0 | <0.1 | 0 | NA | NA | NA | 8 |
|  | HPO_4_^2–^ | I | <0.01 | 0 | <0.01 | 0 | NA | NA | NA | 2 |
|  |  | II | <0.01 | 0 | 0.05 | 0.13 | 0.000 | 0.000 | 0.000 | 10 |
|  |  | III | <0.01 | 0 | <0.01 | 0.00 | 0.000 | 0.000 | 0.000 | 22 |
|  |  | IV | <0.01 | 0 | 0.03 | 0.06 | 0.000 | 0.002 | NA | 7 |
|  | NO_3_^–^ | I | 0.035 | 0.021 | 0.035 | 0.021 | NA | NA | NA | 2 |
|  |  | II | 0.086 | 0.103 | 0.191 | 0.278 | 0.001 | 0.003 | 0.001 | 10 |
|  |  | III | 1.2 | 1.6 | 1.7 | 1.8 | 0.004 | 0.046 | 0.004 | 22 |
|  |  | IV | 0.17 | 0.12 | 0.14 | 0.09 | 0.714 | 0.377 | NA | 7 |
|  | SO_4_^2–^ | I | 1.02 | 0.51 | 1.02 | 0.49 | NA | NA | NA | 2 |
|  |  | II | 0.62 | 0.39 | 0.97 | 0.83 | 0.002 | 0.046 | 0.003 | 10 |
|  |  | III | 8.2 | 8.9 | 17.0 | 21.6 | 0.000 | 0.000 | 0.000 | 22 |
|  |  | IV | 59 | 43 | 88 | 64 | 0.323 | 0.227 | NA | 7 |

Me – median, MAD – median absolute deviation, M – mean, SD – standard deviation

## *Table S 4. Subsoil properties within the studied catena*

| **Properties** | | **Zone** | **Depth, cm** | **Me** | **MAD** | **M** | **SD** | **Normality test p–value** | | | **N** |
| --- | --- | --- | --- | --- | --- | --- | --- | --- | --- | --- | --- |
|  |  |  |  |  |  |  |  | **Shapiro** | **Lillie** | **Anderson** |  |
| Physico–chemical properties | EC, µS/cm | I | 10–20 | 94 | 99 | 94 | 94 | NA | NA | NA | 2 |
|  |  | I | 20–200 | 50 | 31 | 53 | 27 | 0.323 | 0.589 | 0.411 | 9 |
|  |  | I | 200–300 | 94 | 48 | 94 | 46 | NA | NA | NA | 2 |
|  |  | II | 10–20 | 64 | 18 | 66 | 14 | 0.804 | 0.879 | NA | 5 |
|  |  | II | 20–200 | 48 | 0 | 48 | NA | NA | NA | NA | 1 |
|  |  | III | 10–20 | 153 | 7 | 175 | 50 | 0.015 | NA | NA | 4 |
|  | Eh, mV | I | 10–20 | 335 | 34 | 335 | 33 | NA | NA | NA | 2 |
|  |  | I | 20–200 | 506 | 36 | 487 | 61 | 0.018 | 0.041 | 0.025 | 9 |
|  |  | I | 200–300 | 497 | 10 | 497 | 10 | NA | NA | NA | 2 |
|  |  | II | 10–20 | 510 | 31 | 417 | 210 | 0.001 | 0.003 | NA | 5 |
|  |  | II | 20–200 | 547 | 0 | 547 | NA | NA | NA | NA | 1 |
|  |  | III | 10–20 | 624 | 28 | 607 | 52 | 0.231 | NA | NA | 4 |
|  | pH | I | 10–20 | 5.9 | 0.5 | 5.9 | 0.5 | NA | NA | NA | 2 |
|  |  | I | 20–200 | 6.3 | 0.1 | 6.1 | 0.5 | 0.038 | 0.044 | 0.022 | 9 |
|  |  | I | 200–300 | 6.8 | 0.8 | 6.8 | 0.8 | NA | NA | NA | 2 |
|  |  | II | 10–20 | 5.9 | 0.1 | 6.0 | 0.3 | 0.190 | 0.235 | NA | 5 |
|  |  | II | 20–200 | 6.3 | 0.0 | 6.3 | NA | NA | NA | NA | 1 |
|  |  | III | 10–20 | 4.0 | 0.2 | 3.9 | 0.4 | 0.405 | NA | NA | 4 |
|  | LOI, g/100g | I | 10–20 | 13 | 13 | 13 | 13 | NA | NA | NA | 2 |
|  |  | I | 20–200 | 6.5 | 3.0 | 5.6 | 2.9 | 0.925 | 0.641 | NA | 7 |
|  |  | I | 200–300 | 5.7 | 0.5 | 5.7 | 0.4 | NA | NA | NA | 2 |
|  |  | II | 10–20 | 6.2 | 0.4 | 5.6 | 1.6 | 0.045 | NA | NA | 4 |
|  |  | II | 20–200 | 4.2 | 0.0 | 4.2 | NA | NA | NA | NA | 1 |
|  |  | III | 10–20 | 10.0 | 1.9 | 8.5 | 4.6 | 0.185 | NA | NA | 4 |
|  | SOC, g/100g | I | 10–20 | 9.1 | 2.3 | 9.1 | 2.2 | NA | NA | NA | 2 |
|  |  | I | 20–200 | 0.52 | 0.49 | 1.57 | 2.72 | 0.000 | 0.008 | 0.000 | 9 |
|  |  | I | 200–300 | 0.30 | 0.41 | 0.30 | 0.39 | NA | NA | NA | 2 |
|  |  | II | 10–20 | 0.29 | 0.04 | 0.46 | 0.46 | 0.003 | 0.002 | NA | 5 |
|  |  | II | 20–200 | 5.2 | 0.0 | 5.2 | NA | NA | NA | NA | 1 |
|  |  | III | 10–20 | 0.17 | 0.01 | 0.17 | 0.01 | NA | NA | NA | 2 |
| Grain–size fractions, vol. % | PM_<1_ | I | 10–20 | 0.30 | 0.15 | 0.30 | 0.14 | NA | NA | NA | 2 |
|  |  | I | 20–200 | 0.90 | 1.19 | 1.11 | 1.01 | 0.148 | 0.110 | 0.171 | 9 |
|  |  | I | 200–300 | 4.3 | 2.7 | 4.3 | 2.5 | NA | NA | NA | 2 |
|  |  | II | 10–20 | 2.8 | 0.9 | 3.0 | 0.6 | 0.827 | 0.691 | NA | 5 |
|  |  | II | 20–200 | 1.6 | 0.0 | 1.6 | NA | NA | NA | NA | 1 |
|  |  | III | 10–20 | 30 | 16 | 30 | 13 | 0.160 | NA | NA | 4 |
|  | PM_1000-500_ | I | 10–20 | 44 | 27 | 44 | 25 | NA | NA | NA | 2 |
|  |  | I | 20–200 | 37 | 29 | 39 | 29 | 0.508 | 0.942 | 0.695 | 9 |
|  |  | I | 200–300 | 4.2 | 6.2 | 4.2 | 5.9 | NA | NA | NA | 2 |
|  |  | II | 10–20 | 11 | 5 | 12 | 10 | 0.709 | 0.333 | NA | 5 |
|  |  | II | 20–200 | 33 | 0 | 33 | NA | NA | NA | NA | 1 |
|  |  | III | 10–20 | <0.1 | 0 | 0.2 | 0.4 | 0.001 | NA | NA | 4 |
|  | PM_10-5_ | I | 10–20 | 1.4 | 0.9 | 1.4 | 0.8 | NA | NA | NA | 2 |
|  |  | I | 20–200 | 2.1 | 2.7 | 3.1 | 3.2 | 0.092 | 0.130 | 0.108 | 9 |
|  |  | I | 200–300 | 10.4 | 5.0 | 10.4 | 4.8 | NA | NA | NA | 2 |
|  |  | II | 10–20 | 7.2 | 1.2 | 8.6 | 3.1 | 0.105 | 0.221 | NA | 5 |
|  |  | II | 20–200 | 5.1 | 0.0 | 5.1 | NA | NA | NA | NA | 1 |
|  |  | III | 10–20 | 3.9 | 3.3 | 4.7 | 4.5 | 0.697 | NA | NA | 4 |
|  | PM_250-50_ | I | 10–20 | 22 | 18 | 22 | 18 | NA | NA | NA | 2 |
|  |  | I | 20–200 | 14 | 6 | 14 | 8 | 0.816 | 0.788 | 0.788 | 9 |
|  |  | I | 200–300 | 24 | 5 | 24 | 5 | NA | NA | NA | 2 |
|  |  | II | 10–20 | 22 | 9 | 19 | 8 | 0.600 | 0.432 | NA | 5 |
|  |  | II | 20–200 | 15 | 0 | 15 | NA | NA | NA | NA | 1 |
|  |  | III | 10–20 | 1.5 | 2.2 | 5.3 | 8.7 | 0.033 | NA | NA | 4 |
|  | PM_500-250_ | I | 10–20 | 20 | 4 | 20 | 4 | NA | NA | NA | 2 |
|  |  | I | 20–200 | 19 | 7 | 17 | 10 | 0.234 | 0.060 | 0.130 | 9 |
|  |  | I | 200–300 | 10.6 | 13.6 | 10.6 | 13.0 | NA | NA | NA | 2 |
|  |  | II | 10–20 | 12 | 6 | 12 | 9 | 0.987 | 0.908 | NA | 5 |
|  |  | II | 20–200 | 22 | 0 | 22 | NA | NA | NA | NA | 1 |
|  |  | III | 10–20 | <0.1 | 0 | 8.0 | 16.0 | 0.001 | NA | NA | 4 |
|  | PM_50-10_ | I | 10–20 | 10.4 | 5.0 | 10.4 | 4.8 | NA | NA | NA | 2 |
|  |  | I | 20–200 | 10.9 | 9.0 | 19.7 | 20.6 | 0.013 | 0.005 | 0.010 | 9 |
|  |  | I | 200–300 | 28 | 9 | 28 | 9 | NA | NA | NA | 2 |
|  |  | II | 10–20 | 27 | 4 | 31 | 14 | 0.066 | 0.042 | NA | 5 |
|  |  | II | 20–200 | 18 | 0 | 18 | NA | NA | NA | NA | 1 |
|  |  | III | 10–20 | 3.7 | 1.0 | 4.1 | 1.9 | 0.375 | NA | NA | 4 |
|  | PM_5-1_ | I | 10–20 | 1.2 | 0.7 | 1.2 | 0.6 | NA | NA | NA | 2 |
|  |  | I | 20–200 | 3.5 | 4.4 | 4.0 | 3.5 | 0.174 | 0.322 | 0.196 | 9 |
|  |  | I | 200–300 | 17 | 10 | 17 | 9 | NA | NA | NA | 2 |
|  |  | II | 10–20 | 11 | 2 | 12 | 3 | 0.038 | 0.077 | NA | 5 |
|  |  | II | 20–200 | 6.8 | 0.0 | 6.8 | NA | NA | NA | NA | 1 |
|  |  | III | 10–20 | 48 | 10 | 45 | 17 | 0.417 | NA | NA | 4 |
| Major elements, g/100g | Al_2_O_3_ | I | 10–20 | 19 | 7 | 19 | 6 | NA | NA | NA | 2 |
|  |  | I | 20–200 | 23 | 3 | 23 | 2 | 0.704 | 0.668 | 0.558 | 9 |
|  |  | I | 200–300 | 23 | 1 | 23 | 1 | NA | NA | NA | 2 |
|  |  | II | 10–20 | 21 | 1 | 21 | 3 | 0.346 | 0.086 | NA | 5 |
|  |  | II | 20–200 | 20 | 0 | 20 | NA | NA | NA | NA | 1 |
|  |  | III | 10–20 | 54 | 5 | 53 | 5 | 0.833 | NA | NA | 4 |
|  | CaO | I | 10–20 | 4.5 | 0.7 | 4.5 | 0.7 | NA | NA | NA | 2 |
|  |  | I | 20–200 | 4.6 | 1.1 | 3.9 | 1.4 | 0.206 | 0.150 | 0.183 | 9 |
|  |  | I | 200–300 | 2.6 | 0.8 | 2.6 | 0.8 | NA | NA | NA | 2 |
|  |  | II | 10–20 | 2.7 | 0.3 | 2.7 | 0.7 | 0.937 | 0.803 | NA | 5 |
|  |  | II | 20–200 | 2.9 | 0.0 | 2.9 | NA | NA | NA | NA | 1 |
|  |  | III | 10–20 | 0.07 | 0.10 | 0.08 | 0.09 | 0.140 | NA | NA | 4 |
|  | Fe_2_O_3_ | I | 10–20 | 9.1 | 1.5 | 9.1 | 1.4 | NA | NA | NA | 2 |
|  |  | I | 20–200 | 10.4 | 0.6 | 10.7 | 1.9 | 0.253 | 0.050 | 0.117 | 9 |
|  |  | I | 200–300 | 9.6 | 0.4 | 9.6 | 0.4 | NA | NA | NA | 2 |
|  |  | II | 10–20 | 7.5 | 0.9 | 7.2 | 1.1 | 0.609 | 0.604 | NA | 5 |
|  |  | II | 20–200 | 7.1 | 0.0 | 7.1 | NA | NA | NA | NA | 1 |
|  |  | III | 10–20 | 8.7 | 1.0 | 13.1 | 9.7 | 0.008 | NA | NA | 4 |
|  | K_2_O | I | 10–20 | 1.1 | 0.0 | 1.1 | 0.0 | NA | NA | NA | 2 |
|  |  | I | 20–200 | 1.0 | 0.3 | 1.0 | 0.4 | 0.984 | 0.927 | 0.890 | 9 |
|  |  | I | 200–300 | 1.2 | 0.1 | 1.2 | 0.1 | NA | NA | NA | 2 |
|  |  | II | 10–20 | 1.7 | 0.1 | 1.9 | 0.6 | 0.207 | 0.066 | NA | 5 |
|  |  | II | 20–200 | 1.9 | 0.0 | 1.9 | NA | NA | NA | NA | 1 |
|  |  | III | 10–20 | 0.11 | 0.01 | 0.08 | 0.06 | 0.051 | NA | NA | 4 |
|  | MgO | I | 10–20 | 1.5 | 0.1 | 1.5 | 0.1 | NA | NA | NA | 2 |
|  |  | I | 20–200 | 1.5 | 0.1 | 1.5 | 0.1 | 0.946 | 0.888 | 0.892 | 9 |
|  |  | I | 200–300 | 1.6 | 0.1 | 1.6 | 0.1 | NA | NA | NA | 2 |
|  |  | II | 10–20 | 1.5 | 0.0 | 1.5 | 0.2 | 0.466 | 0.200 | NA | 5 |
|  |  | II | 20–200 | 1.6 | 0.0 | 1.6 | NA | NA | NA | NA | 1 |
|  |  | III | 10–20 | 0.35 | 0.08 | 0.28 | 0.19 | 0.163 | NA | NA | 4 |
|  | MnO | I | 10–20 | 2073 | 326 | 2073 | 311 | NA | NA | NA | 2 |
|  |  | I | 20–200 | 2427 | 329 | 2444 | 461 | 0.524 | 0.451 | 0.414 | 9 |
|  |  | I | 200–300 | 2187 | 14 | 2187 | 13 | NA | NA | NA | 2 |
|  |  | II | 10–20 | 1761 | 434 | 1659 | 385 | 0.660 | 0.703 | NA | 5 |
|  |  | II | 20–200 | 1624 | 0 | 1624 | NA | NA | NA | NA | 1 |
|  |  | III | 10–20 | 67 | 33 | 102 | 98 | 0.099 | NA | NA | 4 |
|  | P_2_O_5_ | I | 10–20 | 0.37 | 0.08 | 0.37 | 0.08 | NA | NA | NA | 2 |
|  |  | I | 20–200 | 0.22 | 0.12 | 0.23 | 0.10 | 0.682 | 0.680 | 0.741 | 9 |
|  |  | I | 200–300 | 0.20 | 0.10 | 0.20 | 0.10 | NA | NA | NA | 2 |
|  |  | II | 10–20 | 0.14 | 0.03 | 0.16 | 0.05 | 0.207 | 0.367 | NA | 5 |
|  |  | II | 20–200 | 0.12 | 0.00 | 0.12 | NA | NA | NA | NA | 1 |
|  |  | III | 10–20 | 0.18 | 0.04 | 0.14 | 0.09 | 0.086 | NA | NA | 4 |
|  | SiO_2_ | I | 10–20 | 57 | 1 | 57 | 1 | NA | NA | NA | 2 |
|  |  | I | 20–200 | 57 | 3 | 58 | 4 | 0.172 | 0.408 | 0.166 | 9 |
|  |  | I | 200–300 | 59 | 1 | 59 | 1 | NA | NA | NA | 2 |
|  |  | II | 10–20 | 65 | 1 | 66 | 2 | 0.171 | 0.565 | NA | 5 |
|  |  | II | 20–200 | 66 | 0 | 66 | NA | NA | NA | NA | 1 |
|  |  | III | 10–20 | 35 | 7 | 31 | 14 | 0.282 | NA | NA | 4 |
|  | TiO_2_ | I | 10–20 | 1.2 | 0.3 | 1.2 | 0.3 | NA | NA | NA | 2 |
|  |  | I | 20–200 | 1.6 | 0.1 | 1.6 | 0.2 | 0.372 | 0.513 | 0.232 | 9 |
|  |  | I | 200–300 | 1.4 | 0.1 | 1.4 | 0.1 | NA | NA | NA | 2 |
|  |  | II | 10–20 | 1.2 | 0.1 | 1.2 | 0.1 | 0.324 | 0.432 | NA | 5 |
|  |  | II | 20–200 | 1.1 | 0.0 | 1.1 | NA | NA | NA | NA | 1 |
|  |  | III | 10–20 | 2.5 | 0.6 | 2.5 | 0.5 | 0.808 | NA | NA | 4 |
| Micro elements, mg/kg | As | I | 10–20 | 5.0 | 3.0 | 5.0 | 2.8 | NA | NA | NA | 2 |
|  |  | I | 20–200 | 6.0 | 3.0 | 6.9 | 3.6 | 0.131 | 0.071 | 0.125 | 9 |
|  |  | I | 200–300 | 10.0 | 1.5 | 10.0 | 1.4 | NA | NA | NA | 2 |
|  |  | II | 10–20 | 9.0 | 1.5 | 9.2 | 2.6 | 0.955 | 0.864 | NA | 5 |
|  |  | II | 20–200 | 8.0 | 0.0 | 8.0 | NA | NA | NA | NA | 1 |
|  |  | III | 10–20 | 48 | 15 | 48 | 16 | 0.870 | NA | NA | 4 |
|  | Co | I | 10–20 | 44 | 27 | 44 | 25 | NA | NA | NA | 2 |
|  |  | I | 20–200 | 73 | 6 | 75 | 25 | 0.070 | 0.067 | 0.022 | 9 |
|  |  | I | 200–300 | 51 | 2 | 51 | 2 | NA | NA | NA | 2 |
|  |  | II | 10–20 | 38 | 10 | 35 | 10 | 0.851 | 0.679 | NA | 5 |
|  |  | II | 20–200 | 36 | 0 | 36 | NA | NA | NA | NA | 1 |
|  |  | III | 10–20 | 110 | 27 | 135 | 73 | 0.133 | NA | NA | 4 |
|  | Cr | I | 10–20 | 46 | 1 | 46 | 1 | NA | NA | NA | 2 |
|  |  | I | 20–200 | 44 | 7 | 42 | 7 | 0.798 | 0.761 | 0.718 | 9 |
|  |  | I | 200–300 | 48 | 2 | 48 | 2 | NA | NA | NA | 2 |
|  |  | II | 10–20 | 43 | 6 | 43 | 6 | 0.611 | 0.509 | NA | 5 |
|  |  | II | 20–200 | 46 | 0 | 46 | NA | NA | NA | NA | 1 |
|  |  | III | 10–20 | 63 | 13 | 69 | 23 | 0.367 | NA | NA | 4 |
|  | Cu | I | 10–20 | 29 | 11 | 29 | 11 | NA | NA | NA | 2 |
|  |  | I | 20–200 | 30 | 4 | 32 | 4 | 0.310 | 0.188 | 0.319 | 9 |
|  |  | I | 200–300 | 30 | 4 | 30 | 4 | NA | NA | NA | 2 |
|  |  | II | 10–20 | 31 | 4 | 32 | 10 | 0.373 | 0.122 | NA | 5 |
|  |  | II | 20–200 | 34 | 0 | 34 | NA | NA | NA | NA | 1 |
|  |  | III | 10–20 | 160 | 118 | 169 | 103 | 0.117 | NA | NA | 4 |
|  | Ni | I | 10–20 | 36 | 1 | 36 | 1 | NA | NA | NA | 2 |
|  |  | I | 20–200 | 32 | 4 | 32 | 3 | 0.373 | 0.533 | 0.449 | 9 |
|  |  | I | 200–300 | 32 | 1 | 32 | 1 | NA | NA | NA | 2 |
|  |  | II | 10–20 | 33 | 3 | 33 | 4 | 0.980 | 0.928 | NA | 5 |
|  |  | II | 20–200 | 36 | 0 | 36 | NA | NA | NA | NA | 1 |
|  |  | III | 10–20 | 71 | 14 | 73 | 16 | 0.857 | NA | NA | 4 |
|  | Pb | I | 10–20 | 12 | 10 | 12 | 10 | NA | NA | NA | 2 |
|  |  | I | 20–200 | 18 | 18 | 29 | 27 | 0.047 | 0.144 | 0.057 | 9 |
|  |  | I | 200–300 | 52 | 8 | 52 | 8 | NA | NA | NA | 2 |
|  |  | II | 10–20 | 42 | 12 | 43 | 17 | 0.923 | 0.647 | NA | 5 |
|  |  | II | 20–200 | 38 | 0 | 38 | NA | NA | NA | NA | 1 |
|  |  | III | 10–20 | 206 | 69 | 207 | 74 | 0.810 | NA | NA | 4 |
|  | Sr | I | 10–20 | 244 | 1 | 244 | 1 | NA | NA | NA | 2 |
|  |  | I | 20–200 | 241 | 28 | 230 | 50 | 0.292 | 0.288 | 0.170 | 9 |
|  |  | I | 200–300 | 319 | 4 | 319 | 4 | NA | NA | NA | 2 |
|  |  | II | 10–20 | 296 | 67 | 315 | 51 | 0.260 | 0.387 | NA | 5 |
|  |  | II | 20–200 | 295 | 0 | 295 | NA | NA | NA | NA | 1 |
|  |  | III | 10–20 | 284 | 28 | 297 | 54 | 0.330 | NA | NA | 4 |
|  | V | I | 10–20 | 166 | 42 | 166 | 40 | NA | NA | NA | 2 |
|  |  | I | 20–200 | 195 | 12 | 189 | 27 | 0.011 | 0.099 | 0.016 | 9 |
|  |  | I | 200–300 | 172 | 11 | 172 | 11 | NA | NA | NA | 2 |
|  |  | II | 10–20 | 148 | 39 | 150 | 28 | 0.695 | 0.735 | NA | 5 |
|  |  | II | 20–200 | 135 | 0 | 135 | NA | NA | NA | NA | 1 |
|  |  | III | 10–20 | 274 | 105 | 283 | 94 | 0.237 | NA | NA | 4 |
|  | Zn | I | 10–20 | 139 | 8 | 139 | 8 | NA | NA | NA | 2 |
|  |  | I | 20–200 | 126 | 9 | 125 | 13 | 0.093 | 0.086 | 0.111 | 9 |
|  |  | I | 200–300 | 131 | 5 | 131 | 5 | NA | NA | NA | 2 |
|  |  | II | 10–20 | 114 | 7 | 122 | 17 | 0.135 | 0.201 | NA | 5 |
|  |  | II | 20–200 | 106 | 0 | 106 | NA | NA | NA | NA | 1 |
|  |  | III | 10–20 | 400 | 56 | 354 | 130 | 0.158 | NA | NA | 4 |
| Mineral composition, g/100g | Albite | I | 10–20 | 8.3 | 0.0 | 8.3 | NA | NA | NA | NA | 1 |
|  |  | I | 20–200 | 8.5 | 4.7 | 7.3 | 3.9 | 0.452 | 0.219 | 0.343 | 10 |
|  |  | I | 200–300 | 1.6 | 0.1 | 2.3 | 1.4 | NA | NA | NA | 3 |
|  |  | II | 10–20 | 9.3 | 4.0 | 10.2 | 5.9 | 0.203 | 0.257 | NA | 7 |
|  |  | II | 20–200 | 4.8 | 0.0 | 4.8 | NA | NA | NA | NA | 1 |
|  |  | III | 10–20 | 0 | 0 | <0.1 | 0 | NA | NA | NA | 7 |
|  | Anatase | I | 10–20 | 0.3 | 0.0 | 0.3 | NA | NA | NA | NA | 1 |
|  |  | I | 20–200 | 0.3 | 0.4 | 0.6 | 0.8 | 0.011 | 0.050 | 0.022 | 10 |
|  |  | I | 200–300 | 0.9 | 0.7 | 0.9 | 0.6 | NA | NA | NA | 3 |
|  |  | II | 10–20 | 0.6 | 0.6 | 1.0 | 0.9 | 0.017 | 0.093 | NA | 7 |
|  |  | II | 20–200 | 0.7 | 0.0 | 0.7 | NA | NA | NA | NA | 1 |
|  |  | III | 10–20 | 16 | 5 | 13 | 6 | 0.088 | 0.222 | NA | 7 |
|  | Anorthite | I | 10–20 | 41 | 0 | 41 | NA | NA | NA | NA | 1 |
|  |  | I | 20–200 | 44 | 22 | 34 | 25 | 0.022 | 0.164 | 0.025 | 10 |
|  |  | I | 200–300 | 4.1 | 3.7 | 6.2 | 5.9 | NA | NA | NA | 3 |
|  |  | II | 10–20 | 17 | 3 | 15 | 9 | 0.606 | 0.373 | NA | 7 |
|  |  | II | 20–200 | 20 | 0 | 20 | NA | NA | NA | NA | 1 |
|  |  | III | 10–20 | <0.1 | 0 | 1.1 | 2.8 | 0.000 | 0.000 | NA | 7 |
|  | Clinoptilolite | I | 10–20 | 8.2 | 0.0 | 8.2 | NA | NA | NA | NA | 1 |
|  |  | I | 20–200 | 9.0 | 3.9 | 8.7 | 3.3 | 0.341 | 0.283 | 0.390 | 10 |
|  |  | I | 200–300 | 9.5 | 0.3 | 7.7 | 3.3 | NA | NA | NA | 3 |
|  |  | II | 10–20 | 9.2 | 1.0 | 8.7 | 3.7 | 0.263 | 0.096 | NA | 7 |
|  |  | II | 20–200 | 9.5 | 0.0 | 9.5 | NA | NA | NA | NA | 1 |
|  |  | III | 10–20 | 15 | 7 | 14 | 6 | 0.576 | 0.498 | NA | 7 |
|  | Cristobalite | I | 10–20 | 6.4 | 0.0 | 6.4 | NA | NA | NA | NA | 1 |
|  |  | I | 20–200 | <0.1 | 0 | 0.5 | 0.8 | 0.001 | 0.002 | 0.000 | 10 |
|  |  | I | 200–300 | 1.7 | 0.6 | 3.8 | 4.0 | NA | NA | NA | 3 |
|  |  | II | 10–20 | 0.4 | 0.6 | 1.2 | 2.2 | 0.000 | 0.003 | NA | 7 |
|  |  | II | 20–200 | 1.3 | 0.0 | 1.3 | NA | NA | NA | NA | 1 |
|  |  | III | 10–20 | 0 | 0 | 0 | 0 | NA | NA | NA | 7 |
|  | Goethite | I | 10–20 | 1.8 | 0.0 | 1.8 | NA | NA | NA | NA | 1 |
|  |  | I | 20–200 | 0 | 0 | 0.2 | 0.4 | 0.000 | 0.000 | 0.000 | 10 |
|  |  | I | 200–300 | 1.0 | 0.4 | 1.0 | 0.4 | NA | NA | NA | 3 |
|  |  | II | 10–20 | 0.3 | 0.4 | 0.4 | 0.4 | 0.056 | 0.166 | NA | 7 |
|  |  | II | 20–200 | <0.1 | 0 | <0.1 | NA | NA | NA | NA | 1 |
|  |  | III | 10–20 | <0.1 | 0 | <0.1 | 0 | NA | NA | NA | 7 |
|  | Hematite | I | 10–20 | <0.1 | 0 | <0.1 | NA | NA | NA | NA | 1 |
|  |  | I | 20–200 | <0.1 | 0 | 0.2 | 0.4 | 0.000 | 0.000 | 0.000 | 10 |
|  |  | I | 200–300 | 0.2 | 0.3 | 0.2 | 0.3 | NA | NA | NA | 3 |
|  |  | II | 10–20 | 0.2 | 0.3 | 0.4 | 0.5 | 0.019 | 0.012 | NA | 7 |
|  |  | II | 20–200 | 0.2 | 0.0 | 0.2 | NA | NA | NA | NA | 1 |
|  |  | III | 10–20 | <0.1 | 0 | <0.1 | 0 | NA | NA | NA | 7 |
|  | Heulandite | I | 10–20 | <0.1 | 0 | <0.1 | NA | NA | NA | NA | 1 |
|  |  | I | 20–200 | <0.1 | 0 | 0.7 | 1.5 | 0.000 | 0.000 | 0.000 | 10 |
|  |  | I | 200–300 | 6.0 | 1.8 | 5.2 | 2.6 | NA | NA | NA | 3 |
|  |  | II | 10–20 | <0.1 | 0 | 0.2 | 0.5 | 0.000 | 0.000 | NA | 7 |
|  |  | II | 20–200 | <0.1 | 0 | <0.1 | NA | NA | NA | NA | 1 |
|  |  | III | 10–20 | 1.2 | 1.8 | 2.8 | 3.3 | 0.057 | 0.193 | NA | 7 |
|  | Kaolinite | I | 10–20 | 6.2 | 0.0 | 6.2 | NA | NA | NA | NA | 1 |
|  |  | I | 20–200 | 6.0 | 2.8 | 6.2 | 2.0 | 0.444 | 0.551 | 0.395 | 10 |
|  |  | I | 200–300 | 6.6 | 0.9 | 7.3 | 1.8 | NA | NA | NA | 3 |
|  |  | II | 10–20 | 5.1 | 1.8 | 5.6 | 2.5 | 0.826 | 0.812 | NA | 7 |
|  |  | II | 20–200 | 3.0 | 0.0 | 3.0 | NA | NA | NA | NA | 1 |
|  |  | III | 10–20 | 56 | 5 | 57 | 8 | 0.820 | 0.690 | NA | 7 |
|  | Microcline | I | 10–20 | 8.8 | 0.0 | 8.8 | NA | NA | NA | NA | 1 |
|  |  | I | 20–200 | 10.9 | 3.0 | 10.0 | 3.7 | 0.410 | 0.371 | 0.378 | 10 |
|  |  | I | 200–300 | 6.8 | 0.9 | 6.7 | 0.8 | NA | NA | NA | 3 |
|  |  | II | 10–20 | 10.2 | 3.6 | 12.9 | 4.8 | 0.083 | 0.093 | NA | 7 |
|  |  | II | 20–200 | 13 | 0 | 13 | NA | NA | NA | NA | 1 |
|  |  | III | 10–20 | 2.5 | 0.6 | 2.4 | 1.3 | 0.309 | 0.123 | NA | 7 |
|  | Pyrite | I | 10–20 | <0.1 | 0 | <0.1 | NA | NA | NA | NA | 1 |
|  |  | I | 20–200 | 0.4 | 0.4 | 0.4 | 0.3 | 0.168 | 0.306 | 0.210 | 10 |
|  |  | I | 200–300 | 0 | 0 | 0.07 | 0.12 | NA | NA | NA | 3 |
|  |  | II | 10–20 | 0.2 | 0.0 | 0.2 | 0.1 | 0.183 | 0.019 | NA | 7 |
|  |  | II | 20–200 | 0.1 | 0.0 | 0.1 | NA | NA | NA | NA | 1 |
|  |  | III | 10–20 | 0.3 | 0.1 | 0.4 | 0.4 | 0.017 | 0.007 | NA | 7 |
|  | Quartz | I | 10–20 | 5.8 | 0.0 | 5.8 | NA | NA | NA | NA | 1 |
|  |  | I | 20–200 | 2.2 | 1.9 | 4.0 | 4.4 | 0.002 | 0.056 | 0.004 | 10 |
|  |  | I | 200–300 | 6.2 | 8.3 | 6.3 | 5.7 | NA | NA | NA | 3 |
|  |  | II | 10–20 | 15 | 3 | 12 | 5 | 0.116 | 0.114 | NA | 7 |
|  |  | II | 20–200 | 21 | 0 | 21 | NA | NA | NA | NA | 1 |
|  |  | III | 10–20 | 0.2 | 0.3 | 2.6 | 3.9 | 0.011 | 0.058 | NA | 7 |
|  | Smectite | I | 10–20 | 13 | 0 | 13 | NA | NA | NA | NA | 1 |
|  |  | I | 20–200 | 15 | 20 | 27 | 30 | 0.014 | 0.080 | 0.019 | 10 |
|  |  | I | 200–300 | 53 | 3 | 52 | 3 | NA | NA | NA | 3 |
|  |  | II | 10–20 | 30 | 7 | 32 | 11 | 0.090 | 0.174 | NA | 7 |
|  |  | II | 20–200 | 26 | 0 | 26 | NA | NA | NA | NA | 1 |
|  |  | III | 10–20 | <0.1 | 0 | 5.8 | 7.2 | 0.005 | 0.007 | NA | 7 |
| Naex, µg/kg |  | I | 10–20 | 121 | 0 | 121 | NA | NA | NA | NA | 1 |
|  |  | II | 10–20 | 1568 | 2067 | 1568 | 1971 | NA | NA | NA | 2 |
|  |  | III | 10–20 | 74 | 12 | 78 | 15 | NA | NA | NA | 3 |
|  |  | I | 10–20 | 1.0 | 0.0 | 1.0 | NA | NA | NA | NA | 1 |
|  |  | II | 10–20 | 7.0 | 8.9 | 7.0 | 8.5 | NA | NA | NA | 2 |
|  |  | III | 10–20 | <0.1 | 0 | 0 | 0 | NA | NA | NA | 3 |
| Short-range-order Al, Fe and Si compounds, g/kg | Al_ox_ | I | 10–20 | 9.2 | 0.0 | 9.2 | NA | NA | NA | NA | 1 |
|  |  | I | 20–200 | 15 | 7 | 15 | 7 | NA | NA | NA | 2 |
|  |  | II | 10–20 | 2.1 | 0.4 | 2.1 | 0.4 | NA | NA | NA | 2 |
|  |  | III | 10–20 | 1.3 | 0.0 | 1.4 | 0.2 | NA | NA | NA | 3 |
|  | Fe_ox_ | I | 10–20 | 12 | 0 | 12 | NA | NA | NA | NA | 1 |
|  |  | I | 20–200 | 18 | 6 | 18 | 6 | NA | NA | NA | 2 |
|  |  | II | 10–20 | 3.0 | 0.4 | 3.0 | 0.4 | NA | NA | NA | 2 |
|  |  | III | 10–20 | 1.0 | 0.1 | 1.2 | 0.4 | NA | NA | NA | 3 |
|  | Si_ox_ | I | 10–20 | 2.2 | 0.0 | 2.2 | NA | NA | NA | NA | 1 |
|  |  | I | 20–200 | 7.4 | 1.5 | 7.4 | 1.4 | NA | NA | NA | 2 |
|  |  | II | 10–20 | 1.3 | 0.2 | 1.3 | 0.2 | NA | NA | NA | 2 |
|  |  | III | 10–20 | 0.34 | 0.21 | 0.42 | 0.26 | NA | NA | NA | 3 |
| Cation composition, mmol (+)/100g | Ca^2+^ | I | 10–20 | 23 | 25 | 23 | 24 | NA | NA | NA | 2 |
|  |  | I | 20–200 | 10.0 | 1.1 | 10.9 | 2.6 | 0.339 | 0.052 | 0.187 | 9 |
|  |  | I | 200–300 | 10.5 | 6.0 | 10.5 | 5.7 | NA | NA | NA | 2 |
|  |  | II | 10–20 | 33 | 11 | 28 | 13 | 0.449 | 0.482 | NA | 5 |
|  |  | II | 20–200 | 15 | 0 | 15 | NA | NA | NA | NA | 1 |
|  |  | III | 10–20 | 16 | 4 | 16 | 4 | 0.782 | NA | NA | 4 |
|  | K^+^ | I | 10–20 | 3.2 | 2.5 | 3.2 | 2.4 | NA | NA | NA | 2 |
|  |  | I | 20–200 | 0.56 | 0.28 | 0.67 | 0.34 | 0.284 | 0.547 | 0.311 | 9 |
|  |  | I | 200–300 | 0.67 | 0.07 | 0.67 | 0.07 | NA | NA | NA | 2 |
|  |  | II | 10–20 | 0.76 | 0.09 | 0.81 | 0.24 | 0.449 | 0.235 | NA | 5 |
|  |  | II | 20–200 | 0.80 | 0.00 | 0.80 | NA | NA | NA | NA | 1 |
|  |  | III | 10–20 | 0.47 | 0.06 | 0.65 | 0.42 | 0.014 | NA | NA | 4 |
|  | Mg^2+^ | I | 10–20 | 12 | 12 | 12 | 12 | NA | NA | NA | 2 |
|  |  | I | 20–200 | 4.2 | 1.0 | 4.1 | 0.8 | 0.895 | 0.423 | 0.822 | 9 |
|  |  | I | 200–300 | 3.7 | 1.7 | 3.7 | 1.7 | NA | NA | NA | 2 |
|  |  | II | 10–20 | 4.5 | 0.9 | 4.5 | 1.1 | 0.948 | 0.954 | NA | 5 |
|  |  | II | 20–200 | 4.3 | 0.0 | 4.3 | NA | NA | NA | NA | 1 |
|  |  | III | 10–20 | 4.9 | 0.9 | 4.9 | 0.7 | 0.041 | NA | NA | 4 |
|  | Na^+^ | I | 10–20 | 6.0 | 3.4 | 6.0 | 3.2 | NA | NA | NA | 2 |
|  |  | I | 20–200 | 4.8 | 0.7 | 4.4 | 0.8 | 0.646 | 0.217 | 0.540 | 9 |
|  |  | I | 200–300 | 4.4 | 1.4 | 4.4 | 1.4 | NA | NA | NA | 2 |
|  |  | II | 10–20 | 5.3 | 2.5 | 5.2 | 1.6 | 0.444 | 0.470 | NA | 5 |
|  |  | II | 20–200 | 6.4 | 0.0 | 6.4 | NA | NA | NA | NA | 1 |
|  |  | III | 10–20 | 3.1 | 1.9 | 3.1 | 1.6 | 0.827 | NA | NA | 4 |
|  | NH_4_^+^ | I | 10–20 | 0.03 | 0 | 0.03 | 0 | NA | NA | NA | 2 |
|  |  | I | 20–200 | 0.03 | 0 | 0.03 | 0 | NA | NA | NA | 9 |
|  |  | I | 200–300 | 0.03 | 0 | 0.03 | 0 | NA | NA | NA | 2 |
|  |  | II | 10–20 | 0.03 | 0 | 0.03 | 0 | NA | NA | NA | 5 |
|  |  | II | 20–200 | 0.03 | 0 | 0.03 | 0 | NA | NA | NA | 1 |
|  |  | III | 10–20 | 0.52 | 0.72 | 0.55 | 0.60 | 0.078 | NA | NA | 4 |
| Anion composition, mmol (–)/100g | Cl^–^ | I | 10–20 | 1.3 | 0.9 | 1.3 | 0.8 | NA | NA | NA | 2 |
|  |  | I | 20–200 | 1.2 | 0.5 | 1.4 | 0.6 | 0.537 | 0.174 | 0.394 | 9 |
|  |  | I | 200–300 | 1.4 | 1.0 | 1.4 | 1.0 | NA | NA | NA | 2 |
|  |  | II | 10–20 | 0.82 | 0.15 | 0.98 | 0.34 | 0.058 | 0.146 | NA | 5 |
|  |  | II | 20–200 | 1.6 | 0.0 | 1.6 | NA | NA | NA | NA | 1 |
|  |  | III | 10–20 | 1.3 | 0.7 | 1.3 | 0.6 | 0.156 | NA | NA | 4 |
|  | F^–^ | I | 10–20 | 0.42 | 0.41 | 0.42 | 0.39 | NA | NA | NA | 2 |
|  |  | I | 20–200 | 0.96 | 0.43 | 0.92 | 0.36 | 0.364 | 0.344 | 0.339 | 9 |
|  |  | I | 200–300 | 1.4 | 0.8 | 1.4 | 0.7 | NA | NA | NA | 2 |
|  |  | II | 10–20 | 2.5 | 0.9 | 2.5 | 1.0 | 0.938 | 0.966 | NA | 5 |
|  |  | II | 20–200 | 1.6 | 0.0 | 1.6 | NA | NA | NA | NA | 1 |
|  |  | III | 10–20 | 1.4 | 0.5 | 1.4 | 0.4 | 0.455 | NA | NA | 4 |
|  | HCO_3_^–^ | I | 10–20 | 0.21 | 0.01 | 0.21 | 0.01 | NA | NA | NA | 2 |
|  |  | I | 20–200 | 0.24 | 0.06 | 0.20 | 0.07 | 0.189 | 0.021 | 0.095 | 9 |
|  |  | I | 200–300 | 0.10 | 0.00 | 0.10 | NA | NA | NA | NA | 1 |
|  |  | II | 10–20 | 0.63 | 0.56 | 0.74 | 0.61 | 0.460 | NA | NA | 4 |
|  |  | II | 20–200 | 0.16 | 0.00 | 0.16 | NA | NA | NA | NA | 1 |
|  |  | III | 10–20 | <0.1 | 0 | 0.37 | 0.64 | NA | NA | NA | 3 |
|  | HPO_4_^2–^ | I | 10–20 | <0.001 | 0 | <0.001 | 0 | NA | NA | NA | 2 |
|  |  | I | 20–200 | <0.001 | 0 | 0.001 | 0.003 | 0.000 | 0.000 | 0.000 | 9 |
|  |  | I | 200–300 | 0.003 | 0.004 | 0.003 | 0.004 | NA | NA | NA | 2 |
|  |  | II | 10–20 | <0.001 | 0 | 0.002 | 0.005 | 0.000 | 0.001 | NA | 5 |
|  |  | II | 20–200 | <0.001 | 0 | <0.001 | NA | NA | NA | NA | 1 |
|  |  | III | 10–20 | <0.001 | 0 | <0.001 | 0 | NA | NA | NA | 4 |
|  | NO_3_^–^ | I | 10–20 | 0.03 | 0.01 | 0.03 | 0.01 | NA | NA | NA | 2 |
|  |  | I | 20–200 | 0.15 | 0.11 | 0.17 | 0.14 | 0.203 | 0.605 | 0.281 | 9 |
|  |  | I | 200–300 | 0.08 | 0.06 | 0.08 | 0.06 | NA | NA | NA | 2 |
|  |  | II | 10–20 | 0.18 | 0.10 | 0.19 | 0.08 | 0.792 | 0.912 | NA | 5 |
|  |  | II | 20–200 | 0.17 | 0.00 | 0.17 | NA | NA | NA | NA | 1 |
|  |  | III | 10–20 | 0.23 | 0.21 | 0.27 | 0.25 | 0.723 | NA | NA | 4 |
|  | SO_4_^2–^ | I | 10–20 | 0.57 | 0.47 | 0.57 | 0.45 | NA | NA | NA | 2 |
|  |  | I | 20–200 | 0.18 | 0.12 | 0.23 | 0.14 | 0.309 | 0.231 | 0.283 | 9 |
|  |  | I | 200–300 | 0.18 | 0.16 | 0.18 | 0.15 | NA | NA | NA | 2 |
|  |  | II | 10–20 | 0.31 | 0.04 | 0.30 | 0.04 | 0.978 | 0.903 | NA | 5 |
|  |  | II | 20–200 | 0.47 | 0.00 | 0.47 | NA | NA | NA | NA | 1 |
|  |  | III | 10–20 | 2.9 | 0.6 | 3.3 | 1.1 | 0.165 | NA | NA | 4 |

Me – median, MAD – median absolute deviation, M – mean, SD – standard deviation. P-values <0.05 are marked in red.

## *Table S 5. Results of the Mann-Whitney U-test for soil samples collected from different depths*

| Properties | | I | | | | | | II | | | III |
| --- | --- | --- | --- | --- | --- | --- | --- | --- | --- | --- | --- |
|  |  | 1 & 2 | 1 & 3 | 1 & 4 | 2 & 3 | 2 & 4 | 3 & 4 | 1&2 | 1&3 | 2&3 | 1 & 2 |
| Physico–chemical properties | EC | 1.0 | 0.017 | 1.0 | 1.0 | 1.0 | 1.0 | 0.041 | 0.400 | 0.667 | 0.076 |
|  | LOI | 1.0 | 0.100 | 1.0 | 1.0 | 1.0 | 1.0 | 0.059 | 0.800 | 0.800 | 0.388 |
|  | Eh | 0.800 | 0.017 | 0.533 | 0.364 | 0.800 | 0.800 | 0.019 | 0.444 | 0.444 | 0.644 |
|  | pH | 1.0 | 0.953 | 0.953 | 1.0 | 1.0 | 1.0 | 0.681 | 0.967 | 0.967 | 0.541 |
|  | SOC | 1.0 | 0.067 | 0.533 | 0.364 | 1.0 | 1.0 | 0.019 | 1.0 | 0.667 | 0.028 |
| Grain–size fractions | PM_<1_ | 1.0 | 0.643 | 0.455 | 1.0 | 1.0 | 0.455 | 0.015 | 0.726 | 0.667 | 0.006 |
|  | PM_10–5_ | 1.0 | 1.0 | 0.800 | 1.0 | 1.0 | 0.800 | 0.180 | 0.800 | 0.667 | 0.434 |
|  | PM_1000–500_ | 1.0 | 1.0 | 0.800 | 1.0 | 1.0 | 0.800 | 0.215 | 1.0 | 0.667 | 0.015 |
|  | PM_250–50_ | 1.0 | 0.453 | 1.0 | 1.0 | 1.0 | 0.727 | 1.0 | 1.0 | 1.0 | 0.153 |
|  | PM_5–1_ | 1.0 | 0.756 | 0.501 | 1.0 | 1.0 | 0.218 | 0.010 | 0.800 | 0.470 | 0.014 |
|  | PM_50–10_ | 1.0 | 1.0 | 0.800 | 1.0 | 1.0 | 1.0 | 0.569 | 0.800 | 0.667 | 0.005 |
|  | PM_500–250_ | 0.667 | 0.222 | 0.667 | 1.0 | 1.0 | 1.0 | 1.0 | 1.0 | 1.0 | 0.174 |
| Major elements | Al_2_O_3_ | 1.0 | 0.039 | 0.667 | 1.0 | 1.0 | 1.0 | 0.471 | 1.0 | 1.0 | 0.008 |
|  | Fe_2_O_3_ | 1.0 | 0.150 | 0.623 | 0.623 | 1.0 | 0.623 | 0.857 | 0.857 | 1.0 | 0.241 |
|  | K_2_O | 1.0 | 1.0 | 1.0 | 1.0 | 1.0 | 1.0 | 1.0 | 1.0 | 1.0 | 0.003 |
|  | MgO | 1.0 | 1.0 | 1.0 | 1.0 | 1.0 | 1.0 | 0.801 | 0.801 | 0.801 | 0.004 |
|  | MnO | 1.0 | 0.117 | 0.667 | 1.0 | 1.0 | 0.667 | 1.0 | 0.667 | 1.0 | 0.005 |
|  | P_2_O_5_ | 0.667 | 0.017 | 0.667 | 0.667 | 0.667 | 0.667 | 0.046 | 0.343 | 0.534 | 0.050 |
|  | SiO_2_ | 0.875 | 0.601 | 0.601 | 1.0 | 1.0 | 1.0 | 0.095 | 0.332 | 0.763 | 0.017 |
|  | TiO_2_ | 1.0 | 0.149 | 0.501 | 0.622 | 1.0 | 0.622 | 0.569 | 1.0 | 1.0 | 0.007 |
| Micro elements | As | 1.0 | 1.0 | 0.953 | 1.0 | 1.0 | 1.0 | 0.675 | 1.0 | 1.0 | 0.009 |
|  | CaO | 1.0 | 0.206 | 0.276 | 1.0 | 1.0 | 1.0 | 0.179 | 0.578 | 1.0 | 0.052 |
|  | Co | 1.0 | 0.149 | 0.495 | 0.379 | 1.0 | 0.495 | 0.636 | 1.0 | 1.0 | 0.081 |
|  | Cr | 1.0 | 1.0 | 1.0 | 1.0 | 1.0 | 1.0 | 1.0 | 0.465 | 1.0 | 0.087 |
|  | Cu | 1.0 | 0.120 | 1.0 | 1.0 | 1.0 | 1.0 | 1.0 | 1.0 | 1.0 | 0.017 |
|  | Ni | 0.501 | 0.050 | 0.501 | 0.501 | 0.667 | 0.721 | 1.0 | 1.0 | 1.0 | 0.017 |
|  | Pb | 1.0 | 1.0 | 0.800 | 1.0 | 1.0 | 1.0 | 0.170 | 0.889 | 0.889 | 0.009 |
|  | Sr | 1.0 | 1.0 | 0.667 | 1.0 | 1.0 | 0.268 | 0.811 | 1.0 | 1.0 | 0.394 |
|  | V | 1.0 | 0.117 | 0.667 | 1.0 | 1.0 | 0.667 | 1.0 | 1.0 | 1.0 | 0.110 |
|  | Zn | 1.0 | 0.302 | 1.0 | 1.0 | 1.0 | 1.0 | 0.196 | 0.444 | 0.444 | 0.025 |
| Mineral composition | Albite | 1.0 | 1.0 | 0.383 | 1.0 | 1.0 | 0.294 | 0.135 | 1.0 | 1.0 | 0.018 |
|  | Anatase | 1.0 | 1.0 | 0.985 | 1.0 | 1.0 | 1.0 | 1.0 | 1.0 | 1.0 | 0.008 |
|  | Anorthite | 1.0 | 1.0 | 0.459 | 1.0 | 1.0 | 1.0 | 1.0 | 1.0 | 1.0 | 0.103 |
|  | Clinoptilolite | 1.0 | 1.0 | 1.0 | 1.0 | 1.0 | 1.0 | 1.0 | 1.0 | 1.0 | 0.027 |
|  | Cristobalite | 1.0 | 1.0 | 1.0 | 0.604 | 1.0 | 0.245 | 0.557 | 0.874 | 0.760 | 0.029 |
|  | Goethite | 0.745 | 1.0 | 0.318 | 0.318 | 1.0 | 0.157 | 0.748 | 0.965 | 0.965 | 0.403 |
|  | Hematite | na | 1.0 | 0.984 | 1.0 | 1.0 | 1.0 | 0.368 | 0.708 | 1.0 | 0.067 |
|  | Heulandite | 1.0 | 1.0 | 0.920 | 1.0 | 1.0 | 0.079 | 1.0 | 1.0 | 1.0 | 0.032 |
|  | Kaolinite | 1.0 | 1.0 | 1.0 | 1.0 | 1.0 | 1.0 | 1.0 | 0.802 | 1.0 | 0.458 |
|  | Microcline | 1.0 | 1.0 | 0.459 | 1.0 | 1.0 | 1.0 | 1.0 | 1.0 | 1.0 | 0.053 |
|  | Pyrite | 1.0 | 1.0 | 0.433 | 1.0 | 1.0 | 0.945 | 0.684 | 0.826 | 0.826 | 0.624 |
|  | Quartz | 1.0 | 1.0 | 1.0 | 1.0 | 1.0 | 1.0 | 0.750 | 0.750 | 0.750 | 0.018 |
|  | Smectite | 1.0 | 1.0 | 0.459 | 1.0 | 1.0 | 1.0 | 1.0 | 1.0 | 1.0 | 0.297 |
| Cation-anion composition | Ca^2+^ | 1.0 | 0.218 | 1.0 | 1.0 | 1.0 | 1.0 | 1.0 | 1.0 | 1.0 | 0.002 |
|  | Cl^–^ | 1.0 | 1.0 | 1.0 | 1.0 | 1.0 | 1.0 | 0.294 | 0.727 | 0.470 | 0.696 |
|  | F^–^ | 1.0 | 1.0 | 1.0 | 1.0 | 1.0 | 1.0 | 1.0 | 1.0 | 1.0 | 0.515 |
|  | HCO_3_^–^ | 1.0 | 0.358 | 1.0 | 1.0 | 1.0 | 1.0 | 0.938 | 0.487 | 0.800 | 0.788 |
|  | HPO_4_^2–^ | na | 1.0 | 1.0 | 1.0 | 1.0 | 1.0 | 1.0 | 1.0 | 1.0 | 0.481 |
|  | K^+^ | 1.0 | 0.218 | 1.0 | 0.218 | 1.0 | 1.0 | 0.014 | 0.727 | 1.0 | 0.081 |
|  | Mg^2+^ | 1.0 | 0.218 | 1.0 | 1.0 | 1.0 | 1.0 | 0.084 | 0.727 | 1.0 | 0.017 |
|  | Na^+^ | 1.0 | 1.0 | 1.0 | 1.0 | 1.0 | 1.0 | 1.0 | 1.0 | 1.0 | 0.069 |
|  | NH_4_^+^ | 0.441 | 0.009 | 0.441 | na | na | na | 0.441 | 0.688 | na | 0.323 |
|  | NO_3_^–^ | 1.0 | 0.727 | 1.0 | 0.589 | 1.0 | 1.0 | 0.892 | 1.0 | 1.0 | 0.130 |
|  | SO_4_^2–^ | 1.0 | 0.218 | 1.0 | 1.0 | 1.0 | 1.0 | 0.030 | 0.667 | 0.667 | 0.172 |

Sampling depth, cm: 1: 0 – 10, 2: 10 – 20, 3: 20 – 200, 4: 200 – 280. P-values <0.05 are marked in red.

## *Table S 6. Average content and frequency of occurrence of mineral phases in Andosols of different thermal zones and in samples of different temperatures*

| Mineral | Zone | | | | Temperature, °C | | | |
| --- | --- | --- | --- | --- | --- | --- | --- | --- |
|  | I | II | III | IV | <30 | 30 – 50 | 50 – 80 | >80 |
| Albite (probably) | 3a | 3a | 2c | 2b | 3a | 3b | 2c | 2b |
| Anatase | 1b | 2a | 3a | 3a | 1b | 2a | 4a | 3a |
| Anorthite (probably) | 5a | 4a | 2b | 1c | 5a | 3b | 2c | 1b |
| Boehmite | 0– | 0– | 1d | 2b | 0– | 0– | 0– | 3b |
| Heulandite (probably) | 2c | 2d | 1c | 0– | 1c | 2c | 2b | 0– |
| Hematite | 1c | 1b | 1d | 2b | 1c | 1c | 1c | 2b |
| Goethite | 1c | 1c | 0– | 2b | 1d | 1b | 0– | 2b |
| Calcite | 0– | 0– | 1d | 1c | 0– | 0– | 1d | 1d |
| Kaolinite | 3a | 3a | 5a | 5a | 3a | 3a | 5a | 5a |
| Quartz | 2a | 4a | 3b | 3a | 3a | 4b | 3b | 3a |
| Clinoptilolite (probably) | 3a | 3a | 3b | 2c | 3a | 3b | 4a | 2c |
| Cristobalite | 2b | 2b | 2c | 3b | 2b | 2b | 1c | 3b |
| Marcasite | 0– | 0– | 0– | 1c | 0– | 0– | 0– | 1d |
| Feldspars | 3a | 4a | 2b | 2b | 4a | 4a | 2b | 2b |
| Pyrite | 1b | 1b | 1b | 2b | 1b | 1b | 1b | 2b |
| Pyrolusite | 0– | 0– | 0– | 1c | 0– | 0– | 0– | 1d |
| Smectite | 5a | 5b | 4b | 2d | 5a | 5b | 3b | 2d |
| Jarosite | 0– | 0– | 0– | 1c | 0– | 0– | 0– | 1d |

Average contents, %: 1 – <1; 2 – 1–5; 3 – 5–10; 4 – 10–20; 5 – >20. Mineral phase occurrence frequencies: a – always, b – frequently, c – rarely, d – very rarely, dash – not found.

## *Table S 7. Results of the Mann-Whitney U-test for Andosols in zones I – IV*

| **Properties** | **I & II** | **I & III** | **I & IV** | **II & III** | **II & IV** | **III & IV** |
| --- | --- | --- | --- | --- | --- | --- |
| PM_1000–500_ | 0.279 | 0.072 | 0.020 | 0.279 | 0.023 | 0.102 |
| PM_500–250_ | 0.146 | 0.146 | 0.021 | 0.425 | 0.102 | 0.132 |
| PM_250–50_ | 0.759 | 0.221 | 0.004 | 0.207 | 0.001 | 0.207 |
| PM_50–10_ | 0.113 | 0.937 | 0.243 | 0.243 | 0.937 | 0.243 |
| PM_10–5_ | 0.056 | 0.027 | 0.058 | 1.000 | 1.000 | 1.000 |
| PM_5–1_ | 0.050 | 0.001 | 0.002 | 0.097 | 0.002 | 0.097 |
| PM_<1_ | 0.074 | 0.001 | 0.001 | 0.026 | 0.001 | 0.074 |
| рН | 0.453 | 0.000 | 0.000 | 0.000 | 0.000 | 0.137 |
| EC | 0.114 | 0.000 | 0.000 | 0.001 | 0.000 | 0.000 |
| LOI | 1.000 | 0.653 | 0.653 | 1.000 | 1.000 | 1.000 |
| SOC | 1.000 | 1.000 | 1.000 | 1.000 | 1.000 | 1.000 |
| Eh | 0.241 | 0.000 | 0.000 | 0.000 | 0.000 | 0.670 |
| TiO_2_ | 0.034 | 0.166 | 0.006 | 0.006 | 0.000 | 0.043 |
| V | 0.011 | 0.294 | 0.294 | 0.007 | 0.025 | 0.935 |
| Cr | 0.321 | 0.183 | 0.001 | 0.018 | 0.001 | 0.008 |
| MnO | 0.000 | 0.000 | 0.000 | 0.000 | 0.000 | 0.921 |
| Fe_2_O_3_ | 0.001 | 0.026 | 0.001 | 0.955 | 0.001 | 0.001 |
| Co | 0.006 | 0.726 | 0.001 | 0.726 | 0.001 | 0.001 |
| Ni | 0.339 | 0.128 | 0.257 | 0.339 | 0.257 | 0.059 |
| Cu | 0.826 | 0.162 | 0.005 | 0.332 | 0.023 | 0.401 |
| Zn | 1.000 | 1.000 | 1.000 | 1.000 | 1.000 | 1.000 |
| As | 0.233 | 0.000 | 0.007 | 0.000 | 0.015 | 0.839 |
| Pb | 0.390 | 0.000 | 0.014 | 0.000 | 0.016 | 0.807 |
| CaO | 0.015 | 0.000 | 0.000 | 0.000 | 0.000 | 0.010 |
| Al_2_O_3_ | 0.268 | 0.011 | 0.001 | 0.008 | 0.001 | 0.118 |
| SiO_2_ | 0.001 | 0.182 | 0.001 | 0.182 | 0.001 | 0.008 |
| P_2_O_5_ | 0.648 | 0.582 | 1.000 | 1.000 | 1.000 | 1.000 |
| K_2_O | 0.000 | 0.013 | 0.001 | 0.000 | 0.001 | 0.084 |
| MgO | 0.826 | 0.000 | 0.000 | 0.000 | 0.001 | 0.659 |
| Sr | 0.003 | 0.002 | 0.830 | 0.666 | 0.653 | 0.215 |
| Kaolinite | 0.890 | 0.000 | 0.002 | 0.000 | 0.002 | 0.890 |
| Smectite | 0.524 | 0.006 | 0.022 | 0.000 | 0.034 | 0.524 |
| Microcline | 0.084 | 0.002 | 0.002 | 0.000 | 0.002 | 0.009 |
| Albite | 1.000 | 0.000 | 0.003 | 0.000 | 0.003 | 1.000 |
| Anorthite | 0.409 | 0.000 | 0.005 | 0.000 | 0.003 | 0.688 |
| Clinoptilolite | 0.314 | 1.000 | 0.004 | 1.000 | 0.004 | 0.020 |
| Heulandite | 1.000 | 1.000 | 0.501 | 1.000 | 1.000 | 0.491 |
| Anatase | 0.125 | 0.000 | 0.002 | 0.002 | 0.004 | 0.767 |
| Pyrite | 1.000 | 1.000 | 1.000 | 1.000 | 1.000 | 1.000 |
| Quartz | 0.000 | 1.000 | 0.591 | 0.010 | 0.011 | 1.000 |
| Cristobalite | 0.564 | 0.564 | 0.357 | 0.118 | 0.558 | 0.047 |
| Hematite | 0.897 | 0.983 | 0.017 | 0.983 | 0.039 | 0.023 |
| Goethite | 0.613 | 0.071 | 0.120 | 0.082 | 0.120 | 0.002 |
| Jarosite | - | - | 0.252 | - | 0.252 | 0.183 |
| Marcasite | - | - | 0.252 | - | 0.252 | 0.183 |
| Pyrolusite | - | - | 0.252 | - | 0.252 | 0.183 |
| Calcite | - | 0.893 | 0.629 | 0.893 | 0.629 | 0.893 |
| Boehmite | - | 0.869 | 0.003 | 0.869 | 0.004 | 0.004 |
| HCO_3_– | 0.016 | 0.000 | 0.000 | 0.000 | 0.000 | 0.064 |
| Na^+^ | 0.604 | 0.806 | 0.604 | 0.957 | 0.604 | 0.291 |
| NH_4_^+^ | 1.000 | 0.033 | 0.034 | 0.046 | 0.045 | 1.000 |
| K^+^ | 0.466 | 1.000 | 1.000 | 0.775 | 0.466 | 0.998 |
| Mg^2+^ | 0.121 | 0.056 | 0.027 | 0.616 | 0.116 | 0.083 |
| Ca^2+^ | 0.008 | 0.004 | 0.017 | 0.727 | 0.750 | 0.750 |
| F^–^ | 0.138 | 0.029 | 0.766 | 0.766 | 0.766 | 0.766 |
| Cl^–^ | 1.000 | 1.000 | 1.000 | 1.000 | 1.000 | 1.000 |
| NO_3_^–^ | 1.000 | 0.008 | 1.000 | 0.026 | 1.000 | 0.070 |
| HPO_4_^2–^ | 1.000 | 1.000 | 1.000 | 1.000 | 1.000 | 1.000 |
| SO_4_^–^ | 0.022 | 0.000 | 0.000 | 0.000 | 0.000 | 0.001 |
| Al_ox_ | 0.229 | 0.024 | 0.143 | 0.461 | 1.000 | 0.461 |
| Si_ox_ | 0.171 | 0.050 | 0.114 | 0.093 | 0.171 | 0.171 |
| Fe_ox_ | 0.229 | 0.024 | 0.971 | 0.971 | 0.343 | 0.040 |

P-values <0.05 are marked in red.

## *Table S 8. Properties of Andosols studied in zones I – IV*

| **Properties** | | **Zone** | **Me** | **MAD** | **M** | **SD** | **Normality test p–value** | | | **n** |
| --- | --- | --- | --- | --- | --- | --- | --- | --- | --- | --- |
|  |  |  |  |  |  |  | **Shapiro** | **Lillie** | **Anderson** |  |
| Physico–chemical features | SOC, g/100g | I | 1.5 | 2.1 | 4.5 | 5.7 | 0.001 | 0.004 | 0.001 | 17 |
|  |  | II | 1.3 | 1.7 | 3.5 | 3.8 | 0.005 | 0.003 | 0.004 | 14 |
|  |  | III | 1.4 | 1.7 | 2.9 | 3.7 | 0.000 | 0.003 | 0.000 | 19 |
|  |  | IV | 1.3 | 0.9 | 1.9 | 1.6 | 0.075 | 0.089 | NA | 6 |
|  | pH | I | 6.2 | 0.3 | 6.1 | 0.6 | 0.177 | 0.111 | 0.089 | 17 |
|  |  | II | 5.8 | 0.3 | 5.9 | 0.5 | 0.435 | 0.725 | 0.452 | 15 |
|  |  | III | 3.7 | 0.4 | 3.8 | 0.6 | 0.030 | 0.062 | 0.017 | 26 |
|  |  | IV | 3.4 | 0.5 | 3.4 | 0.4 | 0.774 | 0.538 | 0.682 | 8 |
|  | Eh, mV | I | 478 | 77 | 356 | 248 | 0.000 | 0.005 | 0.000 | 17 |
|  |  | II | 167 | 518 | 141 | 371 | 0.006 | 0.017 | 0.005 | 14 |
|  |  | III | 609 | 59 | 533 | 246 | 0.000 | 0.000 | 0.000 | 26 |
|  |  | IV | 613 | 28 | 610 | 22 | 0.575 | 0.783 | 0.658 | 8 |
|  | EC, µS/cm | I | 71 | 61 | 94 | 70 | 0.034 | 0.323 | 0.052 | 17 |
|  |  | II | 81 | 49 | 128 | 80 | 0.020 | 0.009 | 0.011 | 15 |
|  |  | III | 263 | 164 | 434 | 441 | 0.000 | 0.000 | 0.000 | 26 |
|  |  | IV | 2240 | 1263 | 2279 | 962 | 0.420 | 0.669 | 0.551 | 8 |
|  | LOI | I | 6.6 | 3.7 | 11.0 | 10.7 | 0.002 | 0.006 | 0.001 | 14 |
|  |  | II | 7.0 | 5.0 | 11.4 | 7.7 | 0.020 | 0.003 | 0.012 | 14 |
|  |  | III | 11 | 4 | 13 | 10 | 0.000 | 0.002 | 0.000 | 24 |
|  |  | IV | 11.8 | 2.7 | 12.1 | 1.9 | 0.387 | 0.459 | NA | 7 |
| Grain–size fractions, vol. % | PM_1000–500_ | I | 37.0 | 23.9 | 36.2 | 24.9 | 0.422 | 0.894 | 0.627 | 17 |
|  |  | II | 22.1 | 16.3 | 22.8 | 17.0 | 0.343 | 0.668 | 0.517 | 15 |
|  |  | III | 10.9 | 16.2 | 17.2 | 18.8 | 0.001 | 0.024 | 0.000 | 26 |
|  |  | IV | 0 | 0 | 2.0 | 4.3 | 0.000 | 0.004 | NA | 7 |
|  | PM_500–250_ | I | 19.8 | 7.1 | 18.7 | 9.5 | 0.029 | 0.024 | 0.026 | 17 |
|  |  | II | 15.0 | 7.0 | 13.9 | 7.1 | 0.195 | 0.708 | 0.289 | 15 |
|  |  | III | 9.3 | 13.8 | 12.8 | 11.5 | 0.019 | 0.139 | 0.038 | 26 |
|  |  | IV | 0 | 0 | 3.4 | 7.6 | 0.000 | 0.002 | NA | 7 |
|  | PM_250–50_ | I | 17.2 | 8.3 | 17.5 | 8.6 | 0.997 | 1.000 | 0.992 | 17 |
|  |  | II | 20.2 | 7.1 | 19.3 | 6.7 | 0.983 | 0.805 | 0.885 | 15 |
|  |  | III | 15.6 | 7.1 | 12.6 | 8.7 | 0.016 | 0.056 | 0.016 | 26 |
|  |  | IV | 0.7 | 0.7 | 3.7 | 5.6 | 0.002 | 0.001 | NA | 7 |
|  | PM_50–10_ | I | 10.7 | 7.0 | 16.9 | 16.2 | 0.001 | 0.003 | 0.000 | 17 |
|  |  | II | 24.7 | 8.0 | 26.1 | 13.8 | 0.008 | 0.008 | 0.005 | 15 |
|  |  | III | 15.8 | 10.2 | 17.0 | 10.9 | 0.419 | 0.479 | 0.468 | 26 |
|  |  | IV | 22.8 | 4.1 | 24.9 | 9.4 | 0.757 | 0.478 | NA | 7 |
|  | PM_10–5_ | I | 1.5 | 1.3 | 3.3 | 3.8 | 0.001 | 0.001 | 0.000 | 17 |
|  |  | II | 6.4 | 2.8 | 6.2 | 3.0 | 0.590 | 0.869 | 0.640 | 15 |
|  |  | III | 5.4 | 3.9 | 6.4 | 4.1 | 0.087 | 0.348 | 0.135 | 26 |
|  |  | IV | 9.5 | 8.5 | 10.1 | 7.6 | 0.499 | 0.343 | NA | 7 |
|  | PM_5–1_ | I | 1.6 | 1.6 | 4.5 | 6.0 | 0.000 | 0.003 | 0.000 | 17 |
|  |  | II | 8.3 | 3.9 | 7.9 | 4.2 | 0.796 | 0.951 | 0.820 | 15 |
|  |  | III | 11.9 | 13.3 | 19.9 | 17.9 | 0.002 | 0.001 | 0.001 | 26 |
|  |  | IV | 35.3 | 13.2 | 30.9 | 10.6 | 0.676 | 0.300 | NA | 7 |
|  | PM_1_ | I | 0.4 | 0.4 | 1.2 | 1.6 | 0.000 | 0.001 | 0.000 | 17 |
|  |  | II | 1.9 | 1.2 | 1.9 | 1.0 | 0.789 | 0.973 | 0.889 | 15 |
|  |  | III | 3.6 | 4.3 | 11.5 | 13.8 | 0.000 | 0.000 | 0.000 | 26 |
|  |  | IV | 16.9 | 10.5 | 17.5 | 8.1 | 0.461 | 0.680 | NA | 7 |
| Major elements, g/100g | Al_2_O_3_ | I | 22.0 | 4.4 | 20.4 | 4.6 | 0.040 | 0.024 | 0.029 | 17 |
|  |  | II | 20.0 | 3.0 | 19.6 | 3.4 | 0.129 | 0.072 | 0.133 | 15 |
|  |  | III | 30 | 17 | 34 | 16 | 0.117 | 0.249 | 0.151 | 26 |
|  |  | IV | 45 | 4 | 45 | 5 | 0.553 | 0.879 | 0.513 | 8 |
|  | CaO | I | 4.7 | 0.5 | 4.1 | 1.2 | 0.007 | 0.009 | 0.003 | 17 |
|  |  | II | 3.0 | 0.8 | 3.2 | 0.7 | 0.048 | 0.061 | 0.040 | 14 |
|  |  | III | 0.71 | 0.82 | 0.82 | 1.00 | 0.000 | 0.000 | 0.000 | 26 |
|  |  | IV | 0 | 0 | 0.03 | 0.06 | 0.000 | 0.000 | 0.000 | 8 |
|  | Fe_2_O_3_ | I | 10.1 | 1.3 | 9.8 | 1.9 | 0.848 | 0.644 | 0.568 | 17 |
|  |  | II | 7.7 | 0.7 | 7.6 | 0.9 | 0.271 | 0.320 | 0.192 | 15 |
|  |  | III | 7.7 | 1.9 | 9.4 | 6.1 | 0.000 | 0.000 | 0.000 | 26 |
|  |  | IV | 25 | 12 | 25 | 8 | 0.294 | 0.678 | 0.462 | 8 |
|  | K_2_O | I | 1.08 | 0.16 | 1.06 | 0.27 | 0.121 | 0.366 | 0.061 | 17 |
|  |  | II | 1.8 | 0.1 | 1.8 | 0.4 | 0.002 | 0.000 | 0.001 | 14 |
|  |  | III | 0.68 | 0.55 | 0.69 | 0.54 | 0.034 | 0.361 | 0.067 | 26 |
|  |  | IV | 0.18 | 0.04 | 0.23 | 0.14 | 0.010 | 0.002 | 0.008 | 8 |
|  | MgO | I | 1.55 | 0.07 | 1.54 | 0.10 | 0.685 | 0.764 | 0.545 | 17 |
|  |  | II | 1.53 | 0.04 | 1.54 | 0.10 | 0.073 | 0.047 | 0.029 | 14 |
|  |  | III | 0.72 | 0.30 | 0.72 | 0.36 | 0.791 | 0.852 | 0.766 | 26 |
|  |  | IV | 0.60 | 0.41 | 0.57 | 0.29 | 0.046 | 0.205 | 0.055 | 8 |
|  | MnO | I | 2205 | 314 | 2234 | 440 | 0.569 | 0.364 | 0.319 | 17 |
|  |  | II | 1818 | 145 | 1771 | 243 | 0.008 | 0.007 | 0.007 | 14 |
|  |  | III | 434 | 309 | 577 | 538 | 0.001 | 0.002 | 0.001 | 26 |
|  |  | IV | 451 | 182 | 488 | 255 | 0.346 | 0.521 | 0.340 | 8 |
|  | P_2_O_5_ | I | 0.30 | 0.18 | 0.31 | 0.15 | 0.936 | 0.969 | 0.938 | 17 |
|  |  | II | 0.22 | 0.14 | 0.25 | 0.11 | 0.051 | 0.179 | 0.070 | 14 |
|  |  | III | 0.24 | 0.10 | 0.24 | 0.10 | 0.687 | 0.296 | 0.596 | 26 |
|  |  | IV | 0.20 | 0.17 | 0.25 | 0.16 | 0.477 | 0.403 | 0.483 | 8 |
|  | SiO_2_ | I | 57.0 | 1.5 | 57.4 | 3.2 | 0.013 | 0.133 | 0.040 | 17 |
|  |  | II | 64 | 3 | 63 | 4 | 0.117 | 0.120 | 0.094 | 14 |
|  |  | III | 62 | 4 | 52 | 18 | 0.000 | 0.000 | 0.000 | 26 |
|  |  | IV | 30 | 11 | 27 | 10 | 0.621 | 0.863 | 0.688 | 8 |
|  | TiO_2_ | I | 1.4 | 0.3 | 1.4 | 0.3 | 0.905 | 0.589 | 0.727 | 17 |
|  |  | II | 1.2 | 0.1 | 1.2 | 0.1 | 0.271 | 0.577 | 0.290 | 15 |
|  |  | III | 1.6 | 0.5 | 1.7 | 0.6 | 0.450 | 0.145 | 0.308 | 26 |
|  |  | IV | 2.3 | 0.5 | 2.2 | 0.5 | 0.774 | 0.804 | 0.735 | 8 |
| Micro elements, mg/kg | As | I | 6.0 | 3.0 | 6.9 | 3.1 | 0.212 | 0.411 | 0.269 | 17 |
|  |  | II | 8.0 | 1.5 | 8.2 | 1.9 | 0.088 | 0.026 | 0.038 | 14 |
|  |  | III | 15 | 8 | 25 | 21 | 0.000 | 0.001 | 0.000 | 26 |
|  |  | IV | 18 | 2 | 22 | 12 | 0.123 | 0.010 | 0.042 | 8 |
|  | Co | I | 62 | 22 | 59 | 28 | 0.489 | 0.569 | 0.489 | 17 |
|  |  | II | 34 | 9 | 31 | 10 | 0.307 | 0.151 | 0.267 | 14 |
|  |  | III | 32 | 29 | 114 | 174 | 0.000 | 0.000 | 0.000 | 26 |
|  |  | IV | 930 | 340 | 752 | 426 | 0.178 | 0.181 | 0.170 | 8 |
|  | Cr | I | 46 | 4 | 44 | 6 | 0.111 | 0.124 | 0.094 | 17 |
|  |  | II | 45 | 2 | 44 | 3 | 0.015 | 0.044 | 0.021 | 14 |
|  |  | III | 48 | 5 | 59 | 29 | 0.000 | 0.000 | 0.000 | 26 |
|  |  | IV | 94 | 27 | 93 | 25 | 0.739 | 0.856 | 0.827 | 8 |
|  | Cu | I | 29 | 4 | 29 | 7 | 0.041 | 0.028 | 0.066 | 17 |
|  |  | II | 30 | 10 | 30 | 9 | 0.334 | 0.196 | 0.356 | 14 |
|  |  | III | 34 | 19 | 81 | 82 | 0.000 | 0.000 | 0.000 | 26 |
|  |  | IV | 65 | 42 | 86 | 60 | 0.048 | 0.168 | 0.059 | 8 |
|  | Ni | I | 34 | 3 | 34 | 3 | 0.253 | 0.452 | 0.385 | 17 |
|  |  | II | 35 | 2 | 35 | 3 | 0.755 | 0.608 | 0.703 | 14 |
|  |  | III | 38 | 10 | 48 | 22 | 0.000 | 0.000 | 0.000 | 26 |
|  |  | IV | 22 | 26 | 23 | 19 | 0.444 | 0.503 | 0.541 | 8 |
|  | Pb | I | 19 | 18 | 27 | 23 | 0.016 | 0.057 | 0.017 | 17 |
|  |  | II | 37 | 18 | 32 | 16 | 0.752 | 0.366 | 0.537 | 14 |
|  |  | III | 78 | 53 | 110 | 90 | 0.000 | 0.008 | 0.000 | 26 |
|  |  | IV | 91 | 22 | 98 | 55 | 0.519 | 0.156 | 0.275 | 8 |
|  | Sr | I | 243 | 24 | 245 | 46 | 0.031 | 0.042 | 0.015 | 17 |
|  |  | II | 291 | 34 | 300 | 38 | 0.056 | 0.012 | 0.046 | 14 |
|  |  | III | 312 | 93 | 394 | 205 | 0.000 | 0.000 | 0.000 | 26 |
|  |  | IV | 230 | 202 | 215 | 141 | 0.501 | 0.722 | 0.613 | 8 |
|  | V | I | 180 | 28 | 173 | 33 | 0.184 | 0.299 | 0.214 | 17 |
|  |  | II | 145 | 16 | 144 | 19 | 0.991 | 0.939 | 0.971 | 15 |
|  |  | III | 207 | 64 | 209 | 72 | 0.805 | 0.481 | 0.724 | 26 |
|  |  | IV | 214 | 56 | 208 | 60 | 0.710 | 0.845 | 0.709 | 8 |
|  | Zn | I | 133 | 10 | 136 | 29 | 0.000 | 0.001 | 0.000 | 17 |
|  |  | II | 128 | 23 | 134 | 22 | 0.374 | 0.514 | 0.444 | 14 |
|  |  | III | 131 | 45 | 215 | 165 | 0.000 | 0.000 | 0.000 | 26 |
|  |  | IV | 150 | 103 | 162 | 90 | 0.227 | 0.689 | 0.317 | 8 |
| Mineral composition, g/100g | Albite | I | 6.5 | 3.9 | 6.4 | 3.6 | 0.376 | 0.595 | 0.389 | 17 |
|  |  | II | 5.5 | 3.3 | 7.3 | 4.8 | 0.006 | 0.064 | 0.017 | 18 |
|  |  | III | 0 | 0 | 1.3 | 2.2 | 0.000 | 0.000 | 0.000 | 25 |
|  |  | IV | 0 | 0 | 1.2 | 2.0 | 0.001 | 0.033 | 0.002 | 9 |
|  | Anatase | I | 0.3 | 0.4 | 0.6 | 0.7 | 0.001 | 0.000 | 0.001 | 17 |
|  |  | II | 0.8 | 0.3 | 1.0 | 0.9 | 0.000 | 0.000 | 0.000 | 18 |
|  |  | III | 6.6 | 5.3 | 7.3 | 6.1 | 0.006 | 0.034 | 0.006 | 25 |
|  |  | IV | 8 | 3.4 | 7.2 | 3.7 | 0.383 | 0.665 | 0.374 | 9 |
|  | Anorthite | I | 39 | 26 | 30 | 22 | 0.016 | 0.035 | 0.014 | 17 |
|  |  | II | 17 | 4 | 17 | 7 | 0.207 | 0.076 | 0.126 | 18 |
|  |  | III | 0 | 0 | 3.0 | 4.3 | 0.000 | 0.000 | 0.000 | 25 |
|  |  | IV | 0 | 0 | 1.0 | 2.1 | 0.000 | 0.002 | 0.000 | 9 |
|  | Boehmite | I | 0 | 0 | 0 | 0 | NA | NA | NA | 17 |
|  |  | II | 0 | 0 | 0 | 0 | NA | NA | NA | 18 |
|  |  | III | 0 | 0 | 0.9 | 4.3 | 0.000 | 0.000 | 0.000 | 25 |
|  |  | IV | 3.6 | 4.5 | 3.4 | 3.0 | 0.074 | 0.311 | 0.125 | 9 |
|  | Calcite | I | 0 | 0 | 0 | 0 | NA | NA | NA | 17 |
|  |  | II | 0 | 0 | 0 | 0 | NA | NA | NA | 18 |
|  |  | III | 0 | 0 | 0.1 | 0.3 | 0.000 | 0.000 | 0.000 | 25 |
|  |  | IV | 0 | 0 | 0.5 | 1.0 | 0.000 | 0.000 | 0.000 | 9 |
|  | Clinoptilolite | I | 8.2 | 2.7 | 8.2 | 2.8 | 0.725 | 0.831 | 0.865 | 17 |
|  |  | II | 9.4 | 3.5 | 9.9 | 3.8 | 0.699 | 0.336 | 0.663 | 18 |
|  |  | III | 9.1 | 8.5 | 9.1 | 6.7 | 0.112 | 0.293 | 0.146 | 25 |
|  |  | IV | 0 | 0 | 1.9 | 4.4 | 0.000 | 0.000 | 0.000 | 9 |
|  | Cristobalite | I | 0.2 | 0.3 | 1.7 | 2.6 | 0.000 | 0.003 | 0.000 | 17 |
|  |  | II | 0.9 | 0.8 | 1.8 | 2.4 | 0.000 | 0.000 | 0.000 | 18 |
|  |  | III | 0 | 0 | 2.4 | 5.3 | 0.000 | 0.000 | 0.000 | 25 |
|  |  | IV | 2.4 | 3.6 | 7.9 | 9.7 | 0.014 | 0.025 | 0.013 | 9 |
|  | Goethite | I | 0 | 0 | 0.4 | 0.6 | 0.000 | 0.000 | 0.000 | 17 |
|  |  | II | 0 | 0 | 0.2 | 0.3 | 0.000 | 0.000 | 0.000 | 18 |
|  |  | III | 0 | 0 | 0.1 | 0.5 | 0.000 | 0.000 | 0.000 | 25 |
|  |  | IV | 0.7 | 1.0 | 3.4 | 4.3 | 0.029 | 0.028 | 0.032 | 9 |
|  | Hematite | I | 0 | 0 | 0.2 | 0.3 | 0.000 | 0.000 | 0.000 | 17 |
|  |  | II | 0.1 | 0.1 | 0.2 | 0.4 | 0.000 | 0.000 | 0.000 | 18 |
|  |  | III | 0 | 0 | 0.8 | 2.1 | 0.000 | 0.000 | 0.000 | 25 |
|  |  | IV | 3.1 | 3.9 | 4.3 | 5.2 | 0.029 | 0.054 | 0.035 | 9 |
|  | Heulandite | I | 0 | 0 | 1.5 | 2.4 | 0.000 | 0.000 | 0.000 | 17 |
|  |  | II | 0 | 0 | 0.5 | 1.3 | 0.000 | 0.000 | 0.000 | 18 |
|  |  | III | 0 | 0 | 1.5 | 3.2 | 0.000 | 0.000 | 0.000 | 25 |
|  |  | IV | 0 | 0 | 0 | 0 | NA | NA | NA | 9 |
|  | Jarosite | I | 0 | 0 | 0 | 0 | NA | NA | NA | 17 |
|  |  | II | 0 | 0 | 0 | 0 | NA | NA | NA | 18 |
|  |  | III | 0 | 0 | 0 | 0 | NA | NA | NA | 25 |
|  |  | IV | 0 | 0 | 0.3 | 0.7 | 0.000 | 0.000 | 0.000 | 9 |
|  | Kaolinite | I | 6.2 | 1.0 | 6.4 | 1.7 | 0.461 | 0.287 | 0.268 | 17 |
|  |  | II | 5.2 | 2.0 | 7.0 | 6.2 | 0.000 | 0.000 | 0.000 | 18 |
|  |  | III | 54 | 17 | 51 | 23 | 0.390 | 0.078 | 0.241 | 25 |
|  |  | IV | 55 | 24 | 54 | 21 | 0.713 | 0.945 | 0.826 | 9 |
|  | Marcasite | I | 0 | 0 | 0 | 0 | NA | NA | NA | 17 |
|  |  | II | 0 | 0 | 0 | 0 | NA | NA | NA | 18 |
|  |  | III | 0 | 0 | 0 | 0 | NA | NA | NA | 25 |
|  |  | IV | 0 | 0 | 0.0 | 0.1 | 0.000 | 0.000 | 0.000 | 9 |
|  | Microcline | I | 9.6 | 3.3 | 9.4 | 3.1 | 0.807 | 0.913 | 0.863 | 17 |
|  |  | II | 10.5 | 2.7 | 12.1 | 3.3 | 0.013 | 0.016 | 0.012 | 18 |
|  |  | III | 3.7 | 3.3 | 4.7 | 3.6 | 0.043 | 0.011 | 0.028 | 25 |
|  |  | IV | 1.2 | 1.8 | 1.6 | 1.5 | 0.373 | 0.613 | 0.435 | 9 |
|  | Pyrite | I | 0.2 | 0.3 | 0.3 | 0.3 | 0.016 | 0.055 | 0.017 | 17 |
|  |  | II | 0.2 | 0.1 | 0.2 | 0.1 | 0.184 | 0.177 | 0.132 | 18 |
|  |  | III | 0.3 | 0.3 | 0.5 | 1.1 | 0.000 | 0.000 | 0.000 | 25 |
|  |  | IV | 0.6 | 0.9 | 1.4 | 2.7 | 0.000 | 0.000 | 0.000 | 9 |
|  | Pyrolusite | I | 0 | 0 | 0 | 0 | NA | NA | NA | 17 |
|  |  | II | 0 | 0 | 0 | 0 | NA | NA | NA | 18 |
|  |  | III | 0 | 0 | 0 | 0 | NA | NA | NA | 25 |
|  |  | IV | 0 | 0 | 0.1 | 0.4 | 0.000 | 0.000 | 0.000 | 9 |
|  | Quartz | I | 3.4 | 2.8 | 4.4 | 4.0 | 0.003 | 0.085 | 0.005 | 17 |
|  |  | II | 14 | 3 | 14 | 4 | 0.420 | 0.133 | 0.215 | 18 |
|  |  | III | 4.5 | 6.7 | 7.3 | 7.3 | 0.001 | 0.003 | 0.000 | 25 |
|  |  | IV | 8.2 | 9.2 | 8.0 | 6.0 | 0.222 | 0.449 | 0.275 | 9 |
|  | Smectite | I | 26 | 34 | 30 | 25 | 0.069 | 0.225 | 0.099 | 17 |
|  |  | II | 29 | 6 | 30 | 8 | 0.018 | 0.212 | 0.073 | 18 |
|  |  | III | 0.6 | 0.9 | 9.9 | 11.7 | 0.000 | 0.000 | 0.000 | 25 |
|  |  | IV | 0 | 0 | 3.7 | 11.0 | 0.000 | 0.000 | 0.000 | 9 |
| Short-range-order Al, Fe and Si compounds, g/kg | Al_ox_ | I | 9.69 | 2.62 | 11.35 | 5.56 | 0.254 | NA | NA | 4 |
|  |  | II | 2.54 | 0.70 | 2.71 | 0.91 | 0.739 | NA | NA | 4 |
|  |  | III | 1.72 | 0.44 | 2.20 | 1.24 | 0.005 | 0.007 | 0.007 | 8 |
|  |  | IV | 2.82 | 0.44 | 2.89 | 0.49 | 0.780 | NA | NA | 4 |
|  | Fe_ox_ | I | 12.92 | 3.35 | 14.26 | 5.35 | 0.487 | NA | NA | 4 |
|  |  | II | 3.68 | 1.00 | 4.33 | 2.04 | 0.249 | NA | NA | 4 |
|  |  | III | 4.93 | 2.97 | 4.16 | 2.60 | 0.241 | 0.427 | 0.233 | 8 |
|  |  | IV | 19.14 | 7.15 | 17.84 | 8.20 | 0.804 | NA | NA | 4 |
|  | Si_ox_ | I | 4.29 | 3.26 | 4.74 | 3.17 | 0.276 | NA | NA | 4 |
|  |  | II | 1.22 | 0.08 | 1.25 | 0.15 | 0.391 | NA | NA | 4 |
|  |  | III | 0.39 | 0.14 | 0.43 | 0.16 | 0.769 | 0.498 | 0.564 | 8 |
|  |  | IV | 0.27 | 0.20 | 0.26 | 0.20 | 0.971 | NA | NA | 4 |
| Cation composition, mmol (+)/100g | Ca^2+^ | I | 10 | 5 | 16 | 12 | 0.000 | 0.001 | 0.000 | 15 |
|  |  | II | 34 | 11 | 31 | 13 | 0.871 | 0.519 | 0.697 | 16 |
|  |  | III | 28 | 11 | 32 | 18 | 0.001 | 0.001 | 0.000 | 26 |
|  |  | IV | 30 | 14 | 46 | 33 | 0.042 | 0.139 | NA | 7 |
|  | K^+^ | I | 0.73 | 0.53 | 1.81 | 2.29 | 0.000 | 0.000 | 0.000 | 15 |
|  |  | II | 1.7 | 1.4 | 2.6 | 2.3 | 0.002 | 0.021 | 0.001 | 16 |
|  |  | III | 1.8 | 1.7 | 1.9 | 1.5 | 0.025 | 0.310 | 0.069 | 26 |
|  |  | IV | 0.84 | 0.61 | 1.11 | 0.79 | 0.108 | 0.522 | NA | 7 |
|  | Mg^2+^ | I | 4.4 | 1.3 | 6.6 | 5.5 | 0.000 | 0.000 | 0.000 | 15 |
|  |  | II | 6.2 | 3.3 | 8.9 | 5.8 | 0.013 | 0.028 | 0.007 | 16 |
|  |  | III | 8.4 | 4.5 | 10.0 | 6.8 | 0.000 | 0.018 | 0.001 | 26 |
|  |  | IV | 14.0 | 10.1 | 32.1 | 37.1 | 0.006 | 0.123 | NA | 7 |
|  | Na^+^ | I | 4.8 | 1.1 | 4.6 | 1.3 | 0.017 | 0.257 | 0.055 | 15 |
|  |  | II | 5.2 | 1.5 | 5.3 | 1.4 | 0.400 | 0.878 | 0.600 | 16 |
|  |  | III | 5.0 | 3.7 | 8.2 | 10.6 | 0.000 | 0.000 | 0.000 | 26 |
|  |  | IV | 73 | 14 | 56 | 38 | 0.028 | 0.063 | NA | 7 |
|  | NH_4_^+^ | I | 0.032 | 0 | 0.27 | 0.62 | 0.000 | 0.000 | 0.000 | 15 |
|  |  | II | 0.032 | 0 | 0.46 | 0.93 | 0.000 | 0.000 | 0.000 | 16 |
|  |  | III | 0.82 | 1.17 | 3.69 | 5.32 | 0.000 | 0.000 | 0.000 | 26 |
|  |  | IV | 2.8 | 4.1 | 4.3 | 5.9 | 0.026 | 0.282 | NA | 7 |
| Anion composition, mmol (–)/100g | Cl^–^ | I | 1.3 | 0.6 | 1.4 | 0.6 | 0.737 | 0.549 | 0.758 | 15 |
|  |  | II | 1.1 | 0.4 | 1.2 | 0.4 | 0.381 | 0.426 | 0.396 | 16 |
|  |  | III | 1.4 | 0.5 | 1.7 | 1.1 | 0.000 | 0.001 | 0.000 | 26 |
|  |  | IV | 1.4 | 0.7 | 1.6 | 1.1 | 0.134 | 0.126 | NA | 7 |
|  | F^–^ | I | 0.83 | 0.52 | 0.87 | 0.46 | 0.915 | 0.904 | 0.887 | 15 |
|  |  | II | 2.3 | 1.4 | 2.8 | 3.1 | 0.000 | 0.014 | 0.001 | 16 |
|  |  | III | 1.7 | 1.1 | 1.9 | 1.4 | 0.004 | 0.073 | 0.045 | 26 |
|  |  | IV | 1.3 | 0.9 | 1.3 | 0.6 | 0.837 | 0.970 | NA | 7 |
|  | HCO_3_^–^ | I | 0.23 | 0.07 | 0.24 | 0.13 | 0.001 | 0.011 | 0.003 | 16 |
|  |  | II | 0.36 | 0.25 | 0.61 | 0.47 | 0.010 | 0.015 | 0.007 | 14 |
|  |  | III | 0 | 0 | 0.102 | 0.267 | 0.000 | 0.000 | 0.000 | 23 |
|  |  | IV | 0 | 0 | 0 | 0 | NA | NA | NA | 8 |
|  | HPO_4_^2–^ | I | 0 | 0 | 0.001 | 0.003 | 0.000 | 0.000 | 0.000 | 15 |
|  |  | II | 0 | 0 | 0.032 | 0.104 | 0.000 | 0.000 | 0.000 | 16 |
|  |  | III | 0 | 0 | 0.001 | 0.003 | 0.000 | 0.000 | 0.000 | 26 |
|  |  | IV | 0 | 0 | 0.026 | 0.057 | 0.000 | 0.002 | NA | 7 |
|  | NO_3_^–^ | I | 0.08 | 0.07 | 0.12 | 0.12 | 0.003 | 0.113 | 0.009 | 15 |
|  |  | II | 0.14 | 0.16 | 0.19 | 0.22 | 0.000 | 0.004 | 0.001 | 16 |
|  |  | III | 0.67 | 0.96 | 1.48 | 1.69 | 0.000 | 0.002 | 0.000 | 26 |
|  |  | IV | 0.17 | 0.12 | 0.14 | 0.09 | 0.714 | 0.377 | NA | 7 |
|  | SO_4_^2–^ | I | 0.25 | 0.22 | 0.37 | 0.36 | 0.003 | 0.041 | 0.004 | 15 |
|  |  | II | 0.51 | 0.29 | 0.73 | 0.72 | 0.000 | 0.001 | 0.000 | 16 |
|  |  | III | 5.1 | 4.8 | 14.9 | 20.4 | 0.000 | 0.000 | 0.000 | 26 |
|  |  | IV | 59 | 43 | 88 | 64 | 0.323 | 0.227 | NA | 7 |
| Exchangeable fraction, µg/kg | Na^+^ | I | 115 | 9 | 115 | 8 | NA | NA | NA | 2 |
|  |  | II | 160 | 51 | 847 | 1411 | 0.002 | NA | NA | 4 |
|  |  | III | 78 | 18 | 88 | 23 | 0.181 | 0.263 | NA | 7 |
|  |  | IV | 78 | 10 | 81 | 12 | 0.438 | NA | NA | 4 |

## *Table S 9. Results of the Mann-Whitney U-test for topsoil in zones I – IV*

| **Properties** | **Analyses** | **I & II** | **I & III** | **I & IV** | **II & III** | **II & IV** | **III & IV** |
| --- | --- | --- | --- | --- | --- | --- | --- |
| Physico-chemical features | EC | 1.0 | 0.017 | 1.0 | 1.0 | 1.0 | 1.0 |
|  | LOI | 1.0 | 0.100 | 1.0 | 1.0 | 1.0 | 1.0 |
|  | Eh | 0.800 | 0.017 | 0.533 | 0.364 | 0.800 | 0.800 |
|  | SOC | 1.0 | 0.067 | 0.533 | 0.364 | 1.0 | 1.0 |
|  | рН | 1.0 | 0.953 | 0.953 | 1.0 | 1.0 | 1.0 |
| Grain-size fractions | PM_1_ | 1.0 | 0.643 | 0.455 | 1.0 | 1.0 | 0.455 |
|  | PM_1000–500_ | 1.0 | 1.0 | 0.800 | 1.0 | 1.0 | 0.800 |
|  | PM_10–5_ | 1.0 | 1.0 | 0.800 | 1.0 | 1.0 | 0.800 |
|  | PM_250–50_ | 1.0 | 0.453 | 1.0 | 1.0 | 1.0 | 0.727 |
|  | PM_500–250_ | 0.667 | 0.222 | 0.667 | 1.0 | 1.0 | 1.0 |
|  | PM_50–10_ | 1.0 | 1.0 | 0.800 | 1.0 | 1.0 | 1.0 |
|  | PM_5–1_ | 1.0 | 0.756 | 0.501 | 1.0 | 1.0 | 0.218 |
| Major elements | Al_2_O_3_ | 1.0 | 0.039 | 0.667 | 1.0 | 1.0 | 1.0 |
|  | CaO | 1.0 | 0.206 | 0.276 | 1.0 | 1.0 | 1.0 |
|  | Fe_2_O_3_ | 1.0 | 0.150 | 0.623 | 0.623 | 1.0 | 0.623 |
|  | K_2_O | 1.0 | 1.0 | 1.0 | 1.0 | 1.0 | 1.0 |
|  | MgO | 1.0 | 1.0 | 1.0 | 1.0 | 1.0 | 1.0 |
|  | MnO | 1.0 | 0.117 | 0.667 | 1.0 | 1.0 | 0.667 |
|  | P_2_O_5_ | 0.667 | 0.017 | 0.667 | 0.667 | 0.667 | 0.667 |
|  | SiO_2_ | 0.875 | 0.601 | 0.601 | 1.0 | 1.0 | 1.0 |
|  | TiO_2_ | 1.0 | 0.149 | 0.501 | 0.622 | 1.0 | 0.622 |
| Micro elements | As | 1.0 | 1.0 | 0.953 | 1.0 | 1.0 | 1.0 |
|  | Co | 1.0 | 0.149 | 0.495 | 0.379 | 1.0 | 0.495 |
|  | Cr | 1.0 | 1.0 | 1.0 | 1.0 | 1.0 | 1.0 |
|  | Cu | 1.0 | 0.120 | 1.0 | 1.0 | 1.0 | 1.0 |
|  | Ni | 0.501 | 0.050 | 0.501 | 0.501 | 0.667 | 0.721 |
|  | Pb | 1.0 | 1.0 | 0.800 | 1.0 | 1.0 | 1.0 |
|  | Sr | 1.0 | 1.0 | 0.667 | 1.0 | 1.0 | 0.268 |
|  | V | 1.0 | 0.117 | 0.667 | 1.0 | 1.0 | 0.667 |
|  | Zn | 1.0 | 0.302 | 1.0 | 1.0 | 1.0 | 1.0 |
| Mineral composition | Albite | 1.0 | 1.0 | 0.383 | 1.0 | 1.0 | 0.294 |
|  | Anatase | 1.0 | 1.0 | 0.985 | 1.0 | 1.0 | 1.0 |
|  | Anorthite | 1.0 | 1.0 | 0.459 | 1.0 | 1.0 | 1.0 |
|  | Clinoptilolite | 1.0 | 1.0 | 1.0 | 1.0 | 1.0 | 1.0 |
|  | Cristobalite | 1.0 | 1.0 | 1.0 | 0.604 | 1.0 | 0.245 |
|  | Goethite | 0.745 | 1.0 | 0.318 | 0.318 | 1.0 | 0.157 |
|  | Hematite | 1.0 | 1.0 | 0.984 | 1.0 | 1.0 | 1.0 |
|  | Heulandite | 1.0 | 1.0 | 0.920 | 1.0 | 1.0 | 0.079 |
|  | Kaolinite | 1.0 | 1.0 | 1.0 | 1.0 | 1.0 | 1.0 |
|  | Microcline | 1.0 | 1.0 | 0.459 | 1.0 | 1.0 | 1.0 |
|  | Pyrite | 1.0 | 1.0 | 0.433 | 1.0 | 1.0 | 0.945 |
|  | Quartz | 1.0 | 1.0 | 1.0 | 1.0 | 1.0 | 1.0 |
|  | Smectite | 1.0 | 1.0 | 0.459 | 1.0 | 1.0 | 1.0 |
| Cation-anion composition | Ca^2+^ | 1.0 | 0.218 | 1.0 | 1.0 | 1.0 | 1.0 |
|  | Cl^–^ | 1.0 | 1.0 | 1.0 | 1.0 | 1.0 | 1.0 |
|  | F^–^ | 1.0 | 1.0 | 1.0 | 1.0 | 1.0 | 1.0 |
|  | HCO_3_^–^ | 1.0 | 0.358 | 1.0 | 1.0 | 1.0 | 1.0 |
|  | HPO_4_^2–^ | 1.0 | 1.0 | 1.0 | 1.0 | 1.0 | 1.0 |
|  | K^+^ | 1.0 | 0.218 | 1.0 | 0.218 | 1.0 | 1.0 |
|  | Mg^2+^ | 1.0 | 0.218 | 1.0 | 1.0 | 1.0 | 1.0 |
|  | Na^+^ | 1.0 | 1.0 | 1.0 | 1.0 | 1.0 | 1.0 |
|  | NH_4_^+^ | 0.441 | 0.009 | 0.441 | 1.0 | 1.0 | 1.0 |
|  | NO_3_^–^ | 1.0 | 0.727 | 1.0 | 0.589 | 1.0 | 1.0 |
|  | SO_4_^2–^ | 1.0 | 0.218 | 1.0 | 1.0 | 1.0 | 1.0 |

P-values <0.05 are marked in red.

## *Table S 10. The chemical composition of topsoil (0 – 10 cm) in non-heated Andosols of the Kamchatka Peninsula*

| Element | Silandic Andosols (Arenic) | Aluandic Andosols (Loamic) | Gleyic Aluandic Andosols (Loamic) | Gleyic Aluandic Andosols (Clayic) | Silandic Andosols (Arenic) in the Kamchatka peninsula | | |
| --- | --- | --- | --- | --- | --- | --- | --- |
|  |  |  |  |  | Whole peninsula | North Province | Central Province |
| Al, g/100g | 5.4 | 6.3 | **10** | **16** | 5.1 | na | na |
| As, mg/kg | 6.5 | 8.0 | **12** | **18** | 7.2 | 6.6 | 1.8 |
| Ca, g/100g | 3.6 | **2.9** | **0.7** | **0.0** | 2.2 | na | na |
| Co, mg/kg | 36 | **27** | 31 | **930** | 10 | 6 | 5.9 |
| Cr, mg/kg | 49 | 45 | 48 | **94** | 42 | 43 | 11 |
| Cu, mg/kg | 26 | 24 | 32 | **65** | 35 | 59 | 41 |
| Fe, g/100g | 6.0 | **5.5** | **5.3** | **17** | 2.5 | na | na |
| K, g/100g | 0.90 | **1.51** | **0.68** | **0.15** | 1.3 | na | na |
| Mg, g/100g | 0.94 | 0.92 | **0.47** | **0.36** | 0.89 | na | na |
| Mn, mg/kg | 1550 | **1441** | **394** | **349** | 700 | 548 | 515 |
| Ni, mg/kg | 38 | 35 | 36 | 22 | 18 | 18 | 6.9 |
| P, g/100g | 0.21 | 0.15 | 0.11 | 0.09 | na | 0.3 | 0.13 |
| Pb, mg/kg | 20 | 25 | **63** | **91** | 7.8 | 6.3 | 11 |
| Si, g/100g | 26 | **28** | 29 | **14** | na | na | na |
| Sr, mg/kg | 237 | **286** | **320** | 230 | 251 | 139 | 65 |
| Ti, g/100g | 0.48 | **0.50** | **0.68** | **1.00** | 0.24 | 0.3 | 0.19 |
| V, mg/kg | 154 | **145** | **196** | 214 | 90 | 92 | 70 |
| Zn, mg/kg | 143 | 138 | 118 | 150 | 60 | 37 | 45 |
| n | 4 | 9 | 22 | 8 | 290 | 80 | 120 |
| References | This study | | | | ^21^ | ^22^ | |

Element concentrations in topsoil in zones II-IV significantly different from those in zone I are shown in bold.

## *Table S 11. Relative contents of major minerals in samples from soils of different temperatures*

| Groups of minerals | Temperature, °C | | | |
| --- | --- | --- | --- | --- |
|  | <30 | 30 – 50 | 50 – 80 | >80 |
| Clay | *Sm > K* | *Sm > K* | *K > Sm* | *K > Sm* |
| Non-clay | *Pl > Zt > Qz* | *Pl > Qz > Zt* | *Zt >Qz> Pl* | *Qz> Pl > Zt* |
| Plagioclases and feldspars | *An > Mс > Ab* | *An = Mс> Ab* | *Mс > An > Ab* | *Mс > An > Ab* |
| Fe-containing | *Py > Hem ** | *Py = Hem* | *Py / Hem *** | *Hem > Gth > Py ** |
| Zeolites | *Cpt > Hul* | *Cpt > Hul* | *Cpt > Hul* | No* |

*Predominantly. **One of two minerals present in a sample. *Sm* – smectite, *K* – kaolinite, *An* – anorthite, *Mc* – microcline, *Ab* – albite, *Py* – pyrite, *Hem* – hematite, *Cpt* – clinoptilolite, *Hul* – heulandite, *Pl* – plagioclases, *Zt* – zeolites, *Qz* – quartz, *Gth* – goethite.

## *Table S 12. Net Relatedness Index (NRI) calculated using three phylogenetic trees of the flora of the Geysernaya River valley*

| Flora | Magnoliophyta | | | Vascular plants | | |
| --- | --- | --- | --- | --- | --- | --- |
|  | NRI* | P-value* | N species | NRI* | P-value* | species |
| Species found at the distant territories (both non-heated and heated) were removed from the analysis | | | | | | |
| All thermophytes | 0.4±1.0 | 0.343±0.303 | 45 | 0.8±0.4 | 0.225±0.134 | 49 |
| Facultative thermophytes | 1.7±0.5 | **0.042±0.036** | 30 | 1.5±0.8 | 0.060±0.059 | 33 |
| Obligate thermophytes | 1.3±0.8 | 0.084±0.083 | 15 | 2.1±0.6 | **0.012±0.011** | 16 |
| Species found at the distant territories (both non-heated and heated) specified as in the L.I. Rassokhina’s list | | | | | | |
| All thermophytes | -0.2±0.6 | 0.587±0.231 | 154 | 0.7±0.6 | 0.247±0.161 | 167 |
| Facultative thermophytes | 0.4±0.8 | 0.532±0.233 | 130 | 0.9±0.8 | 0.198±0.186 | 142 |
| Obligate thermophytes | 2.4±1.1 | **0.014±0.013** | 24 | 3.6±0.5 | **0.001±0** | 25 |

* Me (median) and MAD (median absolute deviation). P-values < 0.05 are marked in bold.

## *Table S 13. Significantly overrepresented and underrepresented plant taxa of the flora of the Valley of Geysers*

| Taxa | Overrepresented | | Underrepresented | |
| --- | --- | --- | --- | --- |
|  | I | II | I | II |
| **Families** | | | | |
| Flora of the valley of the Geysernaya River, except species observed within and near the transect | *Ericaceae* | n.e. | *-* | n.e. |
| Non-thermal species | *Thelypteridaceae,*  *Equisetaceae,*  *Betulaceae* | *Ericaceae,*  *Saxifragaceae* | *-* | *Caryophyllaceae,*  *Juncaceae* |
| Facultative thermophytes | *-* | *Caryophyllaceae,*  *Asteraceae,*  *Onagraceae* | *-* | *Cyperaceae,*  *Ericaceae* |
| Obligate thermophytes | *Rosaceae* | *Cyperaceae* | *-* | *-* |
| All thermophytes | *Alliaceae* | *Caryophyllaceae,*  *Juncaceae* | *-* | *Ericaceae,*  *Saxifragaceae* |
| **Orders** | | | | |
| Flora of the valley of the Geysernaya River, except species observed within and near the transect | *Poales,*  *Ericales* | n.e. | *Asparagales* | n.e. |
| Non-thermal species | *Equisetales,*  *Fagales,*  *Polypodiales,*  *Liliales* | *Ericales* | *Poales* | *-* |
| Facultative thermophytes | *Asparagales* | *Caryophyllales,*  *Myrtales* | *Poales* | *Ericales* |
| Obligate thermophytes | *Rosales* | *Poales* | *-* | *-* |
| All thermophytes | *Asparagales,*  *Rosales* | *-* | *-* | *Ericales* |
| **Classes** | | | | |
| Flora of the valley of the Geysernaya River, except species observed within and near the catena | *-* | n.e. | *-* | n.e. |
| Non-thermal species | *Equisetopsida, Polypodiopsida* | *-* | *-* | *-* |
| Facultative thermophytes | *-* | *-* | *-* |  |
| Obligate thermophytes | *-* | *Liliopsida* | *-* | *Magnoliopsida* |
| All thermophytes | *-* | *-* | *-* |  |
| **Phylums** | | | | |
| Flora of the valley of the Geysernaya River, except species observed within and near the catena | *Magnoliophyta* | n.e. | *Polypodiophyta* | n.e. |
| Non-thermal species | *Polypodiophyta* | *-* | *Magnoliophyta* | *-* |
| Facultative thermophytes | *-* | *-* | *-* | *-* |
| Obligate thermophytes | *-* | *-* | *-* | *-* |
| All thermophytes | - | - | - | *-* |

I – catena (points 1 – 16) and its surrounding territory (groups T and B), II – the valley of the Geysernaya River: data from ^18,19^ supplemented by this study (all groups: T, B, Ya+R, see *Table S 14*). n.e. – not evaluated.

## *Table S 14. Register of species characterizing the flora of the valley of the Geysernaya River*

| Taxa | | | | | | Flora | | |
| --- | --- | --- | --- | --- | --- | --- | --- | --- |
| Species | Genus | Family | Order | Class | Phylum | Catena and its surrounding territory | | Geyzer Valley (all) |
|  |  |  |  |  |  | T | TB | Ya+R |
| *Acetosella vulgaris* | *Acetosella* | *Polygonaceae* | *Caryophyllales* | Magnoliopsida | Magnoliophyta | no | no | yes |
| *Aconitum fischeri* | *Aconitum* | *Ranunculaceae* | *Ranunculales* | Magnoliopsida | Magnoliophyta | yes | yes | yes |
| *Aconogonon tripterocarpum* | *Aconogonon* | *Polygonaceae* | *Caryophyllales* | Magnoliopsida | Magnoliophyta | no | no | yes |
| *Agrostis clavata* | *Agrostis* | *Poaceae* | *Poales* | Liliopsida | Magnoliophyta | no | no | yes |
| *Agrostis geminata* | *Agrostis* | *Poaceae* | *Poales* | Liliopsida | Magnoliophyta | yes | yes | yes |
| *Agrostis mertensii* | *Agrostis* | *Poaceae* | *Poales* | Liliopsida | Magnoliophyta | no | no | yes |
| *Agrostis pauzhetica* | *Agrostis* | *Poaceae* | *Poales* | Liliopsida | Magnoliophyta | no | no | yes |
| *Agrostis scabra* | *Agrostis* | *Poaceae* | *Poales* | Liliopsida | Magnoliophyta | no | yes | yes |
| *Agrostis vinealis* | *Agrostis* | *Poaceae* | *Poales* | Liliopsida | Magnoliophyta | no | no | yes |
| *Allium ochotense* | *Allium* | *Alliaceae* | *Asparagales* | Liliopsida | Magnoliophyta | yes | yes | yes |
| *Allium strictum* | *Allium* | *Alliaceae* | *Asparagales* | Liliopsida | Magnoliophyta | no | yes | yes |
| *Allocarya orientalis* | *Allocarya* | *Boraginaceae* | *Boraginales* | Magnoliopsida | Magnoliophyta | no | no | yes |
| *Alnus alnobetula* | *Alnus* | *Betulaceae* | *Fagales* | Magnoliopsida | Magnoliophyta | no | yes | yes |
| *Alopecurus stejnegeri* | *Alopecurus* | *Poaceae* | *Poales* | Liliopsida | Magnoliophyta | no | no | yes |
| *Anaphalis margaritacea* | *Anaphalis* | *Asteraceae* | *Synandrae* | Magnoliopsida | Magnoliophyta | no | no | yes |
| *Androsace chamaejasme* | *Androsace* | *Primulaceae* | *Ericales* | Magnoliopsida | Magnoliophyta | no | no | yes |
| *Androsace elongata* | *Androsace* | *Primulaceae* | *Ericales* | Magnoliopsida | Magnoliophyta | no | no | yes |
| *Anemone narcissiflora* | *Anemone* | *Ranunculaceae* | *Ranunculales* | Magnoliopsida | Magnoliophyta | no | no | yes |
| *Angelica genuflexa* | *Angelica* | *Apiaceae* | *Apiales* | Magnoliopsida | Magnoliophyta | no | no | yes |
| *Angelica lucida* | *Angelica* | *Apiaceae* | *Apiales* | Magnoliopsida | Magnoliophyta | yes | yes | yes |
| *Antennaria dioica* | *Antennaria* | *Asteraceae* | *Synandrae* | Magnoliopsida | Magnoliophyta | no | no | yes |
| *Antennaria microphylla* | *Antennaria* | *Asteraceae* | *Synandrae* | Magnoliopsida | Magnoliophyta | no | no | yes |
| *Arabidopsis lyrata* | *Arabidopsis* | *Brassicaceae* | *Brassicales* | Magnoliopsida | Magnoliophyta | no | yes | yes |
| *Arabis hirsuta* | *Arabis* | *Brassicaceae* | *Brassicales* | Magnoliopsida | Magnoliophyta | no | yes | yes |
| *Arabis stelleri* | *Arabis* | *Brassicaceae* | *Brassicales* | Magnoliopsida | Magnoliophyta | no | no | yes |
| *Arctous alpina* | *Arctous* | *Ericaceae* | *Ericales* | Magnoliopsida | Magnoliophyta | no | no | yes |
| *Arnica lessingii* | *Arnica* | *Asteraceae* | *Synandrae* | Magnoliopsida | Magnoliophyta | no | no | yes |
| *Artemisia arctica* | *Artemisia* | *Asteraceae* | *Synandrae* | Magnoliopsida | Magnoliophyta | no | no | yes |
| *Artemisia campestris ssp* | *Artemisia* | *Asteraceae* | *Synandrae* | Magnoliopsida | Magnoliophyta | no | no | yes |
| *Artemisia vulgaris* | *Artemisia* | *Asteraceae* | *Synandrae* | Magnoliopsida | Magnoliophyta | yes | yes | yes |
| *Aruncus dioicus* | *Aruncus* | *Rosaceae* | *Rosales* | Magnoliopsida | Magnoliophyta | yes | yes | yes |
| *Asplenium incisum* | *Asplenium* | *Aspleniaceae* | *Polypodiales* | Polypodiopsida | Polypodiophyta | no | no | yes |
| *Asplenium viride* | *Asplenium* | *Aspleniaceae* | *Polypodiales* | Polypodiopsida | Polypodiophyta | no | no | yes |
| *Astragalus alpinus* | *Astragalus* | *Fabaceae* | *Fabales* | Magnoliopsida | Magnoliophyta | no | no | yes |
| *Athyrium alpestre* | *Athyrium* | *Woodsiaceae* | *Polypodiales* | Polypodiopsida | Polypodiophyta | no | no | yes |
| *Athyrium filix* | *Athyrium* | *Woodsiaceae* | *Polypodiales* | Polypodiopsida | Polypodiophyta | no | no | yes |
| *Barbarea orthoceras* | *Barbarea* | *Brassicaceae* | *Brassicales* | Magnoliopsida | Magnoliophyta | no | no | yes |
| *Betula ermanii* | *Betula* | *Betulaceae* | *Fagales* | Magnoliopsida | Magnoliophyta | no | yes | yes |
| *Betula nana* | *Betula* | *Betulaceae* | *Fagales* | Magnoliopsida | Magnoliophyta | no | no | yes |
| *Bidens kamtschatica* | *Bidens* | *Asteraceae* | *Synandrae* | Magnoliopsida | Magnoliophyta | no | no | yes |
| *Bolboschoenus planiculmis* | *Bolboschoenus* | *Cyperaceae* | *Poales* | Liliopsida | Magnoliophyta | no | no | yes |
| *Botrychium lanceolatum* | *Botrychium* | *Ophioglossaceae* | *Ophioglossales* | Psilotopsida | Polypodiophyta | no | yes | yes |
| *Botrychium lunaria* | *Botrychium* | *Ophioglossaceae* | *Ophioglossales* | Psilotopsida | Polypodiophyta | no | no | yes |
| *Botrychium robustum* | *Botrychium* | *Ophioglossaceae* | *Ophioglossales* | Psilotopsida | Polypodiophyta | no | no | yes |
| *Bryanthus gmelinii* | *Bryanthus* | *Ericaceae* | *Ericales* | Magnoliopsida | Magnoliophyta | no | no | yes |
| *Bupleurum ranunculoides* | *Bupleurum* | *Apiaceae* | *Apiales* | Magnoliopsida | Magnoliophyta | no | no | yes |
| *Cacalia kamtschatica* | *Parasenecio* | *Asteraceae* | *Synandrae* | Magnoliopsida | Magnoliophyta | no | yes | yes |
| *Calamagrostis purpurascens* | *Calamagrostis* | *Poaceae* | *Poales* | Liliopsida | Magnoliophyta | no | no | yes |
| *Calamagrostis purpurea* | *Calamagrostis* | *Poaceae* | *Poales* | Liliopsida | Magnoliophyta | yes | yes | yes |
| *Calamagrostis sachalinensis* | *Calamagrostis* | *Poaceae* | *Poales* | Liliopsida | Magnoliophyta | no | no | yes |
| *Callitriche palustris* | *Callitriche* | *Callitrichaceae* | *Lamiales* | Magnoliopsida | Magnoliophyta | no | no | yes |
| *Campanula lasiocarpa* | *Campanula* | *Campanulaceae* | *Synandrae* | Magnoliopsida | Magnoliophyta | no | no | yes |
| *Capsella bursa* | *Capsella* | *Brassicaceae* | *Brassicales* | Magnoliopsida | Magnoliophyta | no | no | yes |
| *Cardamine flexuosa* | *Cardamine* | *Brassicaceae* | *Brassicales* | Magnoliopsida | Magnoliophyta | no | no | yes |
| *Cardamine hirsuta* | *Cardamine* | *Brassicaceae* | *Brassicales* | Magnoliopsida | Magnoliophyta | no | no | no+yes |
| *Carex brunnescens* | *Carex* | *Cyperaceae* | *Poales* | Liliopsida | Magnoliophyta | no | no | no+yes |
| *Carex canescens* | *Carex* | *Cyperaceae* | *Poales* | Liliopsida | Magnoliophyta | no | no | no+yes |
| *Carex curvula* | *Carex* | *Cyperaceae* | *Poales* | Liliopsida | Magnoliophyta | no | no | yes |
| *Carex flavocuspis* | *Carex* | *Cyperaceae* | *Poales* | Liliopsida | Magnoliophyta | no | no | yes |
| *Carex gaudichaudiana* | *Carex* | *Cyperaceae* | *Poales* | Liliopsida | Magnoliophyta | no | no | yes |
| *Carex longirostrata* | *Carex* | *Cyperaceae* | *Poales* | Liliopsida | Magnoliophyta | yes | yes | yes |
| *Carex macloviana* | *Carex* | *Cyperaceae* | *Poales* | Liliopsida | Magnoliophyta | no | no | yes |
| *Carex magellanica* | *Carex* | *Cyperaceae* | *Poales* | Liliopsida | Magnoliophyta | no | no | yes |
| *Carex podocarpa* | *Carex* | *Cyperaceae* | *Poales* | Liliopsida | Magnoliophyta | no | no | yes |
| *Carex saxatilis* | *Carex* | *Cyperaceae* | *Poales* | Liliopsida | Magnoliophyta | no | no | yes |
| *Cassiope lycopodioides* | *Cassiope* | *Ericaceae* | *Ericales* | Magnoliopsida | Magnoliophyta | no | no | yes |
| *Castilleja pallida* | *Castilleja* | *Scrophulariaceae* | *Lamiales* | Magnoliopsida | Magnoliophyta | no | no | yes |
| *Cerastium alpinum* | *Cerastium* | *Caryophyllaceae* | *Caryophyllales* | Magnoliopsida | Magnoliophyta | no | no | yes |
| *Cerastium fontanum* | *Cerastium* | *Caryophyllaceae* | *Caryophyllales* | Magnoliopsida | Magnoliophyta | no | no | yes |
| *Cicuta virosa* | *Cicuta* | *Apiaceae* | *Apiales* | Magnoliopsida | Magnoliophyta | no | yes | yes |
| *Circaea alpina* | *Circaea* | *Onagraceae* | *Myrtales* | Magnoliopsida | Magnoliophyta | no | no | yes |
| *Cirsium kamtschaticum* | *Cirsium* | *Asteraceae* | *Synandrae* | Magnoliopsida | Magnoliophyta | yes | yes | yes |
| *Clematis alpina* | *Atragene* | *Ranunculaceae* | *Ranunculales* | Magnoliopsida | Magnoliophyta | no | no | yes |
| *Comarum palustre* | *Comarum* | *Rosaceae* | *Rosales* | Magnoliopsida | Magnoliophyta | no | no | yes |
| *Corallorhiza trifida* | *Corallorhiza* | *Orchidaceae* | *Asparagales* | Liliopsida | Magnoliophyta | no | no | yes |
| *Cornus suecica* | *Cornus* | *Cornaceae* | *Cornales* | Magnoliopsida | Magnoliophyta | no | no | yes |
| *Cryptogramma crispa* | *Cryptogramma* | *Adiantaceae* | *Pteridales* | Polypodiopsida | Polypodiophyta | no | no | yes |
| *Cystopteris fragilis* | *Cystopteris* | *Woodsiaceae* | *Polypodiales* | Polypodiopsida | Polypodiophyta | no | no | yes |
| *Dactylorhiza aristata* | *Dactylorhiza* | *Orchidaceae* | *Asparagales* | Liliopsida | Magnoliophyta | yes | yes | yes |
| *Danthonia intermedia* | *Danthonia* | *Poaceae* | *Poales* | Liliopsida | Magnoliophyta | no | no | yes |
| *Dasiphora fruticosa* | *Potentilla* | *Rosaceae* | *Rosales* | Magnoliopsida | Magnoliophyta | no | no | yes |
| *Delphinium brachycentrum* | *Delphinium* | *Ranunculaceae* | *Ranunculales* | Magnoliopsida | Magnoliophyta | no | no | yes |
| *Deschampsia atropurpurea* | *Deschampsia* | *Poaceae* | *Poales* | Liliopsida | Magnoliophyta | no | no | yes |
| *Deschampsia cespitosa* | *Deschampsia* | *Poaceae* | *Poales* | Liliopsida | Magnoliophyta | no | no | no+yes |
| *Diapensia lapponica* | *Diapensia* | *Diapensiaceae* | *Ericales* | Magnoliopsida | Magnoliophyta | no | no | yes |
| *Diphasiastrum alpinum* | *Diphasiastrum* | *Lycopodiaceae* | *Lycopodiales* | Lycopodiopsida | Lycopodiophyta | no | no | yes |
| *Draba nemorosa* | *Draba* | *Brassicaceae* | *Brassicales* | Magnoliopsida | Magnoliophyta | no | no | yes |
| *Draba nivalis* | *Draba* | *Brassicaceae* | *Brassicales* | Magnoliopsida | Magnoliophyta | no | no | yes |
| *Dryas octopetala* | *Dryas* | *Rosaceae* | *Rosales* | Magnoliopsida | Magnoliophyta | no | no | yes |
| *Dryopteris expansa* | *Dryopteris* | *Dryopteridaceae* | *Polypodiales* | Polypodiopsida | Polypodiophyta | no | yes | yes |
| *Eleocharis kamtschatica* | *Eleocharis* | *Cyperaceae* | *Poales* | Liliopsida | Magnoliophyta | no | yes | yes |
| *Elymus repens* | *Elymus* | *Poaceae* | *Poales* | Liliopsida | Magnoliophyta | no | yes | yes |
| *Elymus trachycaulus* | *Elymus* | *Poaceae* | *Poales* | Liliopsida | Magnoliophyta | no | no | yes |
| *Empetrum nigrum* | *Empetrum* | *Empetraceae* | *Ericales* | Magnoliopsida | Magnoliophyta | no | no | yes |
| *Epilobium angustifolium* | *Epilobium* | *Onagraceae* | *Myrtales* | Magnoliopsida | Magnoliophyta | no | yes | yes |
| *Epilobium ciliatum* | *Epilobium* | *Onagraceae* | *Myrtales* | Magnoliopsida | Magnoliophyta | yes | yes | yes |
| *Epilobium fauriei* | *Epilobium* | *Onagraceae* | *Myrtales* | Magnoliopsida | Magnoliophyta | no | no | yes |
| *Epilobium hornemannii* | *Epilobium* | *Onagraceae* | *Myrtales* | Magnoliopsida | Magnoliophyta | no | no | yes |
| *Epilobium latifolium* | *Epilobium* | *Onagraceae* | *Myrtales* | Magnoliopsida | Magnoliophyta | no | no | yes |
| *Epilobium palustre* | *Epilobium* | *Onagraceae* | *Myrtales* | Magnoliopsida | Magnoliophyta | no | no | yes |
| *Equisetum arvense* | *Equisetum* | *Equisetaceae* | *Equisetales* | Equisetopsida | Polypodiophyta | ? | no | yes |
| *Equisetum sylvaticum* | *Equisetum* | *Equisetaceae* | *Equisetales* | Equisetopsida | Polypodiophyta | ? | no | yes |
| *Erigeron acris* | *Erigeron* | *Asteraceae* | *Synandrae* | Magnoliopsida | Magnoliophyta | no | no | yes |
| *Erigeron uniflorus* | *Erigeron* | *Asteraceae* | *Synandrae* | Magnoliopsida | Magnoliophyta | no | no | no+yes |
| *Eriophorum angustifolium* | *Eriophorum* | *Cyperaceae* | *Poales* | Liliopsida | Magnoliophyta | no | no | yes |
| *Eriophorum scheuchzeri* | *Eriophorum* | *Cyperaceae* | *Poales* | Liliopsida | Magnoliophyta | no | no | yes |
| *Eritrichium nanum* | *Eritrichium* | *Boraginaceae* | *Boraginales* | Magnoliopsida | Magnoliophyta | no | no | yes |
| *Erysimum cheiranthoides* | *Erysimum* | *Brassicaceae* | *Brassicales* | Magnoliopsida | Magnoliophyta | no | no | yes |
| *Euphrasia arctica* | *Euphrasia* | *Scrophulariaceae* | *Lamiales* | Magnoliopsida | Magnoliophyta | no | no | yes |
| *Euphrasia maximoviczii* | *Euphrasia* | *Scrophulariaceae* | *Lamiales* | Magnoliopsida | Magnoliophyta | no | no | yes |
| *Eurybia sibirica* | *Eurybia* | *Asteraceae* | *Synandrae* | Magnoliopsida | Magnoliophyta | no | no | yes |
| *Fallopia convolvulus* | *Fallopia* | *Polygonaceae* | *Caryophyllales* | Magnoliopsida | Magnoliophyta | no | yes | yes |
| *Festuca altaica* | *Festuca* | *Poaceae* | *Poales* | Liliopsida | Magnoliophyta | no | no | yes |
| *Filipendula camtschatica* | *Filipendula* | *Rosaceae* | *Rosales* | Magnoliopsida | Magnoliophyta | yes | yes | yes |
| *Fimbristylis dichotoma* | *Fimbristylis* | *Cyperaceae* | *Poales* | Liliopsida | Magnoliophyta | yes | yes | no+yes |
| *Fritillaria camtschatcensis* | *Fritillaria* | *Liliaceae* | *Liliales* | Liliopsida | Magnoliophyta | no | yes | yes |
| *Gagea serotina* | *Gagea* | *Liliaceae* | *Liliales* | Liliopsida | Magnoliophyta | no | no | yes |
| *Galeopsis bifida* | *Galeopsis* | *Lamiaceae* | *Lamiales* | Magnoliopsida | Magnoliophyta | no | no | yes |
| *Galium boreale* | *Galium* | *Rubiaceae* | *Gentianales* | Magnoliopsida | Magnoliophyta | yes | yes | yes |
| *Gentiana glauca* | *Gentiana* | *Gentianaceae* | *Gentianales* | Magnoliopsida | Magnoliophyta | no | no | yes |
| *Gentianella auriculata* | *Gentianella* | *Gentianaceae* | *Gentianales* | Magnoliopsida | Magnoliophyta | no | no | yes |
| *Geranium pratense* | *Geranium* | *Geraniaceae* | *Geraniales* | Magnoliopsida | Magnoliophyta | yes | no | yes |
| *Geum aleppicum* | *Geum* | *Rosaceae* | *Rosales* | Magnoliopsida | Magnoliophyta | no | yes | yes |
| *Geum macrophyllum* | *Geum* | *Rosaceae* | *Rosales* | Magnoliopsida | Magnoliophyta | no | no | yes |
| *Glyceria alnasteretum* | *Glyceria* | *Poaceae* | *Poales* | Liliopsida | Magnoliophyta | no | no | yes |
| *Gnaphalium uliginosum* | *Gnaphalium* | *Asteraceae* | *Synandrae* | Magnoliopsida | Magnoliophyta | no | no | yes |
| *Gymnocarpium dryopteris* | *Gymnocarpium* | *Woodsiaceae* | *Polypodiales* | Polypodiopsida | Polypodiophyta | no | no | yes |
| *Hackelia deflexa* | *Hackelia* | *Boraginaceae* | *Boraginales* | Magnoliopsida | Magnoliophyta | no | no | yes |
| *Harrimanella hypnoides* | *Harrimanella* | *Ericaceae* | *Ericales* | Magnoliopsida | Magnoliophyta | no | no | yes |
| *Hedysarum hedysaroides* | *Hedysarum* | *Fabaceae* | *Fabales* | Magnoliopsida | Magnoliophyta | no | no | yes |
| *Heracleum lanatum* | *Heracleum* | *Apiaceae* | *Apiales* | Magnoliopsida | Magnoliophyta | yes | yes | yes |
| *Hieracium triste* | *Hieracium* | *Asteraceae* | *Synandrae* | Magnoliopsida | Magnoliophyta | no | no | yes |
| *Hieracium umbellatum* | *Hieracium* | *Asteraceae* | *Synandrae* | Magnoliopsida | Magnoliophyta | no | yes | yes |
| *Hierochloe alpina* | *Hierochloa* | *Poaceae* | *Poales* | Liliopsida | Magnoliophyta | no | no | yes |
| *Huperzia selago* | *Huperzia* | *Lycopodiaceae* | *Lycopodiales* | Lycopodiopsida | Lycopodiophyta | no | yes | yes |
| *Impatiens noli* | *Impatiens* | *Balsaminaceae* | *Ericales* | Magnoliopsida | Magnoliophyta | no | no | yes |
| *Iris setosa* | *Iris* | *Iridaceae* | *Asparagales* | Liliopsida | Magnoliophyta | no | yes | yes |
| *Juncus alpinoarticulatus* | *Juncus* | *Juncaceae* | *Poales* | Liliopsida | Magnoliophyta | no | no | yes |
| *Juncus articulatus* | *Juncus* | *Juncaceae* | *Poales* | Liliopsida | Magnoliophyta | no | no | yes |
| *Juncus balticus* | *Juncus* | *Juncaceae* | *Poales* | Liliopsida | Magnoliophyta | no | no | yes |
| *Juncus biglumis* | *Juncus* | *Juncaceae* | *Poales* | Liliopsida | Magnoliophyta | no | no | yes |
| *Juncus bufonius* | *Juncus* | *Juncaceae* | *Poales* | Liliopsida | Magnoliophyta | no | no | yes |
| *Juncus filiformis* | *Juncus* | *Juncaceae* | *Poales* | Liliopsida | Magnoliophyta | no | no | yes |
| *Juniperus sibirica* | *Juniperus* | *Cupressaceae* | *Pinales* | Pinopsida | Pinophyta | no | no | yes |
| *Lagedium sibiricum* | *Lagedium* | *Asteraceae* | *Synandrae* | Magnoliopsida | Magnoliophyta | no | no | no+yes |
| *Lagotis glauca* | *Lagotis* | *Scrophulariaceae* | *Lamiales* | Magnoliopsida | Magnoliophyta | no | no | yes |
| *Ledum palustre* | *Ledum* | *Ericaceae* | *Ericales* | Magnoliopsida | Magnoliophyta | no | no | yes |
| *Leontodon taraxacoides* | *Leontodon* | *Asteraceae* | *Synandrae* | Magnoliopsida | Magnoliophyta | no | no | yes |
| *Leymus mollis* | *Leymus* | *Poaceae* | *Poales* | Liliopsida | Magnoliophyta | no | no | yes |
| *Lilium medeoloides* | *Lilium* | *Liliaceae* | *Liliales* | Liliopsida | Magnoliophyta | no | no | yes |
| *Loiseleuria procumbens* | *Loiseleuria* | *Ericaceae* | *Ericales* | Magnoliopsida | Magnoliophyta | no | no | yes |
| *Luzula arcuata* | *Luzula* | *Juncaceae* | *Poales* | Liliopsida | Magnoliophyta | no | no | yes |
| *Luzula campestris* | *Luzula* | *Juncaceae* | *Poales* | Liliopsida | Magnoliophyta | no | no | yes |
| *Luzula multiflora* | *Luzula* | *Juncaceae* | *Poales* | Liliopsida | Magnoliophyta | no | no | yes |
| *Lycopodium annotinum* | *Lycopodium* | *Lycopodiaceae* | *Lycopodiales* | Lycopodiopsida | Lycopodiophyta | no | no | yes |
| *Lycopodium clavatum* | *Lycopodium* | *Lycopodiaceae* | *Lycopodiales* | Lycopodiopsida | Lycopodiophyta | no | no | yes |
| *Lycopus uniflorus* | *Lycopus* | *Lamiaceae* | *Lamiales* | Magnoliopsida | Magnoliophyta | no | yes | yes |
| *Lysimachia europaea* | *Lysimachia* | *Primulaceae* | *Ericales* | Magnoliopsida | Magnoliophyta | no | yes | yes |
| *Maianthemum dilatatum* | *Maianthemum* | *Asparagaceae* | *Asparagales* | Liliopsida | Magnoliophyta | yes | yes | yes |
| *Malaxis monophyllos* | *Malaxis* | *Orchidaceae* | *Asparagales* | Liliopsida | Magnoliophyta | no | no | yes |
| *Matricaria chamomilla* | *Matricaria* | *Asteraceae* | *Synandrae* | Magnoliopsida | Magnoliophyta | no | no | yes |
| *Matricaria matricarioides* | *Matricaria* | *Asteraceae* | *Synandrae* | Magnoliopsida | Magnoliophyta | no | no | yes |
| *Matteuccia struthiopteris* | *Matteuccia* | *Onocleaceae* | *Polypodiales* | Polypodiopsida | Polypodiophyta | no | yes | yes |
| *Moehringia lateriflora* | *Moehringia* | *Caryophyllaceae* | *Caryophyllales* | Magnoliopsida | Magnoliophyta | yes | yes | yes |
| *Myosotis laxa ssp* | *Myosotis* | *Boraginaceae* | *Boraginales* | Magnoliopsida | Magnoliophyta | no | no | yes |
| *Ophioglossum thermale* | *Ophioglossum* | *Ophioglossaceae* | *Ophioglossales* | Psilotopsida | Polypodiophyta | no | yes | yes |
| *Ophioglossum vulgatum* | *Ophioglossum* | *Ophioglossaceae* | *Ophioglossales* | Psilotopsida | Polypodiophyta | yes | no | yes |
| *Oreopteris quelpaertensis* | *Oreopteris* | *Thelypteridaceae* | *Polypodiales* | Polypodiopsida | Polypodiophyta | no | yes | yes |
| *Orobanche ramosa* | *Orobanche* | *Orobanchaceae* | *Lamiales* | Magnoliopsida | Magnoliophyta | no | no | yes |
| *Orthilia secunda* | *Orthilia* | *Ericaceae* | *Ericales* | Magnoliopsida | Magnoliophyta | no | no | yes |
| *Oxyria digyna* | *Oxyria* | *Polygonaceae* | *Caryophyllales* | Magnoliopsida | Magnoliophyta | no | no | yes |
| *Oxytropis kamtschatica* | *Oxytropis* | *Fabaceae* | *Fabales* | Magnoliopsida | Magnoliophyta | no | no | yes |
| *Oxytropis revoluta* | *Oxytropis* | *Fabaceae* | *Fabales* | Magnoliopsida | Magnoliophyta | no | no | yes |
| *Papaver alboroseum* | *Papaver* | *Papaveraceae* | *Ranunculales* | Magnoliopsida | Magnoliophyta | no | no | yes |
| *Parasenecio hastiformis* | *Parasenecio* | *Asteraceae* | *Synandrae* | Magnoliopsida | Magnoliophyta | no | no | yes |
| *Parnassia palustris* | *Parnassia* | *Parnassiaceae* | *Celastrales* | Magnoliopsida | Magnoliophyta | no | no | yes |
| *Parrya nudicaulis* | *Parrya* | *Brassicaceae* | *Brassicales* | Magnoliopsida | Magnoliophyta | no | no | no+yes |
| *Pedicularis capitata* | *Pedicularis* | *Scrophulariaceae* | *Lamiales* | Magnoliopsida | Magnoliophyta | no | no | yes |
| *Pedicularis eriophora* | *Pedicularis* | *Scrophulariaceae* | *Lamiales* | Magnoliopsida | Magnoliophyta | no | no | yes |
| *Pedicularis labradorica* | *Pedicularis* | *Scrophulariaceae* | *Lamiales* | Magnoliopsida | Magnoliophyta | no | no | yes |
| *Pedicularis resupinata* | *Pedicularis* | *Scrophulariaceae* | *Lamiales* | Magnoliopsida | Magnoliophyta | yes | yes | yes |
| *Pedicularis verticillata* | *Pedicularis* | *Scrophulariaceae* | *Lamiales* | Magnoliopsida | Magnoliophyta | no | no | yes |
| *Persicaria vivipara* | *Persicaria* | *Polygonaceae* | *Caryophyllales* | Magnoliopsida | Magnoliophyta | no | no | yes |
| *Phalaris arundinacea* | *Phalaris* | *Poaceae* | *Poales* | Liliopsida | Magnoliophyta | no | no | yes |
| *Phegopteris connectilis* | *Phegopteris* | *Thelypteridaceae* | *Polypodiales* | Polypodiopsida | Polypodiophyta | no | yes | yes |
| *Phleum alpinum* | *Phleum* | *Poaceae* | *Poales* | Liliopsida | Magnoliophyta | no | no | yes |
| *Phleum pratense* | *Phleum* | *Poaceae* | *Poales* | Liliopsida | Magnoliophyta | no | no | yes |
| *Phyllodoce caerulea* | *Phyllodoce* | *Ericaceae* | *Ericales* | Magnoliopsida | Magnoliophyta | no | no | yes |
| *Picris hieracioides* | *Picris* | *Asteraceae* | *Synandrae* | Magnoliopsida | Magnoliophyta | yes | yes | yes |
| *Pinus pumila* | *Pinus* | *Pinaceae* | *Pinales* | Pinopsida | Pinophyta | no | no | yes |
| *Plantago asiatica* | *Plantago* | *Plantaginaceae* | *Lamiales* | Magnoliopsida | Magnoliophyta | no | yes | yes |
| *Plantago major* | *Plantago* | *Plantaginaceae* | *Lamiales* | Magnoliopsida | Magnoliophyta | no | no | yes |
| *Platanthera camtschatica* | *Platanthera* | *Orchidaceae* | *Asparagales* | Liliopsida | Magnoliophyta | no | no | yes |
| *Platanthera chorisiana* | *Platanthera* | *Orchidaceae* | *Asparagales* | Liliopsida | Magnoliophyta | no | no | yes |
| *Poa annua* | *Poa* | *Poaceae* | *Poales* | Liliopsida | Magnoliophyta | no | no | yes |
| *Poa lanata* | *Poa* | *Poaceae* | *Poales* | Liliopsida | Magnoliophyta | no | no | yes |
| *Poa leptocoma* | *Poa* | *Poaceae* | *Poales* | Liliopsida | Magnoliophyta | no | no | yes |
| *Poa platyantha* | *Poa* | *Poaceae* | *Poales* | Liliopsida | Magnoliophyta | no | no | yes |
| *Poa pratensis* | *Poa* | *Poaceae* | *Poales* | Liliopsida | Magnoliophyta | no | no | yes |
| *Polemonium boreale* | *Polemonium* | *Gentianaceae* | *Gentianales* | Magnoliopsida | Magnoliophyta | no | yes | yes |
| *Polemonium caeruleum* | *Polemoniceae* | *Gentianaceae* | *Gentianales* | Magnoliopsida | Magnoliophyta | no | no | yes |
| *Polygonum aviculare* | *Polygonum* | *Polygonaceae* | *Caryophyllales* | Magnoliopsida | Magnoliophyta | no | no | yes |
| *Polystichum braunii* | *Polystichum* | *Dryopteridaceae* | *Polypodiales* | Polypodiopsida | Polypodiophyta | no | no | yes |
| *Polystichum lonchitis* | *Polystichum* | *Dryopteridaceae* | *Polypodiales* | Polypodiopsida | Polypodiophyta | no | no | yes |
| *Populus suaveolens* | *Populus* | *Salicaceae* | *Malpighiales* | Magnoliopsida | Magnoliophyta | no | no | yes |
| *Populus tremula* | *Populus* | *Salicaceae* | *Malpighiales* | Magnoliopsida | Magnoliophyta | no | no | yes |
| *Potamogeton alpinus* | *Potamogeton* | *Potamogetonaceae* | *Alismatales* | Liliopsida | Magnoliophyta | no | no | yes |
| *Potentilla fragarioides* | *Potentilla* | *Rosaceae* | *Rosales* | Magnoliopsida | Magnoliophyta | yes | yes | yes |
| *Potentilla nivea* | *Potentilla* | *Rosaceae* | *Rosales* | Magnoliopsida | Magnoliophyta | no | no | yes |
| *Potentilla norvegica* | *Potentilla* | *Rosaceae* | *Rosales* | Magnoliopsida | Magnoliophyta | no | no | yes |
| *Potentilla uniflora* | *Potentilla* | *Rosaceae* | *Rosales* | Magnoliopsida | Magnoliophyta | no | no | yes |
| *Primula cuneifolia* | *Primula* | *Primulaceae* | *Ericales* | Magnoliopsida | Magnoliophyta | no | no | yes |
| *Ptarmica camtschatica* | *Ptarmica* | *Asteraceae* | *Synandrae* | Magnoliopsida | Magnoliophyta | no | yes | yes |
| *Pteridium aquilinum* | *Pteridium* | *Dennstaedtiaceae* | *Polypodiales* | Polypodiopsida | Polypodiophyta | yes | yes | yes |
| *Pyrola minor* | *Pyrola* | *Ericaceae* | *Ericales* | Magnoliopsida | Magnoliophyta | no | no | yes |
| *Ranunculus eschscholtzii* | *Ranunculus* | *Ranunculaceae* | *Ranunculales* | Magnoliopsida | Magnoliophyta | no | no | yes |
| *Ranunculus hyperboreus* | *Ranunculus* | *Ranunculaceae* | *Ranunculales* | Magnoliopsida | Magnoliophyta | no | no | yes |
| *Ranunculus japonicus* | *Ranunculus* | *Ranunculaceae* | *Ranunculales* | Magnoliopsida | Magnoliophyta | no | no | yes |
| *Ranunculus nivalis* | *Ranunculus* | *Ranunculaceae* | *Ranunculales* | Magnoliopsida | Magnoliophyta | no | no | yes |
| *Ranunculus pygmaeus* | *Ranunculus* | *Ranunculaceae* | *Ranunculales* | Magnoliopsida | Magnoliophyta | no | no | yes |
| *Ranunculus repens* | *Ranunculus* | *Ranunculaceae* | *Ranunculales* | Magnoliopsida | Magnoliophyta | no | yes | yes |
| *Rhodiola integrifolia* | *Rhodiola* | *Crassulaceae* | *Saxifragales* | Magnoliopsida | Magnoliophyta | no | no | yes |
| *Rhododendron aureum* | *Rhododendron* | *Ericaceae* | *Ericales* | Magnoliopsida | Magnoliophyta | no | no | yes |
| *Rorippa islandica* | *Rorippa* | *Brassicaceae* | *Brassicales* | Magnoliopsida | Magnoliophyta | no | no | yes |
| *Rorippa palustris* | *Rorippa* | *Brassicaceae* | *Brassicales* | Magnoliopsida | Magnoliophyta | no | no | yes |
| *Rosa davurica* | *Rosa* | *Rosaceae* | *Rosales* | Magnoliopsida | Magnoliophyta | no | yes | yes |
| *Rubus arcticus* | *Rubus* | *Rosaceae* | *Rosales* | Magnoliopsida | Magnoliophyta | no | no | yes |
| *Rubus idaeus* | *Rubus* | *Rosaceae* | *Rosales* | Magnoliopsida | Magnoliophyta | no | no | yes |
| *Rumex acetosa* | *Rumex* | *Polygonaceae* | *Caryophyllales* | Magnoliopsida | Magnoliophyta | no | no | yes |
| *Rumex aquaticus* | *Rumex* | *Polygonaceae* | *Caryophyllales* | Magnoliopsida | Magnoliophyta | no | yes | yes |
| *Sagina saginoides* | *Sagina* | *Caryophyllaceae* | *Caryophyllales* | Magnoliopsida | Magnoliophyta | no | no | yes |
| *Salix arctica* | *Salix* | *Salicaceae* | *Malpighiales* | Magnoliopsida | Magnoliophyta | no | no | yes |
| *Salix caprea* | *Salix* | *Salicaceae* | *Malpighiales* | Magnoliopsida | Magnoliophyta | no | no | yes |
| *Salix chamissonis* | *Salix* | *Salicaceae* | *Malpighiales* | Magnoliopsida | Magnoliophyta | no | no | yes |
| *Salix fuscescens* | *Salix* | *Salicaceae* | *Malpighiales* | Magnoliopsida | Magnoliophyta | no | no | yes |
| *Salix polaris* | *Salix* | *Salicaceae* | *Malpighiales* | Magnoliopsida | Magnoliophyta | no | no | yes |
| *Salix pulchra* | *Salix* | *Salicaceae* | *Malpighiales* | Magnoliopsida | Magnoliophyta | no | yes | yes |
| *Salix reticulata* | *Salix* | *Salicaceae* | *Malpighiales* | Magnoliopsida | Magnoliophyta | no | no | yes |
| *Salix tschuktschorum* | *Salix* | *Salicaceae* | *Malpighiales* | Magnoliopsida | Magnoliophyta | no | no | yes |
| *Salix udensis* | *Salix* | *Salicaceae* | *Malpighiales* | Magnoliopsida | Magnoliophyta | no | no | yes |
| *Sanguisorba officinalis* | *Sanguisorba* | *Rosaceae* | *Rosales* | Magnoliopsida | Magnoliophyta | no | yes | yes |
| *Saussurea nuda* | *Saussurea* | *Asteraceae* | *Synandrae* | Magnoliopsida | Magnoliophyta | no | no | yes |
| *Saxifraga bronchialis* | *Saxifraga* | *Saxifragaceae* | *Saxifragales* | Magnoliopsida | Magnoliophyta | no | no | yes |
| *Saxifraga merkii* | *Saxifraga* | *Saxifragaceae* | *Saxifragales* | Magnoliopsida | Magnoliophyta | no | no | yes |
| *Saxifraga nelsoniana* | *Saxifraga* | *Saxifragaceae* | *Saxifragales* | Magnoliopsida | Magnoliophyta | no | yes | yes |
| *Saxifraga rivularis* | *Saxifraga* | *Saxifragaceae* | *Saxifragales* | Magnoliopsida | Magnoliophyta | no | no | yes |
| *Schoenoplectus tabernaemontani* | *Schoenoplectus* | *Cyperaceae* | *Poales* | Liliopsida | Magnoliophyta | no | no | yes |
| *Scirpus microcarpus* | *Scirpus* | *Cyperaceae* | *Poales* | Liliopsida | Magnoliophyta | no | no | yes |
| *Sedum aizoon* | *Sedum* | *Crassulaceae* | *Saxifragales* | Magnoliopsida | Magnoliophyta | no | yes | yes |
| *Sedum telephium* | *Sedum* | *Crassulaceae* | *Saxifragales* | Magnoliopsida | Magnoliophyta | yes | yes | yes |
| *Selaginella rupestris* | *Selaginella* | *Selaginellaceae* | *Selaginellales* | Isoetopsida | Lycopodiophyta | no | no | yes |
| *Selaginella selaginoides* | *Selaginella* | *Selaginellaceae* | *Selaginellales* | Isoetopsida | Lycopodiophyta | no | no | no+yes |
| *Senecio cannabifolius* | *Senecio* | *Asteraceae* | *Synandrae* | Magnoliopsida | Magnoliophyta | yes | yes | yes |
| *Senecio resedifolius* | *Senecio* | *Asteraceae* | *Synandrae* | Magnoliopsida | Magnoliophyta | no | no | yes |
| *Sibbaldia procumbens* | *Sibbaldia* | *Rosaceae* | *Rosales* | Magnoliopsida | Magnoliophyta | no | no | yes |
| *Silene scouleri* | *Silene* | *Caryophyllaceae* | *Caryophyllales* | Magnoliopsida | Magnoliophyta | no | no | yes |
| *Solidago spiraeifolia* | *Solidago* | *Asteraceae* | *Synandrae* | Magnoliopsida | Magnoliophyta | no | yes | yes |
| *Sorbus sambucifolia* | *Sorbus* | *Rosaceae* | *Rosales* | Magnoliopsida | Magnoliophyta | no | no | yes |
| *Sparganium hyperboreum* | *Sparganium* | *Typhaceae* | *Poales* | Liliopsida | Magnoliophyta | no | no | yes |
| *Spiraea betulifolia* | *Spiraea* | *Rosaceae* | *Rosales* | Magnoliopsida | Magnoliophyta | no | yes | yes |
| *Spiranthes sinensis* | *Spiranthes* | *Orchidaceae* | *Asparagales* | Liliopsida | Magnoliophyta | no | no | yes |
| *Stellaria borealis* | *Stellaria* | *Caryophyllaceae* | *Caryophyllales* | Magnoliopsida | Magnoliophyta | no | no | yes |
| *Stellaria eschscholtziana* | *Stellaria* | *Caryophyllaceae* | *Caryophyllales* | Magnoliopsida | Magnoliophyta | no | no | yes |
| *Stellaria fenzlii* | *Stellaria* | *Caryophyllaceae* | *Caryophyllales* | Magnoliopsida | Magnoliophyta | no | no | yes |
| *Stellaria media* | *Stellaria* | *Caryophyllaceae* | *Caryophyllales* | Magnoliopsida | Magnoliophyta | no | no | yes |
| *Stellaria radians* | *Stellaria* | *Caryophyllaceae* | *Caryophyllales* | Magnoliopsida | Magnoliophyta | no | no | yes |
| *Streptopus amplexifolius* | *Streptopus* | *Asparagaceae* | *Asparagales* | Liliopsida | Magnoliophyta | no | yes | yes |
| *Taraxacum alaskanum* | *Taraxacum* | *Asteraceae* | *Synandrae* | Magnoliopsida | Magnoliophyta | no | no | yes |
| *Taraxacum ceratophorum* | *Taraxacum* | *Asteraceae* | *Synandrae* | Magnoliopsida | Magnoliophyta | no | no | yes |
| *Taraxacum lateritium* | *Taraxacum* | *Asteraceae* | *Synandrae* | Magnoliopsida | Magnoliophyta | no | no | yes |
| *Taraxacum natschikense* | *Taraxacum* | *Asteraceae* | *Synandrae* | Magnoliopsida | Magnoliophyta | no | no | yes |
| *Thalictrum minus* | *Thalictrum* | *Ranunculaceae* | *Ranunculales* | Magnoliopsida | Magnoliophyta | yes | yes | yes |
| *Tofieldia coccinea* | *Tofieldia* | *Colchicaceae* | *Liliales* | Liliopsida | Magnoliophyta | no | no | yes |
| *Trichophorum cespitosum* | *Trichophorum* | *Cyperaceae* | *Poales* | Liliopsida | Magnoliophyta | no | no | yes |
| *Trifolium repens* | *Trifolium* | *Fabaceae* | *Fabales* | Magnoliopsida | Magnoliophyta | no | no | yes |
| *Triglochin palustre* | *Triglochin* | *Juncaginaceae* | *Alismatales* | Liliopsida | Magnoliophyta | no | no | yes |
| *Trisetum flavescens* | *Trisetum* | *Poaceae* | *Poales* | Liliopsida | Magnoliophyta | no | no | yes |
| *Trisetum molle* | *Trisetum* | *Poaceae* | *Poales* | Liliopsida | Magnoliophyta | no | no | yes |
| *Trisetum spicatum* | *Trisetum* | *Poaceae* | *Poales* | Liliopsida | Magnoliophyta | no | no | yes |
| *Urtica dioica* | *Urtica* | *Urticaceae* | *Rosales* | Magnoliopsida | Magnoliophyta | no | yes | yes |
| *Vaccinium uliginosum* | *Vaccinium* | *Ericaceae* | *Ericales* | Magnoliopsida | Magnoliophyta | no | no | yes |
| *Vaccinium vitis* | *Vaccinium* | *Ericaceae* | *Ericales* | Magnoliopsida | Magnoliophyta | no | no | yes |
| *Valeriana capitata* | *Valeriana* | *Valerianaceae* | *Dipsacales* | Magnoliopsida | Magnoliophyta | no | no | yes |
| *Veratrum albiflorum* | *Veratrum* | *Melanthiaceae* | *Melanthiales* | Liliopsida | Magnoliophyta | no | no | yes |
| *Veratrum album* | *Veratrum* | *Colchicaceae* | *Liliales* | Liliopsida | Magnoliophyta | no | yes | yes |
| *Veronica americana* | *Veronica* | *Scrophulariaceae* | *Lamiales* | Magnoliopsida | Magnoliophyta | no | no | yes |
| *Veronica grandiflora* | *Veronica* | *Scrophulariaceae* | *Lamiales* | Magnoliopsida | Magnoliophyta | no | no | yes |
| *Viola biflora* | *Viola* | *Violaceae* | *Malpighiales* | Magnoliopsida | Magnoliophyta | no | no | yes |
| *Viola epipsila* | *Viola* | *Violaceae* | *Malpighiales* | Magnoliopsida | Magnoliophyta | no | no | yes |
| *Viola langsdorfii* | *Viola* | *Violaceae* | *Malpighiales* | Magnoliopsida | Magnoliophyta | no | yes | yes |
| *Viola sacchalinensis* | *Viola* | *Violaceae* | *Malpighiales* | Magnoliopsida | Magnoliophyta | no | no | yes |
| *Viola selkirkii* | *Viola* | *Violaceae* | *Malpighiales* | Magnoliopsida | Magnoliophyta | yes | yes | yes |
| *Woodsia glabella* | *Woodsia* | *Woodsiaceae* | *Polypodiales* | Polypodiopsida | Polypodiophyta | no | no | yes |
| *Woodsia ilvensis* | *Woodsia* | *Woodsiaceae* | *Polypodiales* | Polypodiopsida | Polypodiophyta | no | no | yes |

T – the studied catena, TB – thermal and non-heated habitats near the catena (long-term research by A.V. Zavadskaya and M.V. Prozorova) ^17^. The Geysernaya River valley’s plant species list compiled by L.I. Rassokhina: Ya – published data ^18^, R – unpublished Rassokhina’s long-term research data supplementing  ^19^, from funds of the Kronotsky Nature Reserve

## *Table S 15. Overrepresentation and underrepresentation of plant taxa in comparison with the data on 292 species occurring in the Valley of Geysers*

| Group | Taxon | Taxon | N of species in catena and its surrounding territory | | | | | Group | N of all species in the Valley of Geysers | | | | |
| --- | --- | --- | --- | --- | --- | --- | --- | --- | --- | --- | --- | --- | --- |
|  |  |  | Given  taxon | Given  group | Given taxon  in the given group | Pover | Punder |  | Given  taxon | Given  group | Given taxon  in the given group | Pover | Punder |
| B1 | Family | *Juncaceae* | 9 | 22 | 0 | 1.0 | 0.492 | B1 | NA | NA | NA | NA | NA |
| B1 | Family | *Valerianaceae* | 1 | 22 | 0 | 1.0 | 0.925 | B1 | NA | NA | NA | NA | NA |
| B1 | Family | *Dennstaedtiaceae* | 1 | 22 | 0 | 1.0 | 0.925 | B1 | NA | NA | NA | NA | NA |
| B1 | Family | *Cupressaceae* | 1 | 22 | 0 | 1.0 | 0.925 | B1 | NA | NA | NA | NA | NA |
| B1 | Family | *Lamiaceae* | 2 | 22 | 0 | 1.0 | 0.856 | B1 | NA | NA | NA | NA | NA |
| B1 | Family | *Dryopteridaceae* | 3 | 22 | 1 | 0.209 | 0.985 | B1 | NA | NA | NA | NA | NA |
| B1 | Family | *Brassicaceae* | 13 | 22 | 1 | 0.644 | 0.747 | B1 | NA | NA | NA | NA | NA |
| B1 | Family | *Pinaceae* | 1 | 22 | 0 | 1.0 | 0.925 | B1 | NA | NA | NA | NA | NA |
| B1 | Family | *Geraniaceae* | 1 | 22 | 0 | 1.0 | 0.925 | B1 | NA | NA | NA | NA | NA |
| B1 | Family | *Boraginaceae* | 4 | 22 | 0 | 1.0 | 0.731 | B1 | NA | NA | NA | NA | NA |
| B1 | Family | *Plantaginaceae* | 2 | 22 | 0 | 1.0 | 0.856 | B1 | NA | NA | NA | NA | NA |
| B1 | Family | *Cornaceae* | 1 | 22 | 0 | 1.0 | 0.925 | B1 | NA | NA | NA | NA | NA |
| B1 | Family | *Crassulaceae* | 3 | 22 | 0 | 1.0 | 0.791 | B1 | NA | NA | NA | NA | NA |
| B1 | Family | *Gentianaceae* | 4 | 22 | 0 | 1.0 | 0.731 | B1 | NA | NA | NA | NA | NA |
| B1 | Family | *Caryophyllaceae* | 10 | 22 | 0 | 1.0 | 0.454 | B1 | NA | NA | NA | NA | NA |
| B1 | Family | *Parnassiaceae* | 1 | 22 | 0 | 1.0 | 0.925 | B1 | NA | NA | NA | NA | NA |
| B1 | Family | *Campanulaceae* | 1 | 22 | 0 | 1.0 | 0.925 | B1 | NA | NA | NA | NA | NA |
| B1 | Family | *Apiaceae* | 5 | 22 | 1 | 0.324 | 0.953 | B1 | NA | NA | NA | NA | NA |
| B1 | Family | *Aspleniaceae* | 2 | 22 | 0 | 1.0 | 0.856 | B1 | NA | NA | NA | NA | NA |
| B1 | Family | *Ophioglossaceae* | 5 | 22 | 1 | 0.324 | 0.953 | B1 | NA | NA | NA | NA | NA |
| B1 | Family | *Thelypteridaceae* | 2 | 22 | 2 | 0.005 | 1.0 | B1 | NA | NA | NA | NA | NA |
| B1 | Family | *Selaginellaceae* | 2 | 22 | 0 | 1.0 | 0.856 | B1 | NA | NA | NA | NA | NA |
| B1 | Family | *Urticaceae* | 1 | 22 | 0 | 1.0 | 0.925 | B1 | NA | NA | NA | NA | NA |
| B1 | Family | *Orobanchaceae* | 1 | 22 | 0 | 1.0 | 0.925 | B1 | NA | NA | NA | NA | NA |
| B1 | Family | *Saxifragaceae* | 4 | 22 | 1 | 0.269 | 0.971 | B1 | NA | NA | NA | NA | NA |
| B1 | Family | *Balsaminaceae* | 1 | 22 | 0 | 1.0 | 0.925 | B1 | NA | NA | NA | NA | NA |
| B1 | Family | *Poaceae* | 30 | 22 | 0 | 1.0 | 0.085 | B1 | NA | NA | NA | NA | NA |
| B1 | Family | *Woodsiaceae* | 6 | 22 | 0 | 1.0 | 0.624 | B1 | NA | NA | NA | NA | NA |
| B1 | Family | *Onagraceae* | 7 | 22 | 0 | 1.0 | 0.577 | B1 | NA | NA | NA | NA | NA |
| B1 | Family | *Orchidaceae* | 6 | 22 | 0 | 1.0 | 0.624 | B1 | NA | NA | NA | NA | NA |
| B1 | Family | *Juncaginaceae* | 1 | 22 | 0 | 1.0 | 0.925 | B1 | NA | NA | NA | NA | NA |
| B1 | Family | *Rosaceae* | 18 | 22 | 1 | 0.764 | 0.603 | B1 | NA | NA | NA | NA | NA |
| B1 | Family | *Primulaceae* | 4 | 22 | 0 | 1.0 | 0.731 | B1 | NA | NA | NA | NA | NA |
| B1 | Family | *Ranunculaceae* | 11 | 22 | 0 | 1.0 | 0.419 | B1 | NA | NA | NA | NA | NA |
| B1 | Family | *Fabaceae* | 5 | 22 | 0 | 1.0 | 0.676 | B1 | NA | NA | NA | NA | NA |
| B1 | Family | *Rubiaceae* | 1 | 22 | 0 | 1.0 | 0.925 | B1 | NA | NA | NA | NA | NA |
| B1 | Family | *Polygonaceae* | 8 | 22 | 1 | 0.467 | 0.886 | B1 | NA | NA | NA | NA | NA |
| B1 | Family | *Equisetaceae* | 2 | 22 | 2 | 0.005 | 1.0 | B1 | NA | NA | NA | NA | NA |
| B1 | Family | *Colchicaceae* | 2 | 22 | 1 | 0.144 | 0.995 | B1 | NA | NA | NA | NA | NA |
| B1 | Family | *Onocleaceae* | 1 | 22 | 1 | 0.075 | 1.0 | B1 | NA | NA | NA | NA | NA |
| B1 | Family | *Asteraceae* | 31 | 22 | 2 | 0.702 | 0.584 | B1 | NA | NA | NA | NA | NA |
| B1 | Family | *Liliaceae* | 3 | 22 | 1 | 0.209 | 0.985 | B1 | NA | NA | NA | NA | NA |
| B1 | Family | *Callitrichaceae* | 1 | 22 | 0 | 1.0 | 0.925 | B1 | NA | NA | NA | NA | NA |
| B1 | Family | *Salicaceae* | 11 | 22 | 1 | 0.581 | 0.805 | B1 | NA | NA | NA | NA | NA |
| B1 | Family | *Papaveraceae* | 1 | 22 | 0 | 1.0 | 0.925 | B1 | NA | NA | NA | NA | NA |
| B1 | Family | *Diapensiaceae* | 1 | 22 | 0 | 1.0 | 0.925 | B1 | NA | NA | NA | NA | NA |
| B1 | Family | *Potamogetonaceae* | 1 | 22 | 0 | 1.0 | 0.925 | B1 | NA | NA | NA | NA | NA |
| B1 | Family | *Betulaceae* | 3 | 22 | 2 | 0.015 | 1.0 | B1 | NA | NA | NA | NA | NA |
| B1 | Family | *Ericaceae* | 12 | 22 | 0 | 1.0 | 0.386 | B1 | NA | NA | NA | NA | NA |
| B1 | Family | *Asparagaceae* | 2 | 22 | 1 | 0.144 | 0.995 | B1 | NA | NA | NA | NA | NA |
| B1 | Family | *Lycopodiaceae* | 4 | 22 | 1 | 0.269 | 0.971 | B1 | NA | NA | NA | NA | NA |
| B1 | Family | *Typhaceae* | 1 | 22 | 0 | 1.0 | 0.925 | B1 | NA | NA | NA | NA | NA |
| B1 | Family | *Cyperaceae* | 18 | 22 | 0 | 1.0 | 0.236 | B1 | NA | NA | NA | NA | NA |
| B1 | Family | *Scrophulariaceae* | 11 | 22 | 0 | 1.0 | 0.419 | B1 | NA | NA | NA | NA | NA |
| B1 | Family | *Empetraceae* | 1 | 22 | 0 | 1.0 | 0.925 | B1 | NA | NA | NA | NA | NA |
| B1 | Family | *Alliaceae* | 2 | 22 | 0 | 1.0 | 0.856 | B1 | NA | NA | NA | NA | NA |
| B1 | Family | *Adiantaceae* | 1 | 22 | 0 | 1.0 | 0.925 | B1 | NA | NA | NA | NA | NA |
| B1 | Family | *Violaceae* | 5 | 22 | 1 | 0.324 | 0.953 | B1 | NA | NA | NA | NA | NA |
| B1 | Family | *Melanthiaceae* | 1 | 22 | 0 | 1.0 | 0.925 | B1 | NA | NA | NA | NA | NA |
| B1 | Family | *Iridaceae* | 1 | 22 | 0 | 1.0 | 0.925 | B1 | NA | NA | NA | NA | NA |
| Tall | Family | *Juncaceae* | 9 | 49 | 0 | 1.0 | 0.189 | Tall | 9 | 167 | 8 | 0.046 | 0.994 |
| Tall | Family | *Valerianaceae* | 1 | 49 | 0 | 1.0 | 0.833 | Tall | 1 | 167 | 0 | 1.000 | 0.432 |
| Tall | Family | *Dennstaedtiaceae* | 1 | 49 | 1 | 0.167 | 1.0 | Tall | 1 | 167 | 1 | 0.568 | 1.000 |
| Tall | Family | *Cupressaceae* | 1 | 49 | 0 | 1.0 | 0.833 | Tall | 1 | 167 | 1 | 0.568 | 1.000 |
| Tall | Family | *Lamiaceae* | 2 | 49 | 1 | 0.306 | 0.973 | Tall | 2 | 167 | 1 | 0.814 | 0.678 |
| Tall | Family | *Dryopteridaceae* | 3 | 49 | 0 | 1.0 | 0.578 | Tall | 3 | 167 | 2 | 0.602 | 0.818 |
| Tall | Family | *Brassicaceae* | 13 | 49 | 1 | 0.912 | 0.330 | Tall | 13 | 167 | 8 | 0.479 | 0.735 |
| Tall | Family | *Pinaceae* | 1 | 49 | 0 | 1.0 | 0.833 | Tall | 1 | 167 | 1 | 0.568 | 1.000 |
| Tall | Family | *Geraniaceae* | 1 | 49 | 1 | 0.167 | 1.0 | Tall | 1 | 167 | 1 | 0.568 | 1.000 |
| Tall | Family | *Boraginaceae* | 4 | 49 | 0 | 1.0 | 0.480 | Tall | 4 | 167 | 4 | 0.102 | 1.000 |
| Tall | Family | *Plantaginaceae* | 2 | 49 | 1 | 0.306 | 0.973 | Tall | 2 | 167 | 2 | 0.322 | 1.000 |
| Tall | Family | *Cornaceae* | 1 | 49 | 0 | 1.0 | 0.833 | Tall | 1 | 167 | 0 | 1.000 | 0.432 |
| Tall | Family | *Crassulaceae* | 3 | 49 | 2 | 0.073 | 0.996 | Tall | 3 | 167 | 3 | 0.182 | 1.000 |
| Tall | Family | *Gentianaceae* | 4 | 49 | 1 | 0.520 | 0.869 | Tall | 4 | 167 | 2 | 0.784 | 0.580 |
| Tall | Family | *Caryophyllaceae* | 10 | 49 | 1 | 0.843 | 0.481 | Tall | 10 | 167 | 9 | 0.028 | 0.997 |
| Tall | Family | *Parnassiaceae* | 1 | 49 | 0 | 1.0 | 0.833 | Tall | 1 | 167 | 1 | 0.568 | 1.000 |
| Tall | Family | *Campanulaceae* | 1 | 49 | 0 | 1.0 | 0.833 | Tall | 1 | 167 | 0 | 1.000 | 0.432 |
| Tall | Family | *Apiaceae* | 5 | 49 | 2 | 0.195 | 0.966 | Tall | 5 | 167 | 3 | 0.627 | 0.718 |
| Tall | Family | *Aspleniaceae* | 2 | 49 | 1 | 0.306 | 0.973 | Tall | 2 | 167 | 2 | 0.322 | 1.000 |
| Tall | Family | *Ophioglossaceae* | 5 | 49 | 2 | 0.195 | 0.966 | Tall | 5 | 167 | 4 | 0.282 | 0.942 |
| Tall | Family | *Thelypteridaceae* | 2 | 49 | 0 | 1.0 | 0.694 | Tall | 2 | 167 | 0 | 1.000 | 0.186 |
| Tall | Family | *Selaginellaceae* | 2 | 49 | 0 | 1.0 | 0.694 | Tall | 2 | 167 | 0 | 1.000 | 0.186 |
| Tall | Family | *Urticaceae* | 1 | 49 | 1 | 0.167 | 1.0 | Tall | 1 | 167 | 1 | 0.568 | 1.000 |
| Tall | Family | *Orobanchaceae* | 1 | 49 | 0 | 1.0 | 0.833 | Tall | 1 | 167 | 1 | 0.568 | 1.000 |
| Tall | Family | *Saxifragaceae* | 4 | 49 | 0 | 1.0 | 0.480 | Tall | 4 | 167 | 0 | 1.000 | 0.034 |
| Tall | Family | *Balsaminaceae* | 1 | 49 | 0 | 1.0 | 0.833 | Tall | 1 | 167 | 1 | 0.568 | 1.000 |
| Tall | Family | *Poaceae* | 30 | 49 | 4 | 0.775 | 0.416 | Tall | 30 | 167 | 17 | 0.586 | 0.568 |
| Tall | Family | *Woodsiaceae* | 6 | 49 | 0 | 1.0 | 0.331 | Tall | 6 | 167 | 1 | 0.994 | 0.056 |
| Tall | Family | *Onagraceae* | 7 | 49 | 2 | 0.330 | 0.907 | Tall | 7 | 167 | 6 | 0.118 | 0.982 |
| Tall | Family | *Orchidaceae* | 6 | 49 | 1 | 0.669 | 0.737 | Tall | 6 | 167 | 5 | 0.184 | 0.968 |
| Tall | Family | *Juncaginaceae* | 1 | 49 | 0 | 1.0 | 0.833 | Tall | 1 | 167 | 1 | 0.568 | 1.000 |
| Tall | Family | *Rosaceae* | 18 | 49 | 6 | 0.059 | 0.983 | Tall | 18 | 167 | 9 | 0.802 | 0.358 |
| Tall | Family | *Primulaceae* | 4 | 49 | 1 | 0.520 | 0.869 | Tall | 4 | 167 | 2 | 0.784 | 0.580 |
| Tall | Family | *Ranunculaceae* | 11 | 49 | 3 | 0.271 | 0.908 | Tall | 11 | 167 | 8 | 0.221 | 0.922 |
| Tall | Family | *Fabaceae* | 5 | 49 | 0 | 1.0 | 0.399 | Tall | 5 | 167 | 3 | 0.627 | 0.718 |
| Tall | Family | *Rubiaceae* | 1 | 49 | 1 | 0.167 | 1.0 | Tall | 1 | 167 | 1 | 0.568 | 1.000 |
| Tall | Family | *Polygonaceae* | 8 | 49 | 1 | 0.772 | 0.604 | Tall | 8 | 167 | 5 | 0.519 | 0.751 |
| Tall | Family | *Equisetaceae* | 2 | 49 | 0 | 1.0 | 0.694 | Tall | 2 | 167 | 0 | 1.000 | 0.186 |
| Tall | Family | *Colchicaceae* | 2 | 49 | 0 | 1.0 | 0.694 | Tall | 2 | 167 | 0 | 1.000 | 0.186 |
| Tall | Family | *Onocleaceae* | 1 | 49 | 0 | 1.0 | 0.833 | Tall | 1 | 167 | 0 | 1.000 | 0.432 |
| Tall | Family | *Asteraceae* | 31 | 49 | 6 | 0.415 | 0.759 | Tall | 31 | 167 | 21 | 0.133 | 0.934 |
| Tall | Family | *Liliaceae* | 3 | 49 | 0 | 1.0 | 0.578 | Tall | 3 | 167 | 1 | 0.920 | 0.398 |
| Tall | Family | *Callitrichaceae* | 1 | 49 | 0 | 1.0 | 0.833 | Tall | 1 | 167 | 0 | 1.000 | 0.432 |
| Tall | Family | *Salicaceae* | 11 | 49 | 0 | 1.0 | 0.130 | Tall | 11 | 167 | 5 | 0.861 | 0.318 |
| Tall | Family | *Papaveraceae* | 1 | 49 | 0 | 1.0 | 0.833 | Tall | 1 | 167 | 0 | 1.000 | 0.432 |
| Tall | Family | *Diapensiaceae* | 1 | 49 | 0 | 1.0 | 0.833 | Tall | 1 | 167 | 0 | 1.000 | 0.432 |
| Tall | Family | *Potamogetonaceae* | 1 | 49 | 0 | 1.0 | 0.833 | Tall | 1 | 167 | 0 | 1.000 | 0.432 |
| Tall | Family | *Betulaceae* | 3 | 49 | 0 | 1.0 | 0.578 | Tall | 3 | 167 | 0 | 1.000 | 0.080 |
| Tall | Family | *Ericaceae* | 12 | 49 | 0 | 1.0 | 0.107 | Tall | 12 | 167 | 2 | 0.999 | 0.005 |
| Tall | Family | *Asparagaceae* | 2 | 49 | 1 | 0.306 | 0.973 | Tall | 2 | 167 | 1 | 0.814 | 0.678 |
| Tall | Family | *Lycopodiaceae* | 4 | 49 | 0 | 1.0 | 0.480 | Tall | 4 | 167 | 1 | 0.966 | 0.216 |
| Tall | Family | *Typhaceae* | 1 | 49 | 0 | 1.0 | 0.833 | Tall | 1 | 167 | 1 | 0.568 | 1.000 |
| Tall | Family | *Cyperaceae* | 18 | 49 | 3 | 0.604 | 0.649 | Tall | 18 | 167 | 10 | 0.642 | 0.550 |
| Tall | Family | *Scrophulariaceae* | 11 | 49 | 1 | 0.870 | 0.427 | Tall | 11 | 167 | 4 | 0.956 | 0.139 |
| Tall | Family | *Empetraceae* | 1 | 49 | 0 | 1.0 | 0.833 | Tall | 1 | 167 | 0 | 1.000 | 0.432 |
| Tall | Family | *Alliaceae* | 2 | 49 | 2 | 0.027 | 1.0 | Tall | 2 | 167 | 2 | 0.322 | 1.000 |
| Tall | Family | *Adiantaceae* | 1 | 49 | 0 | 1.0 | 0.833 | Tall | 1 | 167 | 0 | 1.000 | 0.432 |
| Tall | Family | *Violaceae* | 5 | 49 | 1 | 0.601 | 0.805 | Tall | 5 | 167 | 3 | 0.627 | 0.718 |
| Tall | Family | *Melanthiaceae* | 1 | 49 | 0 | 1.0 | 0.833 | Tall | 1 | 167 | 1 | 0.568 | 1.000 |
| Tall | Family | *Iridaceae* | 1 | 49 | 1 | 0.167 | 1.0 | Tall | 1 | 167 | 1 | 0.568 | 1.000 |
| Tf | Family | *Juncaceae* | 9 | 33 | 0 | 1.0 | 0.337 | Tf | 9 | 142 | 6 | 0.218 | 0.929 |
| Tf | Family | *Valerianaceae* | 1 | 33 | 0 | 1.0 | 0.888 | Tf | 1 | 142 | 0 | 1.000 | 0.517 |
| Tf | Family | *Dennstaedtiaceae* | 1 | 33 | 1 | 0.112 | 1.0 | Tf | 1 | 142 | 1 | 0.483 | 1.000 |
| Tf | Family | *Cupressaceae* | 1 | 33 | 0 | 1.0 | 0.888 | Tf | 1 | 142 | 1 | 0.483 | 1.000 |
| Tf | Family | *Lamiaceae* | 2 | 33 | 1 | 0.212 | 0.988 | Tf | 2 | 142 | 1 | 0.734 | 0.768 |
| Tf | Family | *Dryopteridaceae* | 3 | 33 | 0 | 1.0 | 0.699 | Tf | 3 | 142 | 2 | 0.474 | 0.889 |
| Tf | Family | *Brassicaceae* | 13 | 33 | 1 | 0.795 | 0.560 | Tf | 13 | 142 | 8 | 0.244 | 0.897 |
| Tf | Family | *Pinaceae* | 1 | 33 | 0 | 1.0 | 0.888 | Tf | 1 | 142 | 1 | 0.483 | 1.000 |
| Tf | Family | *Geraniaceae* | 1 | 33 | 1 | 0.112 | 1.0 | Tf | 1 | 142 | 1 | 0.483 | 1.000 |
| Tf | Family | *Boraginaceae* | 4 | 33 | 0 | 1.0 | 0.620 | Tf | 4 | 142 | 3 | 0.286 | 0.947 |
| Tf | Family | *Plantaginaceae* | 2 | 33 | 0 | 1.0 | 0.788 | Tf | 2 | 142 | 1 | 0.734 | 0.768 |
| Tf | Family | *Cornaceae* | 1 | 33 | 0 | 1.0 | 0.888 | Tf | 1 | 142 | 0 | 1.000 | 0.517 |
| Tf | Family | *Crassulaceae* | 3 | 33 | 1 | 0.301 | 0.966 | Tf | 3 | 142 | 2 | 0.474 | 0.889 |
| Tf | Family | *Gentianaceae* | 4 | 33 | 1 | 0.380 | 0.936 | Tf | 4 | 142 | 2 | 0.663 | 0.714 |
| Tf | Family | *Caryophyllaceae* | 10 | 33 | 0 | 1.0 | 0.298 | Tf | 10 | 142 | 8 | 0.041 | 0.993 |
| Tf | Family | *Parnassiaceae* | 1 | 33 | 0 | 1.0 | 0.888 | Tf | 1 | 142 | 1 | 0.483 | 1.000 |
| Tf | Family | *Campanulaceae* | 1 | 33 | 0 | 1.0 | 0.888 | Tf | 1 | 142 | 0 | 1.000 | 0.517 |
| Tf | Family | *Apiaceae* | 5 | 33 | 1 | 0.451 | 0.901 | Tf | 5 | 142 | 2 | 0.793 | 0.532 |
| Tf | Family | *Aspleniaceae* | 2 | 33 | 1 | 0.212 | 0.988 | Tf | 2 | 142 | 2 | 0.232 | 1.000 |
| Tf | Family | *Ophioglossaceae* | 5 | 33 | 1 | 0.451 | 0.901 | Tf | 5 | 142 | 3 | 0.468 | 0.835 |
| Tf | Family | *Thelypteridaceae* | 2 | 33 | 0 | 1.0 | 0.788 | Tf | 2 | 142 | 0 | 1.000 | 0.266 |
| Tf | Family | *Selaginellaceae* | 2 | 33 | 0 | 1.0 | 0.788 | Tf | 2 | 142 | 0 | 1.000 | 0.266 |
| Tf | Family | *Urticaceae* | 1 | 33 | 1 | 0.112 | 1.0 | Tf | 1 | 142 | 1 | 0.483 | 1.000 |
| Tf | Family | *Orobanchaceae* | 1 | 33 | 0 | 1.0 | 0.888 | Tf | 1 | 142 | 1 | 0.483 | 1.000 |
| Tf | Family | *Saxifragaceae* | 4 | 33 | 0 | 1.0 | 0.620 | Tf | 4 | 142 | 0 | 1.000 | 0.070 |
| Tf | Family | *Balsaminaceae* | 1 | 33 | 0 | 1.0 | 0.888 | Tf | 1 | 142 | 1 | 0.483 | 1.000 |
| Tf | Family | *Poaceae* | 30 | 33 | 1 | 0.977 | 0.121 | Tf | 30 | 142 | 13 | 0.778 | 0.352 |
| Tf | Family | *Woodsiaceae* | 6 | 33 | 0 | 1.0 | 0.486 | Tf | 6 | 142 | 1 | 0.982 | 0.124 |
| Tf | Family | *Onagraceae* | 7 | 33 | 2 | 0.179 | 0.967 | Tf | 7 | 142 | 6 | 0.050 | 0.994 |
| Tf | Family | *Orchidaceae* | 6 | 33 | 1 | 0.514 | 0.862 | Tf | 6 | 142 | 4 | 0.311 | 0.908 |
| Tf | Family | *Juncaginaceae* | 1 | 33 | 0 | 1.0 | 0.888 | Tf | 1 | 142 | 1 | 0.483 | 1.000 |
| Tf | Family | *Rosaceae* | 18 | 33 | 2 | 0.624 | 0.672 | Tf | 18 | 142 | 5 | 0.981 | 0.059 |
| Tf | Family | *Primulaceae* | 4 | 33 | 1 | 0.380 | 0.936 | Tf | 4 | 142 | 2 | 0.663 | 0.714 |
| Tf | Family | *Ranunculaceae* | 11 | 33 | 3 | 0.114 | 0.975 | Tf | 11 | 142 | 8 | 0.089 | 0.977 |
| Tf | Family | *Fabaceae* | 5 | 33 | 0 | 1.0 | 0.549 | Tf | 5 | 142 | 3 | 0.468 | 0.835 |
| Tf | Family | *Rubiaceae* | 1 | 33 | 1 | 0.112 | 1.0 | Tf | 1 | 142 | 1 | 0.483 | 1.000 |
| Tf | Family | *Polygonaceae* | 8 | 33 | 1 | 0.619 | 0.777 | Tf | 8 | 142 | 5 | 0.324 | 0.880 |
| Tf | Family | *Equisetaceae* | 2 | 33 | 0 | 1.0 | 0.788 | Tf | 2 | 142 | 0 | 1.000 | 0.266 |
| Tf | Family | *Colchicaceae* | 2 | 33 | 0 | 1.0 | 0.788 | Tf | 2 | 142 | 0 | 1.000 | 0.266 |
| Tf | Family | *Onocleaceae* | 1 | 33 | 0 | 1.0 | 0.888 | Tf | 1 | 142 | 0 | 1.000 | 0.517 |
| Tf | Family | *Asteraceae* | 31 | 33 | 6 | 0.115 | 0.957 | Tf | 31 | 142 | 20 | 0.042 | 0.983 |
| Tf | Family | *Liliaceae* | 3 | 33 | 0 | 1.0 | 0.699 | Tf | 3 | 142 | 1 | 0.863 | 0.526 |
| Tf | Family | *Callitrichaceae* | 1 | 33 | 0 | 1.0 | 0.888 | Tf | 1 | 142 | 0 | 1.000 | 0.517 |
| Tf | Family | *Salicaceae* | 11 | 33 | 0 | 1.0 | 0.263 | Tf | 11 | 142 | 5 | 0.689 | 0.547 |
| Tf | Family | *Papaveraceae* | 1 | 33 | 0 | 1.0 | 0.888 | Tf | 1 | 142 | 0 | 1.000 | 0.517 |
| Tf | Family | *Diapensiaceae* | 1 | 33 | 0 | 1.0 | 0.888 | Tf | 1 | 142 | 0 | 1.000 | 0.517 |
| Tf | Family | *Potamogetonaceae* | 1 | 33 | 0 | 1.0 | 0.888 | Tf | 1 | 142 | 0 | 1.000 | 0.517 |
| Tf | Family | *Betulaceae* | 3 | 33 | 0 | 1.0 | 0.699 | Tf | 3 | 142 | 0 | 1.000 | 0.137 |
| Tf | Family | *Ericaceae* | 12 | 33 | 0 | 1.0 | 0.233 | Tf | 12 | 142 | 2 | 0.996 | 0.023 |
| Tf | Family | *Asparagaceae* | 2 | 33 | 1 | 0.212 | 0.988 | Tf | 2 | 142 | 1 | 0.734 | 0.768 |
| Tf | Family | *Lycopodiaceae* | 4 | 33 | 0 | 1.0 | 0.620 | Tf | 4 | 142 | 1 | 0.930 | 0.337 |
| Tf | Family | *Typhaceae* | 1 | 33 | 0 | 1.0 | 0.888 | Tf | 1 | 142 | 1 | 0.483 | 1.000 |
| Tf | Family | *Cyperaceae* | 18 | 33 | 0 | 1.0 | 0.110 | Tf | 18 | 142 | 4 | 0.995 | 0.019 |
| Tf | Family | *Scrophulariaceae* | 11 | 33 | 1 | 0.737 | 0.644 | Tf | 11 | 142 | 4 | 0.868 | 0.311 |
| Tf | Family | *Empetraceae* | 1 | 33 | 0 | 1.0 | 0.888 | Tf | 1 | 142 | 0 | 1.000 | 0.517 |
| Tf | Family | *Alliaceae* | 2 | 33 | 1 | 0.212 | 0.988 | Tf | 2 | 142 | 1 | 0.734 | 0.768 |
| Tf | Family | *Adiantaceae* | 1 | 33 | 0 | 1.0 | 0.888 | Tf | 1 | 142 | 0 | 1.000 | 0.517 |
| Tf | Family | *Violaceae* | 5 | 33 | 1 | 0.451 | 0.901 | Tf | 5 | 142 | 3 | 0.468 | 0.835 |
| Tf | Family | *Melanthiaceae* | 1 | 33 | 0 | 1.0 | 0.888 | Tf | 1 | 142 | 1 | 0.483 | 1.000 |
| Tf | Family | *Iridaceae* | 1 | 33 | 1 | 0.112 | 1.0 | Tf | 1 | 142 | 1 | 0.483 | 1.000 |
| B2 | Family | *Juncaceae* | 9 | 223 | 9 | 0.080 | 1.0 | Ball | 9 | 127 | 1 | 0.994 | 0.046 |
| B2 | Family | *Valerianaceae* | 1 | 223 | 1 | 0.759 | 1.0 | Ball | 1 | 127 | 1 | 0.432 | 1.000 |
| B2 | Family | *Dennstaedtiaceae* | 1 | 223 | 0 | 1.0 | 0.241 | Ball | 1 | 127 | 0 | 1.000 | 0.568 |
| B2 | Family | *Cupressaceae* | 1 | 223 | 1 | 0.759 | 1.0 | Ball | 1 | 127 | 0 | 1.000 | 0.568 |
| B2 | Family | *Lamiaceae* | 2 | 223 | 1 | 0.942 | 0.425 | Ball | 2 | 127 | 1 | 0.678 | 0.814 |
| B2 | Family | *Dryopteridaceae* | 3 | 223 | 2 | 0.854 | 0.565 | Ball | 3 | 127 | 1 | 0.818 | 0.602 |
| B2 | Family | *Brassicaceae* | 13 | 223 | 11 | 0.354 | 0.865 | Ball | 13 | 127 | 5 | 0.735 | 0.479 |
| B2 | Family | *Pinaceae* | 1 | 223 | 1 | 0.759 | 1.0 | Ball | 1 | 127 | 0 | 1.000 | 0.568 |
| B2 | Family | *Geraniaceae* | 1 | 223 | 0 | 1.0 | 0.241 | Ball | 1 | 127 | 0 | 1.000 | 0.568 |
| B2 | Family | *Boraginaceae* | 4 | 223 | 4 | 0.329 | 1.0 | Ball | 4 | 127 | 0 | 1.000 | 0.102 |
| B2 | Family | *Plantaginaceae* | 2 | 223 | 1 | 0.942 | 0.425 | Ball | 2 | 127 | 0 | 1.000 | 0.322 |
| B2 | Family | *Cornaceae* | 1 | 223 | 1 | 0.759 | 1.0 | Ball | 1 | 127 | 1 | 0.432 | 1.000 |
| B2 | Family | *Crassulaceae* | 3 | 223 | 1 | 0.986 | 0.146 | Ball | 3 | 127 | 0 | 1.000 | 0.182 |
| B2 | Family | *Gentianaceae* | 4 | 223 | 3 | 0.753 | 0.671 | Ball | 4 | 127 | 2 | 0.580 | 0.784 |
| B2 | Family | *Caryophyllaceae* | 10 | 223 | 9 | 0.259 | 0.940 | Ball | 10 | 127 | 1 | 0.997 | 0.028 |
| B2 | Family | *Parnassiaceae* | 1 | 223 | 1 | 0.759 | 1.0 | Ball | 1 | 127 | 0 | 1.000 | 0.568 |
| B2 | Family | *Campanulaceae* | 1 | 223 | 1 | 0.759 | 1.0 | Ball | 1 | 127 | 1 | 0.432 | 1.000 |
| B2 | Family | *Apiaceae* | 5 | 223 | 2 | 0.987 | 0.093 | Ball | 5 | 127 | 2 | 0.718 | 0.627 |
| B2 | Family | *Aspleniaceae* | 2 | 223 | 1 | 0.942 | 0.425 | Ball | 2 | 127 | 0 | 1.000 | 0.322 |
| B2 | Family | *Ophioglossaceae* | 5 | 223 | 2 | 0.987 | 0.093 | Ball | 5 | 127 | 1 | 0.942 | 0.282 |
| B2 | Family | *Thelypteridaceae* | 2 | 223 | 0 | 1.0 | 0.058 | Ball | 2 | 127 | 2 | 0.186 | 1.000 |
| B2 | Family | *Selaginellaceae* | 2 | 223 | 2 | 0.575 | 1.0 | Ball | 2 | 127 | 2 | 0.186 | 1.000 |
| B2 | Family | *Urticaceae* | 1 | 223 | 0 | 1.0 | 0.241 | Ball | 1 | 127 | 0 | 1.000 | 0.568 |
| B2 | Family | *Orobanchaceae* | 1 | 223 | 1 | 0.759 | 1.0 | Ball | 1 | 127 | 0 | 1.000 | 0.568 |
| B2 | Family | *Saxifragaceae* | 4 | 223 | 3 | 0.753 | 0.671 | Ball | 4 | 127 | 4 | 0.034 | 1.000 |
| B2 | Family | *Balsaminaceae* | 1 | 223 | 1 | 0.759 | 1.0 | Ball | 1 | 127 | 0 | 1.000 | 0.568 |
| B2 | Family | *Poaceae* | 30 | 223 | 26 | 0.104 | 0.961 | Ball | 30 | 127 | 13 | 0.568 | 0.586 |
| B2 | Family | *Woodsiaceae* | 6 | 223 | 6 | 0.187 | 1.0 | Ball | 6 | 127 | 5 | 0.056 | 0.994 |
| B2 | Family | *Onagraceae* | 7 | 223 | 5 | 0.776 | 0.536 | Ball | 7 | 127 | 1 | 0.982 | 0.118 |
| B2 | Family | *Orchidaceae* | 6 | 223 | 5 | 0.553 | 0.813 | Ball | 6 | 127 | 1 | 0.968 | 0.184 |
| B2 | Family | *Juncaginaceae* | 1 | 223 | 1 | 0.759 | 1.0 | Ball | 1 | 127 | 0 | 1.000 | 0.568 |
| B2 | Family | *Rosaceae* | 18 | 223 | 11 | 0.958 | 0.113 | Ball | 18 | 127 | 9 | 0.358 | 0.802 |
| B2 | Family | *Primulaceae* | 4 | 223 | 3 | 0.753 | 0.671 | Ball | 4 | 127 | 2 | 0.580 | 0.784 |
| B2 | Family | *Ranunculaceae* | 11 | 223 | 8 | 0.739 | 0.520 | Ball | 11 | 127 | 3 | 0.922 | 0.221 |
| B2 | Family | *Fabaceae* | 5 | 223 | 5 | 0.248 | 1.0 | Ball | 5 | 127 | 2 | 0.718 | 0.627 |
| B2 | Family | *Rubiaceae* | 1 | 223 | 0 | 1.0 | 0.241 | Ball | 1 | 127 | 0 | 1.000 | 0.568 |
| B2 | Family | *Polygonaceae* | 8 | 223 | 6 | 0.701 | 0.614 | Ball | 8 | 127 | 3 | 0.751 | 0.519 |
| B2 | Family | *Equisetaceae* | 2 | 223 | 0 | 1.0 | 0.058 | Ball | 2 | 127 | 2 | 0.186 | 1.000 |
| B2 | Family | *Colchicaceae* | 2 | 223 | 1 | 0.942 | 0.425 | Ball | 2 | 127 | 2 | 0.186 | 1.000 |
| B2 | Family | *Onocleaceae* | 1 | 223 | 0 | 1.0 | 0.241 | Ball | 1 | 127 | 1 | 0.432 | 1.000 |
| B2 | Family | *Asteraceae* | 31 | 223 | 23 | 0.682 | 0.485 | Ball | 31 | 127 | 10 | 0.934 | 0.133 |
| B2 | Family | *Liliaceae* | 3 | 223 | 2 | 0.854 | 0.565 | Ball | 3 | 127 | 2 | 0.398 | 0.920 |
| B2 | Family | *Callitrichaceae* | 1 | 223 | 1 | 0.759 | 1.0 | Ball | 1 | 127 | 1 | 0.432 | 1.000 |
| B2 | Family | *Salicaceae* | 11 | 223 | 10 | 0.210 | 0.955 | Ball | 11 | 127 | 6 | 0.318 | 0.861 |
| B2 | Family | *Papaveraceae* | 1 | 223 | 1 | 0.759 | 1.0 | Ball | 1 | 127 | 1 | 0.432 | 1.000 |
| B2 | Family | *Diapensiaceae* | 1 | 223 | 1 | 0.759 | 1.0 | Ball | 1 | 127 | 1 | 0.432 | 1.000 |
| B2 | Family | *Potamogetonaceae* | 1 | 223 | 1 | 0.759 | 1.0 | Ball | 1 | 127 | 1 | 0.432 | 1.000 |
| B2 | Family | *Betulaceae* | 3 | 223 | 1 | 0.986 | 0.146 | Ball | 3 | 127 | 3 | 0.080 | 1.000 |
| B2 | Family | *Ericaceae* | 12 | 223 | 12 | 0.034 | 1.0 | Ball | 12 | 127 | 10 | 0.005 | 0.999 |
| B2 | Family | *Asparagaceae* | 2 | 223 | 0 | 1.0 | 0.058 | Ball | 2 | 127 | 1 | 0.678 | 0.814 |
| B2 | Family | *Lycopodiaceae* | 4 | 223 | 3 | 0.753 | 0.671 | Ball | 4 | 127 | 3 | 0.216 | 0.966 |
| B2 | Family | *Typhaceae* | 1 | 223 | 1 | 0.759 | 1.0 | Ball | 1 | 127 | 0 | 1.000 | 0.568 |
| B2 | Family | *Cyperaceae* | 18 | 223 | 15 | 0.328 | 0.855 | Ball | 18 | 127 | 8 | 0.550 | 0.642 |
| B2 | Family | *Scrophulariaceae* | 11 | 223 | 10 | 0.210 | 0.955 | Ball | 11 | 127 | 7 | 0.139 | 0.956 |
| B2 | Family | *Empetraceae* | 1 | 223 | 1 | 0.759 | 1.0 | Ball | 1 | 127 | 1 | 0.432 | 1.000 |
| B2 | Family | *Alliaceae* | 2 | 223 | 0 | 1.0 | 0.058 | Ball | 2 | 127 | 0 | 1.000 | 0.322 |
| B2 | Family | *Adiantaceae* | 1 | 223 | 1 | 0.759 | 1.0 | Ball | 1 | 127 | 1 | 0.432 | 1.000 |
| B2 | Family | *Violaceae* | 5 | 223 | 3 | 0.907 | 0.349 | Ball | 5 | 127 | 2 | 0.718 | 0.627 |
| B2 | Family | *Melanthiaceae* | 1 | 223 | 1 | 0.759 | 1.0 | Ball | 1 | 127 | 0 | 1.000 | 0.568 |
| B2 | Family | *Iridaceae* | 1 | 223 | 0 | 1.0 | 0.241 | Ball | 1 | 127 | 0 | 1.000 | 0.568 |
| To | Family | *Juncaceae* | 9 | 16 | 0 | 1.0 | 0.600 | To | 9 | 25 | 2 | 0.173 | 0.967 |
| To | Family | *Valerianaceae* | 1 | 16 | 0 | 1.0 | 0.946 | To | 1 | 25 | 0 | 1.000 | 0.915 |
| To | Family | *Dennstaedtiaceae* | 1 | 16 | 0 | 1.0 | 0.946 | To | 1 | 25 | 0 | 1.000 | 0.915 |
| To | Family | *Cupressaceae* | 1 | 16 | 0 | 1.0 | 0.946 | To | 1 | 25 | 0 | 1.000 | 0.915 |
| To | Family | *Lamiaceae* | 2 | 16 | 0 | 1.0 | 0.894 | To | 2 | 25 | 0 | 1.000 | 0.837 |
| To | Family | *Dryopteridaceae* | 3 | 16 | 0 | 1.0 | 0.845 | To | 3 | 25 | 0 | 1.000 | 0.765 |
| To | Family | *Brassicaceae* | 13 | 16 | 0 | 1.0 | 0.476 | To | 13 | 25 | 0 | 1.000 | 0.307 |
| To | Family | *Pinaceae* | 1 | 16 | 0 | 1.0 | 0.946 | To | 1 | 25 | 0 | 1.000 | 0.915 |
| To | Family | *Geraniaceae* | 1 | 16 | 0 | 1.0 | 0.946 | To | 1 | 25 | 0 | 1.000 | 0.915 |
| To | Family | *Boraginaceae* | 4 | 16 | 0 | 1.0 | 0.799 | To | 4 | 25 | 1 | 0.300 | 0.962 |
| To | Family | *Plantaginaceae* | 2 | 16 | 1 | 0.106 | 0.997 | To | 2 | 25 | 1 | 0.163 | 0.993 |
| To | Family | *Cornaceae* | 1 | 16 | 0 | 1.0 | 0.946 | To | 1 | 25 | 0 | 1.000 | 0.915 |
| To | Family | *Crassulaceae* | 3 | 16 | 1 | 0.155 | 0.992 | To | 3 | 25 | 1 | 0.235 | 0.980 |
| To | Family | *Gentianaceae* | 4 | 16 | 0 | 1.0 | 0.799 | To | 4 | 25 | 0 | 1.000 | 0.700 |
| To | Family | *Caryophyllaceae* | 10 | 16 | 1 | 0.434 | 0.903 | To | 10 | 25 | 1 | 0.595 | 0.795 |
| To | Family | *Parnassiaceae* | 1 | 16 | 0 | 1.0 | 0.946 | To | 1 | 25 | 0 | 1.000 | 0.915 |
| To | Family | *Campanulaceae* | 1 | 16 | 0 | 1.0 | 0.946 | To | 1 | 25 | 0 | 1.000 | 0.915 |
| To | Family | *Apiaceae* | 5 | 16 | 1 | 0.246 | 0.975 | To | 5 | 25 | 1 | 0.361 | 0.941 |
| To | Family | *Aspleniaceae* | 2 | 16 | 0 | 1.0 | 0.894 | To | 2 | 25 | 0 | 1.000 | 0.837 |
| To | Family | *Ophioglossaceae* | 5 | 16 | 1 | 0.246 | 0.975 | To | 5 | 25 | 1 | 0.361 | 0.941 |
| To | Family | *Thelypteridaceae* | 2 | 16 | 0 | 1.0 | 0.894 | To | 2 | 25 | 0 | 1.000 | 0.837 |
| To | Family | *Selaginellaceae* | 2 | 16 | 0 | 1.0 | 0.894 | To | 2 | 25 | 0 | 1.000 | 0.837 |
| To | Family | *Urticaceae* | 1 | 16 | 0 | 1.0 | 0.946 | To | 1 | 25 | 0 | 1.000 | 0.915 |
| To | Family | *Orobanchaceae* | 1 | 16 | 0 | 1.0 | 0.946 | To | 1 | 25 | 0 | 1.000 | 0.915 |
| To | Family | *Saxifragaceae* | 4 | 16 | 0 | 1.0 | 0.799 | To | 4 | 25 | 0 | 1.000 | 0.700 |
| To | Family | *Balsaminaceae* | 1 | 16 | 0 | 1.0 | 0.946 | To | 1 | 25 | 0 | 1.000 | 0.915 |
| To | Family | *Poaceae* | 30 | 16 | 3 | 0.216 | 0.933 | To | 30 | 25 | 4 | 0.242 | 0.905 |
| To | Family | *Woodsiaceae* | 6 | 16 | 0 | 1.0 | 0.713 | To | 6 | 25 | 0 | 1.000 | 0.584 |
| To | Family | *Onagraceae* | 7 | 16 | 0 | 1.0 | 0.673 | To | 7 | 25 | 0 | 1.000 | 0.533 |
| To | Family | *Orchidaceae* | 6 | 16 | 0 | 1.0 | 0.713 | To | 6 | 25 | 1 | 0.416 | 0.916 |
| To | Family | *Juncaginaceae* | 1 | 16 | 0 | 1.0 | 0.946 | To | 1 | 25 | 0 | 1.000 | 0.915 |
| To | Family | *Rosaceae* | 18 | 16 | 4 | 0.011 | 0.999 | To | 18 | 25 | 4 | 0.055 | 0.988 |
| To | Family | *Primulaceae* | 4 | 16 | 0 | 1.0 | 0.799 | To | 4 | 25 | 0 | 1.000 | 0.700 |
| To | Family | *Ranunculaceae* | 11 | 16 | 0 | 1.0 | 0.534 | To | 11 | 25 | 0 | 1.000 | 0.370 |
| To | Family | *Fabaceae* | 5 | 16 | 0 | 1.0 | 0.754 | To | 5 | 25 | 0 | 1.000 | 0.639 |
| To | Family | *Rubiaceae* | 1 | 16 | 0 | 1.0 | 0.946 | To | 1 | 25 | 0 | 1.000 | 0.915 |
| To | Family | *Polygonaceae* | 8 | 16 | 0 | 1.0 | 0.636 | To | 8 | 25 | 0 | 1.000 | 0.487 |
| To | Family | *Equisetaceae* | 2 | 16 | 0 | 1.0 | 0.894 | To | 2 | 25 | 0 | 1.000 | 0.837 |
| To | Family | *Colchicaceae* | 2 | 16 | 0 | 1.0 | 0.894 | To | 2 | 25 | 0 | 1.000 | 0.837 |
| To | Family | *Onocleaceae* | 1 | 16 | 0 | 1.0 | 0.946 | To | 1 | 25 | 0 | 1.000 | 0.915 |
| To | Family | *Asteraceae* | 31 | 16 | 0 | 1.0 | 0.160 | To | 31 | 25 | 1 | 0.946 | 0.230 |
| To | Family | *Liliaceae* | 3 | 16 | 0 | 1.0 | 0.845 | To | 3 | 25 | 0 | 1.000 | 0.765 |
| To | Family | *Callitrichaceae* | 1 | 16 | 0 | 1.0 | 0.946 | To | 1 | 25 | 0 | 1.000 | 0.915 |
| To | Family | *Salicaceae* | 11 | 16 | 0 | 1.0 | 0.534 | To | 11 | 25 | 0 | 1.000 | 0.370 |
| To | Family | *Papaveraceae* | 1 | 16 | 0 | 1.0 | 0.946 | To | 1 | 25 | 0 | 1.000 | 0.915 |
| To | Family | *Diapensiaceae* | 1 | 16 | 0 | 1.0 | 0.946 | To | 1 | 25 | 0 | 1.000 | 0.915 |
| To | Family | *Potamogetonaceae* | 1 | 16 | 0 | 1.0 | 0.946 | To | 1 | 25 | 0 | 1.000 | 0.915 |
| To | Family | *Betulaceae* | 3 | 16 | 0 | 1.0 | 0.845 | To | 3 | 25 | 0 | 1.000 | 0.765 |
| To | Family | *Ericaceae* | 12 | 16 | 0 | 1.0 | 0.504 | To | 12 | 25 | 0 | 1.000 | 0.337 |
| To | Family | *Asparagaceae* | 2 | 16 | 0 | 1.0 | 0.894 | To | 2 | 25 | 0 | 1.000 | 0.837 |
| To | Family | *Lycopodiaceae* | 4 | 16 | 0 | 1.0 | 0.799 | To | 4 | 25 | 0 | 1.000 | 0.700 |
| To | Family | *Typhaceae* | 1 | 16 | 0 | 1.0 | 0.946 | To | 1 | 25 | 0 | 1.000 | 0.915 |
| To | Family | *Cyperaceae* | 18 | 16 | 3 | 0.065 | 0.989 | To | 18 | 25 | 6 | 0.002 | 1.000 |
| To | Family | *Scrophulariaceae* | 11 | 16 | 0 | 1.0 | 0.534 | To | 11 | 25 | 0 | 1.000 | 0.370 |
| To | Family | *Empetraceae* | 1 | 16 | 0 | 1.0 | 0.946 | To | 1 | 25 | 0 | 1.000 | 0.915 |
| To | Family | *Alliaceae* | 2 | 16 | 1 | 0.106 | 0.997 | To | 2 | 25 | 1 | 0.163 | 0.993 |
| To | Family | *Adiantaceae* | 1 | 16 | 0 | 1.0 | 0.946 | To | 1 | 25 | 0 | 1.000 | 0.915 |
| To | Family | *Violaceae* | 5 | 16 | 0 | 1.0 | 0.754 | To | 5 | 25 | 0 | 1.000 | 0.639 |
| To | Family | *Melanthiaceae* | 1 | 16 | 0 | 1.0 | 0.946 | To | 1 | 25 | 0 | 1.000 | 0.915 |
| To | Family | *Iridaceae* | 1 | 16 | 0 | 1.0 | 0.946 | To | 1 | 25 | 0 | 1.000 | 0.915 |
| B1 | Order | *Lycopodiales* | 4 | 22 | 1 | 0.269 | 0.971 | B1 | NA | NA | NA | NA | NA |
| B1 | Order | *Asparagales* | 11 | 22 | 1 | 0.581 | 0.805 | B1 | NA | NA | NA | NA | NA |
| B1 | Order | *Liliales* | 5 | 22 | 2 | 0.047 | 0.997 | B1 | NA | NA | NA | NA | NA |
| B1 | Order | *Brassicales* | 13 | 22 | 1 | 0.644 | 0.747 | B1 | NA | NA | NA | NA | NA |
| B1 | Order | *Ranunculales* | 12 | 22 | 0 | 1.0 | 0.386 | B1 | NA | NA | NA | NA | NA |
| B1 | Order | *Polypodiales* | 15 | 22 | 4 | 0.019 | 0.997 | B1 | NA | NA | NA | NA | NA |
| B1 | Order | *Myrtales* | 7 | 22 | 0 | 1.0 | 0.577 | B1 | NA | NA | NA | NA | NA |
| B1 | Order | *Ericales* | 19 | 22 | 0 | 1.0 | 0.217 | B1 | NA | NA | NA | NA | NA |
| B1 | Order | *Cornales* | 1 | 22 | 0 | 1.0 | 0.925 | B1 | NA | NA | NA | NA | NA |
| B1 | Order | *Pinales* | 2 | 22 | 0 | 1.0 | 0.856 | B1 | NA | NA | NA | NA | NA |
| B1 | Order | *Gentianales* | 5 | 22 | 0 | 1.0 | 0.676 | B1 | NA | NA | NA | NA | NA |
| B1 | Order | *Poales* | 58 | 22 | 0 | 1.0 | 0.006 | B1 | NA | NA | NA | NA | NA |
| B1 | Order | *Lamiales* | 17 | 22 | 0 | 1.0 | 0.256 | B1 | NA | NA | NA | NA | NA |
| B1 | Order | *Dipsacales* | 1 | 22 | 0 | 1.0 | 0.925 | B1 | NA | NA | NA | NA | NA |
| B1 | Order | *Fagales* | 3 | 22 | 2 | 0.015 | 1.0 | B1 | NA | NA | NA | NA | NA |
| B1 | Order | *Alismatales* | 2 | 22 | 0 | 1.0 | 0.856 | B1 | NA | NA | NA | NA | NA |
| B1 | Order | *Pteridales* | 1 | 22 | 0 | 1.0 | 0.925 | B1 | NA | NA | NA | NA | NA |
| B1 | Order | *Synandrae* | 32 | 22 | 2 | 0.719 | 0.562 | B1 | NA | NA | NA | NA | NA |
| B1 | Order | *Rosales* | 19 | 22 | 1 | 0.783 | 0.575 | B1 | NA | NA | NA | NA | NA |
| B1 | Order | *Fabales* | 5 | 22 | 0 | 1.0 | 0.676 | B1 | NA | NA | NA | NA | NA |
| B1 | Order | *Selaginellales* | 2 | 22 | 0 | 1.0 | 0.856 | B1 | NA | NA | NA | NA | NA |
| B1 | Order | *Boraginales* | 4 | 22 | 0 | 1.0 | 0.731 | B1 | NA | NA | NA | NA | NA |
| B1 | Order | *Equisetales* | 2 | 22 | 2 | 0.005 | 1.0 | B1 | NA | NA | NA | NA | NA |
| B1 | Order | *Apiales* | 5 | 22 | 1 | 0.324 | 0.953 | B1 | NA | NA | NA | NA | NA |
| B1 | Order | *Melanthiales* | 1 | 22 | 0 | 1.0 | 0.925 | B1 | NA | NA | NA | NA | NA |
| B1 | Order | *Malpighiales* | 16 | 22 | 2 | 0.340 | 0.892 | B1 | NA | NA | NA | NA | NA |
| B1 | Order | *Geraniales* | 1 | 22 | 0 | 1.0 | 0.925 | B1 | NA | NA | NA | NA | NA |
| B1 | Order | *Caryophyllales* | 18 | 22 | 1 | 0.764 | 0.603 | B1 | NA | NA | NA | NA | NA |
| B1 | Order | *Ophioglossales* | 5 | 22 | 1 | 0.324 | 0.953 | B1 | NA | NA | NA | NA | NA |
| B1 | Order | *Celastrales* | 1 | 22 | 0 | 1.0 | 0.925 | B1 | NA | NA | NA | NA | NA |
| B1 | Order | *Saxifragales* | 7 | 22 | 1 | 0.423 | 0.911 | B1 | NA | NA | NA | NA | NA |
| Tall | Order | *Lycopodiales* | 4 | 49 | 0 | 1.0 | 0.480 | Tall | 4 | 167 | 1 | 0.966 | 0.216 |
| Tall | Order | *Asparagales* | 11 | 49 | 5 | 0.022 | 0.996 | Tall | 11 | 167 | 9 | 0.078 | 0.983 |
| Tall | Order | *Liliales* | 5 | 49 | 0 | 1.0 | 0.399 | Tall | 5 | 167 | 1 | 0.986 | 0.112 |
| Tall | Order | *Brassicales* | 13 | 49 | 1 | 0.912 | 0.330 | Tall | 13 | 167 | 8 | 0.479 | 0.735 |
| Tall | Order | *Ranunculales* | 12 | 49 | 3 | 0.322 | 0.879 | Tall | 12 | 167 | 8 | 0.347 | 0.842 |
| Tall | Order | *Polypodiales* | 15 | 49 | 2 | 0.748 | 0.530 | Tall | 15 | 167 | 6 | 0.946 | 0.140 |
| Tall | Order | *Myrtales* | 7 | 49 | 2 | 0.330 | 0.907 | Tall | 7 | 167 | 6 | 0.118 | 0.982 |
| Tall | Order | *Ericales* | 19 | 49 | 1 | 0.972 | 0.141 | Tall | 19 | 167 | 5 | 0.999 | 0.006 |
| Tall | Order | *Cornales* | 1 | 49 | 0 | 1.0 | 0.833 | Tall | 1 | 167 | 0 | 1.000 | 0.432 |
| Tall | Order | *Pinales* | 2 | 49 | 0 | 1.0 | 0.694 | Tall | 2 | 167 | 2 | 0.322 | 1.000 |
| Tall | Order | *Gentianales* | 5 | 49 | 2 | 0.195 | 0.966 | Tall | 5 | 167 | 3 | 0.627 | 0.718 |
| Tall | Order | *Poales* | 58 | 49 | 7 | 0.897 | 0.199 | Tall | 58 | 167 | 36 | 0.226 | 0.854 |
| Tall | Order | *Lamiales* | 17 | 49 | 3 | 0.562 | 0.691 | Tall | 17 | 167 | 8 | 0.861 | 0.278 |
| Tall | Order | *Dipsacales* | 1 | 49 | 0 | 1.0 | 0.833 | Tall | 1 | 167 | 0 | 1.000 | 0.432 |
| Tall | Order | *Fagales* | 3 | 49 | 0 | 1.0 | 0.578 | Tall | 3 | 167 | 0 | 1.000 | 0.080 |
| Tall | Order | *Alismatales* | 2 | 49 | 0 | 1.0 | 0.694 | Tall | 2 | 167 | 1 | 0.814 | 0.678 |
| Tall | Order | *Pteridales* | 1 | 49 | 0 | 1.0 | 0.833 | Tall | 1 | 167 | 0 | 1.000 | 0.432 |
| Tall | Order | *Synandrae* | 32 | 49 | 6 | 0.449 | 0.731 | Tall | 32 | 167 | 21 | 0.190 | 0.896 |
| Tall | Order | *Rosales* | 19 | 49 | 7 | 0.024 | 0.994 | Tall | 19 | 167 | 10 | 0.733 | 0.441 |
| Tall | Order | *Fabales* | 5 | 49 | 0 | 1.0 | 0.399 | Tall | 5 | 167 | 3 | 0.627 | 0.718 |
| Tall | Order | *Selaginellales* | 2 | 49 | 0 | 1.0 | 0.694 | Tall | 2 | 167 | 0 | 1.000 | 0.186 |
| Tall | Order | *Boraginales* | 4 | 49 | 0 | 1.0 | 0.480 | Tall | 4 | 167 | 4 | 0.102 | 1.000 |
| Tall | Order | *Equisetales* | 2 | 49 | 0 | 1.0 | 0.694 | Tall | 2 | 167 | 0 | 1.000 | 0.186 |
| Tall | Order | *Apiales* | 5 | 49 | 2 | 0.195 | 0.966 | Tall | 5 | 167 | 3 | 0.627 | 0.718 |
| Tall | Order | *Melanthiales* | 1 | 49 | 0 | 1.0 | 0.833 | Tall | 1 | 167 | 1 | 0.568 | 1.000 |
| Tall | Order | *Malpighiales* | 16 | 49 | 1 | 0.950 | 0.219 | Tall | 16 | 167 | 8 | 0.796 | 0.377 |
| Tall | Order | *Geraniales* | 1 | 49 | 1 | 0.167 | 1.0 | Tall | 1 | 167 | 1 | 0.568 | 1.000 |
| Tall | Order | *Caryophyllales* | 18 | 49 | 2 | 0.836 | 0.396 | Tall | 18 | 167 | 14 | 0.051 | 0.985 |
| Tall | Order | *Ophioglossales* | 5 | 49 | 2 | 0.195 | 0.966 | Tall | 5 | 167 | 4 | 0.282 | 0.942 |
| Tall | Order | *Celastrales* | 1 | 49 | 0 | 1.0 | 0.833 | Tall | 1 | 167 | 1 | 0.568 | 1.000 |
| Tall | Order | *Saxifragales* | 7 | 49 | 2 | 0.330 | 0.907 | Tall | 7 | 167 | 3 | 0.872 | 0.352 |
| Tf | Order | *Lycopodiales* | 4 | 33 | 0 | 1.0 | 0.620 | Tf | 4 | 142 | 1 | 0.930 | 0.337 |
| Tf | Order | *Asparagales* | 11 | 33 | 4 | 0.025 | 0.996 | Tf | 11 | 142 | 7 | 0.233 | 0.911 |
| Tf | Order | *Liliales* | 5 | 33 | 0 | 1.0 | 0.549 | Tf | 5 | 142 | 1 | 0.964 | 0.207 |
| Tf | Order | *Brassicales* | 13 | 33 | 1 | 0.795 | 0.560 | Tf | 13 | 142 | 8 | 0.244 | 0.897 |
| Tf | Order | *Ranunculales* | 12 | 33 | 3 | 0.140 | 0.966 | Tf | 12 | 142 | 8 | 0.158 | 0.946 |
| Tf | Order | *Polypodiales* | 15 | 33 | 2 | 0.519 | 0.770 | Tf | 15 | 142 | 6 | 0.822 | 0.348 |
| Tf | Order | *Myrtales* | 7 | 33 | 2 | 0.179 | 0.967 | Tf | 7 | 142 | 6 | 0.050 | 0.994 |
| Tf | Order | *Ericales* | 19 | 33 | 1 | 0.904 | 0.345 | Tf | 19 | 142 | 5 | 0.988 | 0.039 |
| Tf | Order | *Cornales* | 1 | 33 | 0 | 1.0 | 0.888 | Tf | 1 | 142 | 0 | 1.000 | 0.517 |
| Tf | Order | *Pinales* | 2 | 33 | 0 | 1.0 | 0.788 | Tf | 2 | 142 | 2 | 0.232 | 1.000 |
| Tf | Order | *Gentianales* | 5 | 33 | 2 | 0.099 | 0.989 | Tf | 5 | 142 | 3 | 0.468 | 0.835 |
| Tf | Order | *Poales* | 58 | 33 | 1 | 1.0 | 0.005 | Tf | 58 | 142 | 24 | 0.907 | 0.151 |
| Tf | Order | *Lamiales* | 17 | 33 | 2 | 0.591 | 0.705 | Tf | 17 | 142 | 7 | 0.803 | 0.363 |
| Tf | Order | *Dipsacales* | 1 | 33 | 0 | 1.0 | 0.888 | Tf | 1 | 142 | 0 | 1.000 | 0.517 |
| Tf | Order | *Fagales* | 3 | 33 | 0 | 1.0 | 0.699 | Tf | 3 | 142 | 0 | 1.000 | 0.137 |
| Tf | Order | *Alismatales* | 2 | 33 | 0 | 1.0 | 0.788 | Tf | 2 | 142 | 1 | 0.734 | 0.768 |
| Tf | Order | *Pteridales* | 1 | 33 | 0 | 1.0 | 0.888 | Tf | 1 | 142 | 0 | 1.000 | 0.517 |
| Tf | Order | *Synandrae* | 32 | 33 | 6 | 0.131 | 0.950 | Tf | 32 | 142 | 20 | 0.065 | 0.971 |
| Tf | Order | *Rosales* | 19 | 33 | 3 | 0.361 | 0.849 | Tf | 19 | 142 | 6 | 0.961 | 0.101 |
| Tf | Order | *Fabales* | 5 | 33 | 0 | 1.0 | 0.549 | Tf | 5 | 142 | 3 | 0.468 | 0.835 |
| Tf | Order | *Selaginellales* | 2 | 33 | 0 | 1.0 | 0.788 | Tf | 2 | 142 | 0 | 1.000 | 0.266 |
| Tf | Order | *Boraginales* | 4 | 33 | 0 | 1.0 | 0.620 | Tf | 4 | 142 | 3 | 0.286 | 0.947 |
| Tf | Order | *Equisetales* | 2 | 33 | 0 | 1.0 | 0.788 | Tf | 2 | 142 | 0 | 1.000 | 0.266 |
| Tf | Order | *Apiales* | 5 | 33 | 1 | 0.451 | 0.901 | Tf | 5 | 142 | 2 | 0.793 | 0.532 |
| Tf | Order | *Melanthiales* | 1 | 33 | 0 | 1.0 | 0.888 | Tf | 1 | 142 | 1 | 0.483 | 1.000 |
| Tf | Order | *Malpighiales* | 16 | 33 | 1 | 0.859 | 0.444 | Tf | 16 | 142 | 8 | 0.545 | 0.655 |
| Tf | Order | *Geraniales* | 1 | 33 | 1 | 0.112 | 1.0 | Tf | 1 | 142 | 1 | 0.483 | 1.000 |
| Tf | Order | *Caryophyllales* | 18 | 33 | 1 | 0.890 | 0.376 | Tf | 18 | 142 | 13 | 0.031 | 0.991 |
| Tf | Order | *Ophioglossales* | 5 | 33 | 1 | 0.451 | 0.901 | Tf | 5 | 142 | 3 | 0.468 | 0.835 |
| Tf | Order | *Celastrales* | 1 | 33 | 0 | 1.0 | 0.888 | Tf | 1 | 142 | 1 | 0.483 | 1.000 |
| Tf | Order | *Saxifragales* | 7 | 33 | 1 | 0.569 | 0.821 | Tf | 7 | 142 | 2 | 0.928 | 0.253 |
| B2 | Order | *Lycopodiales* | 4 | 223 | 3 | 0.753 | 0.671 | Ball | 4 | 127 | 3 | 0.216 | 0.966 |
| B2 | Order | *Asparagales* | 11 | 223 | 5 | 0.995 | 0.027 | Ball | 11 | 127 | 2 | 0.983 | 0.078 |
| B2 | Order | *Liliales* | 5 | 223 | 3 | 0.907 | 0.349 | Ball | 5 | 127 | 4 | 0.112 | 0.986 |
| B2 | Order | *Brassicales* | 13 | 223 | 11 | 0.354 | 0.865 | Ball | 13 | 127 | 5 | 0.735 | 0.479 |
| B2 | Order | *Ranunculales* | 12 | 223 | 9 | 0.677 | 0.586 | Ball | 12 | 127 | 4 | 0.842 | 0.347 |
| B2 | Order | *Polypodiales* | 15 | 223 | 9 | 0.957 | 0.124 | Ball | 15 | 127 | 9 | 0.140 | 0.946 |
| B2 | Order | *Myrtales* | 7 | 223 | 5 | 0.776 | 0.536 | Ball | 7 | 127 | 1 | 0.982 | 0.118 |
| B2 | Order | *Ericales* | 19 | 223 | 18 | 0.033 | 0.996 | Ball | 19 | 127 | 14 | 0.006 | 0.999 |
| B2 | Order | *Cornales* | 1 | 223 | 1 | 0.759 | 1.0 | Ball | 1 | 127 | 1 | 0.432 | 1.000 |
| B2 | Order | *Pinales* | 2 | 223 | 2 | 0.575 | 1.0 | Ball | 2 | 127 | 0 | 1.000 | 0.322 |
| B2 | Order | *Gentianales* | 5 | 223 | 3 | 0.907 | 0.349 | Ball | 5 | 127 | 2 | 0.718 | 0.627 |
| B2 | Order | *Poales* | 58 | 223 | 51 | 0.010 | 0.997 | Ball | 58 | 127 | 22 | 0.854 | 0.226 |
| B2 | Order | *Lamiales* | 17 | 223 | 14 | 0.378 | 0.824 | Ball | 17 | 127 | 9 | 0.278 | 0.861 |
| B2 | Order | *Dipsacales* | 1 | 223 | 1 | 0.759 | 1.0 | Ball | 1 | 127 | 1 | 0.432 | 1.000 |
| B2 | Order | *Fagales* | 3 | 223 | 1 | 0.986 | 0.146 | Ball | 3 | 127 | 3 | 0.080 | 1.000 |
| B2 | Order | *Alismatales* | 2 | 223 | 2 | 0.575 | 1.0 | Ball | 2 | 127 | 1 | 0.678 | 0.814 |
| B2 | Order | *Pteridales* | 1 | 223 | 1 | 0.759 | 1.0 | Ball | 1 | 127 | 1 | 0.432 | 1.000 |
| B2 | Order | *Synandrae* | 32 | 223 | 24 | 0.642 | 0.528 | Ball | 32 | 127 | 11 | 0.896 | 0.190 |
| B2 | Order | *Rosales* | 19 | 223 | 11 | 0.981 | 0.059 | Ball | 19 | 127 | 9 | 0.441 | 0.733 |
| B2 | Order | *Fabales* | 5 | 223 | 5 | 0.248 | 1.0 | Ball | 5 | 127 | 2 | 0.718 | 0.627 |
| B2 | Order | *Selaginellales* | 2 | 223 | 2 | 0.575 | 1.0 | Ball | 2 | 127 | 2 | 0.186 | 1.000 |
| B2 | Order | *Boraginales* | 4 | 223 | 4 | 0.329 | 1.0 | Ball | 4 | 127 | 0 | 1.000 | 0.102 |
| B2 | Order | *Equisetales* | 2 | 223 | 0 | 1.0 | 0.058 | Ball | 2 | 127 | 2 | 0.186 | 1.000 |
| B2 | Order | *Apiales* | 5 | 223 | 2 | 0.987 | 0.093 | Ball | 5 | 127 | 2 | 0.718 | 0.627 |
| B2 | Order | *Melanthiales* | 1 | 223 | 1 | 0.759 | 1.0 | Ball | 1 | 127 | 0 | 1.000 | 0.568 |
| B2 | Order | *Malpighiales* | 16 | 223 | 13 | 0.432 | 0.788 | Ball | 16 | 127 | 8 | 0.377 | 0.796 |
| B2 | Order | *Geraniales* | 1 | 223 | 0 | 1.0 | 0.241 | Ball | 1 | 127 | 0 | 1.000 | 0.568 |
| B2 | Order | *Caryophyllales* | 18 | 223 | 15 | 0.328 | 0.855 | Ball | 18 | 127 | 4 | 0.985 | 0.051 |
| B2 | Order | *Ophioglossales* | 5 | 223 | 2 | 0.987 | 0.093 | Ball | 5 | 127 | 1 | 0.942 | 0.282 |
| B2 | Order | *Celastrales* | 1 | 223 | 1 | 0.759 | 1.0 | Ball | 1 | 127 | 0 | 1.000 | 0.568 |
| B2 | Order | *Saxifragales* | 7 | 223 | 4 | 0.939 | 0.224 | Ball | 7 | 127 | 4 | 0.352 | 0.872 |
| To | Order | *Lycopodiales* | 4 | 16 | 0 | 1.0 | 0.799 | To | 4 | 25 | 0 | 1.000 | 0.700 |
| To | Order | *Asparagales* | 11 | 16 | 1 | 0.466 | 0.885 | To | 11 | 25 | 2 | 0.238 | 0.943 |
| To | Order | *Liliales* | 5 | 16 | 0 | 1.0 | 0.754 | To | 5 | 25 | 0 | 1.000 | 0.639 |
| To | Order | *Brassicales* | 13 | 16 | 0 | 1.0 | 0.476 | To | 13 | 25 | 0 | 1.000 | 0.307 |
| To | Order | *Ranunculales* | 12 | 16 | 0 | 1.0 | 0.504 | To | 12 | 25 | 0 | 1.000 | 0.337 |
| To | Order | *Polypodiales* | 15 | 16 | 0 | 1.0 | 0.423 | To | 15 | 25 | 0 | 1.000 | 0.255 |
| To | Order | *Myrtales* | 7 | 16 | 0 | 1.0 | 0.673 | To | 7 | 25 | 0 | 1.000 | 0.533 |
| To | Order | *Ericales* | 19 | 16 | 0 | 1.0 | 0.333 | To | 19 | 25 | 0 | 1.000 | 0.175 |
| To | Order | *Cornales* | 1 | 16 | 0 | 1.0 | 0.946 | To | 1 | 25 | 0 | 1.000 | 0.915 |
| To | Order | *Pinales* | 2 | 16 | 0 | 1.0 | 0.894 | To | 2 | 25 | 0 | 1.000 | 0.837 |
| To | Order | *Gentianales* | 5 | 16 | 0 | 1.0 | 0.754 | To | 5 | 25 | 0 | 1.000 | 0.639 |
| To | Order | *Poales* | 58 | 16 | 6 | 0.072 | 0.979 | To | 58 | 25 | 12 | 0.001 | 1.000 |
| To | Order | *Lamiales* | 17 | 16 | 1 | 0.624 | 0.766 | To | 17 | 25 | 1 | 0.789 | 0.566 |
| To | Order | *Dipsacales* | 1 | 16 | 0 | 1.0 | 0.946 | To | 1 | 25 | 0 | 1.000 | 0.915 |
| To | Order | *Fagales* | 3 | 16 | 0 | 1.0 | 0.845 | To | 3 | 25 | 0 | 1.000 | 0.765 |
| To | Order | *Alismatales* | 2 | 16 | 0 | 1.0 | 0.894 | To | 2 | 25 | 0 | 1.000 | 0.837 |
| To | Order | *Pteridales* | 1 | 16 | 0 | 1.0 | 0.946 | To | 1 | 25 | 0 | 1.000 | 0.915 |
| To | Order | *Synandrae* | 32 | 16 | 0 | 1.0 | 0.150 | To | 32 | 25 | 1 | 0.951 | 0.214 |
| To | Order | *Rosales* | 19 | 16 | 4 | 0.014 | 0.998 | To | 19 | 25 | 4 | 0.066 | 0.985 |
| To | Order | *Fabales* | 5 | 16 | 0 | 1.0 | 0.754 | To | 5 | 25 | 0 | 1.000 | 0.639 |
| To | Order | *Selaginellales* | 2 | 16 | 0 | 1.0 | 0.894 | To | 2 | 25 | 0 | 1.000 | 0.837 |
| To | Order | *Boraginales* | 4 | 16 | 0 | 1.0 | 0.799 | To | 4 | 25 | 1 | 0.300 | 0.962 |
| To | Order | *Equisetales* | 2 | 16 | 0 | 1.0 | 0.894 | To | 2 | 25 | 0 | 1.000 | 0.837 |
| To | Order | *Apiales* | 5 | 16 | 1 | 0.246 | 0.975 | To | 5 | 25 | 1 | 0.361 | 0.941 |
| To | Order | *Melanthiales* | 1 | 16 | 0 | 1.0 | 0.946 | To | 1 | 25 | 0 | 1.000 | 0.915 |
| To | Order | *Malpighiales* | 16 | 16 | 0 | 1.0 | 0.399 | To | 16 | 25 | 0 | 1.000 | 0.232 |
| To | Order | *Geraniales* | 1 | 16 | 0 | 1.0 | 0.946 | To | 1 | 25 | 0 | 1.000 | 0.915 |
| To | Order | *Caryophyllales* | 18 | 16 | 1 | 0.646 | 0.745 | To | 18 | 25 | 1 | 0.808 | 0.535 |
| To | Order | *Ophioglossales* | 5 | 16 | 1 | 0.246 | 0.975 | To | 5 | 25 | 1 | 0.361 | 0.941 |
| To | Order | *Celastrales* | 1 | 16 | 0 | 1.0 | 0.946 | To | 1 | 25 | 0 | 1.000 | 0.915 |
| To | Order | *Saxifragales* | 7 | 16 | 1 | 0.327 | 0.950 | To | 7 | 25 | 1 | 0.467 | 0.888 |
| B1 | Class | Pinopsida | 2 | 22 | 0 | 1.0 | 0.856 | B1 | NA | NA | NA | NA | NA |
| B1 | Class | Polypodiopsida | 16 | 22 | 4 | 0.024 | 0.996 | B1 | NA | NA | NA | NA | NA |
| B1 | Class | Isoetopsida | 2 | 22 | 0 | 1.0 | 0.856 | B1 | NA | NA | NA | NA | NA |
| B1 | Class | Psilotopsida | 5 | 22 | 1 | 0.324 | 0.953 | B1 | NA | NA | NA | NA | NA |
| B1 | Class | Equisetopsida | 2 | 22 | 2 | 0.005 | 1.0 | B1 | NA | NA | NA | NA | NA |
| B1 | Class | Lycopodiopsida | 4 | 22 | 1 | 0.269 | 0.971 | B1 | NA | NA | NA | NA | NA |
| B1 | Class | Magnoliopsida | 186 | 22 | 11 | 0.940 | 0.134 | B1 | NA | NA | NA | NA | NA |
| B1 | Class | Liliopsida | 77 | 22 | 3 | 0.958 | 0.124 | B1 | NA | NA | NA | NA | NA |
| Tall | Class | Pinopsida | 2 | 49 | 0 | 1.0 | 0.694 | Tall | 2 | 167 | 2 | 0.322 | 1.000 |
| Tall | Class | Polypodiopsida | 16 | 49 | 2 | 0.781 | 0.483 | Tall | 16 | 167 | 6 | 0.968 | 0.090 |
| Tall | Class | Isoetopsida | 2 | 49 | 0 | 1.0 | 0.694 | Tall | 2 | 167 | 0 | 1.000 | 0.186 |
| Tall | Class | Psilotopsida | 5 | 49 | 2 | 0.195 | 0.966 | Tall | 5 | 167 | 4 | 0.282 | 0.942 |
| Tall | Class | Equisetopsida | 2 | 49 | 0 | 1.0 | 0.694 | Tall | 2 | 167 | 0 | 1.000 | 0.186 |
| Tall | Class | Lycopodiopsida | 4 | 49 | 0 | 1.0 | 0.480 | Tall | 4 | 167 | 1 | 0.966 | 0.216 |
| Tall | Class | Magnoliopsida | 186 | 49 | 33 | 0.316 | 0.790 | Tall | 186 | 167 | 106 | 0.514 | 0.582 |
| Tall | Class | Liliopsida | 77 | 49 | 12 | 0.677 | 0.460 | Tall | 77 | 167 | 48 | 0.157 | 0.899 |
| Tf | Class | Pinopsida | 2 | 33 | 0 | 1.0 | 0.788 | Tf | 2 | 142 | 2 | 0.232 | 1.000 |
| Tf | Class | Polypodiopsida | 16 | 33 | 2 | 0.556 | 0.738 | Tf | 16 | 142 | 6 | 0.875 | 0.265 |
| Tf | Class | Isoetopsida | 2 | 33 | 0 | 1.0 | 0.788 | Tf | 2 | 142 | 0 | 1.000 | 0.266 |
| Tf | Class | Psilotopsida | 5 | 33 | 1 | 0.451 | 0.901 | Tf | 5 | 142 | 3 | 0.468 | 0.835 |
| Tf | Class | Equisetopsida | 2 | 33 | 0 | 1.0 | 0.788 | Tf | 2 | 142 | 0 | 1.000 | 0.266 |
| Tf | Class | Lycopodiopsida | 4 | 33 | 0 | 1.0 | 0.620 | Tf | 4 | 142 | 1 | 0.930 | 0.337 |
| Tf | Class | Magnoliopsida | 186 | 33 | 25 | 0.080 | 0.965 | Tf | 186 | 142 | 96 | 0.085 | 0.947 |
| Tf | Class | Liliopsida | 77 | 33 | 5 | 0.965 | 0.089 | Tf | 77 | 142 | 34 | 0.836 | 0.238 |
| B2 | Class | Pinopsida | 2 | 223 | 2 | 0.575 | 1.0 | Ball | 2 | 127 | 0 | 1.000 | 0.322 |
| B2 | Class | Polypodiopsida | 16 | 223 | 10 | 0.938 | 0.162 | Ball | 16 | 127 | 10 | 0.090 | 0.968 |
| B2 | Class | Isoetopsida | 2 | 223 | 2 | 0.575 | 1.0 | Ball | 2 | 127 | 2 | 0.186 | 1.000 |
| B2 | Class | Psilotopsida | 5 | 223 | 2 | 0.987 | 0.093 | Ball | 5 | 127 | 1 | 0.942 | 0.282 |
| B2 | Class | Equisetopsida | 2 | 223 | 0 | 1.0 | 0.058 | Ball | 2 | 127 | 2 | 0.186 | 1.000 |
| B2 | Class | Lycopodiopsida | 4 | 223 | 3 | 0.753 | 0.671 | Ball | 4 | 127 | 3 | 0.216 | 0.966 |
| B2 | Class | Magnoliopsida | 186 | 223 | 142 | 0.450 | 0.658 | Ball | 186 | 127 | 80 | 0.582 | 0.514 |
| B2 | Class | Liliopsida | 77 | 223 | 62 | 0.169 | 0.899 | Ball | 77 | 127 | 29 | 0.899 | 0.157 |
| To | Class | Pinopsida | 2 | 16 | 0 | 1.0 | 0.894 | To | 2 | 25 | 0 | 1.000 | 0.837 |
| To | Class | Polypodiopsida | 16 | 16 | 0 | 1.0 | 0.399 | To | 16 | 25 | 0 | 1.000 | 0.232 |
| To | Class | Isoetopsida | 2 | 16 | 0 | 1.0 | 0.894 | To | 2 | 25 | 0 | 1.000 | 0.837 |
| To | Class | Psilotopsida | 5 | 16 | 1 | 0.246 | 0.975 | To | 5 | 25 | 1 | 0.361 | 0.941 |
| To | Class | Equisetopsida | 2 | 16 | 0 | 1.0 | 0.894 | To | 2 | 25 | 0 | 1.000 | 0.837 |
| To | Class | Lycopodiopsida | 4 | 16 | 0 | 1.0 | 0.799 | To | 4 | 25 | 0 | 1.000 | 0.700 |
| To | Class | Magnoliopsida | 186 | 16 | 8 | 0.917 | 0.192 | To | 186 | 25 | 10 | 0.996 | 0.012 |
| To | Class | Liliopsida | 77 | 16 | 7 | 0.092 | 0.969 | To | 77 | 25 | 14 | 0.001 | 1.000 |
| B1 | Phylum | Lycopodiophyta | 6 | 22 | 1 | 0.376 | 0.933 | B1 | NA | NA | NA | NA | NA |
| B1 | Phylum | Pinophyta | 2 | 22 | 0 | 1.0 | 0.856 | B1 | NA | NA | NA | NA | NA |
| B1 | Phylum | Polypodiophyta | 23 | 22 | 7 | 0.001 | 1.0 | B1 | NA | NA | NA | NA | NA |
| B1 | Phylum | Magnoliophyta | 263 | 22 | 14 | 1.0 | 0.001 | B1 | NA | NA | NA | NA | NA |
| Tall | Phylum | Lycopodiophyta | 6 | 49 | 0 | 1.0 | 0.331 | Tall | 6 | 167 | 1 | 0.994 | 0.056 |
| Tall | Phylum | Pinophyta | 2 | 49 | 0 | 1.0 | 0.694 | Tall | 2 | 167 | 2 | 0.322 | 1.000 |
| Tall | Phylum | Polypodiophyta | 23 | 49 | 4 | 0.555 | 0.668 | Tall | 23 | 167 | 10 | 0.940 | 0.131 |
| Tall | Phylum | Magnoliophyta | 263 | 49 | 45 | 0.383 | 0.798 | Tall | 263 | 167 | 154 | 0.058 | 0.974 |
| Tf | Phylum | Lycopodiophyta | 6 | 33 | 0 | 1.0 | 0.486 | Tf | 6 | 142 | 1 | 0.982 | 0.124 |
| Tf | Phylum | Pinophyta | 2 | 33 | 0 | 1.0 | 0.788 | Tf | 2 | 142 | 2 | 0.232 | 1.000 |
| Tf | Phylum | Polypodiophyta | 23 | 33 | 3 | 0.491 | 0.751 | Tf | 23 | 142 | 9 | 0.872 | 0.243 |
| Tf | Phylum | Magnoliophyta | 263 | 33 | 30 | 0.530 | 0.707 | Tf | 263 | 142 | 130 | 0.174 | 0.907 |
| B2 | Phylum | Lycopodiophyta | 6 | 223 | 5 | 0.553 | 0.813 | Ball | 6 | 127 | 5 | 0.056 | 0.994 |
| B2 | Phylum | Pinophyta | 2 | 223 | 2 | 0.575 | 1.0 | Ball | 2 | 127 | 0 | 1.000 | 0.322 |
| B2 | Phylum | Polypodiophyta | 23 | 223 | 12 | 0.998 | 0.009 | Ball | 23 | 127 | 13 | 0.131 | 0.940 |
| B2 | Phylum | Magnoliophyta | 263 | 223 | 204 | 0.042 | 0.984 | Ball | 263 | 127 | 109 | 0.974 | 0.058 |
| To | Phylum | Lycopodiophyta | 6 | 16 | 0 | 1.0 | 0.713 | To | 6 | 25 | 0 | 1.000 | 0.584 |
| To | Phylum | Pinophyta | 2 | 16 | 0 | 1.0 | 0.894 | To | 2 | 25 | 0 | 1.000 | 0.837 |
| To | Phylum | Polypodiophyta | 23 | 16 | 1 | 0.738 | 0.639 | To | 23 | 25 | 1 | 0.881 | 0.396 |
| To | Phylum | Magnoliophyta | 263 | 16 | 15 | 0.480 | 0.840 | To | 263 | 25 | 24 | 0.230 | 0.946 |

Ball – all non-thermal spp., B1 – non-thermal spp. near the catena, B2 – thermophyte and non-thermal spp. near the catena, Tall – all thermophytes, Tf – facultative thermophytes, To – obligate thermophytes.

## *Table S 16. Selected characteristics of the plant species in the Valley of Geysers*

| Characteristics | | Obligate thermophytes | Facultative thermophytes | Non-heated territories surrounded the transect studied | Distant territories (both non-heated and heated) |
| --- | --- | --- | --- | --- | --- |
| Substrate association | | | | | |
| Petrophyte | no | 16 | 29 | 21 | 201 |
|  | yes | 0 | 1 | 0 | 19 |
| Temperature association | | | | | |
| Thermophyte | | 4 | 1 | 0 | 9 |
| Mesophyte | | 12 | 32 | 22 | 204 |
| Cryophyte | | 0 | 0 | 0 | 10 |
| Substrate moisture | | | | | |
| Hydrophyte | | 0 | 0 | 0 | 3 |
| Hygrophyte | | 6 | 8 | 4 | 81 |
| Mesophyte | | 8 | 21 | 17 | 106 |
| Xerophyte | | 2 | 4 | 1 | 25 |
| Air humidity | | | | | |
| Psychrophyte | no | 15 | 29 | 20 | 143 |
|  | yes | 1 | 1 | 1 | 75 |
| Distribution range | | | | | |
| Only North Pacific | | 5 | 17 | 10 | 73 |
| North Pacific and North America | | 2 | 1 | 1 | 10 |
| North Pacific, North America and Siberia | | 1 | 3 | 0 | 19 |
| North Pacific, North America, Siberia and Asia | | 0 | 2 | 0 | 5 |
| North Pacific, North America and Eurasia | | 8 | 10 | 11 | 116 |
| Distribution pattern | | | | | |
| Plurizonal | | 1 | 1 | 1 | 13 |
| Boreal | | 10 | 26 | 16 | 81 |
| Arctic-boreal | | 3 | 3 | 1 | 23 |
| Hyparctic | | 1 | 2 | 2 | 53 |
| Arctic | | 0 | 0 | 1 | 44 |
| Alpine | no | 12 | 25 | 12 | 90 |
|  | yes | 3 | 6 | 9 | 128 |
| Main distribution area of narrow-ranged species | | | | | |
| To the north | | 1 | 0 | 0 | 4 |
| Kamchatka and Kuril Islands | | 1 | 0 | 0 | 6 |
| To the south | | 1 | 2 | 0 | 7 |

## *Table S 17. Spearman’s coefficients of correlation between cover values of facultative thermophyte plant species and soil properties (n=12) within the studied catena*

| Soil properties | *Aconitum fischeri* | *Allium ochotense* | *Angelica lucida* | *Artemisia vulgaris* | *Aruncus dioicus* | *Calamagrostis purpurea* | *Cirsium kamtschaticum* | *Dactylorhiza aristata* | *Epilobium ciliatum* | *Filipendula camtschatica* | *Galium boreale* | *Geranium pratense* | *Maianthemum dilatatum* | *Ophioglossum vulgatum* | *Pedicularis resupinata* | *Picris hieracioides* | *Pteridium aquilinum* | *Senecio cannabifolius* | *Thalictrum minus* | *Viola selkirkii* |
| --- | --- | --- | --- | --- | --- | --- | --- | --- | --- | --- | --- | --- | --- | --- | --- | --- | --- | --- | --- | --- |
| Zone | -0.79 | -0.09 | -0.35 | -0.52 | -0.52 | 0.20 | -0.35 | -0.38 | -0.38 | -0.09 | -0.42 | -0.54 | -0.79 | -0.09 | -0.40 | -0.35 | -0.59 | -0.77 | -0.76 | -0.42 |
| T | -0.74 | -0.22 | -0.37 | -0.41 | -0.59 | -0.04 | -0.50 | -0.45 | -0.52 | -0.13 | -0.31 | -0.50 | -0.75 | -0.22 | -0.39 | -0.32 | -0.62 | -0.79 | -0.71 | -0.31 |
| PM_50–1000_ | 0.79 | -0.22 | 0.26 | 0.48 | 0.30 | -0.11 | 0.08 | 0.19 | 0.06 | 0.13 | 0.39 | 0.46 | 0.76 | -0.22 | 0.17 | 0.50 | 0.40 | 0.58 | ***0.80*** | 0.39 |
| PM_1–50_ | -0.74 | 0.48 | 0.01 | -0.43 | -0.07 | 0.15 | 0.17 | 0.00 | 0.13 | -0.04 | -0.39 | -0.25 | -0.76 | 0.48 | 0.01 | -0.38 | -0.18 | -0.43 | ***-0.71*** | -0.39 |
| PM_<1_ | -0.82 | -0.13 | -0.36 | -0.41 | -0.57 | 0.02 | -0.44 | -0.45 | -0.39 | -0.22 | -0.31 | -0.49 | -0.76 | -0.13 | -0.33 | -0.42 | -0.64 | -0.76 | -0.72 | -0.31 |
| pH | 0.48 | 0.48 | 0.79 | 0.19 | ***0.79*** | 0.08 | 0.67 | 0.39 | 0.45 | 0.39 | 0.31 | 0.63 | 0.29 | 0.48 | 0.57 | 0.43 | 0.70 | 0.54 | ***0.58*** | 0.31 |
| EC | -0.11 | -0.13 | -0.35 | -0.70 | -0.05 | -0.05 | 0.04 | -0.13 | -0.26 | 0.39 | -0.31 | -0.61 | -0.34 | -0.13 | -0.33 | -0.13 | -0.11 | -0.38 | -0.50 | -0.31 |
| Eh | -0.36 | -0.48 | -0.60 | -0.51 | -0.40 | -0.01 | -0.41 | -0.65 | -0.32 | -0.13 | -0.04 | -0.69 | -0.29 | -0.48 | -0.36 | -0.51 | -0.67 | -0.56 | -0.43 | -0.04 |
| HCO_3_^–^ | 0.46 | 0.22 | 0.70 | 0.41 | 0.57 | -0.20 | 0.57 | 0.26 | 0.39 | 0.39 | 0.04 | 0.55 | 0.30 | 0.22 | 0.18 | 0.67 | 0.43 | 0.38 | 0.77 | 0.04 |
| TiO_2_ | -0.62 | -0.13 | -0.23 | -0.21 | -0.42 | 0.16 | -0.40 | -0.45 | -0.26 | -0.31 | -0.04 | -0.28 | -0.51 | -0.13 | -0.12 | -0.41 | -0.55 | -0.54 | -0.45 | -0.04 |
| V | -0.56 | -0.13 | -0.20 | -0.53 | -0.39 | 0.30 | -0.46 | -0.45 | -0.26 | -0.39 | 0.13 | -0.18 | -0.41 | -0.13 | 0.02 | -0.47 | -0.49 | -0.44 | -0.39 | 0.13 |
| Cr | -0.22 | -0.13 | -0.14 | -0.12 | -0.05 | 0.02 | -0.17 | -0.45 | 0.06 | -0.31 | 0.31 | -0.14 | -0.05 | -0.13 | 0.16 | -0.48 | -0.36 | -0.15 | -0.05 | 0.31 |
| MnO | 0.46 | 0.31 | 0.57 | 0.40 | 0.64 | -0.04 | 0.46 | 0.19 | 0.52 | 0.04 | 0.48 | 0.59 | 0.47 | 0.31 | 0.59 | 0.17 | 0.46 | 0.59 | 0.66 | 0.48 |
| Fe_2_O_3_ | 0.13 | 0.04 | 0.27 | 0.24 | 0.33 | -0.08 | 0.17 | -0.19 | 0.26 | -0.04 | 0.39 | 0.21 | 0.18 | 0.04 | 0.34 | -0.09 | 0.03 | 0.16 | 0.36 | 0.39 |
| Co | -0.35 | -0.13 | -0.05 | -0.16 | -0.17 | -0.26 | -0.27 | -0.45 | -0.06 | -0.31 | 0.22 | -0.06 | -0.20 | -0.13 | 0.09 | -0.35 | -0.41 | -0.28 | -0.08 | 0.22 |
| Ni | 0.03 | -0.39 | -0.49 | 0.21 | -0.47 | -0.65 | -0.41 | -0.06 | -0.19 | -0.31 | -0.13 | -0.20 | 0.23 | -0.39 | -0.37 | -0.06 | -0.29 | -0.03 | -0.02 | -0.13 |
| Cu | -0.53 | -0.31 | -0.12 | -0.10 | -0.40 | -0.17 | -0.42 | -0.58 | -0.39 | -0.13 | -0.04 | -0.25 | -0.51 | -0.31 | -0.24 | -0.17 | -0.59 | -0.64 | -0.25 | -0.04 |
| Zn | 0.12 | -0.22 | -0.34 | 0.01 | 0.06 | -0.53 | 0.13 | -0.19 | 0.13 | 0.13 | -0.13 | -0.44 | 0.12 | -0.22 | -0.25 | -0.17 | -0.20 | -0.06 | -0.01 | -0.13 |
| As | -0.58 | -0.48 | -0.55 | -0.21 | -0.79 | -0.22 | -0.65 | -0.45 | -0.58 | -0.22 | -0.39 | -0.53 | -0.50 | -0.48 | -0.64 | -0.17 | -0.72 | -0.73 | -0.51 | -0.39 |
| Pb | -0.58 | -0.48 | -0.55 | -0.21 | -0.79 | -0.22 | -0.65 | -0.45 | -0.58 | -0.22 | -0.39 | -0.53 | -0.50 | -0.48 | -0.64 | -0.17 | -0.72 | -0.73 | -0.51 | -0.39 |
| CaO | 0.78 | 0.22 | 0.38 | 0.34 | 0.61 | -0.03 | 0.40 | 0.45 | 0.39 | 0.13 | 0.48 | 0.54 | 0.75 | 0.22 | 0.53 | 0.26 | 0.71 | 0.81 | 0.63 | 0.48 |
| Al_2_O_3_ | -0.77 | -0.22 | -0.30 | -0.30 | -0.56 | 0.10 | -0.45 | -0.52 | -0.45 | -0.13 | -0.31 | -0.48 | -0.76 | -0.22 | -0.39 | -0.30 | -0.67 | -0.81 | -0.63 | -0.31 |
| SiO_2_ | -0.54 | -0.13 | 0.09 | 0.05 | -0.30 | 0.16 | -0.18 | -0.39 | -0.26 | 0.04 | -0.31 | -0.14 | -0.59 | -0.13 | -0.33 | 0.11 | -0.48 | -0.63 | -0.16 | -0.31 |
| P_2_O_5_ | 0.65 | 0.22 | -0.10 | 0.03 | 0.47 | 0.25 | 0.39 | 0.45 | 0.52 | -0.04 | 0.31 | 0.13 | 0.75 | 0.22 | 0.39 | -0.17 | 0.54 | 0.79 | 0.26 | 0.31 |
| K_2_O | 0.10 | 0.22 | 0.56 | 0.59 | 0.39 | -0.10 | 0.42 | 0.06 | 0.26 | 0.31 | -0.04 | 0.33 | -0.05 | 0.22 | 0.11 | 0.41 | 0.18 | 0.06 | 0.41 | -0.04 |
| MgO | 0.41 | 0.22 | 0.72 | 0.45 | 0.57 | -0.10 | 0.40 | 0.13 | 0.39 | 0.13 | 0.39 | 0.68 | 0.37 | 0.22 | 0.46 | 0.41 | 0.39 | 0.45 | ***0.78*** | 0.39 |
| Sr | -0.68 | -0.22 | -0.09 | -0.19 | -0.43 | 0.24 | -0.34 | -0.52 | -0.45 | 0.04 | -0.31 | -0.35 | -0.76 | -0.22 | -0.39 | -0.06 | -0.59 | -0.81 | -0.43 | -0.31 |
| LOI | 0.56 | 0.26 | -0.10 | 0.34 | 0.38 | 0.07 | 0.36 | 0.55 | 0.39 | 0.04 | 0.13 | 0.09 | 0.58 | 0.26 | 0.28 | -0.07 | 0.57 | 0.68 | 0.10 | 0.13 |
| SOC | 0.75 | 0.31 | 0.08 | -0.12 | 0.58 | 0.10 | 0.42 | 0.58 | 0.39 | 0.13 | 0.39 | 0.26 | 0.71 | 0.31 | 0.52 | -0.02 | ***0.78*** | ***0.84*** | 0.25 | 0.39 |
| Kaolinite | -0.32 | -0.31 | -0.68 | -0.54 | -0.40 | 0.18 | -0.24 | -0.29 | -0.29 | 0.04 | -0.39 | -0.78 | -0.34 | -0.31 | -0.52 | -0.37 | -0.46 | -0.50 | -0.63 | -0.39 |
| Smectite | 0.38 | 0.04 | 0.43 | 0.70 | 0.36 | -0.36 | 0.37 | 0.16 | 0.16 | 0.39 | -0.04 | 0.29 | 0.21 | 0.04 | -0.01 | 0.57 | 0.29 | 0.20 | 0.53 | -0.04 |
| Microcline | 0.48 | -0.13 | 0.45 | 0.64 | 0.29 | -0.47 | 0.29 | 0.10 | 0.10 | 0.39 | -0.04 | 0.36 | 0.32 | -0.13 | -0.12 | 0.74 | 0.21 | 0.20 | 0.75 | -0.04 |
| Albite | 0.27 | 0.22 | 0.59 | 0.66 | 0.30 | -0.24 | 0.18 | 0.23 | 0.23 | -0.04 | 0.31 | 0.68 | 0.29 | 0.22 | 0.39 | 0.37 | 0.35 | 0.40 | ***0.58*** | 0.31 |
| Anorthite | 0.71 | 0.22 | 0.38 | 0.47 | 0.51 | -0.10 | 0.42 | 0.49 | 0.49 | 0.04 | 0.31 | 0.58 | 0.75 | 0.22 | 0.39 | 0.35 | 0.61 | 0.80 | 0.73 | 0.31 |
| Clinoptilolite | -0.18 | -0.31 | -0.10 | 0.34 | -0.21 | -0.59 | -0.08 | -0.29 | -0.29 | 0.31 | -0.39 | -0.34 | -0.34 | -0.31 | -0.52 | 0.24 | -0.35 | -0.50 | -0.08 | -0.39 |
| Heulandite | 0.01 | -0.28 | 0.25 | -0.32 | 0.03 | 0.07 | -0.26 | -0.41 | -0.41 | 0.14 | 0.42 | 0.13 | -0.13 | -0.28 | 0.15 | 0.11 | -0.09 | -0.24 | 0.16 | 0.42 |
| Anatase | -0.63 | -0.13 | -0.38 | -0.50 | -0.39 | -0.05 | -0.20 | -0.36 | -0.36 | 0.13 | -0.48 | -0.66 | -0.74 | -0.13 | -0.47 | -0.26 | -0.51 | -0.75 | -0.72 | -0.48 |
| Pyrite | 0.63 | 0.22 | 0.12 | -0.15 | 0.44 | 0.18 | 0.26 | 0.42 | 0.42 | -0.13 | 0.48 | 0.39 | 0.74 | 0.22 | 0.53 | -0.06 | 0.57 | 0.80 | 0.41 | 0.48 |
| Quartz | -0.54 | 0.04 | 0.08 | -0.10 | -0.16 | -0.01 | 0.04 | -0.23 | -0.23 | 0.31 | -0.48 | -0.31 | -0.74 | 0.04 | -0.36 | 0.11 | -0.31 | -0.65 | -0.40 | -0.48 |
| Cristobalite | -0.09 | 0.48 | 0.61 | -0.11 | 0.53 | -0.02 | 0.33 | 0.03 | 0.13 | 0.22 | 0.39 | 0.34 | -0.27 | 0.48 | 0.64 | -0.06 | 0.37 | 0.06 | -0.04 | 0.39 |
| Hematite | -0.49 | -0.32 | -0.26 | -0.30 | -0.40 | -0.47 | -0.31 | -0.47 | -0.47 | 0.14 | -0.32 | -0.50 | -0.60 | -0.32 | -0.47 | -0.08 | -0.53 | -0.73 | -0.44 | -0.32 |
| Goethite | -0.09 | -0.17 | 0.40 | 0.61 | 0.02 | -0.25 | 0.04 | -0.26 | -0.26 | 0.40 | -0.17 | 0.10 | -0.33 | -0.17 | -0.25 | 0.54 | -0.15 | -0.39 | 0.26 | -0.17 |
| Na^+^ | -0.40 | 0.04 | -0.43 | -0.11 | -0.54 | 0.34 | -0.28 | 0.19 | -0.13 | -0.31 | -0.48 | -0.25 | -0.24 | 0.04 | -0.35 | -0.17 | -0.22 | -0.19 | -0.52 | -0.48 |
| NH_4_^+^ | 0.03 | -0.28 | -0.25 | -0.44 | 0.04 | 0.15 | -0.10 | -0.41 | -0.10 | 0.05 | 0.23 | -0.35 | 0.01 | -0.28 | 0.00 | -0.32 | -0.23 | -0.17 | -0.08 | 0.23 |
| K^+^ | 0.78 | 0.13 | 0.08 | 0.16 | 0.65 | -0.16 | 0.49 | 0.32 | 0.45 | 0.22 | 0.39 | 0.15 | 0.73 | 0.13 | 0.40 | 0.01 | 0.58 | 0.73 | 0.44 | 0.39 |
| Mg^2+^ | 0.70 | 0.22 | -0.03 | 0.03 | 0.55 | 0.25 | 0.56 | 0.52 | 0.45 | 0.31 | 0.04 | 0.01 | 0.61 | 0.22 | 0.18 | 0.09 | 0.61 | 0.66 | 0.24 | 0.04 |
| Ca^2+^ | 0.17 | -0.04 | 0.22 | 0.11 | 0.07 | 0.35 | 0.16 | 0.06 | 0.19 | 0.04 | -0.13 | 0.24 | 0.20 | -0.04 | -0.13 | 0.39 | 0.00 | 0.13 | 0.47 | -0.13 |
| F^–^ | -0.20 | -0.48 | -0.25 | -0.10 | -0.22 | -0.01 | -0.12 | -0.52 | -0.45 | 0.48 | -0.39 | -0.63 | -0.46 | -0.48 | -0.64 | 0.16 | -0.48 | -0.71 | -0.22 | -0.39 |
| Cl^–^ | 0.05 | -0.31 | -0.54 | -0.19 | -0.33 | 0.11 | -0.27 | 0.00 | -0.32 | 0.04 | -0.22 | -0.45 | 0.02 | -0.31 | -0.38 | -0.10 | -0.12 | -0.14 | -0.34 | -0.22 |
| NO_3_^–^ | -0.59 | -0.39 | -0.52 | -0.36 | -0.56 | -0.16 | -0.38 | -0.52 | -0.45 | 0.04 | -0.48 | -0.73 | -0.63 | -0.39 | -0.65 | -0.23 | -0.70 | ***-0.81*** | -0.59 | -0.48 |
| HPO_4_^2–^ | -0.07 | -0.21 | -0.07 | 0.01 | 0.08 | -0.31 | 0.10 | -0.31 | -0.31 | 0.57 | -0.21 | -0.48 | -0.39 | -0.21 | -0.31 | 0.10 | -0.14 | -0.48 | -0.24 | -0.21 |
| SO_4_^2–^ | -0.46 | 0.04 | -0.54 | -0.67 | -0.27 | 0.41 | -0.07 | -0.13 | 0.00 | -0.13 | -0.35 | -0.61 | -0.39 | 0.04 | -0.25 | -0.55 | -0.34 | -0.35 | -0.70 | -0.35 |

Coefficients with P-values <0.05 are marked as follows: red – positive values of Spearman’s correlation coefficients, blue – negative values of Spearman’s correlation coefficients, bold italic – Hoeffding’s coefficients (not presented in this table). Т – temperature at the depth of 15 cm.

## *Table S 18. Spearman’s coefficients of correlation between cover values of non-thermal and obligate thermophyte plant species and soil properties (n=12) within the studied catena*

| Soil properties | Non-thermal | | | Obligate thermophytes | | | | | |
| --- | --- | --- | --- | --- | --- | --- | --- | --- | --- |
|  | *Carex longirostrata* | *Equisetum sp.* | *Heracleum lanatum* | *Agrostis geminata* | *Fimbristylis dichotoma* | *Moehringia lateriflora* | *Potentilla fragarioides* | *Sedum telephium* |  |
| Zone | -0.79 | -0.42 | -0.42 | 0.48 | 0.62 | -0.21 | 0.07 | -0.09 |  |
| T | -0.73 | -0.48 | -0.39 | 0.43 | 0.60 | -0.18 | 0.15 | -0.04 |  |
| PM_50–1000_ | 0.75 | 0.31 | 0.48 | -0.29 | -0.51 | 0.04 | 0.05 | 0.22 |  |
| PM_1–50_ | -0.75 | -0.31 | -0.48 | -0.03 | 0.51 | 0.19 | 0.03 | -0.13 |  |
| PM_<1_ | -0.73 | -0.39 | -0.48 | 0.43 | 0.57 | -0.18 | 0.05 | -0.04 |  |
| pH | 0.33 | 0.13 | 0.04 | -0.57 | -0.44 | 0.55 | 0.12 | 0.22 |  |
| EC | -0.38 | -0.22 | -0.04 | 0.36 | 0.40 | -0.40 | -0.10 | -0.48 |  |
| Eh | -0.23 | 0.04 | -0.39 | 0.52 | 0.49 | -0.64 | -0.21 | -0.31 |  |
| HCO_3_^–^ | 0.29 | 0.31 | 0.13 | -0.65 | -0.33 | 0.48 | 0.18 | 0.48 |  |
| TiO_2_ | -0.44 | -0.22 | -0.48 | 0.57 | 0.12 | -0.11 | -0.30 | 0.04 |  |
| V | -0.31 | -0.22 | -0.48 | 0.59 | 0.34 | -0.47 | -0.40 | 0.04 |  |
| Cr | 0.07 | 0.22 | -0.48 | -0.16 | 0.42 | -0.22 | -0.28 | -0.04 |  |
| MnO | 0.56 | 0.39 | -0.04 | -0.57 | -0.44 | 0.40 | 0.07 | 0.22 |  |
| Fe_2_O_3_ | 0.29 | 0.31 | -0.31 | -0.43 | -0.01 | 0.23 | -0.08 | 0.13 |  |
| Co | -0.09 | 0.04 | -0.48 | 0.03 | 0.31 | -0.19 | -0.38 | 0.13 |  |
| Ni | 0.16 | 0.13 | 0.31 | -0.06 | 0.18 | -0.36 | 0.01 | -0.04 |  |
| Cu | -0.44 | -0.22 | -0.48 | 0.36 | 0.18 | -0.02 | -0.13 | 0.22 |  |
| Zn | 0.11 | 0.39 | -0.04 | -0.06 | 0.07 | -0.18 | -0.32 | -0.31 |  |
| As | -0.54 | -0.31 | -0.13 | 0.43 | 0.56 | -0.38 | 0.09 | 0.04 |  |
| Pb | -0.54 | -0.31 | -0.13 | 0.43 | 0.56 | -0.38 | 0.09 | 0.04 |  |
| CaO | 0.76 | 0.31 | 0.39 | -0.50 | -0.50 | 0.19 | -0.13 | -0.04 |  |
| Al_2_O_3_ | -0.73 | -0.39 | -0.48 | 0.51 | 0.39 | -0.07 | 0.02 | 0.04 |  |
| SiO_2_ | -0.58 | -0.22 | -0.39 | 0.57 | -0.24 | 0.26 | 0.44 | 0.39 |  |
| P_2_O_5_ | 0.73 | 0.48 | 0.39 | -0.16 | -0.28 | -0.26 | -0.54 | -0.48 |  |
| K_2_O | -0.03 | 0.13 | -0.13 | -0.57 | -0.31 | 0.83 | 0.51 | 0.39 |  |
| MgO | 0.44 | 0.31 | -0.04 | -0.57 | -0.44 | 0.47 | 0.18 | 0.48 |  |
| Sr | -0.73 | -0.39 | -0.48 | 0.35 | 0.37 | 0.12 | 0.13 | 0.22 |  |
| LOI | 0.53 | 0.26 | 0.48 | -0.31 | -0.43 | 0.19 | 0.03 | -0.48 |  |
| SOC | 0.70 | 0.22 | 0.48 | -0.37 | -0.20 | -0.18 | -0.52 | -0.48 |  |
| Kaolinite | -0.39 | -0.09 | -0.09 | 0.65 | 0.42 | -0.55 | -0.27 | -0.48 |  |
| Smectite | 0.17 | 0.17 | 0.17 | -0.65 | -0.40 | 0.74 | 0.57 | 0.31 |  |
| Microcline | 0.27 | 0.26 | 0.26 | -0.59 | -0.38 | 0.49 | 0.54 | 0.48 |  |
| Albite | 0.33 | 0.09 | 0.09 | -0.47 | -0.57 | 0.63 | 0.21 | 0.48 |  |
| Anorthite | 0.73 | 0.44 | 0.44 | -0.57 | -0.45 | 0.18 | -0.03 | 0.13 |  |
| Clinoptilolite | -0.39 | -0.09 | -0.09 | -0.12 | 0.05 | 0.42 | 0.39 | 0.13 |  |
| Heulandite | -0.01 | -0.28 | -0.28 | 0.07 | 0.26 | -0.15 | 0.02 | 0.23 |  |
| Anatase | -0.76 | -0.35 | -0.35 | 0.24 | 0.68 | -0.15 | 0.08 | -0.22 |  |
| Pyrite | 0.76 | 0.35 | 0.35 | 0.12 | -0.51 | -0.42 | -0.70 | -0.22 |  |
| Quartz | -0.76 | -0.35 | -0.35 | -0.18 | 0.41 | 0.41 | 0.68 | 0.13 |  |
| Cristobalite | -0.14 | -0.31 | -0.44 | -0.50 | -0.05 | 0.61 | 0.24 | 0.04 |  |
| Hematite | -0.60 | -0.32 | -0.32 | 0.08 | 0.61 | -0.10 | 0.22 | 0.00 |  |
| Goethite | -0.33 | -0.17 | -0.17 | -0.25 | -0.33 | 0.85 | 0.75 | 0.52 |  |
| Na^+^ | -0.35 | -0.22 | 0.22 | 0.40 | 0.32 | -0.24 | 0.08 | -0.13 |  |
| NH_4_^+^ | 0.09 | 0.14 | -0.28 | 0.56 | -0.11 | -0.47 | -0.49 | -0.28 |  |
| K^+^ | 0.75 | 0.48 | 0.31 | -0.57 | -0.24 | 0.01 | -0.20 | -0.39 |  |
| Mg^2+^ | 0.54 | 0.39 | 0.48 | -0.16 | -0.38 | -0.07 | -0.38 | -0.48 |  |
| Ca^2+^ | 0.16 | 0.31 | 0.13 | 0.18 | -0.29 | -0.14 | -0.38 | 0.39 |  |
| F^–^ | -0.49 | -0.13 | -0.22 | 0.34 | 0.08 | 0.07 | 0.50 | -0.04 |  |
| Cl^–^ | -0.06 | -0.13 | 0.31 | -0.01 | 0.56 | -0.38 | -0.05 | -0.39 |  |
| NO_3_^–^ | -0.66 | -0.22 | -0.31 | 0.25 | 0.73 | -0.29 | 0.25 | -0.13 |  |
| HPO_4_^2–^ | -0.39 | -0.21 | -0.21 | 0.01 | -0.03 | 0.42 | 0.73 | -0.21 |  |
| SO_4_^2–^ | -0.41 | -0.04 | -0.22 | 0.65 | 0.40 | -0.55 | -0.44 | -0.48 |  |

Coefficients with P-values <0.05 are marked as follows: red – positive values of Spearman’s correlation coefficients, blue – negative values of Spearman’s correlation coefficients, bold italic – Hoeffding’s coefficients (not presented in this table). Т – temperature at the depth of 15 cm.

## *Table S 19. Plant groups predetermined by edaphic conditions at the study site. Results of a comparative analysis of plant distribution and Andosol properties along the studied botanical transect/soil catena.*

| Group | Subgroup | Species |
| --- | --- | --- |
| Mesophytes that prefer cool sandy soils (predominantly in Erman’s birch woods and herbaceous plant communities of Kamchatka) | Non-thermal (Far-East boreal species) | *Aconitum fischeri, Carex longirostrata, Senecio cannabifolius* |
|  | Thermophytes (mostly boreal species) | *Aruncus dioicus, Maianthemum dilatatum, Pteridium aquilinum, Thalictrum minus* |
| Mesophytes that prefer rather acidic than neutral soils with high contents of Mn and SOC, but low contents of NO_3_^–^, Al, As, Pb (and to a lesser degree Sr) and clay minerals, some species (marked in bold) can tolerate high contents of pyrite, other species (marked in red) are suppressed by sulfates and high electrical conductivity (occur in Erman’s birch woods and herbaceous communities of Kamchatka) | Non-thermal (Far-East boreal species) | ***Aconitum fischeri, Carex longirostrata,*** *Senecio cannabifolius* |
|  | Thermophytes (mostly North Pacific species) that occur in Kamchatka’s shrublands | *Pteridium aquilinum, Thalictrum minus, Angelica gmelinii, Artemisia opulenta, Aruncus dioicus, Cirsium kamtschaticum, Geranium erianthum,* ***Maianthemum dilatatum*** |
| Non-thermal species that prefer soils of heavier texture | | *Heracleum lanatum, Pedicularis resupinata** |
| Obligate thermophytes that prefer soils enriched in NO_3_^–^ and hematite | | *Fimbristylis ochotensis* |
| Obligate thermophytes that requite high contents of P, Si (quartz) and goethite, but low sulfate and electrical conductivity | | *Potentilla stolonifera* |
| Obligate thermophytes that prefer soils enriched in SOC | | *Dactylorhiza aristata* |
| Obligate thermophytes sensitive to high As and Pb contents | | *Epilobium glandulosum* |
| Facultative thermophytes that prefer soils with a low carbonate alkalinity and feldspar contents, but high EC, sulfate and kaolinite contents | | *Agrostis geminata* |
| Facultative thermophytes that prefer soils with a high carbonate alkalinity | | *Picris hieracioides* |
| Species without obvious requirements for edaphic factors | Non-thermal | *Calamagrostis purpurea* |
|  | Obligate thermophytes | *Moehringia lateriflora* |
| Mostly boreal species without significant dependence from edaphic factors in Eurasia and North America | Non-thermal | *Equisetum* sp*.* |
|  | Thermophytes | *Allium ochotense, Filipendula camtschatica, Galium boreale, Ophioglossum vulgatum, Sedum telephium, Viola selkirkii* |

*Prefers substrates with relatively high contents of cristobalite and Mn and low contents of clay minerals, bulk As and Pb and water-soluble F and NO_3_^–^.

## *Table S 20. Chemical composition of a free gas (vol. %), which characterized feeding reservoir of the Velikan geyser* ^1^

| H_2_ | Ar | O_2_ | N_2_ | CO_2_ | CO | CH_4_ | H_2_S |
| --- | --- | --- | --- | --- | --- | --- | --- |
| ≤0.45 | ≤0.9 | 0.02–6.6 | 32–49 | 33–62 | 0.003–0.3 | 6–9 | ≤0.03 |

Sampling was performed by A. V. Kiryukhin in September 2013 from hot degassing pool located 8 m from the Velikan geyser (N=2)

## *Table S 21. Characteristics of geysers and water bodies near the studied transect*

| Object | Coordinates ^2^ | | Selected features of water, mg/dm^3^ | | | | | | | | | Date | References |
| --- | --- | --- | --- | --- | --- | --- | --- | --- | --- | --- | --- | --- | --- |
|  | N | E | pH | HCO_3_^–^ | CO_3_^2–^ | Cl^−^ | SO_4_^2–^ | Na^+^ | K^+^ | Ca^2+^ | Mg^2+^ |  |  |
| Bolshoy | 54°26'15.7" | 160°8'11.9" | NA | 59 | | 794 | 163 | 555 | 37 | 23 | NA | ≤2009 | ^3^ |
|  |  |  | 8.6 | 46 | 6 | 610 | 192 | 447 | 37 | 22 | <0,1 | 02.09.2015 | ^1^ |
| Grotik | 54°26'16.8" | 160°8'25.4" | NA | NA | NA | NA | NA | NA | NA | NA | NA | NA |  |
| Kotly | 54°26'15.1" | 160°8'26.9" | NA | NA | NA | NA | NA | NA | NA | NA | NA | NA |  |
| Shchel | 54°26'17.8" | 160°8'23.5" | NA | 61 | | 823 | 144 | 546 | 44 | 18 | NA | ≤2009 | ^3^ |
| Vanna | 54°26'15.1" | 160°8'27.6" | NA | NA | NA | NA | NA | NA | NA | NA | NA | NA |  |
| Vrata Ada | 54°26'15.6" | 160°8'25.1" | NA | NA | NA | NA | NA | NA | NA | NA | NA | NA |  |
| Kotel krasny | 54°26'15,6" | 160°08'22,7" | NA | NA | NA | NA | NA | NA | NA | NA | NA | NA |  |
| Utinoye lake | 54°26'13,7" | 160°08'21,9" | NA | NA | NA | NA | NA | NA | NA | NA | NA | NA |  |
| Teploye lake | 54°26'14,6" | 160°08'22,6" | NA | NA | NA | NA | NA | NA | NA | NA | NA | NA |  |

Objects are named according to ^2,4^. NA – not available.

## *Table S 22. Methods of chemical analyses*

| Parameters | Methods | Device | References |
| --- | --- | --- | --- |
| Soil organic carbon (SOC) | Titrimetry, dichromate digestion | – | ^5^ |
| рН | Potentiometry, 1:2.5 soil:water extract, filtration | Expert-pH, Russia | ^6^ |
| Grain-size fractions | Laser diffraction on samples pre-treated with 4% Na_4_P_2_O_7_ | Analysette 22 (Fritsch, Germany) | ^7^ |
| Total content of elements | A X-Ray fluorescence spectrometry, 30% of samples in duplication | Spectroscan Maks-GV, Russia | – |
| Eh | Potentiometry in soil paste | HI 98120, Germany | – |
| Electrical conductivity | Conductometry, 1:5 soil:water extract | Hanna HI 98331, Germany | – |
| Basicity (HCO_3_^−^) | Titrimetry with 0.02М H_2_SO_4_, 1:5 soil:water extract after filtration | – | ^8^ |
| Cation-anion composition (Ca^2+^, Mg^2+^, Na^+^, NH_4_^+^, K^+^, NO_3_^–^, NO_2_^–^, SO_4_^2–^, Cl^–^) | Water extracts (soil:solution ratio of 1:5) were analyzed by high performance liquid chromatography after filtration | Styer chromatograph (Aquilon, Russia) | – |
| Exchangeable fraction | 1М NH_4_Ac (рН 7,0), AES-ICP after filtration | iCAP-6500 (Thermo Scientific, USA) | ^9^ |
| Short-range-order Al, Fe and Si compounds (Al_ox_, Fe_ox_, Si_ox_) | Acid (pH 3.0) 0.2M ammonium oxalate solution, natural light ^10^, AES-ICP after filtration | iCAP-6500 (Thermo Scientific, USA) | ^11^ |

## *Table S 23. Methods of mineral analyses*

| Type of analysis | Methods | Device and samples | References |
| --- | --- | --- | --- |
| Bulk mineral analysis | X-Ray powder diffractions | Nonoriented samples of bulk samples. Quantitative analysis was carried out by the Rietveld method with PROFEX GUI for BGMN | ^12–14^ |
| Clay mineral analysis | X-Ray diffraction in clay fraction samples: investigation of expandabale minerals | Oriented samples of clay fraction (<1 µm) in air-dried and ethylene glycole colvation | ^12^ |
|  | X-Ray diffraction in clay fraction samples: investigation of kaolinites’ ordering/disordering | Powder samples from non-oriented samples for investigation di-tri species of clay minerals and comparable degree of deffects (ordering/disordering) | ^15^ |

ULTIMA‑IV (Rigaku, Japan) X-Ray diffractometer was operated at 40 kV, 40 mA, 3–65° 2θ; Cu radiation equiped a DTex/Ultra semiconductor detector for bulk and clay mineral analysis.

## *Table S 24. Cover values (%) of vascular plants recorded within the studied catena*

| Species | Points | | | | | | | | | | | | | | | |
| --- | --- | --- | --- | --- | --- | --- | --- | --- | --- | --- | --- | --- | --- | --- | --- | --- |
|  | 1 | 2 | 3 | 4 | 5 | 6 | 7 | 8 | 9 | 10 | 11 | 12 | 13 | 14 | 15 | 16 |
| *Aconitum fischeri* | – | – | – | – | – | – | – | – | – | – | – | 2 | – | 5 | 8 | <1 |
| *Agrostis geminata* | – | – | – | – | <1 | 50 | – | – | – | – | – | – | – | – | – | – |
| *Allium ochotense* | – | – | – | – | – | – | – | – | – | – | – | – | <1 | – | – | – |
| *Angelica gmelinii* | – | – | – | – | – | – | – | – | – | – | 15 | 6 | 1– | <1 | – | – |
| *Artemisia opulenta* | – | – | – | – | – | – | – | – | – | 39 | 10 | – | – | – | <1 | <1 |
| *Aruncus dioicus* | – | – | – | – | – | – | – | – | – | – | – | 60 | 50 | 20 | – | 5 |
| *Calamagrostis purpurea* | – | – | – | – | – | 25 | – | – | – | – | – | – | <1 | – | – | – |
| *Carex longirostrata* | – | – | – | – | – | – | – | – | – | – | – | – | – | 50 | 30 | 30 |
| *Cirsium kamtschaticum* | – | – | – | – | – | – | – | – | – | – | – | 15 | 20 | – | – | <1 |
| *Dactylorhiza aristata* | – | – | – | – | – | – | – | – | – | – | – | – | <1 | – | <1 | – |
| *Epilobium glandulosum* | – | – | – | – | – | – | – | – | – | – | – | – | <1 | – | – | <1 |
| *Equisetum sp.* | – | – | – | – | – | – | – | – | – | – | – | – | – | – | – | 20 |
| *Filipendula camtschatica* | – | – | – | – | – | – | – | – | – | – | – | 10 | – | – | – | – |
| *Fimbristylis ochotensis* | – | – | – | – | – | – | <1 | 10 | 50 | – | – | – | – | – | – | – |
| *Galium boreale* | – | – | – | – | – | – | – | – | – | – | – | – | – | <1 | – | – |
| *Geranium erianthum* | – | – | – | – | – | – | – | – | – | – | 15 | – | 5 | 5 | <1 | – |
| *Heracleum lanatum* | – | – | – | – | – | – | – | – | – | – | – | – | – | – | 2 | – |
| *Senecio cannabifolius* | – | – | – | – | – | – | – | – | – | – | – | – | 5 | 5 | 30 | <1 |
| *Maianthemum dilatatum* | – | – | – | – | – | – | – | – | – | – | – | – | – | 5 | 20 | 5 |
| *Moehringia lateriflora* | – | – | – | – | – | – | – | – | – | 1 | <1 | <1 | <1 | – | – | – |
| *Ophioglossum alaskinum* | – | – | – | – | – | – | – | – | – | – | – | – | <1 | – | – | – |
| *Pedicularis resupinata* | – | – | – | – | – | – | – | – | – | – | – | – | <1 | 2 | – | – |
| *Potentilla stolonifera* | – | – | – | – | – | – | – | 10 | – | 10 | 1 | 1 | – | – | – | – |
| *Pteridium aquilinum* | – | – | – | – | – | – | – | – | – | – | – | 2 | 10 | 5 | 8 | – |
| *Picris kamtschatica* | – | – | – | – | – | – | – | – | – | – | 8 | 2 | – | – | <1 | – |
| *Sedum telephium* | – | – | – | – | – | – | – | – | – | – | 1 | – | – | – | – | – |
| *Thalictrum minus* | – | – | – | – | – | – | – | – | – | – | 50 | 2 | – | 5 | 2 | 10 |
| *Viola selkirkii* | – | – | – | – | – | – | – | – | – | – | – | – | – | <1 | – | – |

# **Supplementary figures**


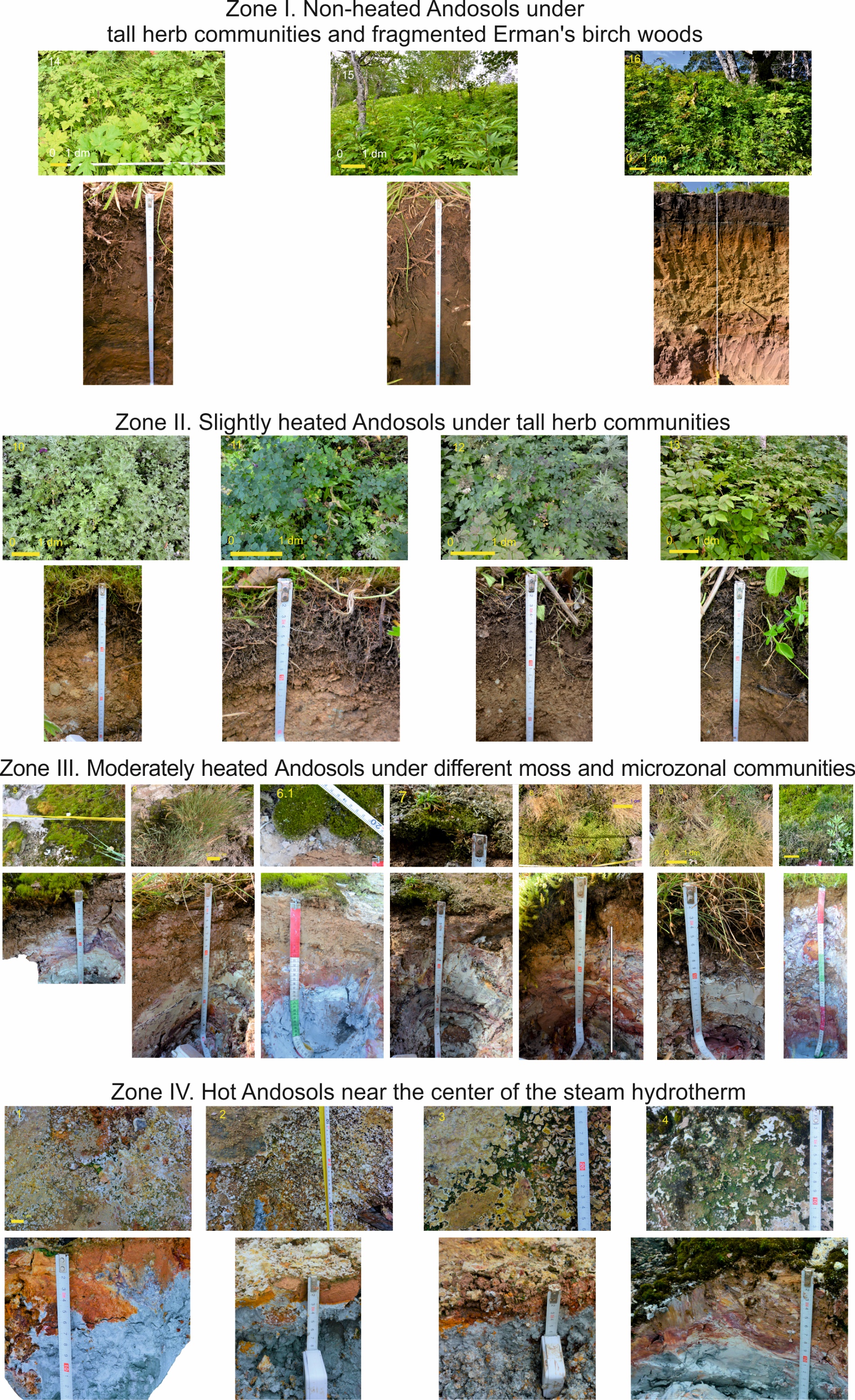


## Figure S 1. Photos of vegetation and soils studied at plots 1 – 16


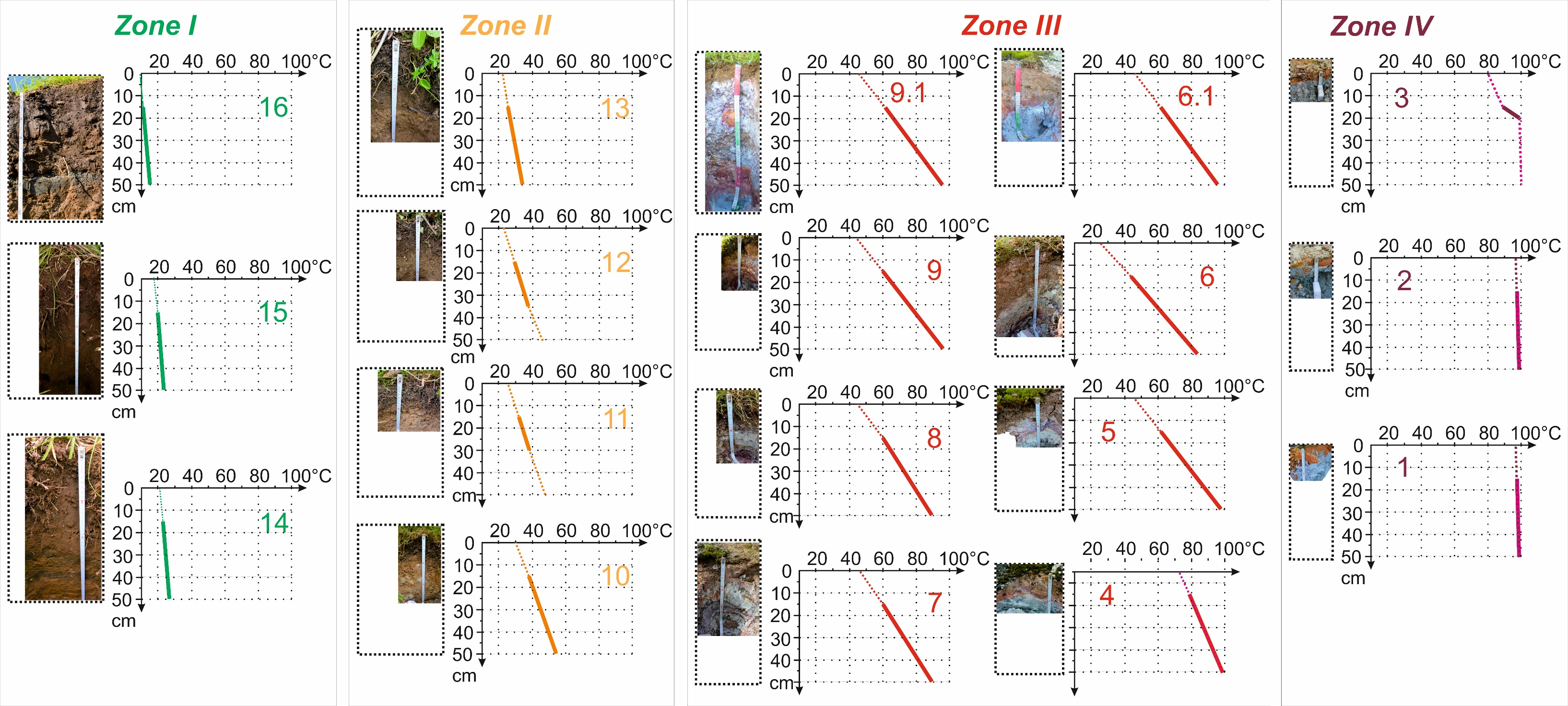


## Figure S 2. Photographs of soil pits (in graphs, numbers of cross-sections are indicated) with temperature curves based on the results of measurements (solid lines) and reconstructions (dotted lines). Photos by A.V. Zavadskaya (vegetation and soils at plots 1 – 16) and I. N. Semenkov (vegetation and soils at plots 6.1 and 9.1).


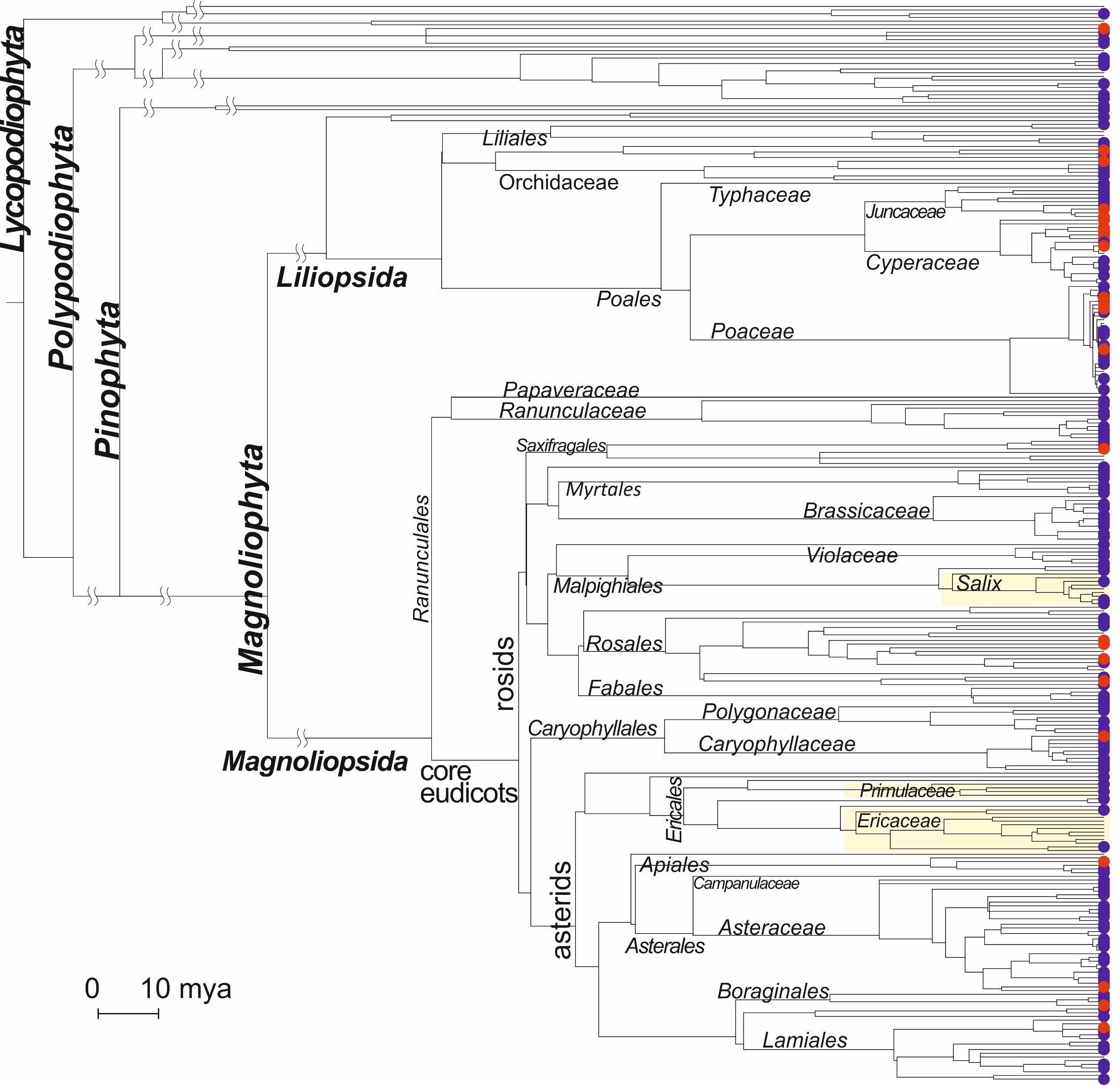


## Figure S 3. The phylogenetic tree of plants of the Geysernaya River valley (data from ^18,19^ supplemented by unpublished data by L.I. Rassokhina). Obligate and facultative thermophytes are marked by red and blue dots, respectively.

| 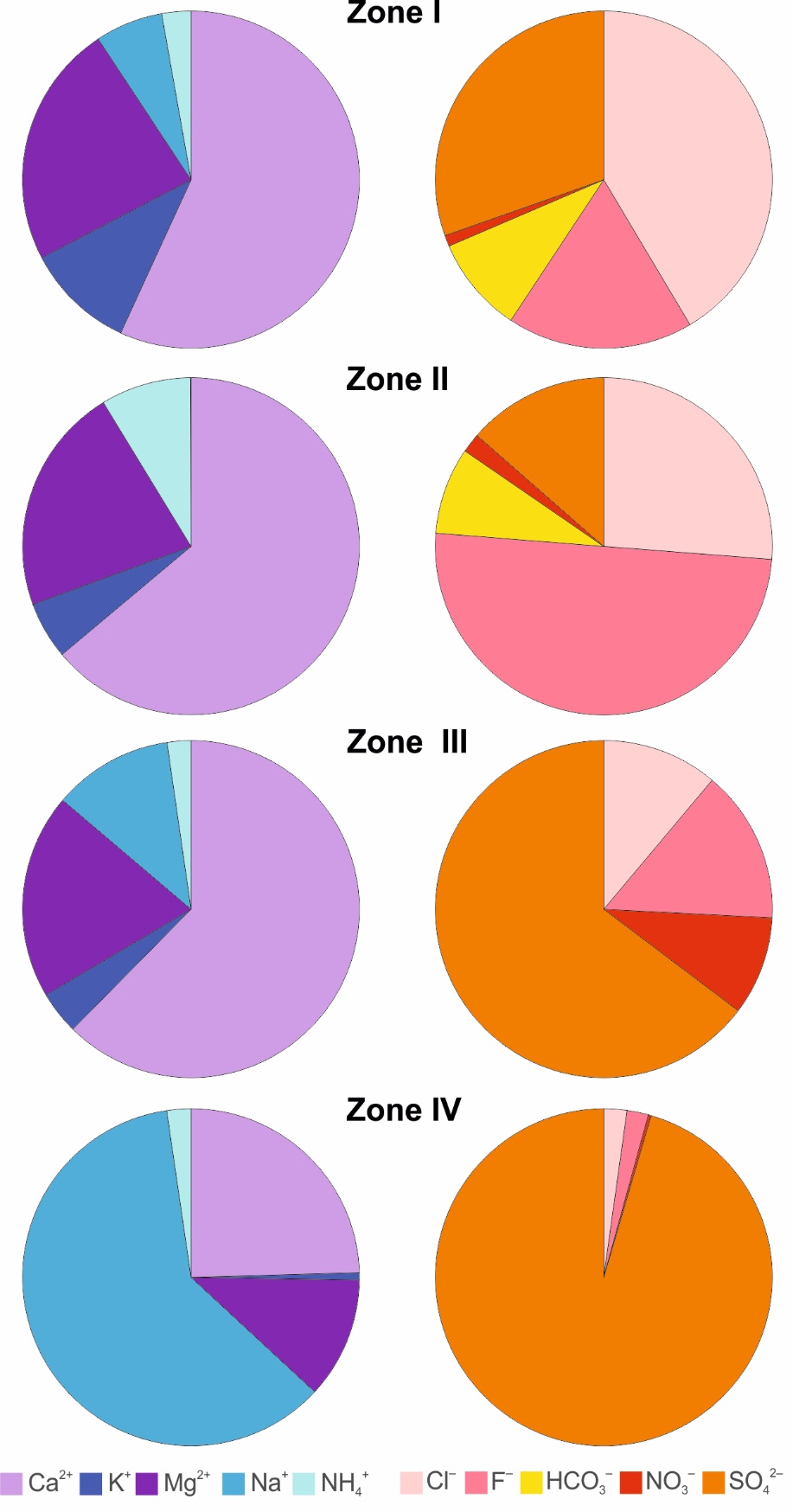 |
| --- |

## Figure S 4. Concentration of cations (left) and anions (right) in water extracts from topsoil (0 – 10 cm) of thermal zones I, II, III and IV – a, b, c and d, respectively.

## Figure S 5. Number of vascular plant species recorded in zones I – IV within the thermal spot and in its surrounding territory. Ecological groups: 1 – non-thermal species, 2 – facultative thermophytes and 3 – obligate thermophytes.


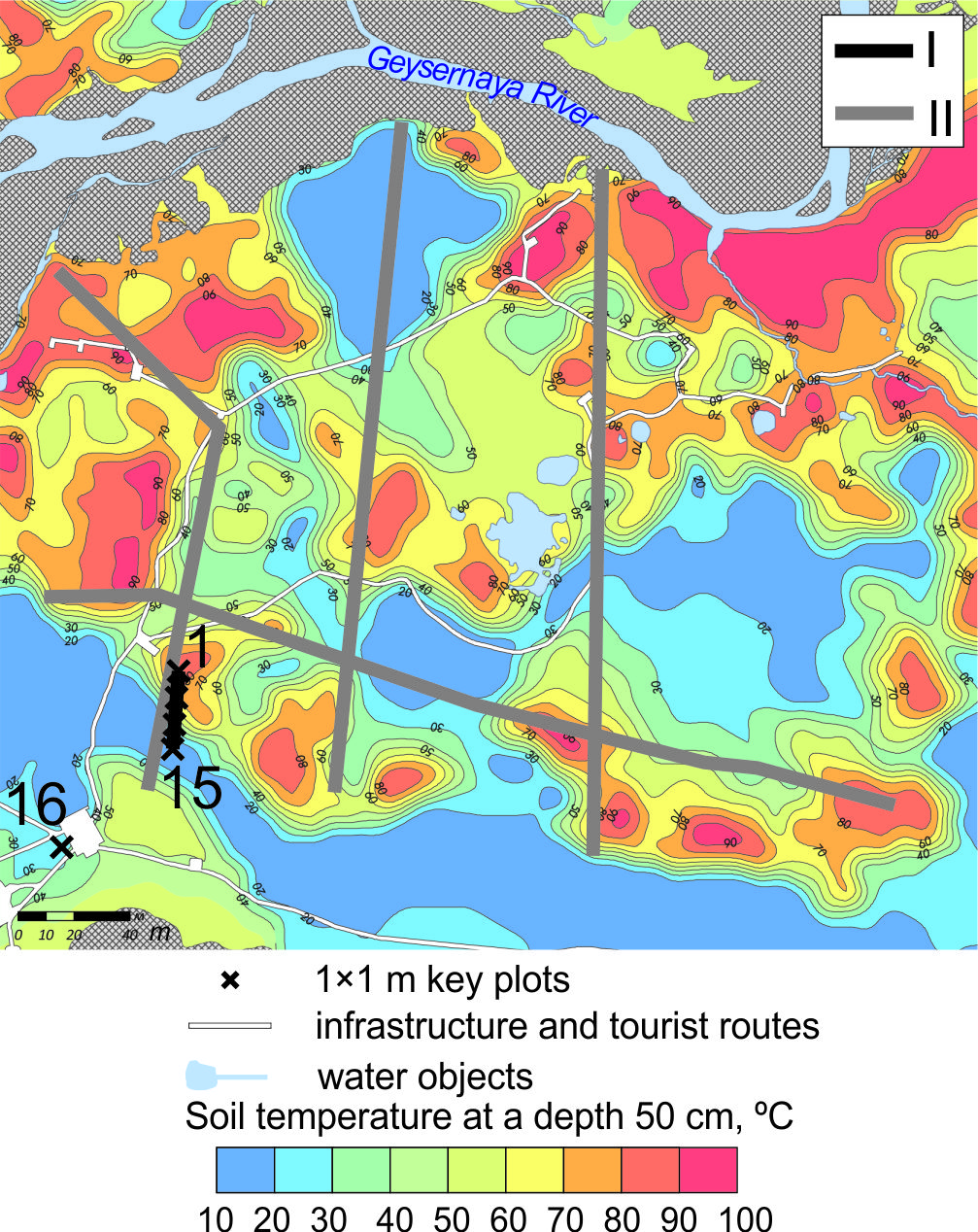


## Figure S 6. Geobotany transects: I – plots of group T (catena, 18 plots); II –plots of group B (178 plots; data by M.V. Prozorova and A.V. Zavadskaya).

The original map ^20^ (open access) was generated using strong correlations between temperature of soils and vegetation cover in the Valley of the Geysers based on description of more than 900 key plots by M.V. Prozorova, V.M. Yablokov, D.M. Panicheva, A.V. Zavadskaya ^17^. Spatial modeling is carried out in ArcGIS-10.0 software using natural neighborhood interpolation method. The final map is conducted using Adobe Illustrators tools (https://www.adobe.com/) on a scale of 1: 2000. Map projection is Universal Transverse Mercator, UTM; coordinate system – WGS-84.

## Figure S 7. Relationships between Al_ox_, Si_ox_ and Fe_ox_ content in Andosols studied and temperature.

# **Supplementary references**

1.         Kiryukhin, A., Sugrobov, V. & Sonnenthal, E. Geysers valley CO2 cycling geological engine (Kamchatka, Russia). *Geofluids* (2018) doi:10.1155/2018/1963618.

2.         Leonov, A. V. *Catalogue of the geysers of Kronotsky Reserve. The Valley of the Geysers and Caldera of Uzon volcano: history and nowadays*. (Restart Ltd., 2017).

3.         Kiryukhin, A. V., Rychkova, T. V. & Dubrovskaya, I. K. Formation of the hydrothermal system in Geysers Valley (Kronotsky Nature Reserve, Kamchatka) and triggers of the Giant Landslide. *Applied Geochemistry* (2012) doi:10.1016/j.apgeochem.2012.02.011.

4.         Leonov, A. V. History of the Cartography and Toponymy of the Valley of Geysers (Kronotsky Reserve, Kamchatka Peninsula, Russia): From Field Drawings to 3D Documents. *Cartographic Journal* (2018) doi:10.1080/00087041.2017.1414020.

5.         ISO 14235. Soil quality - Determination of organic carbon by sulfochromic oxidation. *International standard* (1998).

6.         International Standard. Soil quality — Determination of pH. *ISO 10390:2005* (2005) doi:10.1021/es0620181.

7.         Jillavenkatesa, A., Lum, L.-S. H. & Dapkunas, S. *NIST Recommended Practice Guide Particle Size Characterization*. *Nist* (2001) doi:https://doi.org/10.6028/NBS.SP.960-1.

8.         Kramer, J. R. Alkalinity and acidity. in *Inorganic Species* 85–135 (Elsevier, 1982). doi:10.1016/B978-0-12-498301-4.50008-6.

9.         Soil Survey Staff. *Kellogg Soil Survey Laboratory Methods Manual. Soil Survey Investigations Report No. 42, Version 5.0.* *Kellogg Soil Survey Laboratory Methods Manual* (2014).

10.        Blakemore, L. C., Searle, P. L. & Daly, B. K. Method for chemical analysis of soils. *New Zealand Soil Bureau Scientific Report* (1987).

11.        FAO/WRB. *World reference base for soil resources 2014*. *World Soil Resources Reports No. 106* (2014). doi:10.1017/S0014479706394902.

12.        Moore, D. M. & Reynolds, R. C. Jr. *X-Ray Diffraction and the Identification and Analysis of Clay Minerals*. *Oxford University Press* (Oxford University Press, 1997). doi:10.1017/S0016756898501501.

13.        Post, J. E. & Bish, D. L. Rietveld refinement of crystal structures using powder X-ray diffraction data. in *Modern powder diffraction. Reviews in mineralogy* (eds. Bish, D. L. & Post, J. E.) 227–308 (Mineralogical Society of America, 1989).

14.        Doebelin, N. & Kleeberg, R. Profex: A graphical user interface for the Rietveld refinement program BGMN. *Journal of Applied Crystallography* (2015) doi:10.1107/S1600576715014685.

15.        Sakharov, B. A., Drits, V. A., McCarty, D. K. & Walker, G. M. Modeling powder x-ray diffraction patterns of the clay minerals society kaolinite standards: KGA-1, KGA-1b, and KGa-2. *Clays and Clay Minerals* (2016) doi:10.1346/CCMN.2016.0640307.

16.        FAO. *World reference base for soil resources 2014 International soil classification system*. *World Soil Resources Reports No. 106* (FAO, 2015).

17.        Zavadskaya, A. v. *et al.* *Atlas of the valley of the Geyzernaya river in the Kronotskiy Reserve*. (Krasand, 2015). (In Russian).

18.        Yakubov, V. V. *Vascular plants of the Kronotsky Biosphere Reserve (Kamchatka)*. (1997). doi:(In Russian).

19.        Yakubov, V. V. & Chernyagina, O. A. *Catalog of Flora of Kamchatka (Vasculare Plants)*. (Kamchatsky Petchatnij Dvor, 2003). (In Russian).

20.        Yablokov, V. M. & Zavadskaya, A. V. GIS-modelling of thermal fields of the Valley of the Geysers (Kronotsky Preserve, Kamchatka). in *Proceedings of the International Conference “InterCarto-InterGIS 17”. Sustainable development of territories: GIS theory and practice* (2011).

21.        Zakharikhina, L. v. & Litvinenko, Y. S. Radiogeochemistry of Kamchatka soils. *Eurasian Soil Science* (2016) doi:10.1134/S1064229315090136.

22.        Litvinenko, Y. S. & Zakharikhina, L. v. Zoning and geochemical characterization of volcanic soils on Kamchatka. *Geochemistry International* (2009) doi:10.1134/S0016702909050036.
